# Malnutrition exacerbates pathogenesis of *Lutzomyia longipalpis* sand fly-transmitted *Leishmania donovani*

**DOI:** 10.1038/s42003-025-08106-8

**Published:** 2025-05-13

**Authors:** Eva Iniguez, Johannes Doehl, Pedro Cecilio, Tiago Donatelli Serafim, Caroline Percopo, Yvonne Rangel-Gonzalez, Somaditya Dey, Elvia J. Osorio, Patrick Huffcutt, Sofia Roitman, Claudio Meneses, Mara Short, Jesus G. Valenzuela, Peter C. Melby, Shaden Kamhawi

**Affiliations:** 1 Vector Molecular Biology Section, Laboratory of Malaria and Vector Research, National Institute of Allergy and Infectious Diseases, National Institutes of Health, Rockville, MD USA; 2 Vector Biology Section, Laboratory of Malaria and Vector Research, National Institute of Allergy and Infectious Diseases, National Institutes of Health, Rockville, MD USA; 3 Laboratory of Malaria and Vector Research, National Institute of Allergy and Infectious Diseases, National Institutes of Health, Rockville, MD USA; 4 Post Graduate Department of Zoology, Barasat Government College, Barasat, West Bengal India; 5 Department of Internal Medicine, University of Texas Health Science Center at San Antonio, San Antonio, TX USA; 6 Integrated Data Sciences Section, Research Technologies Branch, National Institute of Allergy and Infectious Diseases, National Institutes of Health, Bethesda, MD 20892 USA

**Keywords:** Parasitic infection, Parasite host response

## Abstract

Visceral leishmaniasis (VL) is transmitted by *Leishmania*-infected sand fly bites and malnutrition is a known risk factor in human VL. Models using sand fly transmission or malnutrition promote parasite dissemination. By investigating features of *L. donovani*-*Lutzomyia longipalpis* transmission to malnourished mice, we show that a comparable IL1-β-driven acute inflammation is maintained in malnourished (MN-SF) and well-nourished (WN-SF) sand fly-infected mice. However, parasite dissemination was more pronounced in MN-SF that had a significantly higher acute (*P* ≤ 0.001) and chronic (*P* ≤ 0.0001) splenic parasite burden compared to WN-SF. Compared to WN-SF, MN-SF exhibited chronic clinical symptoms (*P* ≤ 0.0001), neutrophilia (*P* ≤ 0.001), lymphocytopenia (*P* ≤ 0.0001), increased heme oxygenase-1 (*P* ≤ 0.001) and IL17-A (*P* ≤ 0.0001) levels, dysregulation of liver enzymes, lymph node barrier dysfunction, and augmented dysbiosis, all associated with enhanced VL severity. Combining vector-transmission and malnutrition provides an improved model to study VL pathogenesis and host defense.

## Abstract (web summary)

Vector-transmission of *Leishmania donovani* in malnourished mice produces distinct clinical symptoms in chronic visceral leishmaniasis, enhances parasite burden and dissemination, and disrupts gut microbiota.

## Introduction

Anthroponotic visceral leishmaniasis (VL) is a neglected tropical disease caused by the intracellular protozoan parasite *Leishmania donovani*, with an estimated global incidence of 50,000 to 90,000 new cases annually^1^. The disease impacts the poorest regions of the world, and it is second only to malaria as a cause of death by a parasitic infection^1^. VL is transmitted to humans when infectious metacyclic promastigotes are deposited in the skin during the blood meal of the sand fly (SF) vector. *L. donovani* disseminates from the skin to the liver and spleen causing symptoms including fever, cachexia, splenomegaly, and ultimately death in 95% of the cases if no treatment is administrated.

One third of world’s children living in developing regions such as India and East Africa, where VL is endemic, suffer from malnutrition^2^. Importantly, epidemiological studies have identified malnutrition as a risk factor for severe disease and death from VL in both children and adults^3–7^, with one study reporting an odds ratio of 5.0 and 11.0, respectively^8^. Even though most infected people remain asymptomatic, those who suffer from malnutrition, which is associated with impairment of the immune function, are at a higher risk of developing clinical VL, elevating their morbidity and mortality rates^9^.

Using a malnourished (MN) mouse model of VL, our group demonstrated that dissemination of 10^6^ intradermally-delivered *L. donovani* to visceral organs is enhanced by malnutrition^10–13^. Enhanced dissemination was driven by a significant increase in prostaglandin E_2_ production in the skin, promoting trafficking of infected CCR7^+^-monocytes to visceral organs^11–13^. Comparatively, intradermal injection of 10^5^ culture-derived *L. donovani* metacyclic promastigotes failed to disseminate in well-nourished (WN) animals, and parasite visceralization occurred only in sand fly-initiated infections via a sustained IL-1β-driven inflammatory response^14^. Collectively, these studies implicate distinct inflammatory pathways, related to malnutrition or sand fly-initiated infections, in parasite dissemination^10–15^.

Natural transmission by infected sand fly bites triggers a distinct inflammatory response^14,16^ that offers a better insight into VL pathogenesis, and provides an improved experimental model to study host immunity in response to *Leishmania* infections^15^. Moreover, the strong association of VL with poverty and malnutrition prompted us to investigate the malnutrition-*Leishmania* infection relationship following vector-transmission of the parasites in a murine model. In this study, we present a chronic mouse model representative of childhood malnutrition paired with natural transmission of *L. donovani* by infected sand fly bites. We use this model to investigate the early innate inflammatory response that mediates and promotes parasite dissemination and explore the evolution of VL pathology under more natural conditions that combines malnutrition with vector-transmission of *L. donovani*.

## Results

### Malnourished and well-nourished animals produce comparable early skin inflammatory immune responses after *Leishmania*-infected sand fly bites

To develop our model, BALB/c female mice were assigned to a polynutrient-sufficient (well-nourished, WN) or polynutrient-deficient (malnourished, MN) diet after weaning. As expected, the MN group exhibited a stable stunted body weight while WN mice significantly gained weight steadily (Fig. S1a and Supplementary Data 2 Appendix Table 166). At week 5–7 after feeding on their respective diets, WN and MN mice were challenged in both ears with 10^5^ intradermally delivered metacyclic promastigotes or via bites of 20 *L. donovani*-infected sand flies^14^. Throughout the study, maturity of sand fly infections (Fig. S1b) was assessed prior to each sand fly transmission, and experiments were only analyzed when a comparable feeding behavior was observed for WN and MN groups (Fig. S1c). Of note, unchallenged malnourished and well-nourished animals will be referred to as MN and WN, respectively, throughout the manuscript.

**Fig. 1:**
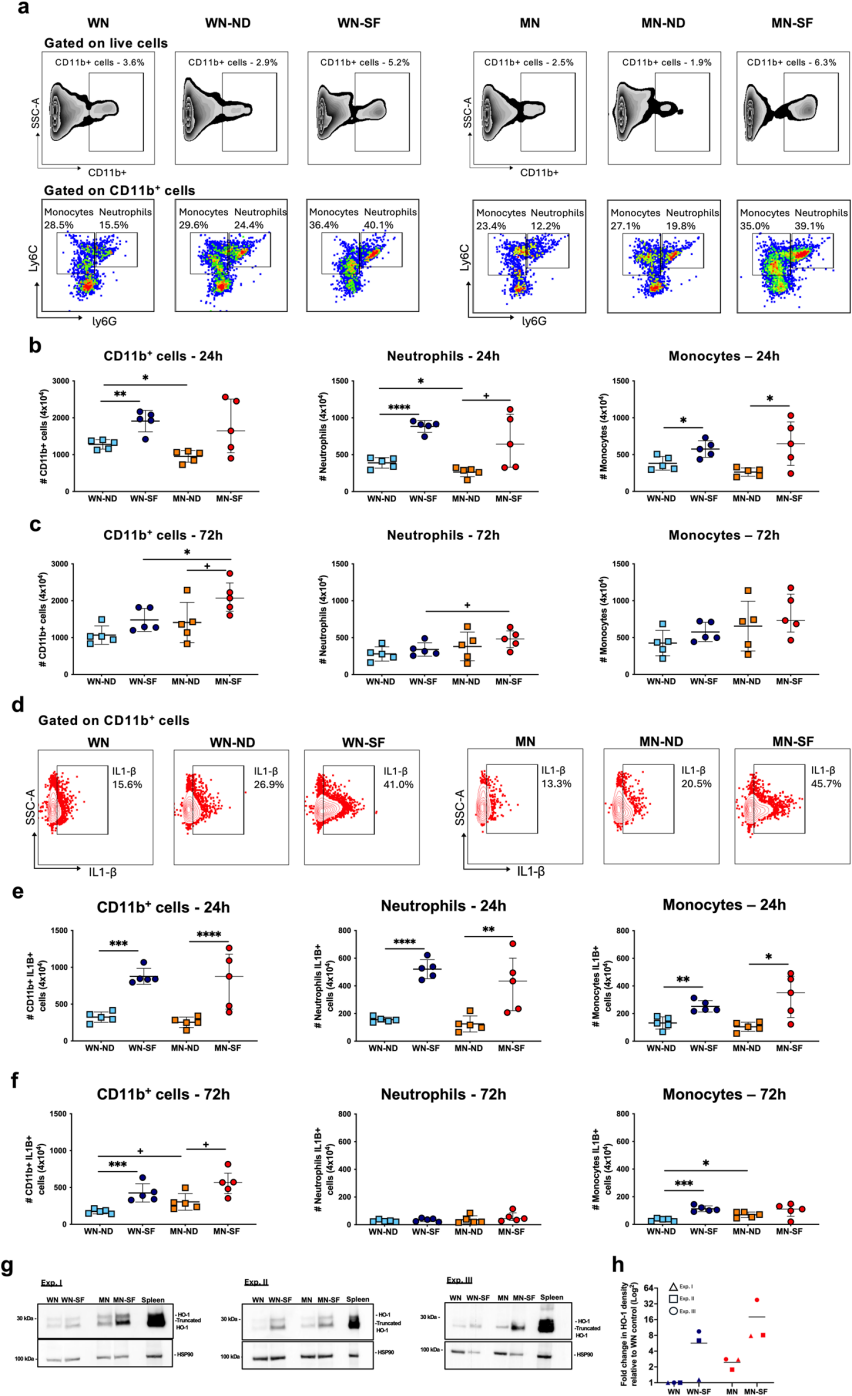
Malnourished animals produce a sustained IL1-β driven inflammatory response in ear skin following *Leishmania donovani*-infected sand fly bites. **a** Representative inflammatory infiltrate of live cells gated on CD11b+ myeloid skin cells, then neutrophils (Ly6C^+^Ly6G^+^) and monocytes (Ly6C^+^Ly6G^−^) at 24 h post-challenge. Number of CD11b^+^ cells, neutrophils, and inflammatory monocytes at 24 h (**b**) and 72 h (**c**) post-challenge. **d** Representative inflammatory infiltrate of neutrophils and inflammatory monocytes expressing IL1-β on CD11b^+^ cells at 24 h post-challenge. Number of CD11b^+^ cells, neutrophils, and monocytes expressing IL1-β at 24 h (**e**) and 72 h (**f**) post-challenge. **g** Heme oxygenase 1 (HO-1) expression in skin tissue lysate 72 h post-infected bites by Western blot. HSP90, loading control. **h** Log 2-fold change of HO-1 density relative to WN-unchallenged controls. All groups were first normalized against the HSP90 loading control from (**g**). WN well-nourished, MN Malnourished, SF sand fly, ND needle. Data are representative of two experiments, *n* = 5 animals per group, and each data point represents a pool of 2 ears per animal (**a**–**f**). **g**, **h** Western blots from three independent experiments, *n* = 5–10 pooled ears per group. Data shows the median with interquartile range (IQR) (**b**, **c**, **e**, **f**), and mean (**h**). *p* ≤ 0.05 was considered significant. ^+^*p* ≤ 0.09, *p ≤ 0.05, **p ≤ 0.01, ***p ≤ 0.001, and ****p ≤ 0.0001. Significance was calculated by pairwise comparison by linear contrast expression (**b**, **c**, **e**, **f**) and Dunn’s test (**h**). Refer to Supplementary Data 2 file for the full statistical analysis report. All experiments were blinded.

MN-SF animals exhibited the intense early inflammatory response associated with *Leishmania*-infected sand fly bites (Fig. 1). CD11b^+^ cells were recovered from mice ears (Fig. S1d) at 24 h and 72 h post-challenge. At 24 h, the recruitment of CD11b^+^ cells was 1.48-fold and 1.58-fold higher in animals infected via sand fly bites compared to needle (ND)-infected WN and MN mice, respectively (Fig. 1a, b). As previously reported^14^, this inflammation was driven by neutrophils whose number was 2.16 and 2.23 times higher in WN-SF and MN-SF mice when compared to the needle-infected groups, respectively (Fig. 1a, b). Although the overall number of CD11b^+^ cells remained higher in the MN-SF compared to the WN-SF and MN-ND groups at 72 h, the influx of neutrophils decreased in both WN-SF and MN-SF animals (Fig. 1c). Similarly, a significantly higher number of monocytes were recruited to the bite site at 24 h in WN-SF and MN-SF compared to ND-challenged animals, a phenotype that was lost by 72 h (Fig. 1b, c). Of note, no significant difference was observed in the steady state number of myeloid cells recovered from unchallenged ears of WN and MN animals (Figs. 1a and S1e).

Previously, we established the important role of IL-1β in the early stages of *L. donovani* dissemination after sand fly transmission to WN mice^14^. In the present study, we observed a robust and comparable expression of IL-1β in WN-SF and MN-SF animals at 24 h post-infected bites that was associated with a significantly higher number of IL-1β^+^ neutrophils (3.44-fold and 3.70-fold higher) and IL-1β^+^ monocytes (1.76-fold and 3.34-fold higher) compared to WN-ND or MN-ND, respectively (Fig. 1d, e). Interestingly, the number of CD11b^+^IL-1β^+^ cells remained higher in sand fly- compared to needle-infected groups at 72 h post-bites, potentially attributable to monocytes considering the observed decrease in the number of neutrophils (Fig. 1f). Of note, the number of CD11b^+^IL-1β^+^ cells was 1.60-fold higher in MN-SF compared to WN-SF animals (Fig. 1f).

Heme oxygenase-1 (HO-1), a pleiotropic cytoprotective enzyme that diminishes heme-mediated tissue damage, played a critical role in controlling skin inflammation after infected sand fly bites^16^. Western blot analyses from ear cell lysates of both WN-SF and MN-SF mice reinforced HO-1 induction after infected bites (Figs. 1g, h and S1f). Interestingly, HO-1 expression was highly upregulated in the MN-SF compared to WN-SF animals, and was produced at some level in steady state unchallenged MN ears (Figs. 1g, h and S1f), indicating, as previously reported^12^, that a basal level of inflammation may exist in MN animals that is further exacerbated by infected sand fly bites.

### Malnutrition enhances early parasite visceralization after *L. donovani*-infected sand fly bites

**Fig. 2:**
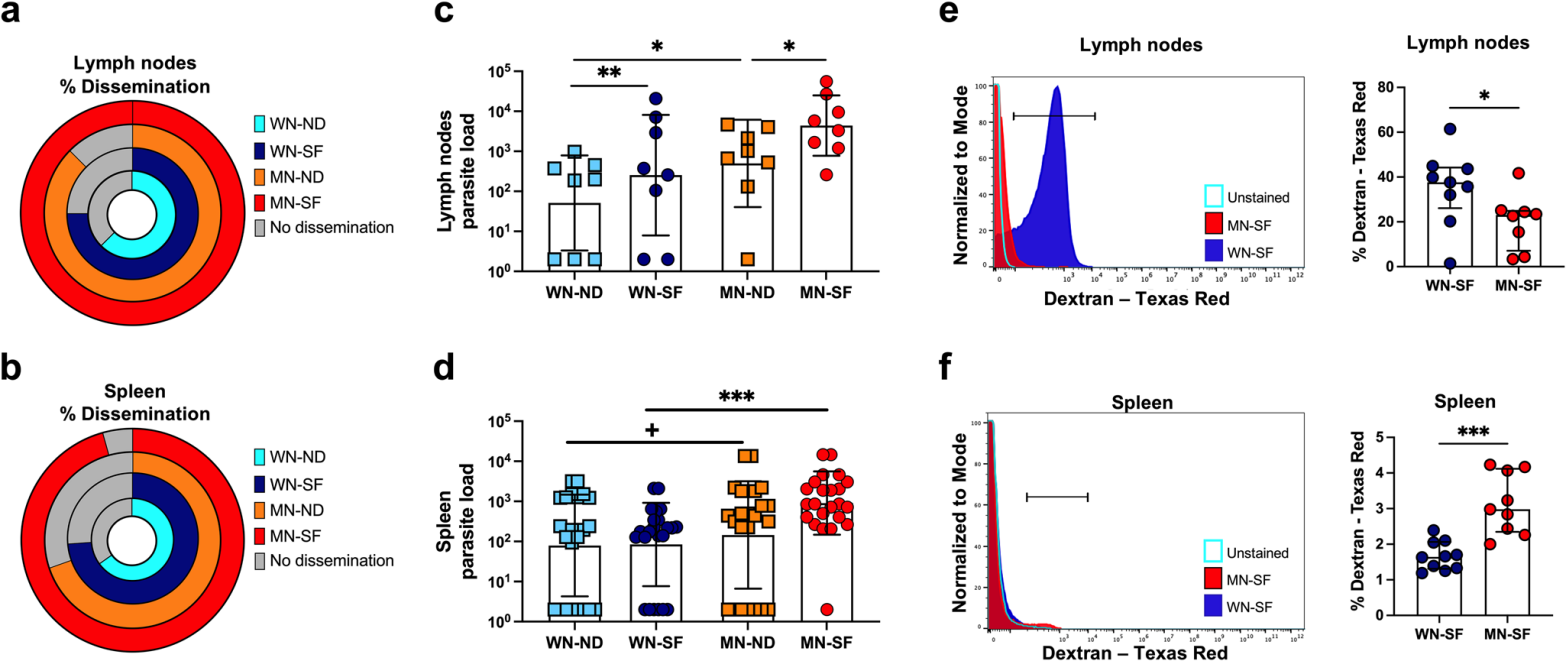
Malnourished animals exhibit early parasite visceralization after *L. donovani*-infected sand fly bites. Frequency of parasite dissemination to the lymph node (**a**) and spleen (**b**). Lymph node (**c**) and spleen (**d**) parasite load 72 h post-challenge. Accumulation of dextran-Texas red fluorescence in the lymph nodes (**e**) and spleen (**f**) of WN and MN animals at 72 h post-challenge. Flow cytometry gates were set up with unstained samples from lymph nodes and spleen collected from a WN animal injected with 1xPBS as control. WN well-nourished, MN Malnourished, SF sand fly, ND needle. Cumulative frequency of *n* = 8 animals per group (**a**), or *n* = 23 animals per group (**b**). Cumulative data of 2 experiments (**a**, **c**) and 5 experiments (**b**, **d**), *n* = 3–5 samples per group per experiment. Cumulative data of 2 experiments, *n* = 4–5 samples per group per experiment (**e**, **f**). Data shows the median with IQR (**c**–**f**); *p* ≤ 0.05 was considered significant. ^+^*p* ≤ 0.09, *p ≤ 0.05, **p ≤ 0.01, ***p ≤ 0.001, and ****p ≤ 0.0001. Significance was calculated by Fisher’s exact test (**a**), pairwise Chi-square test (**b**), and pairwise comparison based on estimated marginal means (**c**–**f**). Refer to Supplementary Data 2 file for full the statistical analysis report. All experiments were blinded.

Using qPCR for *Leishmania* detection (Fig. S2a, b), we demonstrated that at 72 h post-infection, transmission by *L. donovani*-infected sand fly bites induced a higher frequency of parasite dissemination from the skin to the lymph nodes and spleen in MN-SF animals (100% and 95.7%, respectively), as compared to WN-SF (75.0%, 73.9%), MN-ND (87.5%, 60.6%), and WN-ND (62.5%, 65.2%) animals (Fig. 2a, b). Of note, the MN-SF group showed a significantly higher odds ratio (8.32, *p* = 0.047) of parasite dissemination to the spleen when compared to the MN-ND group (Supplementary Data 2, Appendix Table 65). Furthermore, the parasite load in the lymph nodes was significantly higher in WN-SF and MN-SF compared to WN-ND and MN-ND animals, respectively (Fig. 2c), potentially promoted by the acute inflammatory burst caused by vector-transmission. However, MN groups displayed a higher splenic parasite burden compared to their WN counterparts (Fig. 2d) indicating that malnutrition is the main driver of parasite growth in the spleen in the long-term.

As previously reported^13^, enhanced dissemination of *L. donovani* to the spleen in MN animals may have been facilitated by a dysfunction of lymph node barrier. To assess lymph node barrier function, we injected WN-SF and MN-SF animals intradermally with a fluorescently labeled 10,000 kDa-Dextran 72 h after infection by sand fly bites and immediately collected the draining lymph nodes and spleen of infected animals for Flow cytometry analysis (Fig. 2e, f). When compared to WN-SF animals, the accumulation of dextran was significantly decreased in the lymph nodes and significantly increased in the spleen of MN-SF animals confirming that the lymph node barrier in MN animals is disrupted (Fig. 2e, f).

### Malnourished *Leishmania*-infected mice exhibit clinical symptoms and exacerbated pathogenicity in chronic VL

**Fig. 3:**
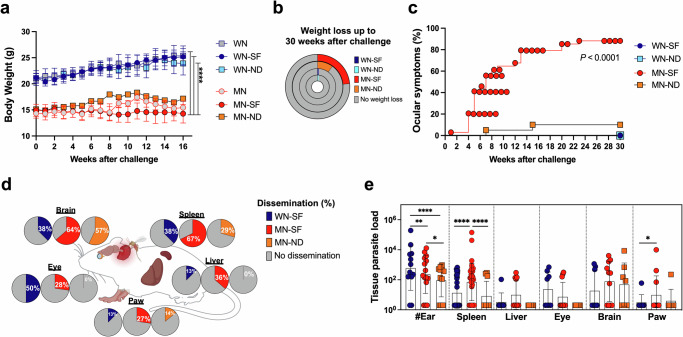
Sand fly-infected malnourished animals exhibit a chronic distinct VL pathogenicity and enhanced parasite dissemination. **a** Animal body weight up to 16 weeks after challenge. **b** Percent of animals reaching 20% of body weight loss up to 30 weeks after challenge. **c** Percent of animals showing ocular symptoms. **d** Frequency of animals with parasite dissemination to the spleen, liver, eye, brain, and paw. **e** Tissue parasite load by qPCR up to 30 weeks after challenge. #Ear site of infected bites, WN well-nourished, MN Malnourished, SF sand fly, ND needle. Cumulative data of 2 experiments. *n* = 4–5 animals per group (**a**). Cumulative data of 5 experiments. *n* = 4–5 animals per group (**b**, **c**). Cumulative data of 2–5 experiments. *n* = 4–5 animals per group (**d**, **e**). Data shows the mean with 95% CI (**a**), and the median with IQR (**e**). *p* ≤ 0.05 was considered significant. *p ≤ 0.05, **p ≤ 0.01, and ****p ≤ 0.0001. Significance was calculated by pairwise comparison by Linear Contrast Expression (**a**), pairwise Fisher’s Exact test (**b**), pairwise Log-rank Mantel–Cox test (**c**), Fisher’s Exact test or Chi-square for the spleen (**d**), and pairwise comparison based one estimated marginal means (**e**). Refer to Supplementary Data 2 file for the full statistical analysis report. All experiments were blinded.

To investigate if host susceptibility and severity of infection can be aggravated by prolonged malnutrition, we developed a chronic model of infection in MN mice and followed the course of infection for up to 30 weeks post-challenge. After challenge, MN animals maintained a significantly stunted weight compared to WN groups in unchallenged, needle-, and sand fly-infected groups (Fig. 3a, *p* ≤ 0.0001, Supplementary Data 2 Appendix Table 95). Within a period of 30-weeks post-challenge, 23.5% of MN-SF compared to only 10.0% of MN-ND animals reached the study endpoint, losing 20% of their body weight (Fig. 3b). Strikingly, 88.2% of MN-SF mice also exhibited clinical symptoms, including ocular (Fig. S3a), observed as early as week 5 post-transmission (Fig. 3c, *p* ≤ 0.0001). In contrast, only 10% of the MN-ND animals and none of the WN challenged animals portrayed these symptoms (Fig. 3a–c). Of note, malnutrition was a higher predictor of ocular pathology than sand fly transmission (Supplementary Data 2 Appendix Table 103). Odds ratios indicate that MN-SF mice were about 133.6 times more likely develop ocular pathology than WN-SF mice (*p* ≤ 0.0001), while mice infected by sand fly bites were 14.8-times more likely to developed ocular pathology compared to needle-infected mice (*p* ≤ 0.0001).

The distinct clinical profile observed in chronically infected mice was further reinforced by a generally higher proportion of parasite dissemination to internal organs in MN-SF-infected mice compared to either WN-SF- or MN-ND-infected groups (Fig. 3d). Moreover, the parasite burden was significantly higher in the spleen of MN-SF compared to WN-SF- or MN-ND groups, and in the paw of MN-SF compared to WN-SF animals (Fig. 3e). Interestingly, parasite persistence at the infection site in the ear was observed for all groups (Fig. 3e). Notably, the parasite burden in the ear was significantly higher in WN-SF compared to MN-SF mice, and both groups had a significantly larger number of parasites than the MN-ND group (Figs. 3e and S3b). The difference in the weight of organs between groups was only observed between WN and MN groups for the spleen and was less pronounced for the liver (Fig. S3c). Overall, our results indicate that chronic malnutrition leads to a distinct exacerbated VL pathogenicity that is only observed when MN mice are infected via sand fly bites.

### Malnourished animals infected by sand fly bites exhibit enhanced systemic inflammatory and metabolic biomarkers in chronic VL

**Fig. 4:**
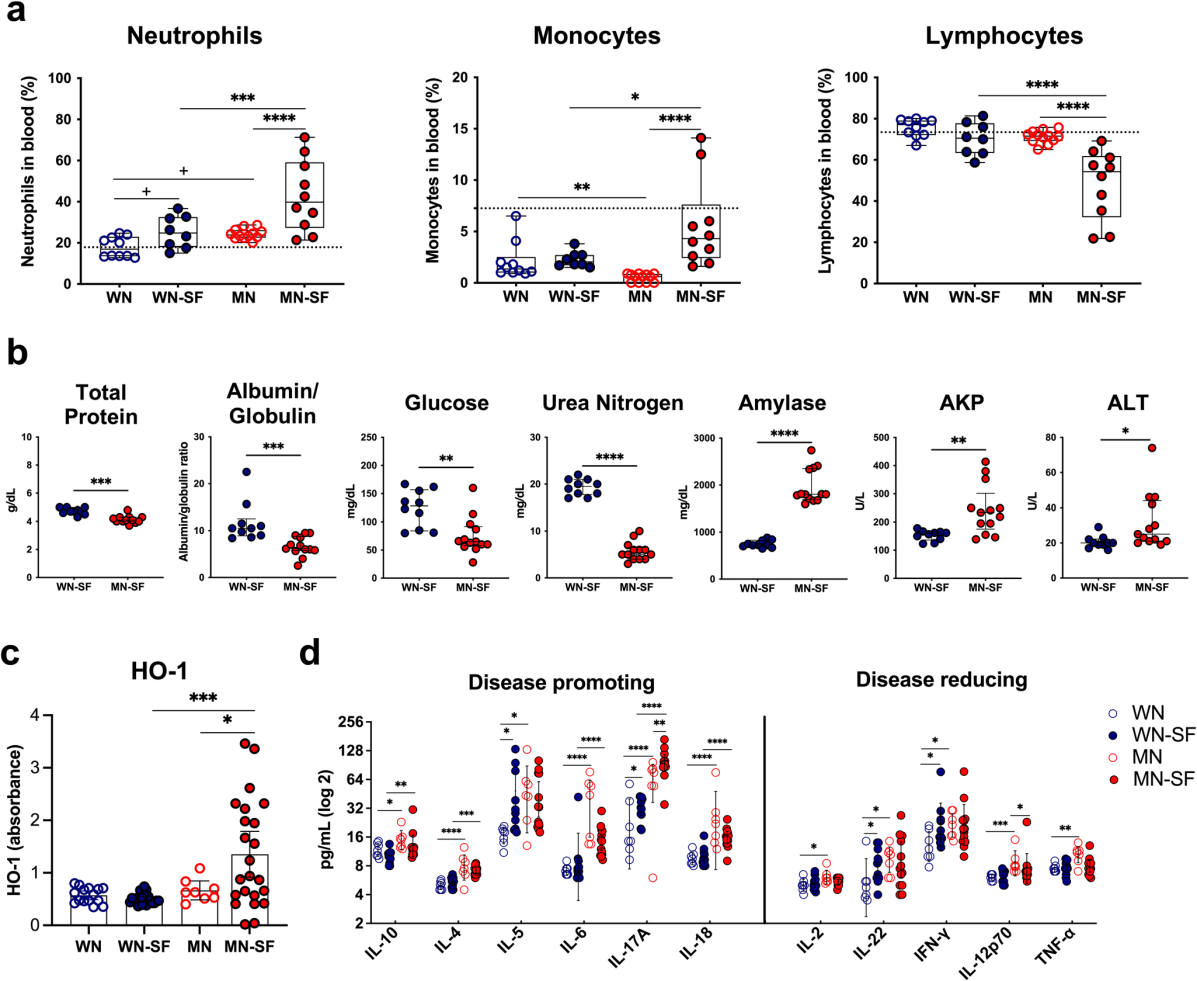
Systemic inflammatory and metabolic mediators are altered in sand fly-infected malnourished animals in the chronic stage of VL. **a**–**d** WN and MN animals were followed up to 30 weeks post-infected sand fly bites. **a** Percent of circulating neutrophils, monocytes, and lymphocytes, **b** Serum chemistry panel analytes, **c** Serum HO-1 levels, and **d** Serum cytokine levels. Unchallenged WN and MN animals were included as baseline controls. Dotted line in (**a**), hematology normal reference levels in unchallenged mice^18^. WN well-nourished, MN Malnourished, SF sand fly challenge. Cumulative data of 2 (**a**, **b**, **d**, **e**) or 4 (**c**) independent experiments, *n* ≥ 3 animals per group. Data shows the mean with min and max points (**a**), median with IQR (**b**), and mean with 95% CI (**c**, **d**). *p* ≤ 0.05 was considered significant. ^+^*p* ≤ 0.09, *p ≤ 0.05, **p ≤ 0.01, ***p ≤ 0.001, and ****p ≤ 0.0001. Significance was calculated by pairwise comparison based on estimated marginal means (**a**, **c**, **d**), and estimated marginal means for total protein, albumin, globulin, glucose, and urea nitrogen or linear contrast expression for ALT, ALP and amylase (**b**). Refer to Supplementary Data 2 file for the full statistical analysis report. All experiments were blinded.

Next, we wanted to investigate how VL biomarkers are altered by malnourishment after natural infection by sand flies. We performed a hematological analysis from blood of animals followed up to 30 weeks after *L. donovani* infection. The percent of circulating neutrophils increased significantly due to malnourishment and was highest in the MN-SF group (Fig. 4a). Additionally, all MN-SF animals showed values above the reported normal counts of ~17% for neutrophils in circulation^17,18^. Similarly, MN-SF mice had the highest percent of circulating monocytes, that showed a significant 2.44-fold increase compared to WN-SF animals (Fig. 4a). Conversely, the MN-SF group exhibited a significant decrease in the percent of lymphocytes when compared to other groups (Fig. 4a), with some MN-SF animals displaying values below the normal reference count of 70–80% for lymphocytes in circulation^17,18^.

We also carried out a comprehensive metabolic panel analysis which showed that MN-SF infected mice had a significantly lower median value of total serum protein, albumin/globulin ratio, glucose, and urea nitrogen, while exhibiting significantly higher levels of amylase, alkaline phosphatase (AKP), and alanine aminotransferase (ALT) liver enzymes than the WN-SF group (Fig. 4b). Only urea nitrogen and AKP were significantly altered in the MN-SF infected group when compared to MN unchallenged animals (Fig. S4). Though not significant, levels of ALT were also increased in MN-SF animals after infected vector bites (Fig. S4).

We next measured the circulating levels of HO-1, associated with clinical VL in humans^19^, and induced by prolonged bleeding at the bite site^16^. Mirroring our findings at the bite site, we detected a significant upregulation of HO-1 serum levels in MN-SF animals but only after infection via sand fly bites (Fig. 4c).

We also noted that the serum levels of a panel of 11 cytokines were significantly increased in unchallenged MN compared to WN animals supporting the existence of a basal level of inflammation in the former (Fig. 4d). More so, after challenge by infected sand fly bites, the levels of IL-10, IL-4, IL-6, IL17-A, and IL-18 cytokines were significantly higher in MN-SF animals when compared to the WN-SF group. Interestingly, a significant 1.78-fold increase of IL17-A was exhibited by MN-SF animals when compared to MN animals (Fig. 4d). Of note, when comparing WN to WN-SF, the levels of IL-5, IL-22, IFN-γ, and IL-17A were significantly increased while a decrease was observed for IL-10 (Fig. 4d). Additionally, the levels of IL-6 and TNF-α were significantly reduced in MN-SF compared to MN mice. The overall observed outcome shows that circulating mediators involved in inflammation and pathogenicity were enhanced in our chronic model of VL, resulting from the combined effect of malnutrition and transmission via infected sand fly bites.

### Dysbiosis of intestinal microbiota is observed in chronically malnourished animals infected with *L. donovani* by sand fly bites

**Fig. 5:**
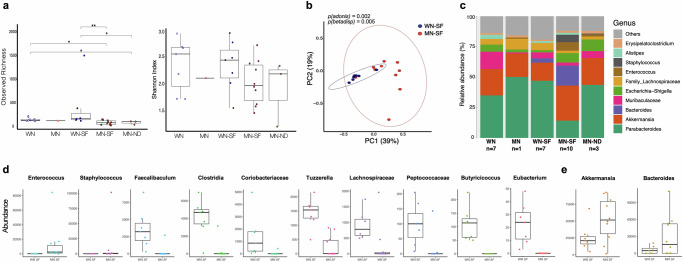
Enhanced gut dysbiosis is observed in sand fly-infected malnourished animals in the chronic stage of VL. **a**–**e** Microbes in fecal matter from animals at baseline (WN and MN), after infected vector bites (WN-SF and MN-SF), or needle challenge (MN-ND) were compared. **a** Box plots showing the alpha diversity among groups calculated using observed richness and Shannon index values. **b** Principal coordinate analysis (PCoA) plot showing the beta diversity of WN-SF and MN-SF animals generated using a Bray–Curtis distance matrix. **c** Relative abundance bar plot of the top ten genera across groups. **d** Raw abundance box plots showing the top statistically significant differentially abundant bacteria for WN-SF compared to MN-SF. **e** Raw abundance plots for Akkermansia and Bacteroides, enriched in some MN-SF animals, but not significantly. Cumulative data of 2 experiments, *n* ≥ 3 animals per group, except for MN baseline where only one sample was analyzed. Data shows the mean with min and max points (**a**, **d**, **e**); *p ≤ 0.05 was considered significant, calculated by Wilcoxon test (**a**); differential abundance and significance were calculated using the ANCOM-BC tool (FDR adjusted *p* value ≤ 0.05) (**d**, **e**). All experiments were blinded.

To explore whether the gut microbiome of chronically MN animals would be influenced by vector-transmitted *L. donovani*, we performed 16S rRNA sequencing of fecal matter. Using observed richness values, the alpha diversity in MN animals infected by needle (MN-ND), or sand fly bites (MN-SF) was significantly lower than in WN animals infected or not by sand fly bites (Fig. 5a). The same pattern was observed in the context of Shannon Index values, but without a significant difference between groups (Fig. 5a). When vector-initiated infections were compared by Bray–Curtis PCoA, results indicated a significant difference in microbial community composition and dispersion between WN-SF and MN-SF animals (Fig. 5b, *p(adonis)* = 0.002*, p(betadisp)* = 0.005). This is further reinforced by a clear shift in the microbiome composition of the MN-SF group compared to all other groups when considering the relative abundance levels of the top 10 genera (Figs. 5c and S5a). We identified ten statistically significant differentially abundant genera between MN-SF and WN-SF (Figs. 5d and S5b). More specifically, bacteria belonging to the *Enterococcus* and *Staphylococcus* genera were found to be differentially enriched in the MN-SF group, exhibiting a 4.33-fold and 4.7-fold increase compared to WN-SF animals, respectively. The other eight families and genera (Faecalibaculum, Clostridia, Coriobacteriaceae, *Tuzzerella*, Lachnospiraceae, Peptococcaceae, *Butyricicoccus*, and Eubacterium) were differentially enriched in the WN-SF group and contracted in the MN-SF mice (Figs. 5d and S5b). Though the genera *Akkermansia* and *Bacteroides* appear to be enriched in some of the MN-SF animals, this difference was not significantly different when compared to WN-SF animals (Figs. 5e and S5a, b). Of note, we identified gut-associated phenotypic changes, namely fecal discoloration and bloating of digestive organs such as the stomach and intestines, in chronically malnourished animals. Collectively, these data demonstrate that nourishment is a significant driver of microbial community diversity and malnutrition renders animals more susceptible to dysbiosis even after vector challenge.

## Discussion

The strong association between VL, poverty, and malnutrition^9,20^ prompted us to investigate a murine model of VL that mirrors childhood malnutrition paired with natural transmission of *L. donovani* by sand fly bites. Developing a VL model that combines both sand fly-initiated infections and malnutrition provides the opportunity to interrogate the contribution of each to disease evolution and enhanced pathogenicity. Interestingly, we observed a clear dichotomy of responses that were mainly driven by either vector-transmission (route of infection) or malnutrition (diet). Vector-transmission was the strongest predictor of early cellular recruitment, while malnourishment had a greater influence on increased parasite burden.

We had previously determined that an acute inflammatory response elicited at the bite site by vector-derived factors promotes parasite dissemination^14^. Our data comparing well-nourished mice, provided a nutrient-rich diet containing similar components to a standard diet commonly used in laboratories, to malnourished animals, fed on a polynutrient deficient diet, infected via needle or sand fly bites, show that vector-initiated infections produce a strong and sustained inflammatory response characterized by an early influx of IL-1β-secreting neutrophils, high expression of HO-1, and enhanced early parasite dissemination from the skin to the spleen, supporting previous findings^14,16^.

To our knowledge, parasite dissemination from the skin to the spleen or other internal organs has been described only after exposure to infected *L. donovani* sand fly bites^14^ or after infection with a high (≥10^6^) parasite inoculum in both malnourished and well-nourished host^11,13,21,22^. In our study, malnourished sand fly infected mice experienced early dissemination of parasites to the lymph nodes and spleen more frequently than the well-nourished sand fly inoculated, malnourished needle inoculated, or well-nourished needle inoculated mice. The latter had the lowest frequency of dissemination, indicating that parasite inoculation by sand fly bite increases the frequency of parasite dissemination, and that in the context of sand fly inoculated parasites, malnourishment further exacerbates the frequency of parasite dissemination. It is worth noting that despite observing a similar infiltration of inflammatory monocytes and neutrophils to the infected bite site of MN-SF and WN-SF animals, it has been recently demonstrated that these cells are more efficient at capturing *Leishmania* parasites in malnourished compared to well-nourished hosts^12^.

By following the evolution of VL up to 30 weeks, we observed that in the chronic stage of the infection, *Leishmania* parasites disseminate to other tissues such as paws, brain, and eyes, observed in all the groups apart from well-nourished needle inoculated mice. Again, the malnourished sand fly infected mice exhibited the highest frequency of parasite dissemination to various organs reinforcing the potency of vector-transmission of *Leishmania* and its enhancement by malnourishment. Studies reporting on finding parasites in the brain of naturally *Leishmania* infected dogs^23,24^, and in mice after intraperitoneal injection with 5 × 10^7^ *L. donovani*^22^, support our findings and highlight the ability of *Leishmania* parasites to cross the blood brain barrier.

We previously reported that parasites persist at bite sites in the skin following experimental vector transmission^25,26^. In the present study, *Leishmania* parasites were recovered from the site of infection up to 30 weeks post-challenge in both malnourished and well-nourished sand fly inoculated animals, and in significantly lower numbers in malnourished needle inoculated mice. Persistence of *Leishmania* parasites in the skin may contribute to infection of vector populations, maintaining and expanding disease foci. Additionally, there is evidence that *Leishmania* parasites favor tropism to the skin, forming patchy parasitized areas in immunodeficient *RAG2*^−^/^−^ mice infected intravenously with *L. donovani*^27^. The implication of parasite dissemination to multiple organs over the long-term, and their persistence in the skin warrants further investigation to determine how these sub-populations contribute to VL epidemiology and transmission in a natural setting.

It is well-established that transmission of parasites by vector bites enhances *Leishmania* pathogenesis^15,28,29^, and vector-transmission of *L. donovani* leads to progressive VL in hamster and dog models of infection^25,26^. However, to our knowledge, this is the first study in which the majority (88.2%) of animals in a murine model of infection with *L. donovani* develop clinical symptoms after parasite delivery by sand fly bites. Only by combining the delivery by vector bites with malnutrition, both present in a natural scenario, did we observe chronic clinical symptoms such as weight loss and ocular distress. Interestingly, eye symptoms have been reported in human VL^30–32^, suggesting that our model is reproducing natural symptoms. In concordance with other studies^12,33^, we hypothesize that the prolonged and sustained basal local and systemic inflammatory milieu that prevails in malnourished animals contributes to enhanced susceptibility to VL, which is further exacerbated by the potency of infected sand fly bites.

Chronic systemic immune-inflammation have been linked to neutrophilia and lymphocytopenia, increased levels of IL-17A, and the development of a leaky gut^33–37^. Furthermore, immune-inflammation and an increased neutrophil to lymphocyte ratio were correlated with enhanced severity of VL in humans and dogs^35,37,38^. In this study, we observed an increased number of circulating neutrophils combined with lymphocytopenia in malnourished sand fly inoculated mice. Additionally, these mice showed the highest levels of IL-17A among all tested groups and exhibited an altered hematological profile similar to changes observed in human VL^39–44^. Furthermore, the malnourished sand fly infected mice exhibited a reduction in total protein and in the ratio of albumin/globulin, and an increase in liver enzyme levels including ALT and AKP. High levels of metabolic biomarkers such as AST, ALT, and AKP have been documented in VL patients from India^42^, Sudan^41,43^, and Ethiopia^40,44^, and a low albumin/globulin ratio, high urea, high ALT, and high creatine have been observed in clinical naturally infected dogs^45^. AKP has also been reported in high inflammatory environments, indicative of liver damage^46^. These findings reinforce the enhanced susceptibility to VL in long-term heightened inflammatory states present in malnourished sand fly infected mice.

There is evidence that acute malnutrition and systemic inflammation are associated to intestinal barrier dysfunction and changes in microbiota, and are characterized by a leaky gut, neutrophilia, and increased circulatory levels of IL1-β and IL17-A^33–36^, all observed in malnourished sand fly infected mice in our study. During malnutrition, the intestinal microbiota undergoes dramatic changes in community composition, and exhibits altered diversity^33,47^. In this study, we observed a loss of microbial diversity in malnourished compared to well-nourished sand fly infected mice. Furthermore, we observed an expansion of Bacteroides and Enterococcus in the malnourished sand fly infected mice that have been reported to play a role in inflammation and pathogenesis^48–50^, inflammatory and autoimmune diseases^50–52^, and in severity of cutaneous leishmaniasis^53,54^. Our study establishes the long-term loss of microbial diversity in malnourished compared to well-nourished mice after challenge by infected vector bites. Future studies that expand on these preliminary observations are needed to fully elucidate the impact of microbial dysbiosis on the progression of VL.

In this study we have pioneered the development of a chronic animal model of infection that combines malnutrition, a major risk factor for VL, and vector-transmission of *L. donovani*. This model provides great advantages in the study of VL pathogenesis and host defense, including the response to drugs and vaccines against *Leishmania* infections. It can also shed more light into the interaction of host- and vector-mediated determinants of parasite dissemination and establishment, bringing us closer to understanding how VL occurs in endemic regions.

## Methods

### Animals

Three-week-old female BALB/c mice were purchased from Jackson Laboratories. Four to 6-week-old male Golden Syrian hamsters were purchased from Harlan Laboratories. Animals were housed under pathogen-free conditions at the National Institute of Allergy and Infectious Diseases (NIAID) Twinbrook animal facility in Rockville, Maryland. All animal experiments were approved by NIAID Animal Care and Use Committee under the LMV23E and LMVR4E animal protocols. The NIAID DIR Animal Care and Use Program complies with the Guide for the Care and Use of Laboratory Animals and with the NIH Office of Animal Care and Use and Animal Research Advisory Committee guidelines. We have complied with all relevant ethical regulation for animal use.

### Mice diet

Post-weaning, 3-week-old BALB/c mice were assigned randomly to a diet group. Animals assigned to the malnourished (MN) group were fed for a minimum of 4 weeks with a polynutrient deficient diet, containing 3% protein, and low iron (10 ppm), and zinc (1 ppm) (TD.99075, Envigo) until stunted weight was observed as described^11^ prior to initiation of experiments. In parallel, animals assigned to the well-nourished (WN) group were fed with a nutrient-rich diet containing 16.9% protein, normal zinc (30 ppm), and iron (100 ppm) (TD.99103, Envigo), containing similar nutritional components (19.5% protein, 81 ppm zinc, and 360 ppm iron) as a standard diet commonly used in laboratories^55^. MN mice received 90% volume by weight of food compared to the WN mice. Mice diet was supplied every day at the same time frame. Animal weight was recorded every week during the experiment. According to the LMV23E animal protocol, infected mice were euthanized by the investigator at a loss of 20% or more of pre-infection weight, the study endpoint.

### Sand fly infections

Four to 6-day-old *Lutzomyia longipalpis* (Jacobina colony) female sand flies were artificially infected as described previously^14^. Rabbit defibrinated blood (Noble Life Science) was spiked with 5 × 10^6^ *Leishmania donovani* (MHOM/SD/62/1S) amastigotes per mL and allowed the sand flies to feed for 2 h in the dark. Amastigotes were isolated from a sick *L. donovani* infected hamster. After infection, fed flies were sorted out and were maintained with 30% sucrose at 26 °C with 75% humidity with 12 h light/dark cycle until transmission. Prior to transmission, the maturity of the infection was assessed by counting the total number of parasites per midgut and the percent of metacyclic promastigotes from infected sand fly midguts (*n* = 5–10) at day 8–12 post-infection.

### Mice anesthesia

Ketamine (100 mg/kg) with xylazine (10 mg/kg) were used to anesthetize mice by intra-peritoneal administration. Eyes were kept moist by the application of LubriFresh ophthalmic ointment (Major Pharmaceuticals) to prevent dryness.

### Transmission by vector bites

Briefly, 20 infected *L. longipalpis* sand flies were applied to a mouse ear and allowed to feed for 1–2 h in the dark. The number of fed sand flies per ear were determined post-transmission.

### Needle challenge by intradermal injection

Parasites were maintained in Schneider’s media (Gibco) supplemented with 20% fetal bovine serum (Thermo Fisher Scientific) and kept at 26 °C until metacyclic promastigotes developed at day 4–5. Culture metacyclic promastigotes were purified using a Ficoll gradient and 10^5^ parasites in a volume of 10 μl were injected intradermally in the ear pinnae of mice^14^.

### Flow cytometry

At 24 h and 72 h post-infection, mice ears were collected and processed as previously described^16^. Briefly, single cell suspensions were stained with the LIVE/DEAD™ fixable Aqua dead cell marker (Thermo Fisher Scientific, L34957, 1:2000 dilution), anti-mouse CD16/32 (TruStain FcX, clone 93; Biolegend, Cat No. 101320, 1:50 dilution), PE-Cy7 CD11b (clone: M1/70; Biolegend, Cat No. 101216), Alexa Fluor 700 Ly6C (clone HK1.4; Biolegend, Cat No. 128024), and PerCP-eFluor 710-Ly6G (clone: 1A; eBioscience, Cat No. 46966882) for 30 min. Cells were fixed (Biolegend, Cat No. 420801) and permeabilized (Biolegend, Cat No. 421002), followed by intracellular staining with APC IL1-β pro-form (clone: NJTEN3; eBioscience, Cat No. 17-7114-80) overnight. All antibodies were used at 1:100 dilution. All cells were acquired using MACSQuant 16 (Miltenyi Biotec) and data analyzed using FlowJo 10 software. Gates were established using Fluorescence Minus One (FMO) and unstained controls.

### HO-1 Western blot—skin lysate

Western blot from heme oxygenase (HO-1) was performed as described previously^16^. Briefly, mouse ear lysate was collected as previously described^14^ 72 h after transmission, and 30 μg of protein lysate was loaded in NuPAGE 4%–12% Bis-Tris protein precast gels (Thermo Fisher Scientific) and transferred to an iBlot 2 Transfer Stack 0.45 mm nitrocellulose membrane using the 7 min standard protocol in the iBlot 2 Gel Transfer Device (Thermo-Fisher Scientific). After transfer, the membrane was incubated with Rabbit anti-HO-1 antibody at 1:100 dilution (abcam, Cat No. ab13243), Rabbit anti-HSP90 (Cell Signaling Technology, Cat No. 4877) at 1:200 dilution, and Goat anti-rabbit secondary antibody conjugated to horseradish peroxidase (HRP) (Cell Signaling Technology, Cat No. 70745) at 1:1000 dilution, following the iBIND apparatus instructions (Invitrogen). After 3 h of incubation, the membrane was developed using the Flash Western Chemiluminescence Kit (Azure Biosystems), and the signal was captured after 1–2 min with Azure c600 chemiluminescence imager (Azure Biosystems).

### DNA extraction and *Leishmania* detection by qPCR

The whole organ was collected and macerated in 1 mL of DNA/RNA shield (Zymo). After maceration, DNA was extracted from 100–200 μL following the DNA/RNA Plus kit tissue protocol (Zymo). A total of 40 ng of DNA was used as template for all tissues. Amplification of kinetoplast minicircle DNA of *Leishmania* was achieved using primers JW11 (5’-CCTATT TTACACCAA CCCCCAGT-3’), JW12 (5’-GGGTAGGG GCGTTCTGCGAAA-3’), and TaqMan probe (5’-[Aminoc6+TxRed] RAAARKKVRTRCAGAAAYCCCGT [BHQ2]-3’) as previously described^14^. A standard curve was produced by spiking naive mice tissue with a known number of *L. donovani* parasites from culture. The number of parasites from experimental samples was determined by fitting the cycle threshold (Ct) values in the equation of the line. All samples were run in duplicate or triplicate in CFX96 Touch Real-Time PCR Detection System, Bio-Rad. The parasite burden per tissue was calculated per mL for all tissues with exception of the spleen and liver. For the spleen and the liver, the parasites per mL were calculated and then divided by the mean weight of the spleen or liver of unchallenged (MN or WN) animals (Fig. S3c).

### Sysmex blood-cell analysis

A 100 μL of blood was collected in EDTA treated tubes (BD) and diluted 1:6 in PBS (Lonza). Diluted blood (60 μl) was run in the Sysmex XN 1000 hematology analyzer for the percent of neutrophils, monocytes, and lymphocytes in the blood.

### Serum collection

Blood was collected in serum collection tubes (BD) by cardiac puncture at the experimental endpoint and centrifuged at 10,000 × *g* for 10 min. Serum was separated and aliquots were kept at −20 °C until analysis.

### Chemical metabolic panel

A comprehensive chemical metabolic pathway was performed on the collected serum and analyzed using the Abaxis comprehensive diagnostic profile mouse panel (Cat No, 500-1038). Metabolic panel included the following analytes: total protein, albumin, globulin, glucose, amylase, alkaline phosphatase (AKP), alanine aminotransferase (ALT), and urea nitrogen.

### HO-1 serum ELISA

Mouse serum was diluted using the kit’s diluent buffer at 1:2 ratio and the ELISA was performed following the recommendations of the mouse Heme Oxygenase 1 Simple Step ELISA Kit (Abcam, Cat No. ab204524).

### Cytokine Luminex multiplex

A panel of 11 serum cytokines was analyzed using the ProcartaPlex Mouse Th1/Th2 Cytokine Panel, 11 plex kit (Invitrogen, Cat No. EPX110-20820-901). Briefly, serum was diluted at a 1:1 ratio and incubated with the magnetic beads overnight at 4 °C using an orbital shaker. The next day, the assay was performed following the manufacturer’s instructions and data were acquired in the Luminex 100 analyzer. The limit of quantification of each cytokine reported in pg/ml is as follows: IL-10 (7.67), IL-4 (1.42), IL-5 (2.26), IL-6 (4.88), IL17-A (1.45), IL-18 (4.6), IL-2 (1.81), IL-22 (11), IFN-γ (0.93), IL-12p70 (1.89), TNF-α (3.17).

### Metagenomic 16S sequencing

#### Genomic DNA extraction

Genomic DNA was extracted from fecal samples using the Cetyltrimethylammonium bromide (CTAB) method. DNA concentration and purity was monitored by running a 1% agarose gel and DNA was diluted to 1 ng/µL in molecular graded water.

#### Amplicon generation

16S rRNA/18SrRNA/ITS genes of distinct regions (16SV4/16SV3/16SV3- V4/16SV4-V5, 18SV4/18SV9, ITS1/ITS2, ArcV4) were amplified using specific barcoded primers (e.g. 16SV4:515F- 806R, 18SV4: 528F-706R, 18SV9: 1380F-1510R). All PCR reactions were carried out with 15 μL of Phusion® High-Fidelity PCR Master Mix (New England Biolabs); 0.2 μM of forward and reverse primers, and 10 ng DNA. PCR conditions, initial denaturation at 98 °C for 1 min, followed by 30 cycles of denaturation at 98 °C for 10 s, annealing at 50 °C for 30 s, and elongation at 72 °C for 30 s and 72 °C for 5 min. PCR products were purified.

#### Library preparation and sequencing

Sequencing libraries were generated, and indexes were added. Quality control of the DNA was checked by Qubit (Invitrogen), qPCR for quantification, and bioanalyzer. Quantified libraries were pooled and sequenced on Illumina platforms, according to effective library concentration and data amount required.

#### Bioinformatics analysis pipeline

Following sequencing, trimmed reads were imported into QIIME2 (v. 2021.4) for processing using the import command^56^. Quality control, denoising, and ASV picking were performed using the DADA2 plugin^57^. Trimming parameters were set to 0 as sequences had already been trimmed by the sequencing facility. To create phylogenetic trees, sequences were aligned with mafft^58^ and were then used to construct a phylogeny with fasttree2^59^ using the command align-to-tree-mafft-fasttree. Taxonomic classification was performed using the QIIME2 naive Bayes classifier, pre-trained on the Silva 138-99 database, containing trimmed sequences representing the region between the 341F/806R primers. The final ASV table, tree file, and taxonomy files were imported into R (v. 4.3.2) for downstream processing and analysis.

Files were combined into one R object using the phyloseq package (v 1.46.0)^60^. We performed additional read processing, including removing reads that were not classified past the Kingdom level, removing reads assigned to Eukaryota and Archaea, and removing singletons. Rarefaction curves were also generated to determine whether sufficient read coverage had been achieved, following which samples were rarefied using the lowest sample read count (104,715). Lastly, ASVs with no taxonomic information were grouped together under the next taxonomic level containing information: i.e., if an ASV was classified at the Family level but not at the Genus level, the genus and species level information would be filled in as Family_NameOfASVFamily.

Alpha diversity plots showing both observed richness and Shannon Index values were created using the phyloseq and ggplot2 (v 3.4.4) packages^61^. Distance matrices were generated using phyloseq, and PCoA coordinates were calculated using the ape package (v 5.7-1)^62^. Additional PERMANOVA and PERMDISP tests were performed using the adonis2 and betadisper commands from the vegan package (v 2.6-4)^63^. To create bar plots, the phyloseq object was transformed to a microtable object using the package file2meco (v 0.7), and plots were then generated using the microeco package (v 1.4.0)^64^. The differential abundance analysis was performed using ANCOM-BC (v 2.4.0) and the corresponding raw abundance boxplots were generated using ggplot2^65,66^.

### Statistics and reproducibility

All experiments were conducted blinded, and the blinded code was broken post-data analysis. Statistical analysis was performed using R software. +*p* ≤ 0.9, *p ≤ 0.05, **p ≤ 0.01, ***p ≤ 0.001, and ****p ≤ 0.0001. Refer to Supplementary Data file 2 for the full statistical report.

### Reporting summary

Further information on research design is available in the Nature Portfolio Reporting Summary linked to this article.

## Supplementary information

Supplementary information

Description of Additional Supplementary Files

Supplementary Data 1

Supplementary Data 2

Reporting Summary

## Data Availability

All data are available as Supplementary data files (Supplementary Data file 1). No data were excluded from our study. Statistical analysis and R code report can be access at https://github.com/joedoehl/Malnutrition-exacerbates-pathogenesis-of-sand-fly-transmitted-Leishmania-donovani.git. 16S DNA FASTQ sequencing files have been uploaded to the NCBI BioProject database and can be access at http://www.ncbi.nlm.nih.gov/bioproject/1245452 with accession number PRJNA1245452.

## Code availability

R code report for statistical analysis can be access at https://github.com/joedoehl/Malnutrition-exacerbates-pathogenesis-of-sand-fly-transmitted-Leishmania-donovani.git. Code was deposited in a DOI-minting repository: 10.5281/zenodo.15127944^67^.
