# Supplementary material for: Malnutrition exacerbates pathogenesis of Lutzomyia longipalpis sand fly-transmitted Leishmania donovani
Source: Commun Biol. 2025 May 13;8:746. doi: 10.1038/s42003-025-08106-8 (PMC12075822; doi:10.1038/s42003-025-08106-8)
Supplement: Supplementary file 1 — Supplementary information [file 42003_2025_8106_MOESM1_ESM.pdf]

Supplementary information

Malnutrition exacerbates pathogenesis of *Lutzomyia longipalpis* sand fly-transmitted *Leishmania donovani*

Eva Iniguez[1*], Johannes Doehl[1#], Pedro Cecilio[2#], Tiago Donatelli Serafim[1], Caroline Percopo[3], Yvonne Rangel-Gonzalez[1], Somaditya Dey[1,4], Elvia J. Osorio[5], Patrick Huffcutt[1], Sofia Roitman[6], Claudio Meneses[1], Mara Short[1], Jesus G. Valenzuela[1], Peter C. Melby[5], Shaden Kamhawi[1*]

#These authors contributed equally

*These authors jointly supervised this work; iniguezea@nih.gov, skamhawi@nih.gov.

[1]Vector Molecular Biology Section, Laboratory of Malaria and Vector Research, National Institute of Allergy and Infectious Diseases, National Institutes of Health, Rockville, MD, USA

[2]Vector Biology Section, Laboratory of Malaria and Vector Research, National Institute of Allergy and Infectious Diseases, National Institutes of Health, Rockville, MD, USA

[3]Laboratory of Malaria and Vector Research, National Institute of Allergy and Infectious Diseases, National Institutes of Health, Rockville, MD, USA

[4]Post Graduate Department of Zoology, Barasat Government College, Barasat, West Bengal, India

[5]Department of Internal Medicine, University of Texas Health Science Center at San Antonio, San Antonio, TX, USA

[6]Integrated Data Sciences Section, Research Technologies Branch, National Institute of Allergy and Infectious Diseases, National Institutes of Health, Bethesda, MD, USA 20892

Supplemental figures

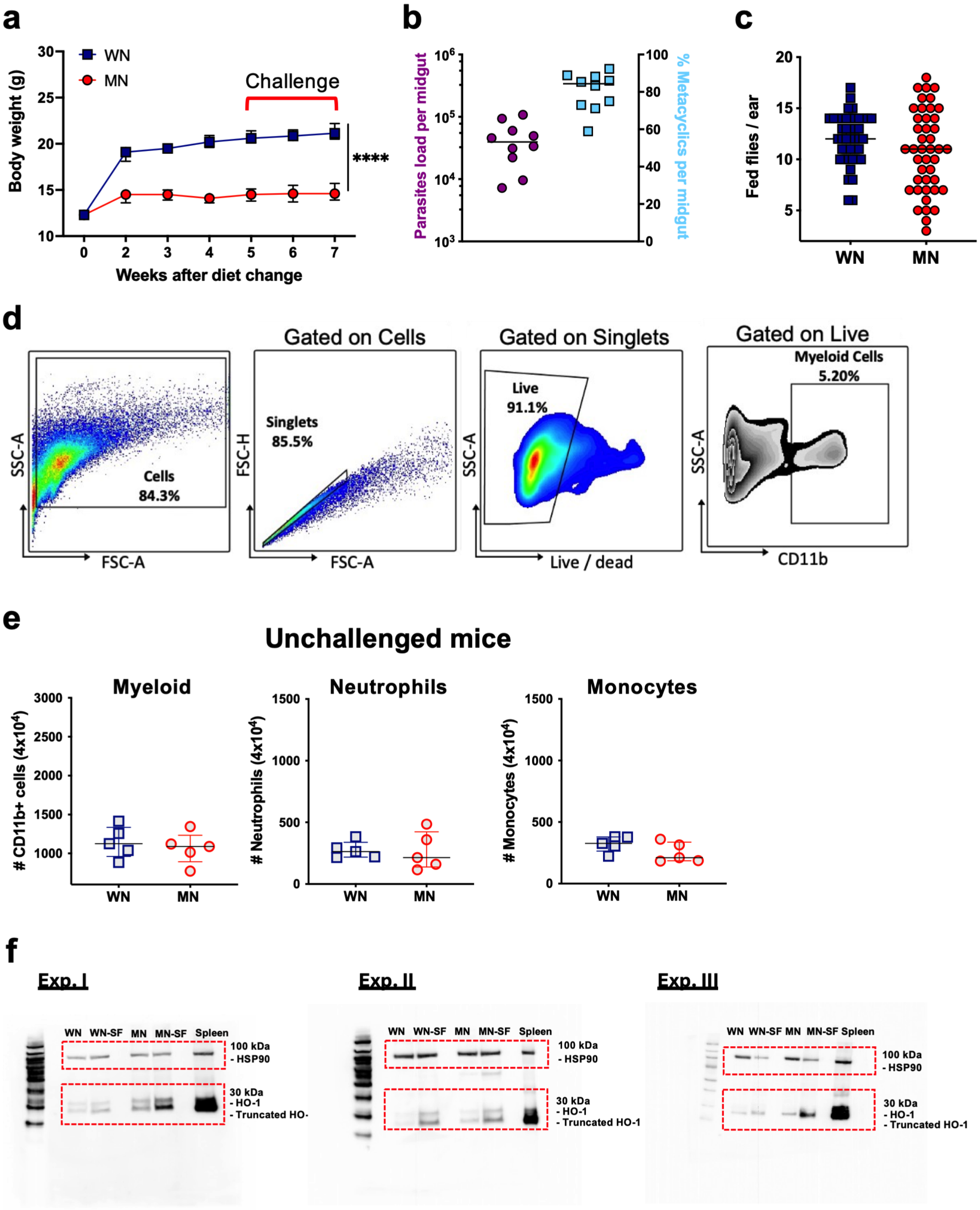

Figure S1. Animal body weight, sand fly infection status and feeding behavior, flow cytometry gating strategy and counts of immune cells in steady state skin

a) Animal body weight post-diet change. BALB/c female mice were assigned to a polynutrient (well-nourished, WN) or polynutrient-deficient (malnourished, MN) diet for up to 7 weeks before challenge. **b)** The status of a representative sand fly infection used in vector-transmission experiments. The number of *Leishmania donovani* parasites per midgut and percent of infectious metacyclic promastigotes was evaluated at day 9-11 post-sand fly infection by artificial membrane feeding. **c)** Feeding score post-transmission. WN and MN mice were exposed to 20 infected sand flies/per ear. **d)** Gating strategy to identify CD11b$^+$ cells, neutrophils (Ly6C$^+$Ly6G$^+$), and inflammatory monocytes (Ly6C$^+$Ly6G$^-$) in skin using flow cytometry. **e)** Number of CD11b$^+$ cells, neutrophils, and inflammatory monocytes in unchallenged WN and MN animals. WN, well-nourished; MN, Malnourished. Cumulative data of 2 experiments, n = 4-5 animals per group **(a)**. Representative data of n = 5 experiments, n = 10 midguts **(b and c)**. Data are representative of 2 experiments, n=5 animals per group, and each data point represents a pool of 2 ears per animal **(e). f)** Uncropped Heme oxygenase 1 (HO-1) expression in skin tissue lysate 72 h post-infected bites by Western blot. HSP90, loading control. WN, well-nourished; MN, Malnourished; SF, sand fly; ND, needle. Western blots from 3 independent experiments, n = 5-10 pooled ears per. Data shows the median with 95% CI **(a)**, median **(b and c)**, and median with IQR **(e)**. ****$P \leq 0.0001$ was considered significant. Significance was calculated by Robust one-way ANOVA **(a),** negative binomial regression model **(c)**, and Linear Contrast Expression **(e)**. Refer to Supplementary Data 2 file for the full statistical analysis report. All experiments were blinded.

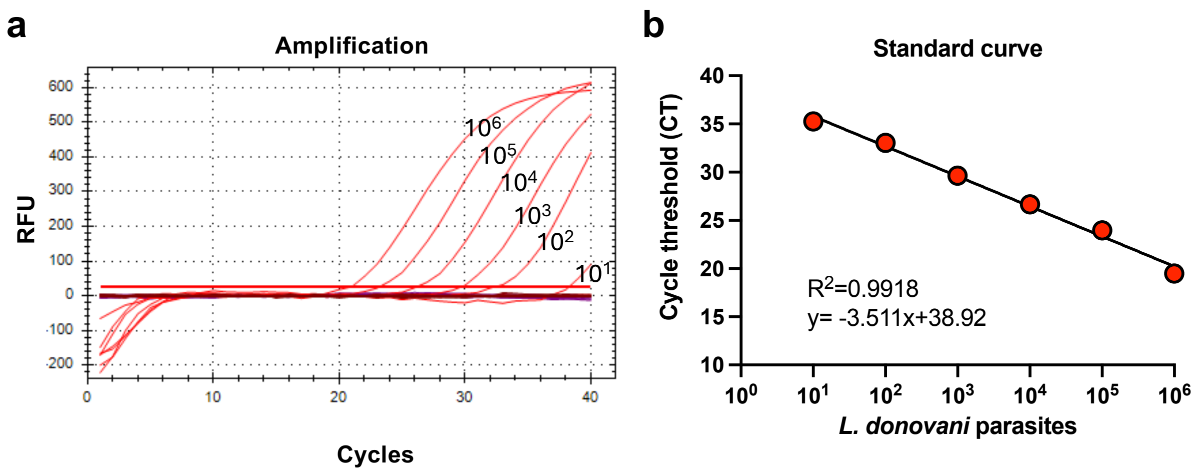

Figure S2. *Leishmania donovani* amplification by qPCR and standard curve

a) Amplification of *L. donovani* parasites from DNA co-extracted after spiking mouse tissues with known numbers of parasites. **b)** Data was used to generate a standard curve of the cycle threshold (CT) that corresponds to the number of spiked *L. donovani* parasites.

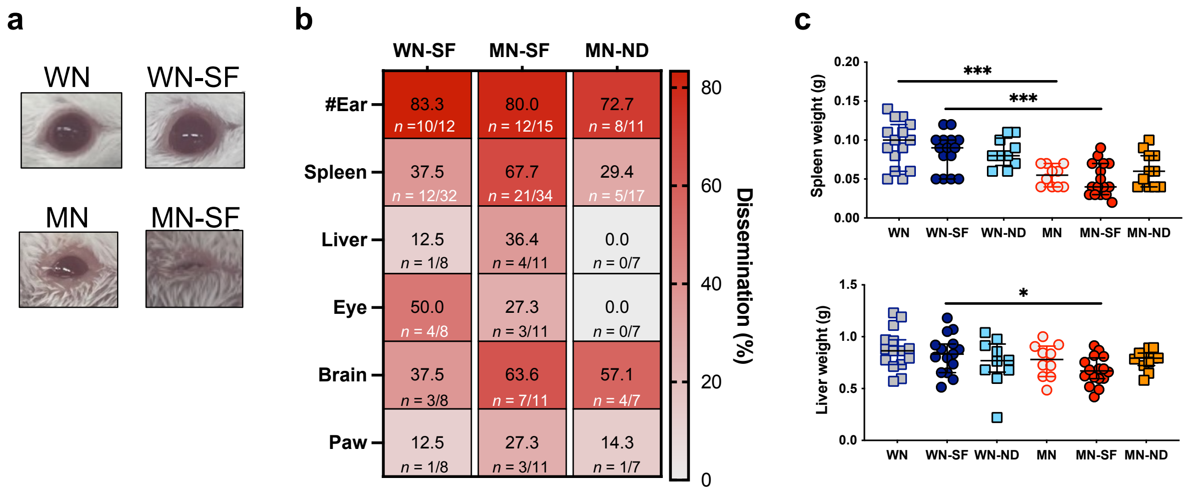

Figure S3. Ocular symptoms and frequency of parasite dissemination in sand fly-infected malnourished animals in the chronic stage of VL

a) Representative images showing the ocular symptoms exhibited in MN-SF animals compared to MN, WN, and WN-SF control groups. **b)** Frequency of parasite dissemination by tissue analyzed by qPCR. **c)** Spleen and liver weight before and after challenge. WN, well-nourished; MN, Malnourished; SF, sand fly; ND, needle. Cumulative data of 2-5 experiments, *n* = 4-5 animals per group **(b).** Cumulative data of 2-4 experiments, *n* = 4-5 animals per group **(c)**. Data shows the median with IQR **(c).** $P \leq 0.05$ was considered significant. *$P \leq 0.05$, and ***$P \leq 0.001$. Significance was calculated by estimated marginal means analysis **(c)**. Refer to Supplementary Data 2 file for the full statistical analysis report. All experiments were blinded.

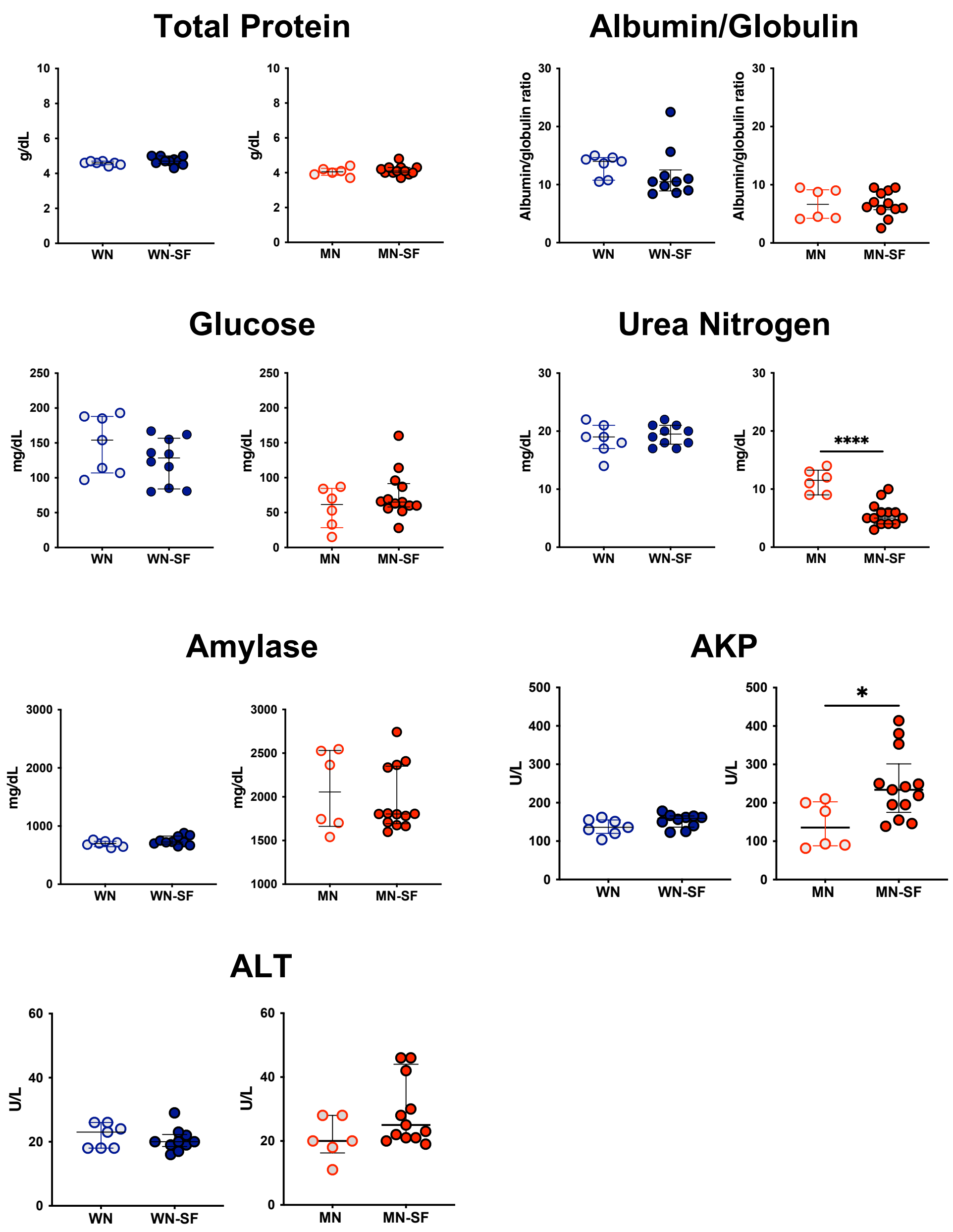

Figure S4. Analysis of circulating levels of metabolic and inflammatory mediators in sand fly-infected malnourished animals in the chronic stage of VL

WN and MN animals were followed up to 30 weeks post-*L. donovani* infected sand fly bites. Serum chemistry panel analytes. WN, well-nourished; MN, Malnourished; SF, sand fly. Cumulative data of 2 experiments, $n \geq 3$ animals per group. Data shows the median with IQR; $P \leq 0.05$ was considered significant. *$P \leq 0.05$, ****$P \leq 0.0001$. Significance was calculated by estimated marginal means for total

protein, albumin, globulin, glucose, and urea nitrogen, or linear contrast expression for ALT, ALP and amylase**.** Refer to Supplementary Data 2 file for the full statistical analysis report. All experiments were blinded.

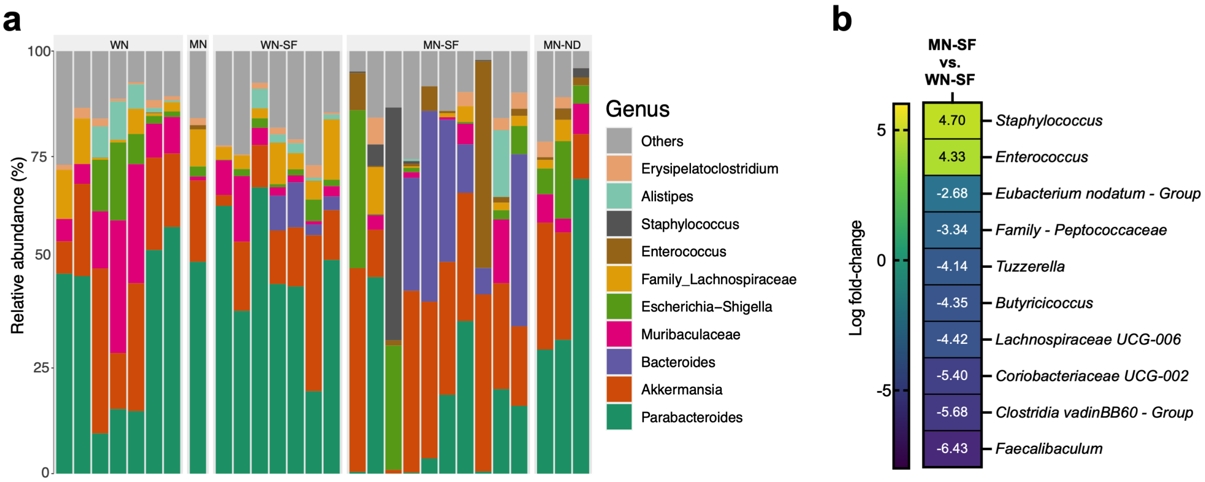

Figure S5. Relative microbial abundance of top genera in sand fly-infected malnourished animals in the chronic stage of VL

a-b) Microbes in fecal matter from animals at baseline for WN and MN, WN-SF and MN-SF, or MN-ND animals were compared. **a)** Relative abundance of the top ten genera per animal. **b)** Log fold change of microbial abundance in MN-SF versus WN-SF animals. Graph shows the top 9 statistically significant bacteria. WN, well-nourished; MN, Malnourished; SF, sand fly; ND, needle. Cumulative data of 2 experiments, $n \geq 3$ samples per group, except for MN baseline where only one sample was analyzed. Differential abundance and significance were calculated using the ANCOM-BC method (FDR adjusted P value ≤ 0.05) **(b)**. All experiments were blinded.
