# Supplementary material for: Malnutrition exacerbates pathogenesis of Lutzomyia longipalpis sand fly-transmitted Leishmania donovani
Source: Commun Biol. 2025 May 13;8:746. doi: 10.1038/s42003-025-08106-8 (PMC12075822; doi:10.1038/s42003-025-08106-8)
Supplement: Supplementary file 2 — Description of Additional Supplementary Files [file 42003_2025_8106_MOESM2_ESM.docx]

Description of Additional Supplementary Files

**File name:** Supplementary Data 1

**Description:** The source data behind all the graphs in the paper.

**File name:** Supplementary Data 2

**Description:** Statistics report.
