# Supplementary material for: Malnutrition exacerbates pathogenesis of Lutzomyia longipalpis sand fly-transmitted Leishmania donovani
Source: Commun Biol. 2025 May 13;8:746. doi: 10.1038/s42003-025-08106-8 (PMC12075822; doi:10.1038/s42003-025-08106-8)
Supplement: Supplementary file 4 — Supplementary Data 2 [file 42003_2025_8106_MOESM4_ESM.pdf]

Statistical report: Malnutrition exacerbates pathogenesis of sand fly-transmitted *Leishmania donovani*

Johannes S. P. Doehl

2024-05-22

Summary

This appendix contains the detailed statistical analyses of all the data presented in the main and supplementary figures of the publication titled: "Malnutrition exacerbates pathogenesis of sand fly-transmitted *Leishmania donovani*". This report was an effort for transparency regarding the applied statistical analyses, in which we also considered retrospective power and sample size calculation to obtain a sense of statistical power in our data to better understand how well our statistical analyses reflected observed biological differences. Insufficient statistical power can result in type II errors, the erroneous acceptance of the null hypothesis that there is no meaning full difference between groups. Even though, we frequently observed lack of statistical power in our data, larger sample sizes were prohibitive due to cost and ethical consideration. That did not detract from the quality of findings presented in the manuscript. On the contrary, observed biological differences were often supported by statistical evaluation and supported the two main observations that infection by sand fly and the nutritional state of the individual had major impacts on the development of leishmaniasis and the infecting parasite, *Leishmania donovani*.

Main Body

General comments

The report is arranged in the order of the figures and their panels as they appear in the main manuscript, to make it easier to accompany. Most data outputs are summarized in tables for easy accessibility. We clearly state the selection process of what we identified as most appropriate comparative analysis for the data for each figure panel. In some cases, data from multiple figure panels were analyzed together as it was one dataset but was split for comprehensive presentation of the data. Also, presented data in the manuscripts figures usually present untransformed datasets, while for the statistical analyses, data transformation was applied as indicated in places. All statistical analyses were performed naive to any expected outcome for unbiased, objective application and interpretation.

Software and packages

All the statistics presented in the manuscript "Malnutrition exacerbates pathogenesis of sand fly-transmitted *Leishmania donovani*" and in this statistical report were produced in RStudio version 2024.4.2.764 [32]. We used R version 4.4.1 [28] and the following R packages: aod v. 1.3.3 [20], betareg v. 3.2.0 [6, 11, 17], bookdown v. 0.40 [37, 38], car v. 3.1.2 [8], caret v. 6.0.94 [18], effectsize v. 0.8.9 [4], emmeans v. 1.10.3 [19], epitools v. 0.5.10.1 [3], ggpubr v. 0.6.0 [14], grid v. 4.4.1 [29], Hmisc v. 5.1.3 [12], janitor v. 2.2.0 [7], knitr v. 1.48 [40, 39, 41], lmtest v. 0.9.40 [44], MASS v. 7.3.61 [34], metan v. 1.18.0 [25], moments v. 0.14.1 [16], multcomp v. 1.4.25 [13], nlme v. 3.1.165 [27, 26], pastecs v. 1.4.2 [10], performance v. 0.12.0 [22], pROC v. 1.18.5 [30], pwrss v. 0.3.1 [5], rcompanion v. 2.4.36 [24], rmarkdown v. 2.27 [42, 43, 2], rstatix v. 0.7.2 [15], sjmisc v. 2.8.10 [21], stringi v. 1.8.4 [9], tidyverse v. 2.0.0 [36], tiff v. 0.1.12 [33], WRS2 v. 1.1.6 [23].[1] For the creation of this statistical report, the author made use of Rmarkdown [1]. The original codes for this statistical report are available as Rmarkdown files through the author's github portal[2].

Main Figures

Figure 1

Panel b and c

Data analysis

Figure 1 b and c present the cell counts of total Myeloid_cells and separately, of Neutrophils and Monocytes from N=60, 60, 60 pools of single cell suspensions, respectively, prepared from BALB/c mouse ears collected 24 h and 72 h post infection with *Leishmania donovani* by "needle" inoculation or infective sand flies (SF) bites. Please, refer to the methods section of the publication for more details on sample preparation. Note that different mice were sampled at 24 h and 72 h post infection, which meant that this dataset satisfied the independence of data points and therefore, did not represent a repeatedly measured dataset. Thus, this dataset contained three between-subject factors, "Diet" (well-nourished [WN] or malnourished [MN]), "Route" (uninfected control [Ctrl], needle inoculation [Needle], infective sand fly bites [SF]) and "Time_point" (collection at 24 h or 72 h). Based on this information, a three-way analysis was indicated.

Thus, we assessed the data for compliance with assumptions for a three-way ANOVA:

- Data normality
- Homogeneity of variance
- No significant outliers

Initial assumption assessment indicated that data transformation was required to meet assumptions for a

[1]R package citations were managed using the 'grateful' package [31], while inter-package function name conflicts were managed with the 'conflicted' package [35]

[2]https://github.com/joedoehl/Malnutrition-exacerbates-pathogenesis-of-sand-fly-transmitted-Leishmania-donovani.git

three-way ANOVA. Thus, we settled for a Box-Cox power transformation of all datasets presented in this figure. Thus, data distribution and variance appear different in the main figure panels in the publication from the once that were used in the analysis post transformation.

Assumption analyses

Data normality

The assessment of the transformed data distribution for each group was conducted by Shpiro-Wilks test and QQ-plot for counts of Myeloid_cells, Neutrophils and Monocytes separately. Note that all groups of all datasets consisted of N=5 pools of mouse ear single-cell suspensions, which made it difficult to assess data distribution reliably by Shapiro-Wilks test.

Myeloid_cells

In spite of assessment limitations due to small group sizes, we concluded based on the Shapiro-Wilks test (Appendix table 1) and QQ-plots (Fig.1b-c-1) that all groups of the dataset were likely to follow a normal distribution.

Appendix Table 1

Myeloid cells: Univariate Shapiro-Wilks test results

Diet	Route	Time_point	variable	statistic	p	Outcome
WN	Ctrl	24_h	Counts	0.9919	0.9860	ns
WN	Ctrl	72_h	Counts	0.9919	0.9860	ns
MN	Ctrl	24_h	Counts	0.9378	0.6502	ns
MN	Ctrl	72_h	Counts	0.9378	0.6502	ns
WN	Needle	24_h	Counts	0.8543	0.2084	ns
WN	Needle	72_h	Counts	0.9022	0.4219	ns
MN	Needle	24_h	Counts	0.8424	0.1716	ns
MN	Needle	72_h	Counts	0.9791	0.9298	ns
WN	SF	24_h	Counts	0.7891	0.0659	ns
WN	SF	72_h	Counts	0.7877	0.0641	ns
MN	SF	24_h	Counts	0.9207	0.5344	ns
MN	SF	72_h	Counts	0.9947	0.9933	ns

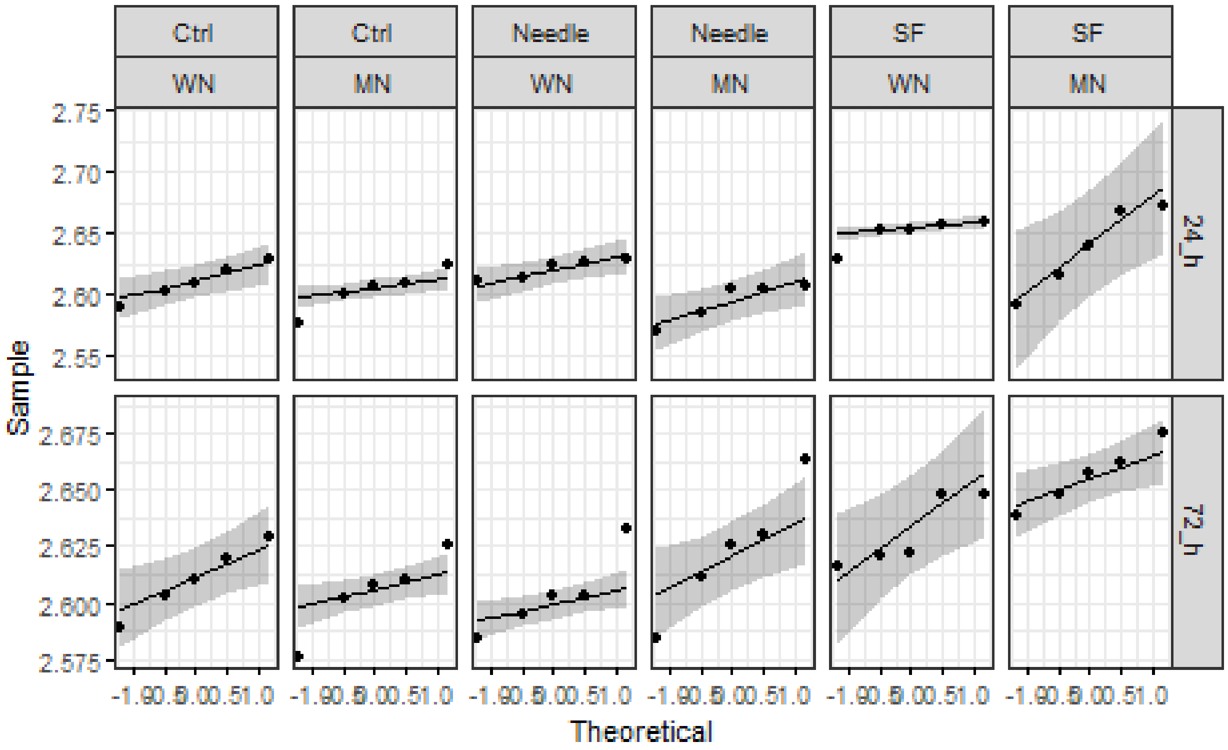

Fig.1b-c-1: QQ-plots of myeloid cell counts split into groups by predictor variables

Neutrophils

In spite of assessment limitations due to small group sizes, we concluded based on the Shapiro-Wilks test (Appendix table 2) and QQ-plots (Fig.1b-c-2) that all groups of the dataset were likely to follow a normal distribution.

Appendix Table 2

Neutrophils: Univariate Shapiro-Wilks test results

Diet	Route	Time_point	variable	statistic	p	Outcome
WN	Ctrl	24_h	Counts	0.9133	0.4879	ns
WN	Ctrl	72_h	Counts	0.9133	0.4879	ns
MN	Ctrl	24_h	Counts	0.9609	0.8144	ns
MN	Ctrl	72_h	Counts	0.9609	0.8144	ns
WN	Needle	24_h	Counts	0.8948	0.3818	ns
WN	Needle	72_h	Counts	0.9849	0.9590	ns
MN	Needle	24_h	Counts	0.8150	0.1068	ns
MN	Needle	72_h	Counts	0.9745	0.9032	ns
WN	SF	24_h	Counts	0.7958	0.0748	ns
WN	SF	72_h	Counts	0.9210	0.5361	ns
MN	SF	24_h	Counts	0.8692	0.2632	ns

MN	SF	72_h	Counts	0.9840	0.9547	ns

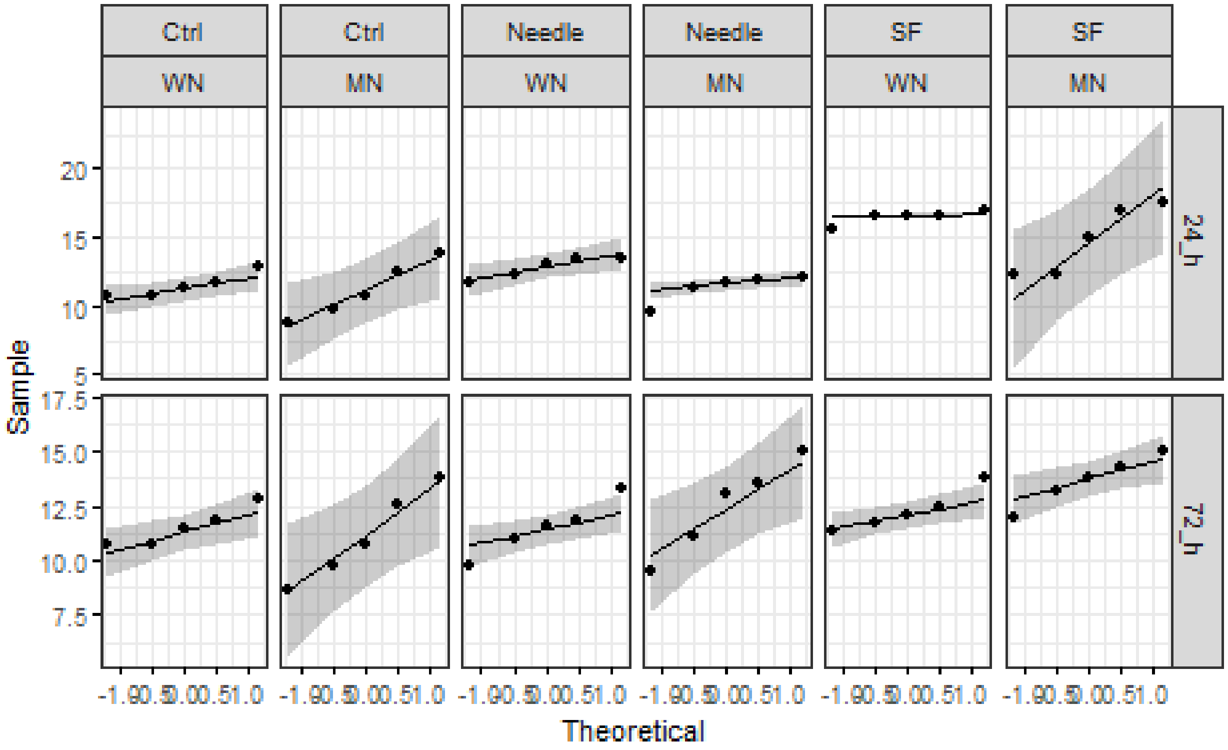

Fig.1b-c-2: QQ-plots of neutrophil counts split into groups by predictor variables

Monocytes

In spite of assessment limitations due to small group sizes, we concluded based on the Shapiro-Wilks test (Appendix table 3) and QQ-plots (Fig.1b-c-3) that all groups of the dataset were likely to follow a normal distribution.

Appendix Table 3

Monocytes: Univariate Shapiro-Wilks test results

Diet	Route	Time_point	variable	statistic	p	Outcome
WN	Ctrl	24_h	Counts	0.8597	0.2271	ns
WN	Ctrl	72_h	Counts	0.8597	0.2271	ns
MN	Ctrl	24_h	Counts	0.8517	0.1999	ns
MN	Ctrl	72_h	Counts	0.8517	0.1999	ns
WN	Needle	24_h	Counts	0.8945	0.3803	ns
WN	Needle	72_h	Counts	0.9625	0.8250	ns
MN	Needle	24_h	Counts	0.9079	0.4552	ns
MN	Needle	72_h	Counts	0.9435	0.6907	ns
WN	SF	24_h	Counts	0.9944	0.9927	ns

WN	SF	72_h	Counts	0.8455	0.1806	ns
MN	SF	24_h	Counts	0.9226	0.5466	ns
MN	SF	72_h	Counts	0.9595	0.8048	ns

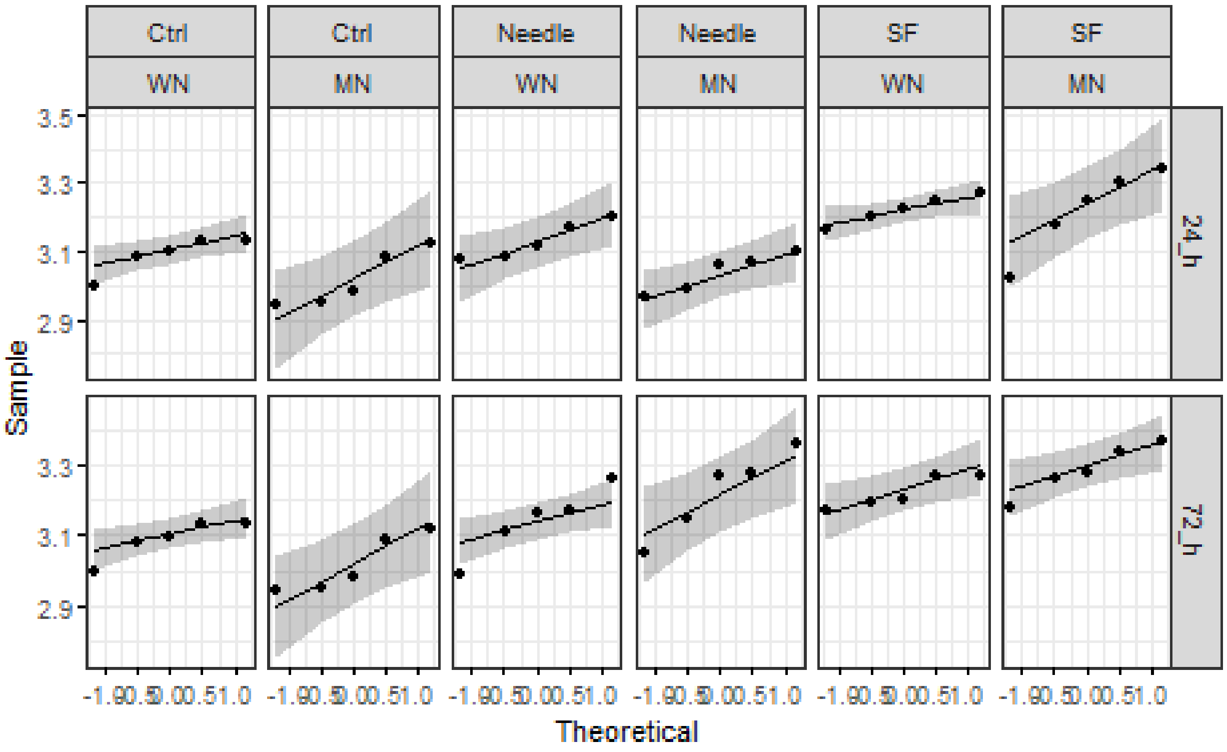

Fig.1b-c-3: QQ-plots of monocyte counts split into groups by predictor variables

Homogeneity of variance The assessment of homogeneity of variance was also conducted for myeloid cell, neutrophil and monocyte counts separately. We employed the Levene's test on each dataset. The p-value for the test suggested that the assumption of homogeneity of variance held for Myeloid_cells (p=0.5236), held for Neutrophils (p=0.0502), and held for Monocytes (p=0.7728).

Outliers It can be difficult to determine outliers in small datasets reliably as the analysis is dependent on the interquartile range of the data per group. We attempted it anyway and found a potential 8 outliers in the Myeloid_cells dataset (Appendix table 4), 5 outliers in the Neutrophils dataset (Appendix table 5), and 3 outliers in the Monocytes dataset (Appendix table 6), of which some were classified as extreme. However, larger datasets may have found these not be outliers, but just peripheral.

Appendix Table 4

Myeloid cells: List of possible outliers

Diet	Route	Time_point	is.outlier	is.extreme
MN	Ctrl	24_h	TRUE	FALSE

MN	Ctrl	24_h	TRUE	FALSE
MN	Ctrl	72_h	TRUE	FALSE
MN	Ctrl	72_h	TRUE	FALSE
WN	Needle	72_h	TRUE	TRUE
MN	Needle	72_h	TRUE	FALSE
MN	Needle	72_h	TRUE	FALSE
WN	SF	24_h	TRUE	TRUE

Appendix Table 5

Neutrophils: List of possible outliers

Diet	Route	Time_point	is.outlier	is.extreme
WN	Needle	72_h	TRUE	FALSE
MN	Needle	24_h	TRUE	TRUE
WN	SF	24_h	TRUE	TRUE
WN	SF	24_h	TRUE	FALSE
WN	SF	72_h	TRUE	FALSE

Appendix Table 6

Monocytes: List of possible outliers

Diet	Route	Time_point	is.outlier	is.extreme
WN	Ctrl	24_h	TRUE	FALSE
WN	Ctrl	72_h	TRUE	FALSE
WN	Needle	72_h	TRUE	FALSE

Three-way analysis

The appropriate three-way analysis was performed for Myeloid_cells, Neutrophils and Monocytes separately.

Myeloid_cells

Based on the assumption tests, we decided to apply a Robust three-way ANOVA to the Myeloid_cells dataset to determine the effects of "Diet", infection "Route" and time post infection ("Time_point") on the Myeloid_cells counts per pooled ear single-cell suspension (Appendix table 7). The test output showed that only the infection "Route" was a statistically significant predictor, while the interaction between Time_point and Diet was statistically significant, too.

Appendix Table 7

Myeloid cells: Robust three-way ANOVA

Predictors	value	p.value	sig.
Diet	0.0048	0.9500	ns
Route	29.5689	0.0007	***
Time_point	0.0447	0.8390	ns
Diet:Route	0.7681	0.7120	ns
Diet:Time_point	7.4482	0.0230	*
Route:Time_point	0.2226	0.9040	ns
Diet:Route:Time_point	7.4542	0.0720	+

We looked for main effects by splitting the data by each predictor separately and analyzed the respective remaining two predictor by a Robust two-way ANOVA. The results showed that, after adjustment of p-values for multiple tests, only the "Route" predictor produced statistically significant p-values, regardless of whether the data was split by "Diet" or "Time-point" (Appendix table 8). This suggested that the only predictor of impact on Myeloid_cells counts was the route of infection.

Appendix Table 8

Myeloid cells: Robust two-way ANOVA

Grouper	Predictor	value	p.value	Sig.	p.value.adj	sig.
Grouped by Route						
Ctrl	Diet	0.7025	0.415	ns	0.5810	ns
Ctrl	Time_point	0.0000	0.999	ns	0.9990	ns
Ctrl	Diet:Time_point	0.0000	0.999	ns	0.9990	ns
Needle	Diet	0.1500	0.707	ns	0.8248	ns
Needle	Time_point	0.4370	0.523	ns	0.6510	ns
Needle	Diet:Time_point	6.7428	0.024	*	0.1008	ns
SF	Diet	0.4354	0.527	ns	0.6510	ns
SF	Time_point	0.0015	0.971	ns	0.9990	ns
SF	Diet:Time_point	4.1861	0.069	+	0.1701	ns
Grouped by Diet						
WN	Route	28.0464	0.001	***	0.0105	*
WN	Time_point	5.2407	0.032	*	0.1120	ns
WN	Route:Time_point	2.5384	0.326	ns	0.5444	ns
MN	Route	19.2056	0.003	**	0.0210	*
MN	Time_point	3.5769	0.076	+	0.1701	ns
MN	Route:Time_point	2.5075	0.337	ns	0.5444	ns

Grouped by Time_point

24_h	Diet	4.6264	0.050	*	0.1500	ns
24_h	Route	17.3213	0.004	**	0.0210	*
24_h	Diet:Route	2.3091	0.368	ns	0.5520	ns
72_h	Diet	3.3991	0.081	+	0.1701	ns
72_h	Route	29.3554	0.001	***	0.0105	*
72_h	Diet:Route	5.2718	0.112	ns	0.2138	ns

For the analysis of the simple simple main effect for each respective predictor, we performed Robust one-way ANOVA with individual predictors of the data split by the other two predictors. The output showed yet again that after the adjustment of p-values for multiple tests, only the "Route" predictor produce statistically significant p-values (Appendix table 9). Here, the differences were observed in the well-nourished group (WN) at 24 h post infection and for the malnourished group at 72 h post infection.

Appendix Table 9

Myeloid cells: Robust one-way ANOVA

Predictor_1	Predictor_2	Effect	test	df1	df2	p.value	effsize	p.value.adj	sig.
Predictor: Time_point									
Ctrl	WN	Time_point	0.0000	1	8.0000	1.0000	0.0000	1.0000	ns
Ctrl	MN	Time_point	0.0000	1	8.0000	1.0000	0.0000	1.0000	ns
Needle	WN	Time_point	3.5282	1	5.5647	0.1132	0.7337	0.2033	ns
Needle	MN	Time_point	3.6121	1	6.2598	0.1041	0.9017	0.2033	ns
SF	WN	Time_point	4.8246	1	7.5253	0.0614	0.7261	0.1965	ns
SF	MN	Time_point	1.3003	1	5.2694	0.3033	0.5259	0.4412	ns
Predictor: Diet									
24_h	Ctrl	Diet	0.3512	1	7.8195	0.5702	0.2800	0.6516	ns
24_h	Needle	Diet	10.1859	1	5.9402	0.0191	0.9926	0.1017	ns
24_h	SF	Diet	0.6400	1	4.9962	0.4600	0.3842	0.6134	ns
72_h	Ctrl	Diet	0.3512	1	7.8195	0.5702	0.2800	0.6516	ns
72_h	Needle	Diet	1.5616	1	6.7245	0.2532	0.5753	0.4051	ns
72_h	SF	Diet	7.3397	1	7.8679	0.0271	0.8992	0.1084	ns
Predictor: Route									
24_h	WN	Route	12.5661	2	7.4933	0.0040	0.9018	0.0323	*
24_h	MN	Route	2.9293	2	7.5513	0.1144	0.6507	0.2033	ns
72_h	WN	Route	3.3900	2	7.9569	0.0861	0.6194	0.2033	ns
72_h	MN	Route	12.7031	2	7.4981	0.0039	0.7858	0.0323	*

For the pairwise comparison, we applied a Linear contrast expression. The output showed that at 24 h post

infection, the sand fly infection route was statistically significantly different from the control and needle inoculum in the well-nourished (WN) group (Appendix table 10). At 72 h, the sand fly infection route was statistically significantly different from the control in the malnourished group. Further, statistically significant difference were observed between well-nourished and malnourished mice inoculated by needle at 24 h post infection and infected by sand fly at 72 h post infection.

Appendix Table 10

Myeloid cells: Pairwise comparison by Linear Contrast Expression

Predictor_1	Predictor_2	Group	Group.1	psihat	ci.lower	ci.upper	p.value	Sig.
Predictor: Time_point								
Ctrl	WN	24_h	72_h	0.0000	-0.0224	0.0224	1.0000	ns
Ctrl	MN	24_h	72_h	0.0000	-0.0261	0.0261	1.0000	ns
Needle	WN	24_h	72_h	0.0169	-0.0055	0.0394	0.1132	ns
Needle	MN	24_h	72_h	-0.0285	-0.0647	0.0078	0.1041	ns
SF	WN	24_h	72_h	0.0197	-0.0012	0.0406	0.0614	+
SF	MN	24_h	72_h	-0.0190	-0.0611	0.0231	0.3033	ns
Predictor: Diet								
24_h	Ctrl	WN	MN	0.0062	-0.0182	0.0306	0.5702	ns
24_h	Needle	WN	MN	0.0261	0.0060	0.0461	0.0191	*
24_h	SF	WN	MN	0.0131	-0.0290	0.0552	0.4600	ns
72_h	Ctrl	WN	MN	0.0062	-0.0182	0.0306	0.5702	ns
72_h	Needle	WN	MN	-0.0193	-0.0561	0.0175	0.2532	ns
72_h	SF	WN	MN	-0.0256	-0.0474	-0.0037	0.0271	*
Predictor: Route								
24_h	WN	Ctrl	Needle	-0.0103	-0.0350	0.0144	0.2350	ns
24_h	WN	Ctrl	SF	-0.0403	-0.0666	-0.0140	0.0040	**
24_h	WN	Needle	SF	-0.0300	-0.0502	-0.0098	0.0040	**
24_h	MN	Ctrl	Needle	0.0096	-0.0225	0.0416	0.4028	ns
24_h	MN	Ctrl	SF	-0.0334	-0.0888	0.0220	0.1542	ns
24_h	MN	Needle	SF	-0.0430	-0.0983	0.0123	0.1421	ns
72_h	WN	Ctrl	Needle	0.0066	-0.0253	0.0385	0.5523	ns
72_h	WN	Ctrl	SF	-0.0206	-0.0498	0.0087	0.1060	ns
72_h	WN	Needle	SF	-0.0272	-0.0595	0.0050	0.1060	ns
72_h	MN	Ctrl	Needle	-0.0189	-0.0665	0.0287	0.2597	ns
72_h	MN	Ctrl	SF	-0.0524	-0.0828	-0.0219	0.0031	**
72_h	MN	Needle	SF	-0.0335	-0.0804	0.0135	0.0936	+

Neutrophils

Based on the assumption tests, we decided to apply a Robust three-way ANOVA to the Neutrophils dataset to determine the effects of “Diet”, infection “Route” and time post infection (“Time_point”) on the Neutrophils count per pooled ear single-cell suspension (Appendix table 11). As for Myeloid_cells, the test output showed that only the infection “Route” was a statistically significant predictor, but none of the interaction terms were.

Appendix Table 11

Neutrophils: Robust three-way ANOVA

Predictors	value	p.value	sig.
Diet	0.1394	0.720	ns
Route	23.0377	0.004	**
Time_point	4.3763	0.065	+
Diet:Route	0.0479	0.979	ns
Diet:Time_point	4.0582	0.074	+
Route:Time_point	5.4366	0.150	ns
Diet:Route:Time_point	1.9427	0.458	ns

We looked for main effects by splitting the data by each predictor separately and analyzed the respective remaining two predictor by a Robust two-way ANOVA. After adjustment of p-values for multiple tests, we observed statistically significant differences between 24 h and 72 h post infection, when the data was split by infestation route, in the well-nourished group for Route, Time_point and their interaction, as much as for infection route in the malnourished group, when the data was split by “Diet”; and for infection routes at 24 h post infection, when the data was split by Time_point (Appendix table 12).

Appendix Table 12

Neutrophils: Robust two-way ANOVA

Grouper	Predictor	value	p.value	Sig.	p.value.adj	sig.
Grouped by Route						
Ctrl	Diet	0.3205	0.583	ns	0.8745	ns
Ctrl	Time_point	0.0000	0.999	ns	0.9990	ns
Ctrl	Diet:Time_point	0.0000	0.999	ns	0.9990	ns
Needle	Diet	0.1839	0.678	ns	0.8899	ns
Needle	Time_point	0.0428	0.841	ns	0.9812	ns
Needle	Diet:Time_point	3.7795	0.078	+	0.1638	ns
SF	Diet	0.0473	0.834	ns	0.9812	ns
SF	Time_point	16.1728	0.003	**	0.0126	*
SF	Diet:Time_point	5.0287	0.053	+	0.1237	ns

Grouped by Diet						
WN	Route	68.7607	0.001	***	0.0053	**
WN	Time_point	31.4785	0.001	***	0.0053	**
WN	Route:Time_point	34.6536	0.001	***	0.0053	**
MN	Route	13.4578	0.009	**	0.0315	*
MN	Time_point	0.0021	0.964	ns	0.9990	ns
MN	Route:Time_point	1.9351	0.423	ns	0.6833	ns
Grouped by Time_point						
24_h	Diet	4.6992	0.050	*	0.1237	ns
24_h	Route	37.4309	0.001	***	0.0053	**
24_h	Diet:Route	0.9951	0.644	ns	0.8899	ns
72_h	Diet	1.3219	0.267	ns	0.5097	ns
72_h	Route	8.1667	0.046	*	0.1237	ns
72_h	Diet:Route	2.1155	0.395	ns	0.6833	ns

For the analysis of the simple simple main effect for each respective predictor, we performed Robust one-way ANOVA with individual predictors of the data split by the other two predictors. The output showed statistically significant p-values for the sand fly inoculation in the well-nourished group for the Time_point predictor and at 24 h post infection in the well-nourished group for the infection route (Appendix table 13).

Appendix Table 13

Neutrophils: Robust one-way ANOVA

Predictor_1	Predictor_2	Effect	test	df1	df2	p.value	effsize	p.value.adj	sig.
Predictor: Time_point									
Ctrl	WN	Time_point	0.0000	1	8.0000	1.0000	0.0000	1.0000	ns
Ctrl	MN	Time_point	0.0000	1	8.0000	1.0000	0.0000	1.0000	ns
Needle	WN	Time_point	4.1593	1	6.4828	0.0840	0.8107	0.2240	ns
Needle	MN	Time_point	1.0452	1	5.6120	0.3486	0.4543	0.5706	ns
SF	WN	Time_point	76.4341	1	5.6989	0.0002	1.0898	0.0013	**
SF	MN	Time_point	0.9078	1	5.5609	0.3803	0.3810	0.5706	ns
Predictor: Diet									
24_h	Ctrl	Diet	0.1603	1	5.3717	0.7043	0.1989	0.8050	ns
24_h	Needle	Diet	7.1359	1	7.4476	0.0302	1.0777	0.1609	ns
24_h	SF	Diet	2.0228	1	4.2581	0.2239	0.5190	0.4478	ns
72_h	Ctrl	Diet	0.1603	1	5.3717	0.7043	0.1989	0.8050	ns
72_h	Needle	Diet	0.7151	1	6.4714	0.4279	0.3795	0.5706	ns

72_h	SF	Diet	4.0661	1	7.7816	0.0795	0.7530	0.2240	ns
Predictor: Route									
24_h	WN	Route	78.7826	2	7.3614	<0.0001	0.9442	0.0002	***
24_h	MN	Route	3.9055	2	6.8243	0.0740	0.7267	0.2240	ns
72_h	WN	Route	0.9855	2	7.8365	0.4152	0.4338	0.5706	ns
72_h	MN	Route	2.7786	2	7.2716	0.1269	0.5443	0.2901	ns

For the pairwise comparison, we applied a Linear contrast expression. The output showed that there were statistically significant differences between time point for well-nourished, sand fly inoculated mice, at 24 h post infection between the well-nourished and malnourished groups for the needle inoculum and at 72 h post infection for the sand fly inoculation (Appendix table 14). Further, we observed differences in the well-nourished group at 24 h post infection between the sand fly inoculum and both, the control and needle inoculum. For the malnourished group we observed statistically significant p-values at 72 h post infection between the sand fly inolculum and the control group.

Appendix Table 14

Neutrophils: Pairwise comparison by Linear Contrast Expression

Predictor_1	Predictor_2	Group	Group.1	psihat	ci.lower	ci.upper	p.value	Sig.
Predictor: Time_point								
Ctrl	WN	24_h	72_h	0.0000	-1.2773	1.2773	1.0000	ns
Ctrl	MN	24_h	72_h	0.0000	-3.0375	3.0375	1.0000	ns
Needle	WN	24_h	72_h	1.3539	-0.2416	2.9494	0.0840	+
Needle	MN	24_h	72_h	-1.0936	-3.7554	1.5683	0.3486	ns
SF	WN	24_h	72_h	4.1934	3.0045	5.3822	0.0002	***
SF	MN	24_h	72_h	1.1910	-1.9272	4.3091	0.3803	ns
Predictor: Diet								
24_h	Ctrl	WN	MN	0.4045	-2.1397	2.9487	0.7043	ns
24_h	Needle	WN	MN	1.4936	0.1874	2.7999	0.0302	*
24_h	SF	WN	MN	1.6467	-1.4926	4.7860	0.2239	ns
72_h	Ctrl	WN	MN	0.4045	-2.1397	2.9487	0.7043	ns
72_h	Needle	WN	MN	-0.9538	-3.6657	1.7581	0.4279	ns
72_h	SF	WN	MN	-1.3556	-2.9135	0.2023	0.0795	+
Predictor: Route								
24_h	WN	Ctrl	Needle	-1.3581	-2.8960	0.1798	0.0309	*
24_h	WN	Ctrl	SF	-4.9856	-6.3932	-3.5781	0.0001	****
24_h	WN	Needle	SF	-3.6276	-4.8538	-2.4014	0.0001	****
24_h	MN	Ctrl	Needle	-0.2689	-3.6094	3.0716	0.8036	ns

24_h	MN	Ctrl	SF	-3.7434	-8.1407	0.6539	0.0534	+
24_h	MN	Needle	SF	-3.4745	-7.5673	0.6183	0.0534	+
72_h	WN	Ctrl	Needle	-0.0042	-2.1190	2.1106	0.9953	ns
72_h	WN	Ctrl	SF	-0.7923	-2.5263	0.9418	0.4595	ns
72_h	WN	Needle	SF	-0.7881	-2.9489	1.3727	0.4595	ns
72_h	MN	Ctrl	Needle	-1.3625	-5.3495	2.6245	0.3412	ns
72_h	MN	Ctrl	SF	-2.5524	-5.9102	0.8053	0.1555	ns
72_h	MN	Needle	SF	-1.1900	-4.6858	2.3059	0.3412	ns

Monocytes

Based on the assumption tests, we decided to apply a Robust three-way ANOVA to the Monocytes dataset to determine the effects of “Diet”, infection “Route” and time post infection (“Time_point”) on the Monocytes counts per pooled ear single-cell suspension (Appendix table 15). The test output showed that only the “Route” predictor was a statistically significant factor. No statistically significant interaction between any predictors was observed.

Appendix Table 15

Monocytes: Robust three-way ANOVA

Predictors	value	p.value	sig.
Diet	0.4677	0.5100	ns
Route	47.2342	0.0001	****
Time_point	3.5637	0.0760	+
Diet:Route	6.4411	0.0830	+
Diet:Time_point	2.4362	0.1370	ns
Route:Time_point	3.9590	0.1950	ns
Diet:Route:Time_point	2.2057	0.3830	ns

We looked for main effects by splitting the data by each predictor separately and analyzed the respective remaining two predictor by a Robust two-way ANOVA. The results showed that, after adjustment of p-values for multiple tests, only the “Route” predictor produced statistically significant p-values, regardless of whether the data was split by “Diet” or “Time-point” (Appendix table 16). This suggested that the only predictor of impact on cell counts was the route of infection. No statistically significant interactions were observed at this level.

Appendix Table 16

Monovytes: Robust two-way ANOVA

Grouper	Predictor	value	p.value	Sig.	p.value.adj	sig.
Grouped by Route						

Ctrl	Diet	5.2338	0.037	*	0.1110	ns
Ctrl	Time_point	0.0000	0.999	ns	0.9990	ns
Ctrl	Diet:Time_point	0.0000	0.999	ns	0.9990	ns
Needle	Diet	0.0138	0.909	ns	0.9990	ns
Needle	Time_point	6.2017	0.027	*	0.0945	+
Needle	Diet:Time_point	4.8351	0.047	*	0.1234	ns
SF	Diet	0.7354	0.413	ns	0.5964	ns
SF	Time_point	0.7811	0.399	ns	0.5964	ns
SF	Diet:Time_point	0.8265	0.387	ns	0.5964	ns
Grouped by Diet						
WN	Route	36.6533	0.001	***	0.0070	**
WN	Time_point	0.0245	0.878	ns	0.9990	ns
WN	Route:Time_point	0.0477	0.978	ns	0.9990	ns
MN	Route	32.3071	0.001	***	0.0070	**
MN	Time_point	5.9000	0.025	*	0.0945	+
MN	Route:Time_point	5.5106	0.107	ns	0.2043	ns
Grouped by Time_point						
24_h	Diet	3.9779	0.065	+	0.1517	ns
24_h	Route	21.9364	0.002	**	0.0105	*
24_h	Diet:Route	1.6608	0.478	ns	0.6274	ns
72_h	Diet	0.6652	0.426	ns	0.5964	ns
72_h	Route	46.8385	0.001	***	0.0070	**
72_h	Diet:Route	6.2872	0.078	+	0.1638	ns

For the analysis of the simple simple main effect for each respective predictor, we performed Robust one-way ANOVA with individual predictors of the data split by the other two predictors. The output showed that after the adjustment of p-values for multiple tests, only the "Route" predictor produce statistically significant p-values in the malnourished group at 72 h post infection (Appendix table 17).

Appendix Table 17

Monocytes: Robust one-way ANOVA

Predictor_1	Predictor_2	Effect	test	df1	df2	p.value	effsize	p.value.adj	sig.
Predictor: Time_point									
Ctrl	WN	Time_point	0.0000	1	8.0000	1.0000	0.0000	1.0000	ns
Ctrl	MN	Time_point	0.0000	1	8.0000	1.0000	0.0000	1.0000	ns
Needle	WN	Time_point	0.0512	1	6.2708	0.8281	0.1320	1.0000	ns
Needle	MN	Time_point	9.3895	1	5.7100	0.0236	0.9343	0.0942	+
SF	WN	Time_point	0.0010	1	7.9009	0.9756	0.0126	1.0000	ns

SF	MN	Time_point	0.9577	1	6.3626	0.3635	0.5490	0.5287	ns
Predictor: Diet									
24_h	Ctrl	Diet	2.6169	1	7.0286	0.1496	0.5569	0.2659	ns
24_h	Needle	Diet	6.5185	1	7.9850	0.0341	0.8413	0.1090	ns
24_h	SF	Diet	0.0009	1	4.8921	0.9767	0.0196	1.0000	ns
72_h	Ctrl	Diet	2.6169	1	7.0286	0.1496	0.5569	0.2659	ns
72_h	Needle	Diet	1.3637	1	7.6805	0.2779	0.5236	0.4446	ns
72_h	SF	Diet	2.6348	1	6.9154	0.1491	0.6089	0.2659	ns
Predictor: Route									
24_h	WN	Route	9.5871	2	7.8674	0.0078	0.8982	0.0622	+
24_h	MN	Route	4.6108	2	7.4049	0.0501	0.7502	0.1335	ns
72_h	WN	Route	7.7990	2	7.5548	0.0145	0.6443	0.0776	+
72_h	MN	Route	14.2513	2	7.7234	0.0026	0.7890	0.0409	*

For the pairwise comparison, we applied a Linear contrast expression. The output showed that at 24 h post infection, the sand fly infection route was statistically significant from the control and needle inoculum in the well-nourished (WN) group (Appendix table 18). At 72 h, the sand fly infection route was statistically significant from the control in the malnourished group. Further, statistically significant difference were observed between well-nourished and malnourished mice inoculated by needle at 24 h post infection and infected by sand fly at 72 h post infection for the needle and sand fly inoculum, respectively.

Appendix Table 18

Monocytes: Pairwise comparison by Linear Contrast Expression

Predictor_1	Predictor_2	Group	Group.1	psihat	ci.lower	ci.upper	p.value	Sig.
Predictor: Time_point								
Ctrl	WN	24_h	72_h	0.0000	-0.0791	0.0791	1.0000	ns
Ctrl	MN	24_h	72_h	0.0000	-0.1169	0.1169	1.0000	ns
Needle	WN	24_h	72_h	-0.0114	-0.1337	0.1108	0.8281	ns
Needle	MN	24_h	72_h	-0.1838	-0.3324	-0.0352	0.0236	*
SF	WN	24_h	72_h	0.0009	-0.0646	0.0664	0.9756	ns
SF	MN	24_h	72_h	-0.0633	-0.2195	0.0928	0.3635	ns
Predictor: Diet								
24_h	Ctrl	WN	MN	0.0700	-0.0322	0.1722	0.1496	ns
24_h	Needle	WN	MN	0.0908	0.0088	0.1728	0.0341	*
24_h	SF	WN	MN	0.0018	-0.1515	0.1551	0.9767	ns
72_h	Ctrl	WN	MN	0.0700	-0.0322	0.1722	0.1496	ns
72_h	Needle	WN	MN	-0.0816	-0.2439	0.0807	0.2779	ns

72_h	SF	WN	MN	-0.0624	-0.1535	0.0287	0.1491	ns
Predictor: Route								
24_h	WN	Ctrl	Needle	-0.0393	-0.1416	0.0629	0.2879	ns
24_h	WN	Ctrl	SF	-0.1324	-0.2246	-0.0401	0.0089	**
24_h	WN	Needle	SF	-0.0930	-0.1863	0.0002	0.0276	*
24_h	MN	Ctrl	Needle	-0.0185	-0.1522	0.1152	0.6868	ns
24_h	MN	Ctrl	SF	-0.2005	-0.4060	0.0049	0.0418	*
24_h	MN	Needle	SF	-0.1820	-0.3834	0.0194	0.0418	*
72_h	WN	Ctrl	Needle	-0.0507	-0.2096	0.1081	0.3508	ns
72_h	WN	Ctrl	SF	-0.1315	-0.2271	-0.0359	0.0109	*
72_h	WN	Needle	SF	-0.0807	-0.2388	0.0774	0.2280	ns
72_h	MN	Ctrl	Needle	-0.2023	-0.4017	-0.0030	0.0258	*
72_h	MN	Ctrl	SF	-0.2638	-0.4067	-0.1210	0.0018	**
72_h	MN	Needle	SF	-0.0615	-0.2581	0.1351	0.3639	ns

Statistical power

Considering the small group N=5 and the low occurrence of statistical significant outcomes, it stood to reason that the study design was statistically underpowered by necessity to keep animal numbers and costs manageable. Thus, we performed a retrospective power analysis on the data by cell-type group to explore this.

Effect size estimation based on partial eta^2

Effect sizes were calculated by predictor and the different potential interaction combinations of them. Appendix tables 19 to 21 show the respective effect sizes. Note that upper ends of the confidence intervals were automatically set to 1 for this type of calculation. Large effect sizes reflected statistically meaningful differences in the data analysis. The partial eta^2 values from the effect size calculation were then used for the retrospective power calculations.

Appendix Table 19

Myeloid cells: Effect size estimation

Parameter	Eta2_partial	CI_low	CI_high	Effect_size	Effect Size
Diet	0.0073	0.0000	1	very small	very small
Route	0.5033	0.3277	1	large	large
Time_point	0.0017	0.0000	1	very small	very small
Diet:Route	0.0398	0.0000	1	small	small
Diet:Time_point	0.1270	0.0174	1	small	medium
Route:Time_point	0.0125	0.0000	1	very small	small

Diet:Route:Time_point	0.0689	0.0000	1	small	medium

Appendix Table 20

Neutrophils: Effect size estimation

Parameter	Eta2_partial	CI_low	CI_high	Effect_size	Effect Size
Diet	0.0041	0.0000	1	very small	very small
Route	0.5220	0.3496	1	large	large
Time_point	0.1716	0.0398	1	medium	large
Diet:Route	0.0016	0.0000	1	very small	very small
Diet:Time_point	0.1087	0.0101	1	small	medium
Route:Time_point	0.2980	0.1175	1	large	large
Diet:Route:Time_point	0.0645	0.0000	1	small	medium

Appendix Table 21

Monocytes: Effect size estimation

Parameter	Eta2_partial	CI_low	CI_high	Effect_size	Effect Size
Diet	0.0204	0.0000	1	small	small
Route	0.4711	0.2909	1	large	large
Time_point	0.0894	0.0035	1	small	medium
Diet:Route	0.0775	0.0000	1	small	medium
Diet:Time_point	0.0668	0.0000	1	small	medium
Route:Time_point	0.0733	0.0000	1	small	medium
Diet:Route:Time_point	0.0476	0.0000	1	small	small

Retrospective minimum total sample size estimation for 80% power

The accepted rule of thumb is to have at least 80% (0.8 as ratio) statistical power. For small mean differences within data with a high level of complexity, this is often hard to achieve, because of cost and ability to manage large sample sizes. For Myeloid_cells, only the predictor(s) "Route" and in interaction(s) "Diet:Time_point" resulted in optimal sample sizes that were within the total sample size of N=60, 60, 60 (Appendix table 22). The large proposed sample sizes for "Diet" and "Time_point" on their own suggested little statistically significant difference between groups by individual predictor. Thus, the most meaningful predictor for Myeloid_cells counts was the route of infection; by needle, sand fly, or no-infection (control).

Appendix Table 22

Myeloid Cells: Minimum optimal sample size calculation

Effect	power	n.total	ncp	df1	df2

Diet					
Diet	0.8	1067	7.863	1	1064.116
Route					
Route	0.8	14	13.197	2	10.022
Time_point					
Time_point	0.8	4599	7.852	1	4596.543
Diet:Route					
Diet	0.8	192	7.931	1	185.328
Route	0.8	236	9.762	2	229.489
Diet:Route	0.8	236	9.762	2	229.489
Diet:Time_point					
Diet	0.8	57	8.149	1	52.006
Time_point	0.8	57	8.149	1	52.006
Diet:Time_point	0.8	57	8.149	1	52.006
Route:Time_point					
Route	0.8	762	9.673	2	755.924
Time_point	0.8	621	7.873	1	614.181
Route:Time_point	0.8	762	9.673	2	755.924
Diet:Route:Time_point					
Diet	0.8	109	8.008	1	96.268
Route	0.8	134	9.876	2	121.519
Time_point	0.8	109	8.008	1	96.268
Diet:Route	0.8	134	9.876	2	121.519
Diet:Time_point	0.8	109	8.008	1	96.268
Route:Time_point	0.8	134	9.876	2	121.519
Diet:Route:Time_point	0.8	134	9.876	2	121.519

Similar observation were made for Neutrophils counts, but here “Time_point” was a more meaningful predictor. Alongside “Route”, proposed sample sizes were well within the total sample size of N=60, 60, 60 (Appendix Table 23).

Appendix Table 23

Neutrophils: Minimum optimal sample size calculation

Effect	power	n.total	ncp	df1	df2

**Diet**					
Diet	0.8	1929	7.857	1	1926.454
**Route**					
Route	0.8	13	13.507	2	9.366
**Time_point**					
Time_point	0.8	40	8.266	1	37.898
**Diet:Route**					
Diet	0.8	4780	7.852	1	4773.506
Route	0.8	5868	9.640	2	5861.611
Diet:Route	0.8	5868	9.640	2	5861.611
**Diet:Time_point**					
Diet	0.8	67	8.098	1	62.403
Time_point	0.8	67	8.098	1	62.403
Diet:Time_point	0.8	67	8.098	1	62.403
**Route:Time_point**					
Route	0.8	27	11.202	2	20.386
Time_point	0.8	22	8.975	1	15.140
Route:Time_point	0.8	27	11.202	2	20.386
**Diet:Route:Time_point**					
Diet	0.8	117	7.996	1	104.054
Route	0.8	144	9.858	2	131.079
Time_point	0.8	117	7.996	1	104.054
Diet:Route	0.8	144	9.858	2	131.079
Diet:Time_point	0.8	117	7.996	1	104.054
Route:Time_point	0.8	144	9.858	2	131.079
Diet:Route:Time_point	0.8	144	9.858	2	131.079

For Monocytes counts, “Diet” became a more meaningful predictor, although it would still have required a 6.3 times larger total sample size than was available for the study (Appendix Table 24). “Time_point” was about as meaningful as it had been for the Myeloid_cells. Thus, only “Route” was a meaningful predictor that was on its own within proposed sample sizes ranges.

Appendix Table 24

Monocytes: Minimum optimal sample size calculation

Effect	power	n.total	ncp	df1	df2

Diet					
Diet	0.8	380	7.889	1	377.085
Route					
Route	0.8	15	12.722	2	11.281
Time_point					
Time_point	0.8	82	8.042	1	79.941
Diet:Route					
Diet	0.8	96	8.021	1	89.461
Route	0.8	118	9.898	2	111.798
Diet:Route	0.8	118	9.898	2	111.798
Diet:Time_point					
Diet	0.8	112	7.991	1	107.66
Time_point	0.8	112	7.991	1	107.66
Diet:Time_point	0.8	112	7.991	1	107.66
Route:Time_point					
Route	0.8	125	9.882	2	118.891
Time_point	0.8	102	8.010	1	95.238
Route:Time_point	0.8	125	9.882	2	118.891
Diet:Route:Time_point					
Diet	0.8	159	7.953	1	146.953
Route	0.8	196	9.794	2	183.745
Time_point	0.8	159	7.953	1	146.953
Diet:Route	0.8	196	9.794	2	183.745
Diet:Time_point	0.8	159	7.953	1	146.953
Route:Time_point	0.8	196	9.794	2	183.745
Diet:Route:Time_point	0.8	196	9.794	2	183.745

Retrospective calculation of statistical power in our data analysis

The observations from the retrospective minimum sample size calculation were reflected in the power calculation for our data. For Myeloid_cells counts, only “Route” and the interaction of “Diet:Time_point” had sufficient statistical power to have a change to find statistically meaning differences in the data (Appendix Table 25).

Appendix Table 25

Myeloid Cells: Statistical power of data

Effect	power	n.total	ncp	df1	df2
Diet					
Diet	0.1	60	0.443	1	58
Route					
Route	1	60	60.805	2	57
Time_point					
Time_point	0.061	60	0.102	1	58
Diet:Route					
Diet	0.341	60	2.487	1	54
Route	0.259	60	2.487	2	54
Diet:Route	0.259	60	2.487	2	54
Diet:Time_point					
Diet	0.827	60	8.73	1	56
Time_point	0.827	60	8.73	1	56
Diet:Time_point	0.827	60	8.73	1	56
Route:Time_point					
Route	0.108	60	0.762	2	54
Time_point	0.138	60	0.762	1	54
Route:Time_point	0.108	60	0.762	2	54
Diet:Route:Time_point					
Diet	0.542	60	4.438	1	48
Route	0.431	60	4.438	2	48
Time_point	0.542	60	4.438	1	48
Diet:Route	0.431	60	4.438	2	48
Diet:Time_point	0.542	60	4.438	1	48
Route:Time_point	0.431	60	4.438	2	48
Diet:Route:Time_point	0.431	60	4.438	2	48

For Neutrophils counts, “Route” and “Time_points as much as their interaction had >80% statistical power, while the interaction”Diet:Time_point” was close to 80% statistical power (Appendix Table 26).

Appendix Table 26

Neutrophils: Statistical power of data

Effect	power	n.total	ncp	df1	df2
Diet					
Diet	0.078	60	0.244	1	58
Route					
Route	1	60	65.534	2	57
Time_point					
Time_point	0.934	60	12.431	1	58
Diet:Route					
Diet	0.061	60	0.099	1	54
Route	0.057	60	0.099	2	54
Diet:Route	0.057	60	0.099	2	54
Diet:Time_point					
Diet	0.758	60	7.317	1	56
Time_point	0.758	60	7.317	1	56
Diet:Time_point	0.758	60	7.317	1	56
Route:Time_point					
Route	0.995	60	25.473	2	54
Time_point	0.999	60	25.473	1	54
Route:Time_point	0.995	60	25.473	2	54
Diet:Route:Time_point					
Diet	0.513	60	4.134	1	48
Route	0.405	60	4.134	2	48
Time_point	0.513	60	4.134	1	48
Diet:Route	0.405	60	4.134	2	48
Diet:Time_point	0.513	60	4.134	1	48
Route:Time_point	0.405	60	4.134	2	48
Diet:Route:Time_point	0.405	60	4.134	2	48

Similar to Myeloid_cells counts, for Monocytes counts, “Route” was the only predictor that produce >80% statistical power on its own, but not in any interaction with “Diet” and/or “Time_point” (Appendix Table 27).

Appendix Table 27

Monocytes: Statistical power of data

Effect	power	n.total	ncp	df1	df2
Diet					
Diet	0.196	60	1.249	1	58
Route					
Route	1	60	53.449	2	57
Time_point					
Time_point	0.665	60	5.888	1	58
Diet:Route					
Diet	0.597	60	5.041	1	54
Route	0.484	60	5.041	2	54
Diet:Route	0.484	60	5.041	2	54
Diet:Time_point					
Diet	0.531	60	4.294	1	56
Time_point	0.531	60	4.294	1	56
Diet:Time_point	0.531	60	4.294	1	56
Route:Time_point					
Route	0.460	60	4.747	2	54
Time_point	0.571	60	4.747	1	54
Route:Time_point	0.460	60	4.747	2	54
Diet:Route:Time_point					
Diet	0.397	60	3.002	1	48
Route	0.304	60	3.002	2	48
Time_point	0.397	60	3.002	1	48
Diet:Route	0.304	60	3.002	2	48
Diet:Time_point	0.397	60	3.002	1	48
Route:Time_point	0.304	60	3.002	2	48
Diet:Route:Time_point	0.304	60	3.002	2	48

Conclusion

In conclusion, it can be said that the infection route (needle, sand fly, or uninfected) was the primary difference maker with respect to observed Myeloid_cells, Neutrophils and Monocytes counts in pooled mouse ear single-cell suspensions. It is of note that the nutritional status of the individuals did not have a

detectable impact on the observed cell counts in the site of infection.

Panel e and f

Data analysis

Figure 1 e and f presented the same samples as shown in figure 1b and c with the added difference that only IL-1β^{+} cells were counted. Again, the outcome variable of total IL-1β^{+} Myeloid_cells counts and separately, IL-1β^{+} Neutrophils and Monocytes counts from N=40, 40, 40 pools of single cell suspensions, respectively, prepared from BALB/c mouse ears collected 24 h and 72 h post infection with *Leishmania donovani* by "needle" inoculation or infective sand flies (SF) bites. Please, refer to the methods section of the publication for more details on sample preparation. Note that different mice were sampled at 24 h and 72 h post infection, which meant that this dataset satisfied the independence of data points and therefore, did not represent a repeatedly measured dataset. Thus, this dataset contained three between-subject factors, "Diet" (well-nourished [WN] or malnourished [MN]), "Route" (uninfected control [Ctrl], needle inoculation [Needle], infective sand fly bites [SF]) and "Time_point" (collection at 24 h or 72 h). Based on this information, a three-way analysis was indicated.

Thus, we assessed the data for compliance with assumptions for a three-way ANOVA:

- Data normality
- Homogeneity of variance
- No significant outliers

Initial assumption assessment indicated that data transformation was required to meet assumptions for a three-way ANOVA only for the Neutrophils counts. Thus, we settled for a Box-Cox power transformation. Conversely, Monocytes did not need to be transformed to apply a Robust three-way ANOVA, while Neutrophils were analyzed by Simple linear regression post Box-Cox power transformation. The data transformation resulted in different data distributions and variances than appear in the main figure in the publication.

Assumption analyses

Data normality

The assessment of the transformed data distribution for each group was conducted by Shpiro-Wilks test and QQ-plot for counts of Myeloid_cells, Neutrophils and Monocytes separately. Note that all groups of all datasets consisted of N=5 pools of mouse ear single-cell suspensions, which made it difficult to assess data distribution reliably by Shapiro-Wilks test.

Myeloid_cells

In spite of assessment limitations due to small group sizes, we concluded based on Shapiro-Wilks test (Appendix table 28) and QQ-plots (Fig.1e-f-1) that all groups of the dataset were likely to follow a normal

distribution with the potential exception of well-nourished, sand fly inoculated mice at 24 h post infection.

Appendix Table 28

Myeloid cells: Univariate Shapiro-Wilks test results

Diet	Route	Time_point	variable	statistic	p	Outcome
WN	Needle	24_h	Counts	0.9773	0.9198	ns
WN	Needle	72_h	Counts	0.9486	0.7275	ns
MN	Needle	24_h	Counts	0.8686	0.2608	ns
MN	Needle	72_h	Counts	0.9146	0.4960	ns
WN	SF	24_h	Counts	0.7364	0.0222	sig.
WN	SF	72_h	Counts	0.8934	0.3742	ns
MN	SF	24_h	Counts	0.8915	0.3649	ns
MN	SF	72_h	Counts	0.9664	0.8520	ns

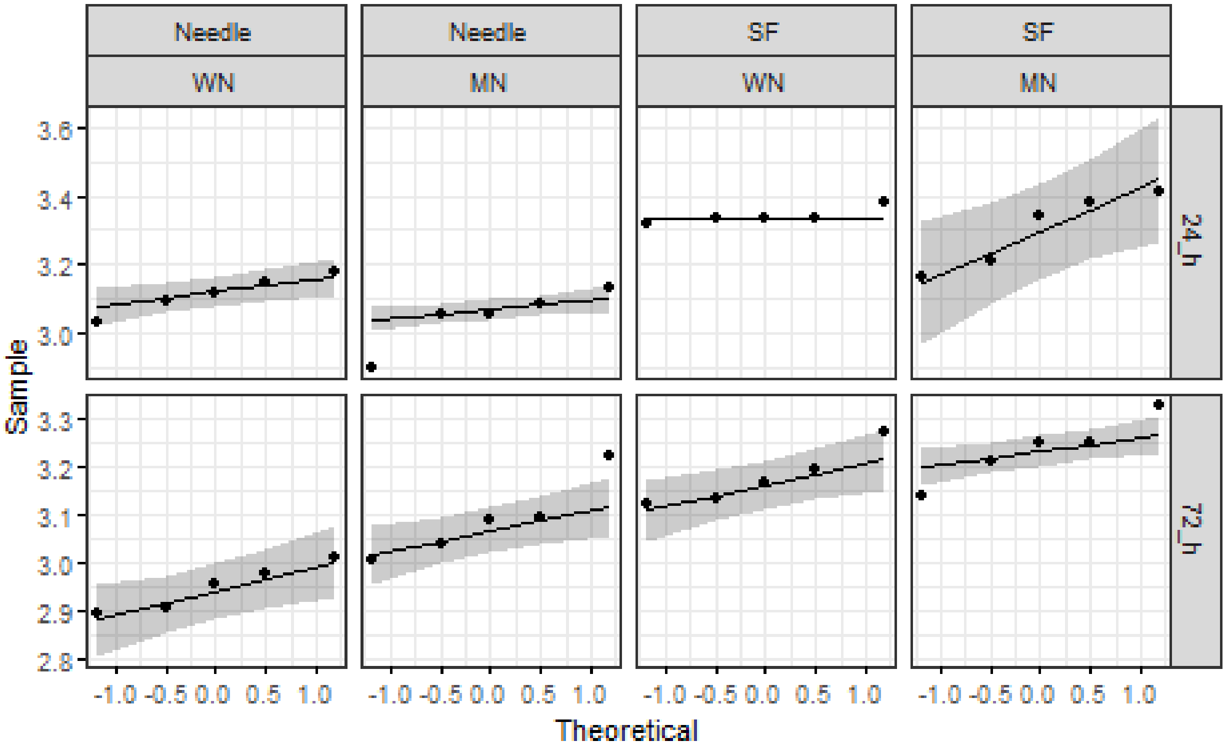

Fig.1e-f-1: QQ-plots of myeloid cell counts split into groups by predictor variables

Neutrophils

In spite of assessment limitations due to small group sizes, We concluded based on Shapiro-Wilks test (Appendix table 29) and QQ-plots (Fig.1e-f-2) that all groups of the dataset were likely to follow a normal distribution.

Appendix Table 29

Neutrophils: Univariate Shapiro-Wilks test results

Diet	Route	Time_point	variable	statistic	p	Outcome
WN	Needle	24_h	Counts	0.9664	0.8514	ns
WN	Needle	72_h	Counts	0.9196	0.5271	ns
MN	Needle	24_h	Counts	0.9650	0.8420	ns
MN	Needle	72_h	Counts	0.8507	0.1967	ns
WN	SF	24_h	Counts	0.9704	0.8776	ns
WN	SF	72_h	Counts	0.9251	0.5632	ns
MN	SF	24_h	Counts	0.9265	0.5725	ns
MN	SF	72_h	Counts	0.9361	0.6383	ns

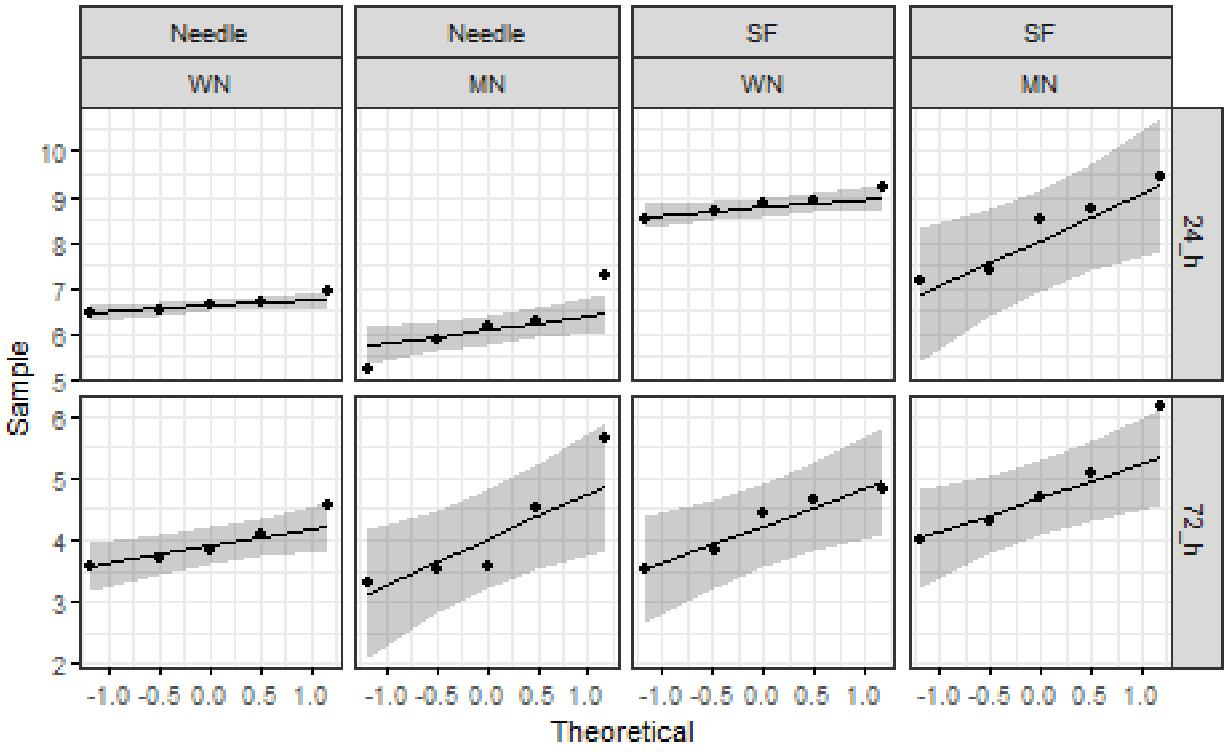

Fig.1e-f-2: QQ-plots of neutrophil counts split into groups by predictor variables

Monocytes

In spite of assessment limitations due to small group sizes, we concluded based on Shapiro-Wilks test (Appendix table 30) and QQ-plots (Fig.1e-f-3) that all groups of the dataset were likely to follow a normal distribution.

Appendix Table 30

Monocytes: Univariate Shapiro-Wilks test results

Diet	Route	Time_point	variable	statistic	p	Outcome
WN	Needle	24_h	Counts	0.9542	0.7674	ns
WN	Needle	72_h	Counts	0.8933	0.3740	ns
MN	Needle	24_h	Counts	0.9561	0.7806	ns
MN	Needle	72_h	Counts	0.8828	0.3221	ns
WN	SF	24_h	Counts	0.8563	0.2151	ns
WN	SF	72_h	Counts	0.9219	0.5422	ns
MN	SF	24_h	Counts	0.9430	0.6874	ns
MN	SF	72_h	Counts	0.8849	0.3319	ns

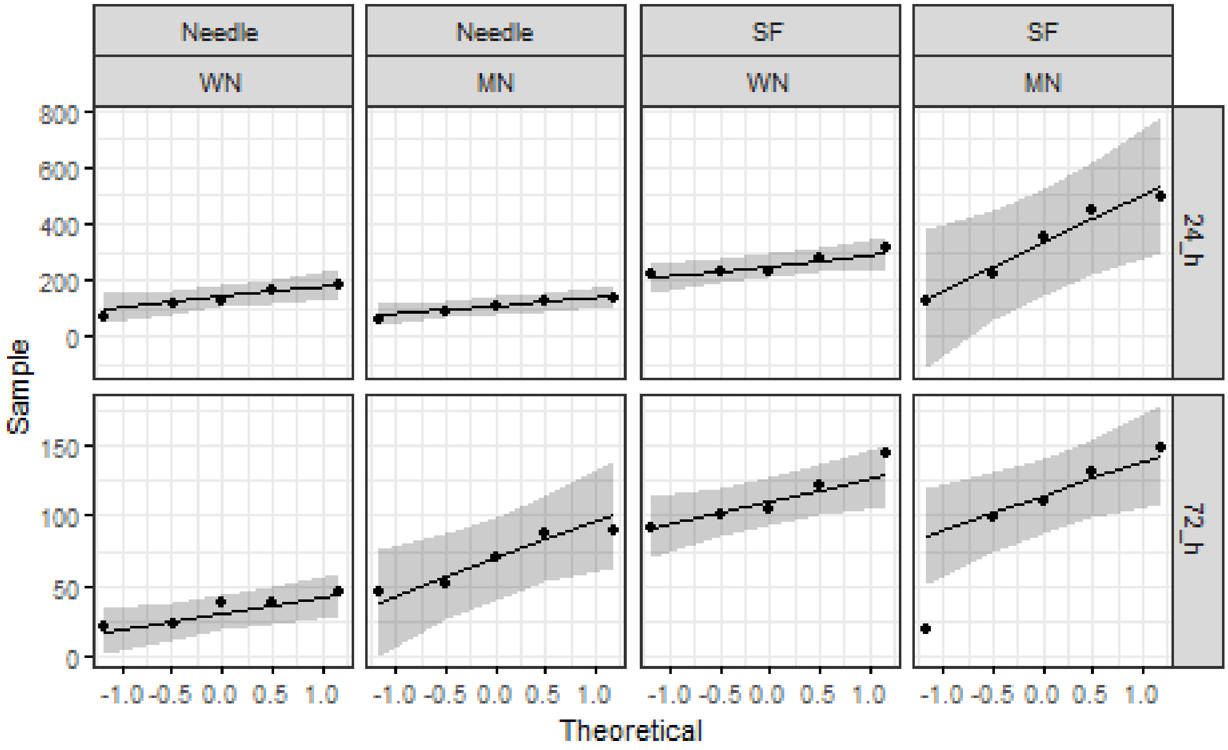

Fig.1e-f-3: QQ-plots of monocyte counts split into groups by predictor variables

Homogeneity of variance The assessment of homogeneity of variance was also conducted for myeloid cell, neutrophil and monocyte counts separately. We employed the Levene's test on each dataset. The p-value for the test suggested that the assumption of homogeneity of variance held for Myeloid_cells (p=0.5389), held for Neutrophils (p=0.401), and was rejected for Monocytes (p=0.0011).

Outliers It can be difficult to determine outliers in small datasets reliably as the analysis is dependent on the interquartile range of the data per group. We attempted it anyway and found a potential 6 outliers in

the Myeloid_cells dataset (Appendix table 31), 2 outliers in the Neutrophils dataset (Appendix table 32), and 1 outliers in the Monocytes dataset (Appendix table 33), of which some were classified as extreme. However, larger datasets may have found these not be outliers, but just peripheral.

Appendix Table 31

Myeloid cells: List of possible outliers

Diet	Route	Time_point	is.outlier	is.extreme
MN	Needle	24_h	TRUE	TRUE
MN	Needle	72_h	TRUE	FALSE
WN	SF	24_h	TRUE	TRUE
WN	SF	24_h	TRUE	TRUE
MN	SF	72_h	TRUE	FALSE
MN	SF	72_h	TRUE	FALSE

Appendix Table 32

Neutrophils: List of possible outliers

Diet	Route	Time_point	is.outlier	is.extreme
MN	Needle	24_h	TRUE	FALSE
MN	Needle	24_h	TRUE	FALSE

Appendix Table 33

Monocytes: List of possible outliers

Diet	Route	Time_point	is.outlier	is.extreme
MN	SF	72_h	TRUE	FALSE

Three-way analysis

The appropriate three-way analysis was performed for Myeloid_cells, Neutrophils and Monocytes separately.

Myeloid_cells

Based on the assumption tests, we decided to apply a Simple linear regression to the Myeloid_cells dataset to determine the effects of “Diet”, infection “Route” and time post infection (“Time_point”) on the Myeloid_cells counts per pooled ear single-cell suspension (Appendix table 34). The appropriateness of the Simple linear regression was confirmed by checking the model residuals for normal distribution (Shapiro-Wilks test: 0.771; Fig.1e-f-4).

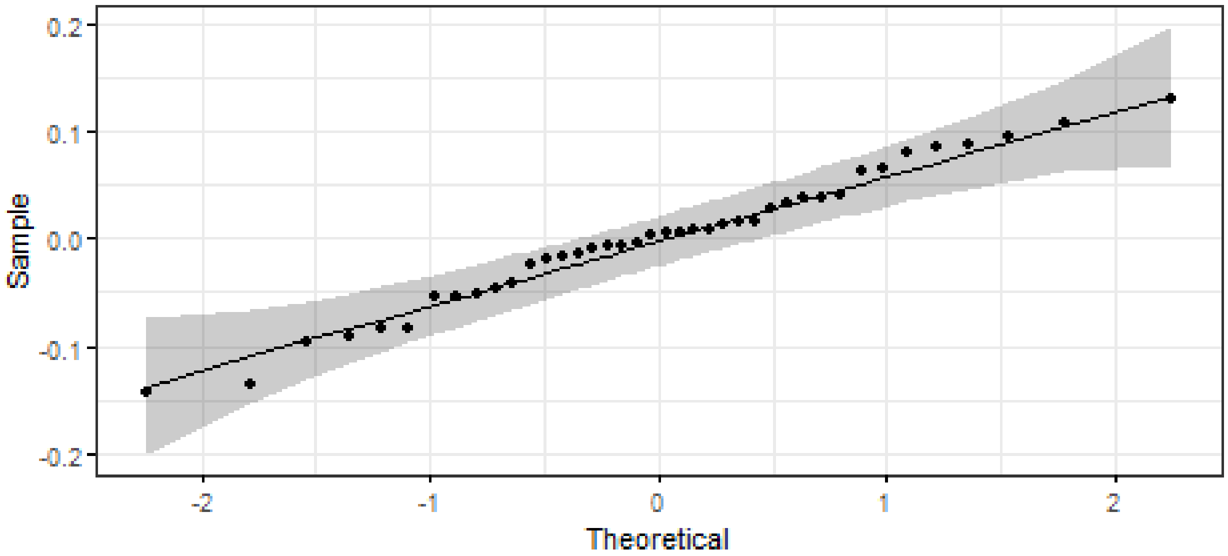

Fig.1e-f-4: QQ-plots of monocyte counts split into groups by predictor variables

The test output of the Simple linear regression showed that only the "Route" and "Time_point" were statistically significant predictors , while the interaction between Time_point and Diet was statistically significant, too. The importance of the "Time_point" predictor was a significant difference for the IL-1β^{+} Myeloid_cells counts, compared to observations in figure 1b & c, when IL-1β^{+} was not considered.

Appendix Table 34

Myeloid cells: Simple linear regression

Predictors	Df	Sum Sq	Mean Sq	F value	Pr(>F)	sig.
Diet	1	0.0056	0.0056	1.1286	0.2960	ns
Route	1	0.4567	0.4567	91.9654	<0.0001	****
Time_point	1	0.0750	0.0750	15.1095	0.0005	***
Diet:Route	1	0.0016	0.0016	0.3309	0.5692	ns
Diet:Time_point	1	0.0585	0.0585	11.7781	0.0017	**
Route:Time_point	1	0.0069	0.0069	1.3874	0.2475	ns
Diet:Route:Time_point	1	0.0080	0.0080	1.6065	0.2141	ns

As we performed a Simple linear regression, we were not able to analyze main and simple simple effects of predictors, but moved immediately to perform a pairwise comparison. We applied a Estimated marginal means analysis and the output showed that at 24 h post infection, the sand fly infection route was statistically significant from the control and needle inoculum in the well-nourished (WN) group (Appendix table 37). At 72 h, the sand fly infection route was statistically significant from the control in the malnourished group. Further, statistically significant difference were observed between well-nourished and malnourished mice inoculated by needle at 24 h post infection and infected by sand fly at 72 h post infection.

Appendix Table 37

Myeloid cells: Pairwise comparison by Estimated marginal means analysis

Pairing	estimate	SE	df	t.ratio	p.value	sig.
WN Needle 24_h - MN Needle 24_h	0.0682	0.0446	32	1.5309	0.9831	ns
WN Needle 24_h - WN SF 24_h	-0.2245	0.0446	32	-5.0377	0.0005	***
WN Needle 24_h - WN Needle 72_h	0.1651	0.0446	32	3.7043	0.0221	*
MN Needle 24_h - MN SF 24_h	-0.2554	0.0446	32	-5.7300	0.0001	****
MN Needle 24_h - MN Needle 72_h	-0.0443	0.0446	32	-0.9951	1.0000	ns
WN SF 24_h - MN SF 24_h	0.0374	0.0446	32	0.8386	1.0000	ns
WN SF 24_h - WN SF 72_h	0.1611	0.0446	32	3.6147	0.0282	*
MN SF 24_h - MN SF 72_h	0.0646	0.0446	32	1.4503	0.9915	ns
WN Needle 72_h - MN Needle 72_h	-0.1412	0.0446	32	-3.1685	0.0900	+
WN Needle 72_h - WN SF 72_h	-0.2285	0.0446	32	-5.1274	0.0004	***
MN Needle 72_h - MN SF 72_h	-0.1464	0.0446	32	-3.2846	0.0671	+
WN SF 72_h - MN SF 72_h	-0.0591	0.0446	32	-1.3258	0.9976	ns

Neutrophils

Based on the assumption tests, we decided to apply a Robust three-way ANOVA to the Neutrophils dataset to determine the effects of “Diet”, infection “Route” and time post infection (“Time_point”) on the Neutrophils counts per pooled ear single-cell suspension (Appendix table 38). As for Myeloid_cells counts, the test output showed that “Route” and “Time_point” were both statistically significant predictors, while here, their interaction was also statistically meaningful rather than “Diet: and”Time_point”.

Appendix Table 38

Neutrophils: Robust three-way ANOVA

Predictors	value	p.value	sig.
Diet	0.6656	0.4400	ns
Route	37.4846	0.0001	****
Time_point	211.6458	0.0010	***
Diet:Route	0.0973	0.7640	ns
Diet:Time_point	2.7393	0.1340	ns
Route:Time_point	11.3037	0.0080	**
Diet:Route:Time_point	0.3065	0.5960	ns

We looked for main effects by splitting the data by each predictor separately and analyzed the respective remaining two predictor by a Robust two-way ANOVA. After adjustment of p-values for multiple tests, we observed statistically significant differences for the “Time_point” variable, when the data was split by “Route” or by “Diet”, for the well-nourished group for Route, Time_point and their interaction were

statistically meaningful, while for the malnourished state, there was no meaningful interaction between these predictors when the data was split by “Diet”, and “Route” was only meaningful at 24 h post infection, when the data was split by Time_point (Appendix table 39).

Appendix Table 39

Neutrophils: Robust two-way ANOVA

Grouper	Predictor	value	p.value	Sig.	p.value.adj	sig.
Grouped by Route						
Needle	Diet	0.2902	0.603	ns	0.6784	ns
Needle	Time_point	68.5031	0.001	***	0.0026	**
Needle	Diet:Time_point	1.2155	0.297	ns	0.3819	ns
SF	Diet	0.0001	0.993	ns	0.9930	ns
SF	Time_point	159.7806	0.001	***	0.0026	**
SF	Diet:Time_point	3.4747	0.086	+	0.1548	ns
Grouped by Diet						
WN	Route	55.4130	0.001	***	0.0026	**
WN	Time_point	479.7172	0.001	***	0.0026	**
WN	Route:Time_point	31.7623	0.001	***	0.0026	**
MN	Route	12.4507	0.003	**	0.0068	**
MN	Time_point	48.2204	0.001	***	0.0026	**
MN	Route:Time_point	2.8633	0.110	ns	0.1800	ns
Grouped by Time_point						
24_h	Diet	3.6650	0.085	+	0.1548	ns
24_h	Route	57.8683	0.001	***	0.0026	**
24_h	Diet:Route	0.0452	0.837	ns	0.8862	ns
72_h	Diet	1.3260	0.272	ns	0.3766	ns
72_h	Route	2.4858	0.140	ns	0.2100	ns
72_h	Diet:Route	0.4236	0.528	ns	0.6336	ns

For the analysis of the simple simple main effect for each respective predictor, we performed Robust one-way ANOVA with individual predictors of the data split by the other two predictors. The output showed “Time_point” post infestation was statistically significant across the board, while “Route” was only statistically significant during the 24 h time point. “Diet” played not significant role as a predictor (Appendix table 40).

Appendix Table 40

Neutrophils: Robust one-way ANOVA

Predictor_1	Predictor_2	Effect	test	df1	df2	p.value	effsize	p.value.adj	sig.
Predictor: Time_point									
Needle	WN	Time_point	203.3793	1	5.9585	<0.0001	1.1183	<0.0001	****
Needle	MN	Time_point	14.4271	1	7.3720	0.0061	1.0154	0.0122	*
SF	WN	Time_point	280.9801	1	5.5483	<0.0001	1.1235	<0.0001	****
SF	MN	Time_point	35.7185	1	7.8502	0.0004	1.0791	0.0011	**
Predictor: Diet									
24_h	Needle	Diet	1.9911	1	4.5651	0.2226	0.7259	0.2945	ns
24_h	SF	Diet	1.7774	1	4.5402	0.2454	0.5247	0.2945	ns
72_h	Needle	Diet	0.1201	1	5.1645	0.7426	0.2162	0.7426	ns
72_h	SF	Diet	1.6983	1	6.9708	0.2339	0.7251	0.2945	ns
Predictor: Route									
24_h	WN	Route	236.8953	1	7.5189	<0.0001	1.1347	<0.0001	****
24_h	MN	Route	14.6124	1	7.4408	0.0058	1.0516	0.0122	*
72_h	WN	Route	0.9975	1	7.0332	0.3510	0.4008	0.3829	ns
72_h	MN	Route	1.5798	1	7.8076	0.2451	0.5136	0.2945	ns

For the pairwise comparison, we applied a Linear contrast expression. As all three factors where dichotomous, the pairwise comparison reflected the one-way ANVOA results (Appendix table 41).

Appendix Table 41

Neutrophils: Pairwise comparison by Linear Contrast Expression

Predictor_1	Predictor_2	Group	Group.1	psihat	ci.lower	ci.upper	p.value	Sig.
Predictor: Time_point								
Needle	WN	24_h	72_h	2.7044	2.2396	3.1692	<0.0001	****
Needle	MN	24_h	72_h	2.0686	0.7938	3.3433	0.0061	**
SF	WN	24_h	72_h	4.5783	3.8966	5.2600	<0.0001	****
SF	MN	24_h	72_h	3.4015	2.0847	4.7184	0.0004	***
Predictor: Diet								
24_h	Needle	WN	MN	0.4732	-0.4142	1.3606	0.2226	ns
24_h	SF	WN	MN	0.5915	-0.5846	1.7676	0.2454	ns
72_h	Needle	WN	MN	-0.1626	-1.3570	1.0319	0.7426	ns
72_h	SF	WN	MN	-0.5853	-1.6481	0.4776	0.2339	ns
Predictor: Route								
24_h	WN	Needle	SF	-2.1745	-2.5040	-1.8451	<0.0001	****

24_h	MN	Needle	SF	-2.0563	-3.3131	-0.7994	0.0058	**
72_h	WN	Needle	SF	-0.3006	-1.0117	0.4104	0.3510	ns
72_h	MN	Needle	SF	-0.7233	-2.0561	0.6095	0.2451	ns

Monocytes

Based on the assumption tests, we decided to apply a Robust three-way ANOVA to the Monocytes dataset to determine the effects of "Diet", infection "Route" and time post infection ("Time_point") on the Monocytes counts per pooled ear single-cell suspension (Appendix table 42). As for Myeloid_cells and Neutrophils counts, "Route" and "Time_point" were statistically significant predictors. However, no significant interaction between predictors was observed for Monocytes counts.

Appendix Table 42

Monocytes: Robust three-way ANOVA

Predictors	value	p.value	sig.
Diet	1.1474	0.360	ns
Route	20.1101	0.006	**
Time_point	24.8237	0.003	**
Diet:Route	0.8115	0.428	ns
Diet:Time_point	0.0604	0.822	ns
Route:Time_point	4.6317	0.096	+
Diet:Route:Time_point	2.4210	0.200	ns

We looked for main effects by splitting the data by each predictor separately and analyzed the respective remaining two predictor by a Robust two-way ANOVA. After adjustment of p-values for multiple tests, we observed statistically significant differences for the "Time_point" variable, when the data was split by "Route" or by "Diet"; in well-nourished and malnourished groups we observed statistical significance for "Route" and "Time_point", while no significant interactions were observed when the data was split by "Diet"; and for "Route" was meaningful at both, 24 h and 72 h post infection, when the data was split by Time_point (Appendix table 43). Overall, "Diet" played no meaningful role as a predictor.

Appendix Table 43

Monovytes: Robust two-way ANOVA

Grouper	Predictor	value	p.value	Sig.	p.value.adj	sig.
Grouped by Route						
Needle	Diet	0.0991	0.760	ns	0.7600	ns
Needle	Time_point	25.6851	0.001	***	0.0045	**
Needle	Diet:Time_point	5.7556	0.034	*	0.0680	+
SF	Diet	0.6918	0.437	ns	0.4916	ns

SF	Time_point	23.4448	0.001	***	0.0045	**
SF	Diet:Time_point	1.2852	0.298	ns	0.3831	ns
Grouped by Diet						
WN	Route	47.9950	0.001	***	0.0045	**
WN	Time_point	68.8826	0.001	***	0.0045	**
WN	Route:Time_point	1.9584	0.189	ns	0.2835	ns
MN	Route	11.5996	0.010	**	0.0225	*
MN	Time_point	12.2280	0.009	**	0.0225	*
MN	Route:Time_point	6.4702	0.038	*	0.0684	+
Grouped by Time_point						
24_h	Diet	0.3760	0.562	ns	0.5951	ns
24_h	Route	20.4756	0.002	**	0.0060	**
24_h	Diet:Route	1.8171	0.223	ns	0.3088	ns
72_h	Diet	0.8951	0.373	ns	0.4476	ns
72_h	Route	18.2554	0.002	**	0.0060	**
72_h	Diet:Route	3.2974	0.105	ns	0.1718	ns

For the analysis of the simple simple main effect for each respective predictor, we performed Robust one-way ANOVA with individual predictors of the data split by the other two predictors. The output showed “Time_point” post infestation was statistically significant across the board with the exception for malnourished individuals inoculated by needle, while “Route” was only statistically significant during the within the well-nourished group at either time point. “Diet” was only statistically significant for the needle inoculated groups at 72 h post infection (Appendix table 44).

Appendix Table 44

Monocytes: Robust one-way ANOVA

Predictor_1	Predictor_2	Effect	test	df1	df2	p.value	effsize	p.value.adj	sig.
Predictor: Time_point									
Needle	WN	Time_point	24.2428	1	4.4410	0.0060	1.0016	0.0181	*
Needle	MN	Time_point	4.1902	1	6.5974	0.0823	0.7082	0.1235	ns
SF	WN	Time_point	46.2024	1	5.9611	0.0005	1.1175	0.0031	**
SF	MN	Time_point	9.6448	1	4.8214	0.0280	0.9127	0.0528	+
Predictor: Diet									
24_h	Needle	Diet	1.2696	1	7.4413	0.2949	0.4586	0.3538	ns
24_h	SF	Diet	1.0760	1	4.5597	0.3514	0.4648	0.3834	ns
72_h	Needle	Diet	12.7301	1	5.9683	0.0119	0.8820	0.0286	*
72_h	SF	Diet	0.2225	1	5.3766	0.6557	0.3242	0.6557	ns

Predictor: Route									
24_h	WN	Route	19.9581	1	7.9596	0.0021	1.1520	0.0085	**
24_h	MN	Route	9.8710	1	4.3690	0.0308	0.8864	0.0528	+
72_h	WN	Route	58.1556	1	5.8388	0.0003	1.1400	0.0031	**
72_h	MN	Route	1.7950	1	5.2796	0.2351	0.5765	0.3135	ns

For the pairwise comparison, we applied a Linear contrast expression. As all three factors where dichotomous, the pairwise comparison reflected the one-way ANVOA results (Appendix table 45).

Appendix Table 45

Monocytes: Pairwise comparison by Linear Contrast Expression

Predictor_1	Predictor_2	Group	Group.1	psihat	ci.lower	ci.upper	p.value	Sig.
Predictor: Time_point								
Needle	WN	24_h	72_h	99.6	45.5687	153.6313	0.0060	**
Needle	MN	24_h	72_h	35.6	-6.0383	77.2383	0.0823	+
SF	WN	24_h	72_h	140.0	89.5221	190.4779	0.0005	***
SF	MN	24_h	72_h	225.6	36.7654	414.4346	0.0280	*
Predictor: Diet								
24_h	Needle	WN	MN	27.8	-29.8466	85.4466	0.2949	ns
24_h	SF	WN	MN	-74.2	-263.5526	115.1526	0.3514	ns
72_h	Needle	WN	MN	-36.2	-61.0582	-11.3418	0.0119	*
72_h	SF	WN	MN	11.4	-49.4453	72.2453	0.6557	ns
Predictor: Route								
24_h	WN	Needle	SF	-120.2	-182.2997	-58.1003	0.0021	**
24_h	MN	Needle	SF	-222.2	-412.1896	-32.2104	0.0308	*
72_h	WN	Needle	SF	-79.8	-105.5774	-54.0226	0.0003	***
72_h	MN	Needle	SF	-32.2	-93.0104	28.6104	0.2351	ns

Statistical power

Considering the small group N=5, it stood to reason that the study design was statistically underpowered by necessity to keep animal number and costs manageable. Thus, we performed a retrospective power analysis on the data by cell-type group to explore this.

Effect size estimation based on partial eta^2

Effect sizes were calculated by predictor and the different potential interaction combinations of them. Appendix tables 46 to 48 show the respective effect sizes. Note that upper ends of the confidence intervals

were automatically set to 1 for this type of calculation. Large effect sizes reflected statistically meaningful differences in the data analysis. The partial eta^2 values from the effect size calculation were then used to the retrospective power calculations.

Appendix Table 46

Myeloid cells: Effect size estimation

Parameter	Eta2_partial	CI_low	CI_high	Effect_size
Diet	0.0125	0.0000	1	very small
Route	0.6490	0.4742	1	large
Time_point	0.3162	0.1108	1	large
Diet:Route	0.0001	0.0000	1	very small
Diet:Time_point	0.0965	0.0000	1	small
Route:Time_point	0.2078	0.0382	1	medium
Diet:Route:Time_point	0.0001	0.0000	1	very small

Appendix Table 47

Neutrophils: Effect size estimation

Parameter	Eta2_partial	CI_low	CI_high	Effect_size
Diet	0.0330	0.0000	1	small
Route	0.5814	0.3864	1	large
Time_point	0.7717	0.6469	1	large
Diet:Route	0.0117	0.0000	1	very small
Diet:Time_point	0.0758	0.0000	1	small
Route:Time_point	0.5408	0.3368	1	large
Diet:Route:Time_point	0.0223	0.0000	1	small

Appendix Table 48

Monocytes: Effect size estimation

Parameter	Eta2_partial	CI_low	CI_high	Effect_size
Diet	0.0241	0.0000	1	small
Route	0.5015	0.2911	1	large
Time_point	0.5500	0.3478	1	large
Diet:Route	0.0142	0.0000	1	very small
Diet:Time_point	0.0023	0.0000	1	very small
Route:Time_point	0.2055	0.0370	1	medium
Diet:Route:Time_point	0.0983	0.0000	1	small

Retrospective minimum total sample size estimation for 80% power

The accepted rule of thumb is to have at least 80% (0.8 as ratio) statistical power in once data. For small mean differences within data with a high level of complexity, this is often hard to achieve, because of cost and ability to manage large sample sizes. For Myeloid_cells, the predictors “Route” and “Time_point” as much as the interaction “Diet:Time_point” resulted in optimal sample sizes that were within the total sample size of N=40, 40, 40 (Appendix table 49). The large proposed sample sizes for “Diet” on its own and within most of its interactions suggested little statistically significant difference between groups with respect to “Diet”.

Appendix Table 49

Myeloid Cells: Minimum optimal sample size calculation

Effect	power	n.total	ncp	df1	df2
Diet					
Diet	0.8	620	7.873	1	617.5
Route					
Route	0.8	7	12.601	1	4.815
Time_point					
Time_point	0.8	20	8.832	1	17.095
Diet:Route					
Diet	0.8	64408	7.849	1	64403.09
Route	0.8	64408	7.849	1	64403.09
Diet:Route	0.8	64408	7.849	1	64403.09
Diet:Time_point					
Diet	0.8	76	8.065	1	71.503
Time_point	0.8	76	8.065	1	71.503
Diet:Time_point	0.8	76	8.065	1	71.503
Route:Time_point					
Route	0.8	33	8.422	1	28.106
Time_point	0.8	33	8.422	1	28.106
Route:Time_point	0.8	33	8.422	1	28.106
Diet:Route:Time_point					
Diet	0.8	83400	7.849	1	83391.34
Route	0.8	83400	7.849	1	83391.34
Time_point	0.8	83400	7.849	1	83391.34
Diet:Route	0.8	83400	7.849	1	83391.34

Diet:Time_point	0.8	83400	7.849	1	83391.34
Route:Time_point	0.8	83400	7.849	1	83391.34
Diet:Route:Time_point	0.8	83400	7.849	1	83391.34

Similar observation were made for Neutrophils counts, but here the interaction "Route:Time_point" was within the total sample size of N=40, 40, 40 (Appendix Table 50). The involvement of "Diet" produced large minimum sample sizes again, suggesting that no true difference may be observed here with respect to "Diet".

Appendix Table 50

Neutrophils: Minimum optimal sample size calculation

Effect	power	n.total	ncp	df1	df2
Diet					
Diet	0.8	232	7.915	1	229.662
Route					
Route	0.8	9	11.256	1	6.104
Time_point					
Time_point	0.8	6	17.344	1	3.132
Diet:Route					
Diet	0.8	665	7.872	1	660.599
Route	0.8	665	7.872	1	660.599
Diet:Route	0.8	665	7.872	1	660.599
Diet:Time_point					
Diet	0.8	98	8.013	1	93.677
Time_point	0.8	98	8.013	1	93.677
Diet:Time_point	0.8	98	8.013	1	93.677
Route:Time_point					
Route	0.8	10	11.518	1	5.78
Time_point	0.8	10	11.518	1	5.78
Route:Time_point	0.8	10	11.518	1	5.78
Diet:Route:Time_point					
Diet	0.8	347	7.894	1	338.278
Route	0.8	347	7.894	1	338.278
Time_point	0.8	347	7.894	1	338.278
Diet:Route	0.8	347	7.894	1	338.278

Diet:Time_point	0.8	347	7.894	1	338.278
Route:Time_point	0.8	347	7.894	1	338.278
Diet:Route:Time_point	0.8	347	7.894	1	338.278

For Monocytes counts, the same observations were true as for Myeloid_cells counts with the exception that "Diet" did not have a major detrimental effect on predicted minimum sample size for the three-way interaction ("Diet:Route:Time_point"; Appendix Table 51).

Appendix Table 51

Monocytes: Minimum optimal sample size calculation

Effect	power	n.total	ncp	df1	df2
Diet					
Diet	0.8	320	7.897	1	317.655
Route					
Route	0.8	11	10.197	1	8.134
Time_point					
Time_point	0.8	9	10.786	1	6.826
Diet:Route					
Diet	0.8	547	7.877	1	542.2
Route	0.8	547	7.877	1	542.2
Diet:Route	0.8	547	7.877	1	542.2
Diet:Time_point					
Diet	0.8	3455	7.853	1	3450.162
Time_point	0.8	3455	7.853	1	3450.162
Diet:Time_point	0.8	3455	7.853	1	3450.162
Route:Time_point					
Route	0.8	33	8.413	1	28.521
Time_point	0.8	33	8.413	1	28.521
Route:Time_point	0.8	33	8.413	1	28.521
Diet:Route:Time_point					
Diet	0.8	75	8.083	1	66.118
Route	0.8	75	8.083	1	66.118
Time_point	0.8	75	8.083	1	66.118
Diet:Route	0.8	75	8.083	1	66.118
Diet:Time_point	0.8	75	8.083	1	66.118

Route:Time_point	0.8	75	8.083	1	66.118
Diet:Route:Time_point	0.8	75	8.083	1	66.118

Retrospective calculation of statistical power in our data analysis

The observations from the retrospective minimum sample size calculation was reflected in the power calculation for our data. For Myeloid_cells counts, only "Route" and "Time_point" and the interaction of "Diet:Time_point" had sufficient statistical power to have a change to find statistically meaning differences in the data (Appendix Table 52).

Appendix Table 52

Myeloid Cells: Statistical power of data

Effect	power	n.total	ncp	df1	df2
Diet					
Diet	0.107	40	0.508	1	38
Route					
Route	1	40	73.967	1	38
Time_point					
Time_point	0.987	40	18.501	1	38
Diet:Route					
Diet	0.051	40	0.005	1	36
Route	0.051	40	0.005	1	36
Diet:Route	0.051	40	0.005	1	36
Diet:Time_point					
Diet	0.521	40	4.273	1	36
Time_point	0.521	40	4.273	1	36
Diet:Time_point	0.521	40	4.273	1	36
Route:Time_point					
Route	0.883	40	10.492	1	36
Time_point	0.883	40	10.492	1	36
Route:Time_point	0.883	40	10.492	1	36
Diet:Route:Time_point					
Diet	0.05	40	0.004	1	32
Route	0.05	40	0.004	1	32
Time_point	0.05	40	0.004	1	32

Diet:Route	0.05	40	0.004	1	32
Diet:Time_point	0.05	40	0.004	1	32
Route:Time_point	0.05	40	0.004	1	32
Diet:Route:Time_point	0.05	40	0.004	1	32

For Neutrophils counts, “Route” and “Time_points as much as their interaction had >80% statistical power, while the interaction”Diet:Time_point” was close to 80% statistical power (Appendix Table 53).

Appendix Table 53

Neutrophils: Statistical power of data

Effect	power	n.total	ncp	df1	df2
Diet					
Diet	0.207	40	1.367	1	38
Route					
Route	1	40	55.554	1	38
Time_point					
Time_point	1	40	135.188	1	38
Diet:Route					
Diet	0.103	40	0.474	1	36
Route	0.103	40	0.474	1	36
Diet:Route	0.103	40	0.474	1	36
Diet:Time_point					
Diet	0.422	40	3.281	1	36
Time_point	0.422	40	3.281	1	36
Diet:Time_point	0.422	40	3.281	1	36
Route:Time_point					
Route	1	40	47.107	1	36
Time_point	1	40	47.107	1	36
Route:Time_point	1	40	47.107	1	36
Diet:Route:Time_point					
Diet	0.153	40	0.912	1	32
Route	0.153	40	0.912	1	32
Time_point	0.153	40	0.912	1	32
Diet:Route	0.153	40	0.912	1	32
Diet:Time_point	0.153	40	0.912	1	32

Route:Time_point	0.153	40	0.912	1	32
Diet:Route:Time_point	0.153	40	0.912	1	32

Similar to Neutrophils counts, for Monocytes counts, "Route" and "Time_points as much as their interaction had >80% statistical power, while the interaction"Diet:Time_point" was close to 80% statistical power (Appendix Table 54).

Appendix Table 54

Monocytes: Statistical power of data

Effect	power	n.total	ncp	df1	df2
Diet					
Diet	0.163	40	0.988	1	38
Route					
Route	1	40	40.247	1	38
Time_point					
Time_point	1	40	48.886	1	38
Diet:Route					
Diet	0.115	40	0.577	1	36
Route	0.115	40	0.577	1	36
Diet:Route	0.115	40	0.577	1	36
Diet:Time_point					
Diet	0.06	40	0.091	1	36
Time_point	0.06	40	0.091	1	36
Diet:Time_point	0.06	40	0.091	1	36
Route:Time_point					
Route	0.879	40	10.347	1	36
Time_point	0.879	40	10.347	1	36
Route:Time_point	0.879	40	10.347	1	36
Diet:Route:Time_point					
Diet	0.526	40	4.362	1	32
Route	0.526	40	4.362	1	32
Time_point	0.526	40	4.362	1	32
Diet:Route	0.526	40	4.362	1	32
Diet:Time_point	0.526	40	4.362	1	32
Route:Time_point	0.526	40	4.362	1	32

Diet:Route:Time_point	0.526	40	4.362	1	32

Conclusion

In conclusion, it can be said that the infection route (needle, sand fly, or uninfected) as much as the time point of data collection (24 h vs. 72 h) were the primary difference makers with respect to observed IL-1β^{+} Myeloid_cells, Neutrophils and Monocytes counts in pooled mouse ear single-cell suspensions. It is of note that the nutritional status of the individuals did not have a detectable impact on the observed IL-1β^{+} cell counts in the site of infection.

Panel h

Here, we are presenting the relative fold difference in the heme oxygenase-1 (HO-1) protein levels in well-nourished (WN) and malnourished (MN) BALB/s mice infected with *Leishmania donovani* parasites via the sand fly route or uninfected. Heat-shock protein 90 (Hsp90) was used as a house-keeping gene control for the Western blot loading control and well-nourished control (WN Ctrl) mice served as a HO-1 concentration reference. Three Western blots were produced with different pooled samples as biological replicas. To normalize the band intensity readings, we first calculated the normalization factor by dividing the Hsp90 value from the WN Ctrl sample of one blot with the Hsp90 values of all other line on all blots. Then we multiplied the HO-1 readings by the normalized Hsp90 readings for each sample lane, respectively. Fold change differences were then calculated by dividing all normalized HO-1 readings with the normalized WN Ctrl HO-1 reading for each Western blot, respectively. This resulted in all WN Ctrl HO-1 readings to be set to 1. As this eliminated any data variance in WN Ctrl group, it was treated as the baseline reference and thus, was disconsidered from the statistical analysis.

The remaining three groups, well-nourished sand fly infected (WN SF), malnourished control (MN Ctrl) and malnourished sand fly infected (MN SF) were analyzed by the Kruskal-Wallis test, were analyzed by Kruskal-Wallis test followed post hoc by the Dunn's test for pairwise comparison. The output showed a p-value of 0.148, which was not statistically significant. The pairwise comparison by Dunn's test confirmed that no statistically significant differences were observed between WN SF, MN Ctrl and MN SF .

Appendix Table 55

Dunn's test

group1	group2	n1	n2	statistic	p	p.adj	p.adj.signif
MN_CTRL	MN_SF	3	3	1.9379	0.0526	0.1579	ns
MN_CTRL	WN_SF	3	3	0.7454	0.4561	0.4661	ns
MN_SF	WN_SF	3	3	-1.1926	0.2330	0.4661	ns

It is of not that the median fold difference of the MN Ctrl group was 2.766 higher than that of the WN Ctrl group, showing that more HO-1 was present in malnourished mice prior to infection, but that did not seem

to have a profound impact on the median HO-1 fold differences compared to the WN Ctrl reference post infection by sand fly for malnourished compared to well-nourished mice (WN SF: 6.379, MN SF: 8.088).

Figure 2

Panel a

Data analysis

We analysed the frequency of *Leishmania donovani* dissemination to the draining lymph node in a total of N=32 well-nourished (WN) and malnourished (MN) BALB/c mice infected intradermally either by "needle" injection or sand fly bite (SF) (N: MN_Needle=8, MN_SF=8, WN_Needle=8, WN_SF=8) by contingency table analysis and logistic regression.

Contingency table

Due to the small sample sizes, there were several expected counts <5, why we opted for the Fisher's Exact test, which had the added benefit of exact p-value calculation. The analysis rendered a p-value of 0.444, suggesting no statistically significant difference between groups. This was confirmed by the pairwise Fisher's Exact test corrected by the Benjamin-Hochberg method (Appendix table 56).

Appendix Table 56

Pairwise Fisher's Exact test

group1	group2	n	estimate	p	conf.low	conf.high	alternative	p.adj	p.adj.signif
MN_Needle	MN_SF	16	Inf	1.000	0.0256	Inf	two.sided	1	ns
MN_Needle	WN_Needle	16	0.2605	0.569	0.0040	4.4010	two.sided	1	ns
MN_Needle	WN_SF	16	0.4516	1.000	0.0064	10.7913	two.sided	1	ns
MN_SF	WN_Needle	16	0.0000	0.200	0.0000	2.2053	two.sided	1	ns
MN_SF	WN_SF	16	0.0000	0.467	0.0000	5.2059	two.sided	1	ns
WN_Needle	WN_SF	16	1.7346	1.000	0.1359	28.9942	two.sided	1	ns

We observed a 3.84-fold and a 2.21-fold reduction in parasite dissemination events in well-nourished animals infected by needle and sand fly, respectively, compared to malnourished, needle inoculated ones, but the 95% confidence intervals were so large that 1 was included, suggesting that this decreased occurrence in dissemination was not statistically significant (Appendix table 57). However, applying a retrospective statistical power calculation showed that the sample size was to small to detect a meaningful difference here and thus, our statistical power was well below the standard 80% (Appendix table 58), but larger sample sizes were prohibitive due to cost and loss of life.

Appendix Table 57

Odds Ratios

Groups	estimate	lower	upper	p.value
MN_Needle	1.0000	NA	NA	NA
MN_SF	Inf	0.0256	Inf	1.0000
WN_Needle	0.2605	0.0040	4.4010	0.5692
WN_SF	0.4516	0.0064	10.7913	1.0000

Appendix Table 58

Retrospective Power Calculation

	Calculation for	
Parameters	Sample size	Statistical power
Statistical power	0.8	**0.367**
Total n	**86**	32
Degrees of freedom	3	3
Non-centrality parameter	10.903	4.103
Type I error rate	0.05	0.05
Type II error rate	0.2	0.633

Logistic regression

We applied a logistic regression model to the same data and assessed the two predictor variables "Diet" and "Route" without an interaction term to assess individual predictor contribution to the outcome. The data output showed that infection route did not have much impact on whether parasites made it to the draining lymph nodes or not (p=0.3472), suggesting a mere 2.57-fold increase in probability of parasite dissemination when sand flies were used (Appendix table 59), which was equivalent to a small effect size (Appendix table 60). Conversely, although not reaching statistical significance either, there was an indication in the data, that "Diet" affects parasites capacity to disseminate to the draining lymph nodes as the p-value approached statistical significance (p=0.0949), indicating a 7.19-fold increase in the probability of parasite dissemination (Appendix table 59), which was equivalent to a large effect size (Appendix table 60). Even so, neither predictor achieved statistical significance according to Wald test (Appendix table 61).

Appendix Table 59

Logistic regression output

Groups	Estimate	lower CI	upper CI	Std. Error	partial.R2	z value	Pr(>|z|)	sig.
(Intercept)	0.3582	-0.9967	1.7994	0.6888	0.0000	0.5200	0.6030	ns

DietMN	1.9727	-0.0533	5.0210	1.1813	0.1206	1.6699	0.0949	+
RouteSF	0.9452	-0.9604	3.1423	1.0055	0.0340	0.9400	0.3472	ns

Appendix Table 60

Odds Ratios

Predictor	OR	2.5 %	97.5 %	Effect_size
(Intercept)	1.4307	0.3691	6.0459	very small
DietMN	7.1900	0.9481	151.5553	large
RouteSF	2.5733	0.3827	23.1574	small

Appendix Table 61

Wald test

Predictor	chi2	df	P
Diet	2.79	1	0.0949
Route	0.88	1	0.3472

However, although none of the predictors was statistically significant in the logistic regression model, thus did not mean that they had no meaningful biological effect. A retrospective sample size and power calculation with the study data showed that the study was well underpowered for the logistic regression, as it was already for the contingency table analysis (Appendix table 62). The proposed minimum total sample size that would have given both predictor a chance to identify a meaningful statistical different by this calculation was 382, which was ~12-times of the study's sample size, which was prohibitive due to cost and excessive loss of life. Even so, there was a good indication of potential biological significance with respect to nutritional status. Considering the predicted probability of parasite dissemination, it can be seen that being malnourished increased the probability of parasite dissemination (Appendix table 63). The large confidence intervals, however, did not render statistical significance, which does not exclude biological significance. Even the route of infection showed at least in the well-nourished model a considerable increase in predicted probability of parasite dissemination from needle to sand fly inoculation. Thus, the lack of statistical power and high data variance prevented the obtainment of statistical significance.

Appendix Table 62

Retrospective power analyses

Calculation	Predictor	Beta0	Beta1	R-square	alpha	Power	TotalN	NCP	Alternative
Sample_size	Diet	1.466	1.973	0.034	0.05	0.80	**144**	2.606	not equal
Sample_size	Route	1.466	0.945	0.121	0.05	0.80	**382**	2.752	not equal
Power	Diet	0.358	1.973	0.034	0.05	**0.48**	32	1.908	not equal

Power	Route	0.358	0.945	0.121	0.05	**0.19**	32	1.117	not equal

Appendix Table 63

Predicted probability of parasite dissemination

Diet	Route	fit	se.fit	Predicted_Probability	lower_CI	upper_CI
WN	Needle	0.3582	0.6888	**0.5886**	0.2706	0.8466
WN	SF	1.3034	0.8107	**0.7864**	0.4291	0.9475
MN	Needle	2.3309	1.0825	**0.9114**	0.5521	0.9885
MN	SF	3.2761	1.2532	**0.9636**	0.6942	0.9968

Panel b

Data analysis

We analyzed the frequency of *Leishmania donovani* dissemination to the spleen in a total of N=92 well-nourished (WN) and malnourished (MN) BALB/c mice infected intradermally either by "needle" injection or sand fly bite (SF) (N: MN_Needle=23, MN_SF=23, WN_Needle=23, WN_SF=23) by contingency table analysis and logistic regression.

Contingency table

Here, we opted for the Chi-square test as assumptions held. The analysis rendered a p-value of 0.0742, suggesting no statistically significant difference between groups. This was confirmed by the pairwise Chi-square test corrected by the Benjamin-Hochberg method (Appendix table 64).

Appendix Table 64

Pairwise Chi-square test

n	group1	group2	statistic	p	df	p.adj	p.adj.signif
46	MN_Needle	MN_SF	3.7829	0.0518	1	0.155	ns
46	MN_Needle	WN_Needle	0.0000	1.0000	1	1.000	ns
46	MN_Needle	WN_SF	0.0000	1.0000	1	1.000	ns
46	MN_SF	WN_Needle	4.9730	0.0257	1	0.154	ns
46	MN_SF	WN_SF	2.6960	0.1010	1	0.202	ns
46	WN_Needle	WN_SF	0.1027	0.7490	1	1.000	ns

However, while we observed a 1.21-fold reduction in parasite dissemination events in well-nourished animals infected by needle, compared to malnourished, needle inoculated ones, we also observed an increase in parasite dissemination in both, malnourished and well-nourished mice, infected by sand fly bite, but only the malnourished group had significantly higher odds of dissemination (Appendix table 65).

Applying a retrospective statistical power calculation showed that the sample size was to small to detect a meaningful statistical difference here and thus, our statistical power was somewhat below the standard 80% (Appendix table 66), but larger sample sizes were prohibitive due to cost and loss of life.

Appendix Table 65

Odds Ratios

Groups	estimate	lower	upper	p.value
MN_Needle	1.0000	NA	NA	NA
MN_SF	8.3203	1.2566	227.4435	0.047
WN_Needle	0.8255	0.2294	2.9144	1.000
WN_SF	1.2305	0.3296	4.7180	1.000

Appendix Table 66

Retrospective Power Calculation

	Calculation for	
Parameters	Sample size	Statistical power
Statistical power	0.8	**0.585**
Total n	**145**	92
Degrees of freedom	3	3
Non-centrality parameter	10.903	6.93
Type I error rate	0.05	0.05
Type II error rate	0.2	0.415

Logistic regression

We applied a logistic regression model to the same data and assessed the two predictor variables “Diet” and “Route” without an interaction term to assess individual predictor contribution to the outcome. The data output showed that infection route had much more impact on whether parasites made it to the spleen or not (p=0.0524) than to the draining lymph nodes (Fig.2a). However, we observed a mere 2.76-fold increase in probability of parasite dissemination when sand flies were used for infection (Appendix table 67), which was equivalent to a small effect size (Appendix table 68). There was also little indication in the data, that “Diet” on its own affected parasite capacity to disseminate to the spleen, indicating a mere 2.15-fold increase in the probability of parasite dissemination (Appendix table 67), which was equivalent to a small effect size (Appendix table 68). Thus, neither predictor on its own achieved statistical significance according to Wald test (Appendix table 69). However, re-running the logistic regression model with an interaction term showed that the interaction between “Diet” and “Route” had much more potency than either predictor on its own, already hinted at by the odds ratios from the chi-square analysis, even though, the interaction term did not achieve statistical significance (Appendix table 70).

Appendix Table 67

Logistic regression output

Groups	Estimate	lower CI	upper CI	Std. Error	partial.R2	z value	Pr(>|z|)	sig.
(Intercept)	0.3695	-0.3890	1.1548	0.3897	0.0000	0.9482	0.3430	ns
DietMN	0.7640	-0.2277	1.8143	0.5156	0.0233	1.4817	0.1384	ns
RouteSF	1.0166	0.0186	2.0970	0.5241	0.0403	1.9396	0.0524	+

Appendix Table 68

Odds Ratios

Predictor	OR	2.5 %	97.5 %	Effect_size
(Intercept)	1.4470	0.6778	3.1733	very small
DietMN	2.1470	0.7964	6.1365	small
RouteSF	2.7638	1.0188	8.1414	small

Appendix Table 69

Wald test

Predictor	chi2	df	P
Diet	2.20	1	0.1384
Route	3.76	1	0.0524

Appendix Table 70

Logistic regression with interaction output

Groups	Estimate	Std. Error	z value	Pr(>|z|)
(Intercept)	0.6286	0.4378	1.4358	0.1510
DietMN	0.1981	0.6301	0.3143	0.7533
RouteSF	0.4128	0.6459	0.6392	0.5227
DietMN:RouteSF	1.8515	1.2914	1.4338	0.1516

A retrospective sample size and power calculation with the study data showed that the study was well underpowered for the logistic regression, as it was already for the contingency table analysis (Appendix table 71). The proposed minimum total sample size that permit both predictor a chance to identify a meaningful statistical different by this calculation was 397, which was ~4-times of the study's sample size, which was prohibitive due to cost and excessive loss of life. Even so, there was a good indication of potential biological significance with respect to interaction between "Diet" and "Route" of infection. Considering the predicted probability of parasite dissemination, it can be seen that parasite transmission

by sand fly bite increased the predicted probability of parasite dissemination from needle inoculation for either nutritional status, respectively, which significant for well-nourished mice as can be seen from the confidence intervals (Appendix table 72). "Diet" on its own had a bigger impact on parasite dissemination for needle inoculation. Thus, there are good indications here that parasite transmission by sand fly had a meaningful biological effect on the probability of parasite dissemination to the spleen, too, which was aided by nutritional status more so for the needle inoculation than for the sand fly transmission.

Appendix Table 71

Retrospective power analyses

Calculation	Predictor	Beta0	Beta1	R-square	alpha	Power	TotalN	NCP	Alternative
Sample_size	Diet	1.157	0.764	0.040	0.05	0.80	**397**	2.773	not equal
Sample_size	Route	1.157	1.017	0.023	0.05	0.80	**246**	2.751	not equal
Power	Diet	0.369	0.764	0.040	0.05	**0.37**	92	1.641	not equal
Power	Route	0.369	1.017	0.023	0.05	**0.56**	92	2.114	not equal

Appendix Table 72

Predicted probability of parasite dissemination

Diet	Route	fit	se.fit	Predicted_Probability	lower_CI	upper_CI
WN	Needle	0.3695	0.3897	**0.5913**	0.4027	0.7564
WN	SF	1.3861	0.4557	**0.8000**	0.6208	0.9071
MN	Needle	1.1335	0.4333	**0.7565**	0.5706	0.8790
MN	SF	2.1502	0.5268	**0.8957**	0.7535	0.9602

Panel c

Here, we present the parasite counts per isolated draining lymph node according to qPCR as a measure of parasite dissemination to the organ. To analyze this data, we had to re-scale it, due to the occurrence of frequent zero-values in instances of no detection, by dividing all value by the smallest non-zero value in the dataset. This resulted in a approximate Poisson / negative binomial distribution, which allowed the convenient analysis of the re-scaled and rounded counts by the appropriate models for these distributions.

We analyzed a total of N=32 BALB/c mice (WN_Needle=8, WN_SF=8, MN_Needle=8, MN_SF=8). These were the same mice as analyzed in figure 2a for parasite dissemination events. Here, we quantified parasite burden per isolated draining lymph node. For the data analysis we tested several Poisson and negative binomial-type regression models. Based on the Akaike information criterion (AIC) we selected a standard negative_binomial regression model for the data analysis post data re-scaling. The model fitted the data well producing no statistically significant departure from 1 for its dispersion ratio (dispersion_ratio: 0.9701, p_value: 0.632) and showing a reasonable pseudo-R^2 (Nagelkerke (Cragg and Uhler): 0.412452). The model output showed that both, "Diet" and "Route" were statistically significant predictors, but there was no statistically significant interaction between these two predictors (Appendix table 73).

Appendix Table 73

Negative binomial regression model output

Predictors	Estimate	Std. Error	z value	Pr(>|z|)	sig.
(Intercept)	2.7568	0.5315	5.1868	<0.0001	****
DietWN	-1.7918	0.7776	-2.3041	0.0212	*
RouteSF	2.0715	0.7470	2.7729	0.0056	**
DietWN:RouteSF	0.5977	1.0762	0.5554	0.5786	ns

The pairwise comparison based on the estimated marginal means showed that well-nourished needle inoculated BALB/c mice had statistically significantly less parasites in the draining lymph nodes than all other groups (Appendix table 74) and clearly clustered on its own (Appendix table 75). On the other hand, malnourished needle inoculated and well-nourished sand fly transmitted infection were comparable in their degree of parasite dissemination. Also, malnourished and well-nourished sand fly transmitted infection clustered together. Together, this data suggested that sand fly transmitted infections had a statistically significantly higher degree of parasite dissemination for each nutritional state, while malnourishment also exacerbated parasite dissemination. Ultimately, the effects of both variables seemed additive, but the regression model did not support this hypothesis, suggesting that either effect acted independently of one another.

Appendix Table 74

Pairwise comparison based on estimated marginal means

Predictor pairs	estimate	SE	df	z.ratio	p.value	sig.
MN Needle - WN Needle	1.7918	0.7776	Inf	2.3041	0.0318	*
MN Needle - MN SF	-2.0715	0.7470	Inf	-2.7729	0.0111	*
WN Needle - WN SF	-2.6692	0.7746	Inf	-3.4458	0.0017	**
MN SF - WN SF	1.1940	0.7439	Inf	1.6050	0.1302	ns

Appendix Table 75

Pairwise comparison letter code

Diet	Route	emmean	SE	df	asymp.LCL	asymp.UCL	.group
WN	Needle	0.9651	0.5676	Inf	-0.4527	2.3828	a
MN	Needle	2.7568	0.5315	Inf	1.4293	4.0844	b
WN	SF	3.6343	0.5271	Inf	2.3177	4.9509	bc
MN	SF	4.8283	0.5249	Inf	3.5171	6.1395	c

Panel d

Here, we present the parasite counts per isolated spleen according to qPCR as a measure of parasite dissemination to the organ. To analyze this data, we had to re-scale it, due to the occurrence of frequent zero-values in instances of no detection, by dividing all value by the smallest non-zero value in the dataset. This resulted in a approximate Poisson / negative binomial distribution, which allowed the convenient analysis of the re-scaled and rounded counts by the appropriate models for these distributions.

We analyzed a total of N=92 BALB/c mice (WN_Needle=23, WN_SF=23, MN_Needle=23, MN_SF=23). These were the same mice as analyzed in figure 2b for parasite dissemination events. Here, we quantified parasite burden per isolated spleen. For the data analysis, we tested several Poisson and negative binomial-type regression models. Based on the Akaike information criterion (AIC), we selected a standard negative_binomial regression model for the data analysis. The model fit of the data was moderate, producing no statistically significant departure from 1 for its dispersion ratio (dispersion_ratio: 1.3039, p_value: 0.392), but producing only a pseudo-R^2 of 0.178589 (Nagelkerke (Cragg and Uhler)). The model output showed that "Diet" was the only statistically significant predictors, with "Route" and the interaction term producing no statistically significant result (Appendix table 76).

Appendix Table 76

Negative binomial regression model output

Predictors	Estimate	Std. Error	z value	Pr(>|z|)	sig.
(Intercept)	2.9095	0.3223	9.0285	<0.0001	****
DietWN	-0.9391	0.4598	-2.0424	0.0411	*
RouteSF	0.3943	0.4549	0.8669	0.3860	ns
DietWN:RouteSF	-1.0005	0.6535	-1.5310	0.1258	ns

The pairwise comparison based on the estimated marginal means showed that different nutritional statuses generally produced statistical significance, with the exception of malnourished and well-nourished needle inoculation, which was close to the significance threshold, though (Appendix table 77). In particular, well-nourished sand fly transmitted infection clustered away from malnourished mice, regardless of infection route (Appendix table 78). Conversely, parasite dissemination in sand fly transmitted infections in malnourished BALB/c mice were clearly more efficient than in a well-nourished setting. Contrary to the draining lymph nodes, parasite dissemination to the spleen was clearly determined more by the animals nutritional state, whether than infection route.

Appendix Table 77

Pairwise comparison based on estimated marginal means

Predictor pairs	estimate	SE	df	z.ratio	p.value	sig.
MN Needle - WN Needle	0.9391	0.4598	Inf	2.0424	0.0617	+
MN Needle - MN SF	-0.3943	0.4549	Inf	-0.8669	0.3860	ns

WN Needle - WN SF	0.6061	0.4692	Inf	1.2919	0.2357	ns
MN SF - WN SF	1.9395	0.4644	Inf	4.1764	0.0002	***

Appendix Table 78

Pairwise comparison letter code

Diet	Route	emmean	SE	df	asymp.LCL	asymp.UCL	.group
WN	SF	1.3643	0.3355	Inf	0.5262	2.2024	a
WN	Needle	1.9705	0.3279	Inf	1.1514	2.7895	ab
MN	Needle	2.9095	0.3223	Inf	2.1046	3.7144	bc
MN	SF	3.3039	0.3211	Inf	2.5019	4.1058	c

Panel e

Here, we investigated the lymph node barrier function in well-nourished and malnourished BALB/c mice infected with *Leishmania donovani* parasite via sand fly bites. The accumulation of intradermally injected 10,000 kDa-Dextran in the draining lymph nodes 72 h post sand fly bite were analyzed by Flow cytometry. We analyzed a total of N=19 BALB/c mice (MN_SF=9, WN_SF=10). For the data analysis, we tested Poisson and negative binomial regression models of the normalized cell counts, or beta regression after conversion of percentiles to ratios. Based on the Akaike information criterion (AIC), we selected a beta_regression model for the data analysis post data conversion to ratios. The model fit of the data was reasonable producing no statistically significant departure from 1 for its dispersion ratio (0.9284), but producing only a pseudo-R^2 of 0.1987. The model output showed that "Diet" was a statistically significant predictors (Appendix table 79) and its inclusion made the model distinct from the null model (Appendix table 80), showing that statistically significantly more Dextran was retained in draining lymph nodes from well-nourished BALB/c mice.

Appendix Table 79

Beta regression model output

Predictors	Estimate	Std. Error	z value	Pr(>|z|)	sig.
(Intercept)	-1.2997	0.2663	-4.8806	<0.0001	****
DietWN	0.7628	0.3418	2.2315	0.0256	*

Appendix Table 80

Predictor significance in the model

Model	#Df	LogLik	Df	Chisq	Pr(>Chisq)	sig.
Null	3	10.1577	NA	NA	NA	?

Diet	2	7.8903	-1	4.5349	0.0332	*

As the only predictor variable was dichotomous, there was strictly no need for a pairwise comparison. But we performed one anyway to ensure that the approach via estimated marginal means was comparable to the model output. The pairwise comparison based on the estimated marginal means showed that different nutritional statuses produced statistical significance comparable to the model output (Appendix table 81 & Appendix table 82). This supported the hypothesis that malnourishment resulted in a breakdown of the lymph node barrier, which could explained the increased parasite dissemination to the spleen in malnourished mice.

Appendix Table 81

Pairwise comparison based on estimated marginal means

Predictor pairs	estimate	SE	df	z.ratio	p.value	sig.
MN - WN	-0.1547	0.0674	Inf	-2.2944	0.0218	*

Appendix Table 82

Pairwise comparison letter code

Diet	emmean	SE	df	asymp.LCL	asymp.UCL	.group
MN	0.2142	0.0448	Inf	0.1137	0.3147	a
WN	0.3689	0.0516	Inf	0.2534	0.4845	b

Panel f

Whereas figure 2e looked at the retention of Dextran in draining lymph nodes, here, we investigated the accumulation of intradermally injected 10,000 kDa-Dextran in the spleen 72 h post sand fly bite, which required transition through the draining lymph node. The samples were also analyzed by Flow cytometry. We analyzed a total of N=19 BALB/c mice (MN_SF=9, WN_SF=10). This were the same mice as in figure 2e. For the data analysis, we tested Poisson and negative binomial regression models of the normalized cell counts, or beta regression after conversion of percentiles to ratios. Based on the Akaike information criterion (AIC), we selected a beta_regression model for the data analysis post data re-scaling. The model fit of the data was reasonable producing no statistically significant departure from 1 for its dispersion ratio (1.119), producing a pseudo-R^2 of 0.893. The model output showed that “Diet” was a statistically significant predictors (Appendix table 83) and its inclusion made the model distinct from the null model (Appendix table 84), showing that statistically significantly more Dextran accumulated in spleens from malnourished BALB/c mice.

Appendix Table 83

Beta regression model output

Predictors	Estimate	Std. Error	z value	Pr(>|z|)	sig.
(Intercept)	-3.2827	0.0485	-67.7093	<0.0001	****
DietWN	-0.9373	0.0862	-10.8747	<0.0001	****

Appendix Table 84

Predictor significance in the model

Model	#Df	LogLik	Df	Chisq	Pr(>Chisq)	sig.
Null	3	78.0812	NA	NA	NA	?
Diet	2	58.7547	-1	38.6529	<0.0001	****

As the only predictor variable was dichotomous, there was strictly no need for a pairwise comparison. But we performed one anyway to ensure that the approach via estimated marginal means was comparable to the model output. The pairwise comparison based on the estimated marginal means showed that different nutritional statuses produced statistical significance comparable to the model output (Appendix table 85 & Appendix table 86). In agreement with the data from figure 2e, this data further supported the hypothesis that malnourishment resulted in a breakdown of the lymph node barrier, which could explained the increased parasite dissemination to the spleen in malnourished mice observed in figures 2c-d.

Appendix Table 85

Pairwise comparison based on estimated marginal means

Predictor pairs	estimate	SE	df	z.ratio	p.value	sig.
MN - WN	0.0217	0.002	Inf	11.0138	<0.0001	****

Appendix Table 86

Pairwise comparison letter code

Diet	emmean	SE	df	asymp.LCL	asymp.UCL	.group
WN	0.0145	0.0010	Inf	0.0122	0.0168	a
MN	0.0362	0.0017	Inf	0.0324	0.0400	b

Figure 3

Panel a

Data analysis

In figure 3a, we present the longitudinal weekly observation of mouse body weight pre and post *Leishmania donovani* infection, either by needle inoculation (needle), sand fly transmission (SF) or not at all (Naive). Information of a total of N=45 BALB/c mice (WN_Control: 8, MN_Control: 8, WN_Needle: 7, MN_Needle: 7, WN_SF: 8, MN_SF: 7) over the course of 22 weeks are shown here; "Week_0" being the weight before shipment, "Week_6" being the first week post-infection, and "Week_22" being the final week before the termination of the experiment.

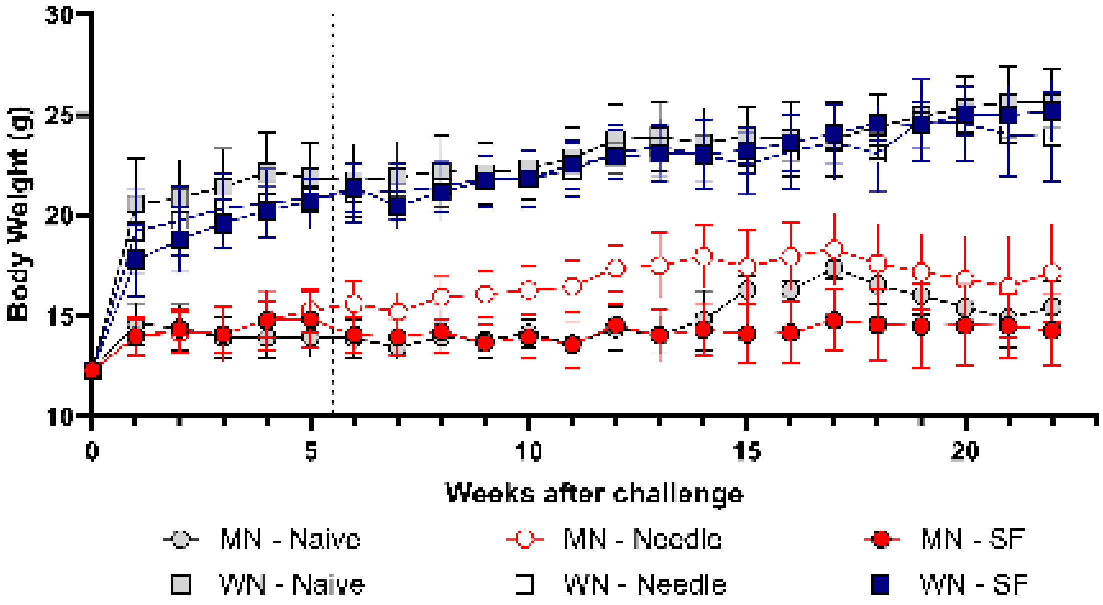

Fig.3a: This an extended version of the main figure 3a from the publication showing as well the pre-infection time-points. The dotted line marked the point of mouse infection.

We would need to analyze the data with a three-way mixed approach that included repeated measures to account for the three predictors; "Time_point" being the within-subject factor, while "Diet" and "Route" were the between-subject factors in the analysis with "Weight_g" being the dependent outcome variable.

For a three-way mixed ANOVA, we had to assess the data for compliance with assumptions:

- Data normality
- Homogeneity of variance

- Homogeneity of Covariance
- No significant outliers
- Assumption of sphericity

Initial assumption assessment indicated that the Gaussian distribution assumption was not met along with the occurrence of several extreme outliers. Data transformation by Box-Cox power transformation reduced the magnitude of violation, although it did not completely remove it. Either way, we present the analysis of the assumption assessment with the transformed data below. Thus, data distribution and variance appear different in the main figure in the publication from the once that were used in the analysis post transformation.

Assumption analyses

Data normality

The assessment of the Box-Cox power transformed data distribution for each group was conducted by Shapiro-Wilks test and QQ-plot after splitting the data by all three predictors. Note that all groups consisted of *N*=WN_Control: 8, MN_Control: 8, WN_Needle: 7, MN_Needle: 7, WN_SF: 8, MN_SF: 7 individuals, which made groups too small to assess data distribution reliably by Shapiro-Wilks test. In spite of this, we performed the analyses by Shapiro-Wilks test (Appendix table 87) and QQ-pots (Fig.3a-1) and found deviations from normality.

Appendix Table 87

Univariate Shapito-Wilks test results

Diet	Route	Time_point	variable	statistic	p	Outcome
Pre-Infection						
WN	Control	Week_0	Counts	0.7729	0.0146	sig.
MN	Control	Week_0	Counts	0.9118	0.3671	ns
WN	Needle	Week_0	Counts	0.6644	0.0015	sig.
MN	Needle	Week_0	Counts	0.8181	0.0615	ns
WN	SF	Week_0	Counts	0.7823	0.0184	sig.
MN	SF	Week_0	Counts	0.8333	0.0860	ns
WN	Control	Week_1	Counts	0.8468	0.0884	ns
MN	Control	Week_1	Counts	0.9006	0.2923	ns
WN	Needle	Week_1	Counts	0.9335	0.5814	ns
MN	Needle	Week_1	Counts	0.9593	0.8128	ns
WN	SF	Week_1	Counts	0.8352	0.0673	ns
MN	SF	Week_1	Counts	0.8678	0.1774	ns
WN	Control	Week_2	Counts	0.8406	0.0764	ns
MN	Control	Week_2	Counts	0.8894	0.2309	ns
WN	Needle	Week_2	Counts	0.9363	0.6056	ns

MN	Needle	Week_2	Counts	0.8935	0.2932	ns
WN	SF	Week_2	Counts	0.8253	0.0531	ns
MN	SF	Week_2	Counts	0.9515	0.7437	ns
WN	Control	Week_3	Counts	0.8942	0.2558	ns
MN	Control	Week_3	Counts	0.9273	0.4919	ns
WN	Needle	Week_3	Counts	0.8753	0.2065	ns
MN	Needle	Week_3	Counts	0.8073	0.0483	sig.
WN	SF	Week_3	Counts	0.8960	0.2656	ns
MN	SF	Week_3	Counts	0.9739	0.9249	ns
WN	Control	Week_4	Counts	0.9525	0.7363	ns
MN	Control	Week_4	Counts	0.9198	0.4281	ns
WN	Needle	Week_4	Counts	0.9146	0.4285	ns
MN	Needle	Week_4	Counts	0.8741	0.2016	ns
WN	SF	Week_4	Counts	0.9291	0.5079	ns
MN	SF	Week_4	Counts	0.9746	0.9292	ns
WN	Control	Week_5	Counts	0.9659	0.8639	ns
MN	Control	Week_5	Counts	0.8773	0.1773	ns
WN	Needle	Week_5	Counts	0.9319	0.5674	ns
MN	Needle	Week_5	Counts	0.8583	0.1463	ns
WN	SF	Week_5	Counts	0.9314	0.5289	ns
MN	SF	Week_5	Counts	0.9633	0.8463	ns
Post-Infestion						
WN	Control	Week_6	Counts	0.9440	0.6508	ns
MN	Control	Week_6	Counts	0.9299	0.5147	ns
WN	Needle	Week_6	Counts	0.9699	0.8976	ns
MN	Needle	Week_6	Counts	0.8327	0.0848	ns
WN	SF	Week_6	Counts	0.9879	0.9911	ns
MN	SF	Week_6	Counts	0.9779	0.9487	ns
WN	Control	Week_7	Counts	0.9637	0.8444	ns
MN	Control	Week_7	Counts	0.9139	0.3825	ns
WN	Needle	Week_7	Counts	0.9147	0.4296	ns
MN	Needle	Week_7	Counts	0.9199	0.4688	ns
WN	SF	Week_7	Counts	0.9801	0.9632	ns
MN	SF	Week_7	Counts	0.9295	0.5467	ns
WN	Control	Week_8	Counts	0.9699	0.8969	ns
MN	Control	Week_8	Counts	0.9504	0.7148	ns
WN	Needle	Week_8	Counts	0.9015	0.3402	ns
MN	Needle	Week_8	Counts	0.8640	0.1643	ns
WN	SF	Week_8	Counts	0.9431	0.6420	ns
MN	SF	Week_8	Counts	0.8841	0.2453	ns

WN	Control	Week_9	Counts	0.9563	0.7743	ns
MN	Control	Week_9	Counts	0.9378	0.5895	ns
WN	Needle	Week_9	Counts	0.8770	0.2135	ns
MN	Needle	Week_9	Counts	0.9078	0.3810	ns
WN	SF	Week_9	Counts	0.9499	0.7105	ns
MN	SF	Week_9	Counts	0.9522	0.7493	ns
WN	Control	Week_10	Counts	0.9366	0.5777	ns
MN	Control	Week_10	Counts	0.9605	0.8145	ns
WN	Needle	Week_10	Counts	0.9026	0.3472	ns
MN	Needle	Week_10	Counts	0.9179	0.4533	ns
WN	SF	Week_10	Counts	0.9133	0.3782	ns
MN	SF	Week_10	Counts	0.9534	0.7606	ns
WN	Control	Week_11	Counts	0.9437	0.6478	ns
MN	Control	Week_11	Counts	0.9641	0.8477	ns
WN	Needle	Week_11	Counts	0.9074	0.3782	ns
MN	Needle	Week_11	Counts	0.8722	0.1940	ns
WN	SF	Week_11	Counts	0.9294	0.5107	ns
MN	SF	Week_11	Counts	0.9553	0.7771	ns
WN	Control	Week_12	Counts	0.9587	0.7981	ns
MN	Control	Week_12	Counts	0.9428	0.6389	ns
WN	Needle	Week_12	Counts	0.9434	0.6696	ns
MN	Needle	Week_12	Counts	0.9293	0.5452	ns
WN	SF	Week_12	Counts	0.9507	0.7186	ns
MN	SF	Week_12	Counts	0.9283	0.5365	ns
WN	Control	Week_13	Counts	0.9768	0.9453	ns
MN	Control	Week_13	Counts	0.9050	0.3205	ns
WN	Needle	Week_13	Counts	0.9768	0.9426	ns
MN	Needle	Week_13	Counts	0.9225	0.4887	ns
WN	SF	Week_13	Counts	0.9049	0.3195	ns
MN	SF	Week_13	Counts	0.9002	0.3323	ns
WN	Control	Week_14	Counts	0.9521	0.7323	ns
MN	Control	Week_14	Counts	0.8924	0.2464	ns
WN	Needle	Week_14	Counts	0.9310	0.5592	ns
MN	Needle	Week_14	Counts	0.9140	0.4244	ns
WN	SF	Week_14	Counts	0.8788	0.1834	ns
MN	SF	Week_14	Counts	0.9498	0.7279	ns
WN	Control	Week_15	Counts	0.9124	0.3715	ns
MN	Control	Week_15	Counts	0.8167	0.0431	sig.
WN	Needle	Week_15	Counts	0.7351	0.0088	sig.
MN	Needle	Week_15	Counts	0.9505	0.7338	ns
WN	SF	Week_15	Counts	0.9126	0.3729	ns

MN	SF	Week_15	Counts	0.9603	0.8210	ns
WN	Control	Week_16	Counts	0.8604	0.1212	ns
MN	Control	Week_16	Counts	0.8289	0.0578	ns
WN	Needle	Week_16	Counts	0.7123	0.0050	sig.
MN	Needle	Week_16	Counts	0.9455	0.6885	ns
WN	SF	Week_16	Counts	0.8795	0.1861	ns
MN	SF	Week_16	Counts	0.9444	0.6783	ns
WN	Control	Week_17	Counts	0.8210	0.0478	sig.
MN	Control	Week_17	Counts	0.8694	0.1487	ns
WN	Needle	Week_17	Counts	0.8980	0.3190	ns
MN	Needle	Week_17	Counts	0.9786	0.9522	ns
WN	SF	Week_17	Counts	0.8581	0.1149	ns
MN	SF	Week_17	Counts	0.9189	0.4612	ns
WN	Control	Week_18	Counts	0.8087	0.0354	sig.
MN	Control	Week_18	Counts	0.9667	0.8713	ns
WN	Needle	Week_18	Counts	0.8892	0.2706	ns
MN	Needle	Week_18	Counts	0.9528	0.7550	ns
WN	SF	Week_18	Counts	0.8360	0.0686	ns
MN	SF	Week_18	Counts	0.9453	0.6869	ns
WN	Control	Week_19	Counts	0.8965	0.2689	ns
MN	Control	Week_19	Counts	0.9282	0.4996	ns
WN	Needle	Week_19	Counts	0.6529	0.0011	sig.
MN	Needle	Week_19	Counts	0.9179	0.4532	ns
WN	SF	Week_19	Counts	0.8780	0.1801	ns
MN	SF	Week_19	Counts	0.9285	0.5383	ns
WN	Control	Week_20	Counts	0.8508	0.0971	ns
MN	Control	Week_20	Counts	0.9540	0.7517	ns
WN	Needle	Week_20	Counts	0.7695	0.0202	sig.
MN	Needle	Week_20	Counts	0.9215	0.4808	ns
WN	SF	Week_20	Counts	0.8922	0.2453	ns
MN	SF	Week_20	Counts	0.9223	0.4875	ns
WN	Control	Week_21	Counts	0.9517	0.7285	ns
MN	Control	Week_21	Counts	0.9221	0.4467	ns
WN	Needle	Week_21	Counts	0.8350	0.0893	ns
MN	Needle	Week_21	Counts	0.9039	0.3553	ns
WN	SF	Week_21	Counts	0.8391	0.0737	ns
MN	SF	Week_21	Counts	0.9255	0.5133	ns
WN	Control	Week_22	Counts	0.9688	0.8884	ns
MN	Control	Week_22	Counts	0.9392	0.6311	ns
WN	Needle	Week_22	Counts	0.8309	0.0816	ns
MN	Needle	Week_22	Counts	0.9453	0.6865	ns

WN	SF	Week_22	Counts	0.8552	0.1075	ns
MN	SF	Week_22	Counts	0.9137	0.4219	ns

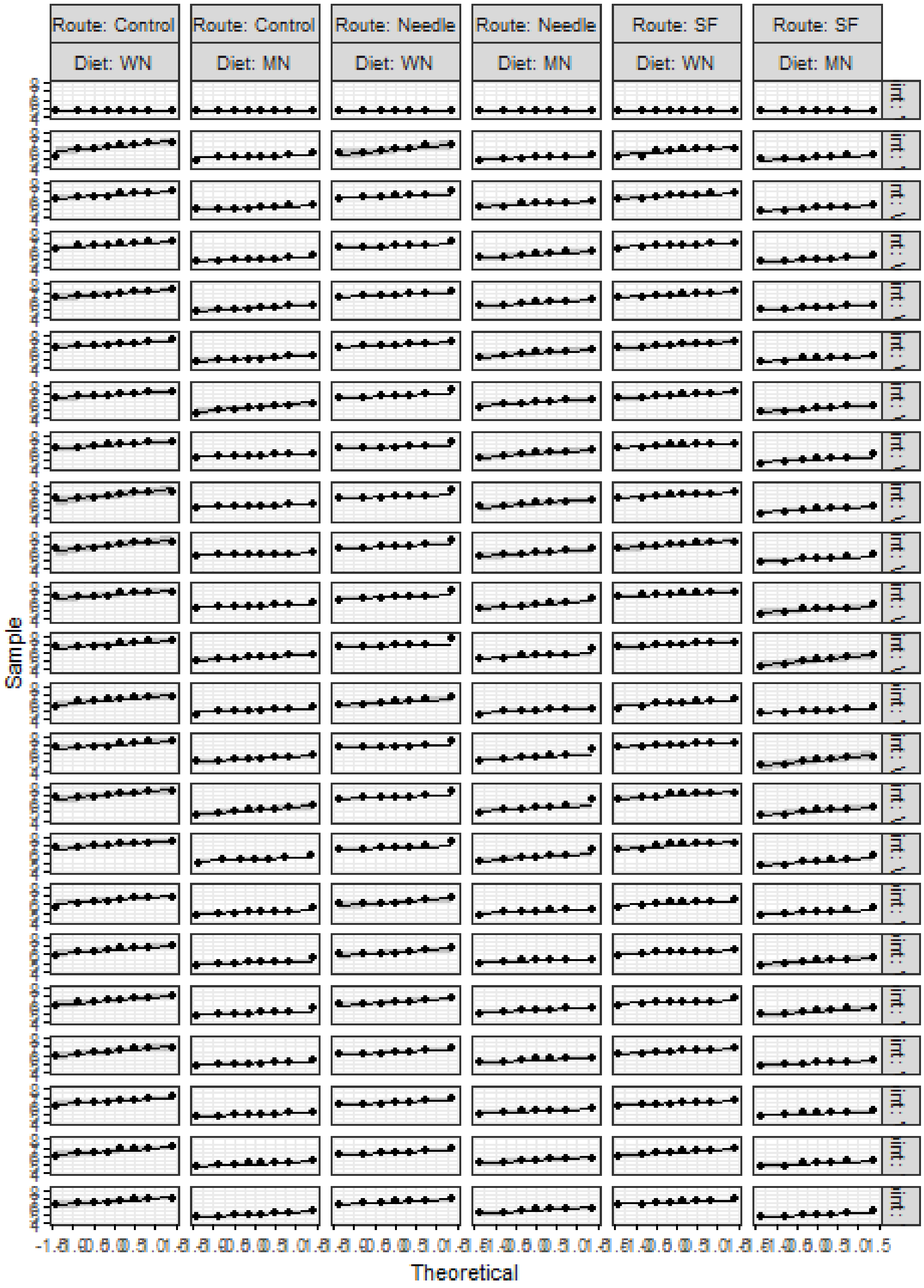

Fig.3a-1: QQ-plots of repeatedly measured mouse weights split into groups by predictor variables

Homogeneity of variance

The assessment of homogeneity of variance was conducted by Levene's test for the dataset split by the within-subject factor ("Time_point"). The analysis output showed that assumption of homogeneity between groups held for each week (Appendix table 88).

Appendix Table 88

Assessment of homogeneity of variance by week

Weeks p.i.	df1	df2	statistic	p	sig.
Pre-Infection					
Week_0	5	39	0.5518	0.7360	ns
Week_1	5	39	1.2268	0.3151	ns
Week_2	5	39	0.4165	0.8344	ns
Week_3	5	39	0.3987	0.8467	ns
Week_4	5	39	0.8811	0.5029	ns
Week_5	5	39	0.6984	0.6279	ns
Post-Infestion					
Week_6	5	39	1.0550	0.3998	ns
Week_7	5	39	1.1844	0.3344	ns
Week_8	5	39	0.8324	0.5347	ns
Week_9	5	39	0.4061	0.8416	ns
Week_10	5	39	0.5260	0.7551	ns
Week_11	5	39	0.3293	0.8922	ns
Week_12	5	39	0.5888	0.7084	ns
Week_13	5	39	0.2664	0.9287	ns
Week_14	5	39	0.1285	0.9850	ns
Week_15	5	39	0.8254	0.5393	ns
Week_16	5	39	1.5470	0.1979	ns
Week_17	5	39	1.6538	0.1688	ns
Week_18	5	39	0.9135	0.4824	ns
Week_19	5	39	0.7931	0.5612	ns
Week_20	5	39	0.9508	0.4595	ns
Week_21	5	39	0.2343	0.9451	ns
Week_22	5	38	0.3294	0.8921	ns

Outliers

It can be difficult to determine outliers in small datasets reliably as the analysis is dependent on the interquartile range of the data per group. We attempted it anyway and found a total of 53 hypothetical outliers of which 7 were classed as extreme (Appendix table 89).

Appendix Table 89

List of possible outliers

Diet	Route	Time_point	is.outlier	is.extreme
Pre-Infection				
WN	Control	Week_0	TRUE	FALSE
WN	Control	Week_1	TRUE	FALSE
MN	Control	Week_1	TRUE	FALSE
MN	Control	Week_1	TRUE	TRUE
MN	Needle	Week_1	TRUE	FALSE
WN	SF	Week_1	TRUE	FALSE
MN	Control	Week_2	TRUE	FALSE
MN	Control	Week_2	TRUE	TRUE
WN	SF	Week_2	TRUE	FALSE
WN	SF	Week_2	TRUE	FALSE
MN	SF	Week_2	TRUE	FALSE
WN	Control	Week_3	TRUE	FALSE
MN	Control	Week_3	TRUE	FALSE
MN	Control	Week_3	TRUE	FALSE
MN	Needle	Week_3	TRUE	FALSE
WN	SF	Week_3	TRUE	FALSE
MN	SF	Week_3	TRUE	FALSE
MN	Control	Week_4	TRUE	FALSE
MN	Control	Week_4	TRUE	FALSE
MN	Needle	Week_4	TRUE	TRUE
WN	SF	Week_4	TRUE	FALSE
MN	Control	Week_5	TRUE	TRUE
MN	Control	Week_5	TRUE	FALSE
WN	SF	Week_5	TRUE	FALSE
WN	SF	Week_5	TRUE	FALSE
Post-Infestion				
MN	Control	Week_6	TRUE	FALSE
MN	Control	Week_6	TRUE	FALSE
WN	SF	Week_9	TRUE	FALSE
WN	Needle	Week_10	TRUE	FALSE
WN	Needle	Week_11	TRUE	FALSE
WN	SF	Week_11	TRUE	FALSE
WN	Needle	Week_12	TRUE	FALSE
WN	Needle	Week_14	TRUE	FALSE
MN	Control	Week_15	TRUE	FALSE

WN	Needle	Week_15	TRUE	FALSE
WN	SF	Week_15	TRUE	FALSE
MN	SF	Week_15	TRUE	FALSE
MN	Control	Week_16	TRUE	FALSE
WN	Needle	Week_16	TRUE	TRUE
MN	Control	Week_17	TRUE	FALSE
WN	Needle	Week_17	TRUE	FALSE
MN	Control	Week_18	TRUE	FALSE
WN	Needle	Week_18	TRUE	FALSE
WN	Needle	Week_19	TRUE	TRUE
MN	Needle	Week_19	TRUE	FALSE
WN	Needle	Week_20	TRUE	TRUE
MN	Needle	Week_20	TRUE	FALSE
WN	Needle	Week_21	TRUE	FALSE
MN	Needle	Week_21	TRUE	FALSE
MN	Control	Week_22	TRUE	FALSE
MN	Control	Week_22	TRUE	FALSE
WN	Needle	Week_22	TRUE	FALSE
MN	Needle	Week_22	TRUE	FALSE

Three-way mixed analysis

Based on the assumption tests, we decided to apply a Robust three-way ANOVA to the dataset to determine the effects of “Diet”, infection “Route” and time pre and post infection (“Time_point”) on mouse weight over time (Appendix table 90). The test output showed that all three individual predictors were statistically significant, so were all two-way and the three-way interaction terms.

Appendix Table 90

Robust three-way mixed ANOVA

Predictors	value	p.value	sig.
Diet	4726.0759	0.0001	****
Route	53.7814	0.0001	****
Time_point	15104.9064	0.0010	***
Diet:Route	155.4204	0.0010	***
Diet:Time_point	4280.0234	0.0010	***
Route:Time_point	106.9048	0.0010	***
Diet:Route:Time_point	204.8501	0.0010	***

We looked for main effects by splitting the data by the within-subject factor (“Time_point”) and analyzed the remaining two predictor (“Diet” and “Roue”) by a Robust two-way ANOVA. The results showed that

both predictors, “Diet” and “Route”, produced statistically significant p-values. While “Diet” had always statistical significance with the exception of “Week_0”, which was unsurprising considering the large gap in body weight between well-nourished and malnourished mice otherwise observed during the 22 week period (Fig. 3a). “Route” was only a statistically significant predictor between “Week_9” and “Week_17”, and the interaction term was statistically significant between “Week_7” and “Week_18” (Appendix table 91). This suggested that the effects of the “Route” of infection were only observed for a limited period of time post infection, while the effects of “Diet” were omnipresent and already established at the point of infection. The interaction suggested that within one or both dietary groups statistically significant differences were observed due to infection route.

Appendix Table 91

Robust two-way ANOVA

Weeks p.i.	Predictor	value	p.value	Sig.
Pre-Infection				
Week_0	Diet	0.6436	0.429	ns
Week_0	Route	4.8913	0.116	ns
Week_0	Diet:Route	0.5024	0.786	ns
Week_1	Diet	83.2007	0.001	***
Week_1	Route	5.6282	0.088	+
Week_1	Diet:Route	2.2941	0.349	ns
Week_2	Diet	128.0593	0.001	***
Week_2	Route	2.6197	0.298	ns
Week_2	Diet:Route	2.3070	0.342	ns
Week_3	Diet	187.7605	0.001	***
Week_3	Route	1.5396	0.484	ns
Week_3	Diet:Route	2.3585	0.335	ns
Week_4	Diet	192.4720	0.001	***
Week_4	Route	0.3976	0.826	ns
Week_4	Diet:Route	6.0356	0.073	+
Week_5	Diet	212.5551	0.001	***
Week_5	Route	0.4535	0.804	ns
Week_5	Diet:Route	5.4285	0.091	+
Post-Infestion				
Week_6	Diet	262.5681	0.001	***
Week_6	Route	2.5042	0.313	ns
Week_6	Diet:Route	6.2856	0.064	+
Week_7	Diet	330.1662	0.001	***
Week_7	Route	5.1005	0.106	ns
Week_7	Diet:Route	7.2688	0.045	*

Week_8	Diet	263.7557	0.001	***
Week_8	Route	4.9514	0.110	ns
Week_8	Diet:Route	7.9498	0.034	*
Week_9	Diet	335.2713	0.001	***
Week_9	Route	8.9642	0.023	*
Week_9	Diet:Route	9.4158	0.019	*
Week_10	Diet	304.2046	0.001	***
Week_10	Route	6.7550	0.053	+
Week_10	Diet:Route	9.4577	0.019	*
Week_11	Diet	378.3575	0.001	***
Week_11	Route	10.0779	0.016	*
Week_11	Diet:Route	16.3481	0.002	**
Week_12	Diet	326.4604	0.001	***
Week_12	Route	12.5730	0.006	**
Week_12	Diet:Route	14.7796	0.003	**
Week_13	Diet	302.1738	0.001	***
Week_13	Route	13.4748	0.005	**
Week_13	Diet:Route	14.6520	0.004	**
Week_14	Diet	208.9694	0.001	***
Week_14	Route	10.6331	0.013	*
Week_14	Diet:Route	13.2573	0.005	**
Week_15	Diet	231.8533	0.001	***
Week_15	Route	10.0367	0.017	*
Week_15	Diet:Route	11.3226	0.011	*
Week_16	Diet	210.5225	0.001	***
Week_16	Route	8.7925	0.026	*
Week_16	Diet:Route	11.9954	0.009	**
Week_17	Diet	177.0197	0.001	***
Week_17	Route	7.6225	0.040	*
Week_17	Diet:Route	11.2424	0.011	*
Week_18	Diet	208.5641	0.001	***
Week_18	Route	3.5529	0.204	ns
Week_18	Diet:Route	11.4103	0.011	*
Week_19	Diet	230.7852	0.001	***
Week_19	Route	4.5003	0.139	ns
Week_19	Diet:Route	3.4689	0.212	ns
Week_20	Diet	259.4895	0.001	***
Week_20	Route	2.0155	0.395	ns
Week_20	Diet:Route	4.1171	0.163	ns
Week_21	Diet	227.8845	0.001	***
Week_21	Route	0.7795	0.689	ns

Week_21	Diet:Route	4.3443	0.143	ns
Week_22	Diet	215.7785	0.001	***
Week_22	Route	2.1574	0.367	ns
Week_22	Diet:Route	7.1401	0.048	*

For the analysis of the simple simple main effect for each respective between-subject factor, we performed Robust one-way ANOVAs with individual between-subject factor of the data split by the other two predictors. The results showed that "Diet" caused statistically significant differences with the exception of "Week_0", which was prior to the assignment of special diets (Appendix table 92). "Route only showed occasionally statistical significant difference; most commonly between"Week_6" and "Week_18", which was only associated with the malnourished group (Appendix table 93).

Appendix Table 92

Robust one-way ANOVA

Time_point	Factor	Effect	test	df1	df2	p.value	effsize	CI_lower	CI_upper	Sig.
Split by Route										
Week_0	Control	Diet	0.9463	1	13.9443	0.3472	0.3265	0.0009	0.7239	ns
Week_0	Needle	Diet	0.0742	1	8.1104	0.7921	0.0880	0.0018	0.8640	ns
Week_0	SF	Diet	0.0073	1	12.9968	0.9334	0.2208	0.0000	0.6874	ns
Week_1	Control	Diet	30.9197	1	10.5864	0.0002	0.9867	0.7579	1.1607	***
Week_1	Needle	Diet	33.6082	1	8.7119	0.0003	1.0360	0.9235	1.1585	***
Week_1	SF	Diet	19.4931	1	10.7930	0.0011	0.9022	0.5425	1.0997	**
Week_2	Control	Diet	46.6180	1	12.5452	<0.0001	1.0575	0.9074	1.1649	****
Week_2	Needle	Diet	49.7907	1	10.7859	<0.0001	1.0994	0.9863	1.2523	****
Week_2	SF	Diet	32.7851	1	11.8409	0.0001	0.9999	0.8331	1.1639	***
Week_3	Control	Diet	71.4565	1	12.4770	<0.0001	1.0818	0.9760	1.1524	****
Week_3	Needle	Diet	66.2027	1	10.2527	<0.0001	1.1338	1.0156	1.2552	****
Week_3	SF	Diet	50.8531	1	11.8824	<0.0001	1.0649	0.9792	1.1777	****
Week_4	Control	Diet	87.8417	1	12.1493	<0.0001	1.1032	1.0040	1.1413	****
Week_4	Needle	Diet	60.9978	1	10.2379	<0.0001	1.1344	1.0132	1.2331	****
Week_4	SF	Diet	45.9854	1	11.0139	<0.0001	1.0600	0.9189	1.1884	****
Week_5	Control	Diet	100.3686	1	12.7279	<0.0001	1.1053	1.0363	1.1542	****
Week_5	Needle	Diet	56.4055	1	11.6501	<0.0001	1.0898	0.9942	1.2567	****
Week_5	SF	Diet	58.6004	1	10.7527	<0.0001	1.0839	0.9806	1.2003	****
Week_6	Control	Diet	88.8819	1	12.0825	<0.0001	1.0922	1.0234	1.1534	****
Week_6	Needle	Diet	54.4571	1	11.9346	<0.0001	1.0689	0.9799	1.2291	****
Week_6	SF	Diet	138.7395	1	12.9947	<0.0001	1.1099	1.0636	1.1608	****
Week_7	Control	Diet	139.0888	1	9.6090	<0.0001	1.1113	1.0622	1.1520	****
Week_7	Needle	Diet	79.5424	1	11.6833	<0.0001	1.1279	1.0372	1.2009	****

Week_7	SF	Diet	115.0358	1	12.9933	<0.0001	1.1055	1.0434	1.1664	****
Week_8	Control	Diet	108.6133	1	11.1301	<0.0001	1.1069	1.0373	1.1390	****
Week_8	Needle	Diet	64.9819	1	11.9851	<0.0001	1.1008	1.0198	1.1840	****
Week_8	SF	Diet	90.4757	1	12.9631	<0.0001	1.0930	1.0282	1.1714	****
Week_9	Control	Diet	138.2405	1	12.6407	<0.0001	1.0938	1.0567	1.1317	****
Week_9	Needle	Diet	59.2647	1	11.9764	<0.0001	1.1281	1.0054	1.2280	****
Week_9	SF	Diet	156.4098	1	12.9750	<0.0001	1.1141	1.0647	1.1674	****
Week_10	Control	Diet	142.1475	1	11.3023	<0.0001	1.1193	1.0511	1.1490	****
Week_10	Needle	Diet	56.2825	1	11.9764	<0.0001	1.1457	1.0089	1.2562	****
Week_10	SF	Diet	118.0082	1	12.9925	<0.0001	1.0907	1.0405	1.1480	****
Week_11	Control	Diet	184.1228	1	12.2479	<0.0001	1.0992	1.0666	1.1448	****
Week_11	Needle	Diet	59.3268	1	11.7074	<0.0001	1.1027	1.0133	1.2393	****
Week_11	SF	Diet	158.6371	1	12.1492	<0.0001	1.1072	1.0679	1.1475	****
Week_12	Control	Diet	124.1778	1	13.5899	<0.0001	1.1083	1.0484	1.1454	****
Week_12	Needle	Diet	63.5146	1	11.9642	<0.0001	1.1025	1.0221	1.2193	****
Week_12	SF	Diet	148.2799	1	12.9212	<0.0001	1.1048	1.0781	1.1607	****
Week_13	Control	Diet	159.1247	1	12.9562	<0.0001	1.1146	1.0659	1.1539	****
Week_13	Needle	Diet	43.9328	1	11.0735	<0.0001	1.0647	0.9797	1.2014	****
Week_13	SF	Diet	124.1739	1	12.1224	<0.0001	1.0937	1.0459	1.1608	****
Week_14	Control	Diet	81.6612	1	13.7559	<0.0001	1.1121	1.0289	1.1862	****
Week_14	Needle	Diet	28.2635	1	11.9462	0.0002	1.1630	0.9098	1.2623	***
Week_14	SF	Diet	121.5173	1	12.1033	<0.0001	1.0929	1.0455	1.1470	****
Week_15	Control	Diet	132.8303	1	11.5452	<0.0001	1.1446	1.0555	1.1641	****
Week_15	Needle	Diet	26.5980	1	10.6701	0.0003	1.1250	0.8928	1.3944	***
Week_15	SF	Diet	127.4723	1	10.3884	<0.0001	1.1094	1.0361	1.1579	****
Week_16	Control	Diet	100.3834	1	8.4872	<0.0001	1.0950	1.0457	1.1333	****
Week_16	Needle	Diet	27.3372	1	11.9350	0.0002	1.1261	0.9201	1.2388	***
Week_16	SF	Diet	110.7167	1	11.2457	<0.0001	1.0967	1.0502	1.1709	****
Week_17	Control	Diet	69.5823	1	8.7894	<0.0001	1.0702	1.0164	1.1259	****
Week_17	Needle	Diet	25.4983	1	11.8638	0.0003	1.0843	0.8552	1.2132	***
Week_17	SF	Diet	104.7430	1	11.4557	<0.0001	1.0962	1.0511	1.1639	****
Week_18	Control	Diet	108.3643	1	12.9680	<0.0001	1.0997	1.0550	1.1702	****
Week_18	Needle	Diet	23.9856	1	11.6560	0.0004	1.0855	0.8390	1.2260	***
Week_18	SF	Diet	123.5300	1	7.7478	<0.0001	1.0956	1.0486	1.1685	****
Week_19	Control	Diet	133.9525	1	12.8372	<0.0001	1.0831	1.0591	1.1506	****
Week_19	Needle	Diet	46.3485	1	11.7773	<0.0001	1.1568	0.9677	1.2498	****
Week_19	SF	Diet	82.2076	1	7.9091	<0.0001	1.0804	1.0251	1.1832	****
Week_20	Control	Diet	157.5335	1	13.9259	<0.0001	1.0929	1.0730	1.1631	****
Week_20	Needle	Diet	44.7878	1	10.7340	<0.0001	1.1444	0.9522	1.2082	****
Week_20	SF	Diet	99.9882	1	7.8372	<0.0001	1.0933	1.0481	1.1832	****
Week_21	Control	Diet	113.5715	1	13.8178	<0.0001	1.0896	1.0582	1.1729	****

Week_21	Needle	Diet	33.1991	1	10.5972	0.0001	1.0890	0.8607	1.2207	***
Week_21	SF	Diet	121.3469	1	11.0681	<0.0001	1.1009	1.0605	1.1678	****
Week_22	Control	Diet	136.2265	1	12.9780	<0.0001	1.1126	1.0586	1.1811	****
Week_22	Needle	Diet	25.6731	1	11.1438	0.0003	1.0823	0.7903	1.2356	***
Week_22	SF	Diet	111.3334	1	10.6612	<0.0001	1.0940	1.0386	1.1744	****
Split by Diet										
Week_0	WN	Route	1.9588	2	12.8725	0.1808	0.4893	0.1490	0.8093	ns
Week_0	MN	Route	1.0762	2	12.1312	0.3713	0.4805	0.1011	0.8476	ns
Week_1	WN	Route	2.2567	2	13.2118	0.1435	0.5778	0.2353	0.9016	ns
Week_1	MN	Route	0.6409	2	12.6553	0.5431	0.4520	0.0778	0.9168	ns
Week_2	WN	Route	1.6924	2	13.2826	0.2215	0.5245	0.1950	0.8207	ns
Week_2	MN	Route	0.0597	2	12.6611	0.9423	0.3626	0.0496	0.9067	ns
Week_3	WN	Route	1.6823	2	12.8046	0.2245	0.5462	0.1318	0.8698	ns
Week_3	MN	Route	0.0564	2	12.3027	0.9454	0.3494	0.0647	0.9018	ns
Week_4	WN	Route	1.8918	2	12.7117	0.1909	0.5329	0.1169	0.8900	ns
Week_4	MN	Route	1.3314	2	12.1316	0.3001	0.5199	0.1351	0.9043	ns
Week_5	WN	Route	0.8912	2	12.7927	0.4342	0.4278	0.0860	0.8367	ns
Week_5	MN	Route	2.4199	2	12.2564	0.1301	0.5479	0.1046	0.8873	ns
Week_6	WN	Route	0.2057	2	12.9847	0.8167	0.3726	0.0547	0.7028	ns
Week_6	MN	Route	4.6012	2	12.5486	0.0317	0.7071	0.3539	0.9990	*
Week_7	WN	Route	1.3374	2	13.0307	0.2963	0.4659	0.0746	0.8409	ns
Week_7	MN	Route	6.6197	2	11.8250	0.0118	0.7229	0.4533	0.9427	*
Week_8	WN	Route	0.5185	2	13.2073	0.6070	0.3676	0.0697	0.7614	ns
Week_8	MN	Route	6.4573	2	12.2947	0.0121	0.7206	0.5076	0.9312	*
Week_9	WN	Route	0.0958	2	13.2133	0.9093	0.3300	0.0387	0.7494	ns
Week_9	MN	Route	9.4920	2	12.4183	0.0032	0.7882	0.6314	0.9290	**
Week_10	WN	Route	0.2026	2	13.3101	0.8191	0.3207	0.0838	0.7937	ns
Week_10	MN	Route	7.8905	2	11.8254	0.0067	0.7947	0.5708	0.9749	**
Week_11	WN	Route	0.2315	2	13.2266	0.7965	0.3586	0.1059	0.7313	ns
Week_11	MN	Route	11.6677	2	11.8647	0.0016	0.8443	0.7023	0.9919	**
Week_12	WN	Route	0.3790	2	13.2137	0.6918	0.3645	0.0436	0.7253	ns
Week_12	MN	Route	12.5813	2	12.6487	0.0010	0.8631	0.7246	1.0332	***
Week_13	WN	Route	0.3800	2	13.2733	0.6911	0.3427	0.0785	0.7311	ns
Week_13	MN	Route	11.0540	2	11.9483	0.0019	0.8523	0.7450	0.9947	**
Week_14	WN	Route	0.1990	2	13.0343	0.8220	0.2911	0.0397	0.7604	ns
Week_14	MN	Route	10.1230	2	12.6525	0.0024	0.8325	0.6854	1.0012	**
Week_15	WN	Route	1.2098	2	12.9990	0.3297	0.5001	0.1221	0.8663	ns
Week_15	MN	Route	6.6894	2	10.4184	0.0135	0.8531	0.5907	1.0766	*
Week_16	WN	Route	0.1823	2	13.0098	0.8354	0.3426	0.0731	0.7330	ns
Week_16	MN	Route	7.9104	2	9.3879	0.0097	0.9453	0.7688	1.1793	**

Week_17	WN	Route	0.1053	2	12.9985	0.9008	0.2752	0.0438	0.7046	ns
Week_17	MN	Route	7.7361	2	9.5588	0.0100	0.9891	0.7925	1.2656	*
Week_18	WN	Route	1.4892	2	11.5452	0.2659	0.4710	0.1032	0.8207	ns
Week_18	MN	Route	4.0466	2	10.9429	0.0484	0.8530	0.4328	1.1840	*
Week_19	WN	Route	0.1186	2	12.3347	0.8891	0.3585	0.0571	0.7342	ns
Week_19	MN	Route	2.5349	2	10.7215	0.1255	0.7664	0.2584	1.2106	ns
Week_20	WN	Route	0.3121	2	12.3417	0.7375	0.3788	0.0410	0.8568	ns
Week_20	MN	Route	1.7845	2	11.1498	0.2126	0.7201	0.1120	1.2486	ns
Week_21	WN	Route	0.9900	2	12.8920	0.3981	0.4395	0.0749	0.7919	ns
Week_21	MN	Route	1.2693	2	12.1936	0.3157	0.5648	0.1110	0.9098	ns
Week_22	WN	Route	1.1111	2	12.8371	0.3589	0.4654	0.1060	0.8061	ns
Week_22	MN	Route	2.6553	2	11.3155	0.1134	0.7444	0.4043	1.2335	ns

Appendix Table 93

Robust one-way ANOVA - significance summary for Route effect

Time_point	Factor	Effect	test	df1	df2	p.value	effsize	CI_lower	CI_upper	Sig.
Week_6	MN	Route	4.6012	2	12.5486	0.0317	0.7071	0.3539	0.9990	*
Week_7	MN	Route	6.6197	2	11.8250	0.0118	0.7229	0.4533	0.9427	*
Week_8	MN	Route	6.4573	2	12.2947	0.0121	0.7206	0.5076	0.9312	*
Week_9	MN	Route	9.4920	2	12.4183	0.0032	0.7882	0.6314	0.9290	**
Week_10	MN	Route	7.8905	2	11.8254	0.0067	0.7947	0.5708	0.9749	**
Week_11	MN	Route	11.6677	2	11.8647	0.0016	0.8443	0.7023	0.9919	**
Week_12	MN	Route	12.5813	2	12.6487	0.0010	0.8631	0.7246	1.0332	***
Week_13	MN	Route	11.0540	2	11.9483	0.0019	0.8523	0.7450	0.9947	**
Week_14	MN	Route	10.1230	2	12.6525	0.0024	0.8325	0.6854	1.0012	**
Week_15	MN	Route	6.6894	2	10.4184	0.0135	0.8531	0.5907	1.0766	*
Week_16	MN	Route	7.9104	2	9.3879	0.0097	0.9453	0.7688	1.1793	**
Week_17	MN	Route	7.7361	2	9.5588	0.0100	0.9891	0.7925	1.2656	*
Week_18	MN	Route	4.0466	2	10.9429	0.0484	0.8530	0.4328	1.1840	*

For the pairwise comparison, we applied a Linear contrast expression. Since the “Diet” predictor only had two factor levels, the output showed the same result as the Robust one-way ANOVA above. For the “Route” predictor, the pairwise comparison presented a more detailed view at where statistically significant differences occurred (Appendix table 94). As for the Robust one-way ANOVA above, all statistical significant differences were observed between “Week_6” and “Week_18” and were restricted to the malnourished groups (Appendix table 95). The main differences between the malnourished groups resided primarily with the needle inoculated group from “Week_6” to “Week_14”, which had had more weight gain than either the malnourished control or sand fly infected groups. From “Week_15” onward, the average mouse weight for the malnourished control group approached that or the needle group and

statistical differences were now only observed compared to the malnourished sand fly infected group, that never seemed to gain weight post infection.

Appendix Table 94

Pairwise comparison by Linear Contrast Expression

Time_point	Factor	Group	Group.1	psihat	ci.lower	ci.upper	p.value	Sig.
Split by Route								
Week_0	Control	WN	MN	-0.0113	-0.0362	0.0136	0.3472	ns
Week_0	Needle	WN	MN	-0.0032	-0.0301	0.0237	0.7921	ns
Week_0	SF	WN	MN	-0.0008	-0.0216	0.0199	0.9334	ns
Week_1	Control	WN	MN	1.1088	0.6678	1.5498	0.0002	***
Week_1	Needle	WN	MN	1.0070	0.6121	1.4019	0.0003	***
Week_1	SF	WN	MN	0.7394	0.3700	1.1089	0.0011	**
Week_2	Control	WN	MN	1.2031	0.8210	1.5852	<0.0001	****
Week_2	Needle	WN	MN	1.0607	0.7290	1.3923	<0.0001	****
Week_2	SF	WN	MN	0.8588	0.5315	1.1860	0.0001	***
Week_3	Control	WN	MN	1.4061	1.0452	1.7670	<0.0001	****
Week_3	Needle	WN	MN	1.1827	0.8599	1.5055	<0.0001	****
Week_3	SF	WN	MN	1.0654	0.7395	1.3913	<0.0001	****
Week_4	Control	WN	MN	1.5285	1.1736	1.8833	<0.0001	****
Week_4	Needle	WN	MN	1.0817	0.7741	1.3893	<0.0001	****
Week_4	SF	WN	MN	1.0296	0.6955	1.3637	<0.0001	****
Week_5	Control	WN	MN	1.4800	1.1601	1.7998	<0.0001	****
Week_5	Needle	WN	MN	1.0373	0.7354	1.3392	<0.0001	****
Week_5	SF	WN	MN	1.1033	0.7852	1.4215	<0.0001	****
Week_6	Control	WN	MN	1.4642	1.1260	1.8023	<0.0001	****
Week_6	Needle	WN	MN	1.0005	0.7049	1.2961	<0.0001	****
Week_6	SF	WN	MN	1.3728	1.1210	1.6245	<0.0001	****
Week_7	Control	WN	MN	1.5867	1.2853	1.8881	<0.0001	****
Week_7	Needle	WN	MN	1.1054	0.8345	1.3762	<0.0001	****
Week_7	SF	WN	MN	1.2398	0.9901	1.4895	<0.0001	****
Week_8	Control	WN	MN	1.5293	1.2068	1.8519	<0.0001	****
Week_8	Needle	WN	MN	0.9968	0.7274	1.2663	<0.0001	****
Week_8	SF	WN	MN	1.3011	1.0055	1.5967	<0.0001	****
Week_9	Control	WN	MN	1.5496	1.2641	1.8352	<0.0001	****
Week_9	Needle	WN	MN	1.0374	0.7437	1.3311	<0.0001	****
Week_9	SF	WN	MN	1.5182	1.2559	1.7805	<0.0001	****
Week_10	Control	WN	MN	1.5132	1.2348	1.7917	<0.0001	****
Week_10	Needle	WN	MN	0.9966	0.7071	1.2861	<0.0001	****
Week_10	SF	WN	MN	1.4703	1.1779	1.7628	<0.0001	****

Week_11	Control	WN	MN	1.7096	1.4357	1.9835	<0.0001	****
Week_11	Needle	WN	MN	1.0347	0.7412	1.3283	<0.0001	****
Week_11	SF	WN	MN	1.6694	1.3810	1.9578	<0.0001	****
Week_12	Control	WN	MN	1.6941	1.3671	2.0211	<0.0001	****
Week_12	Needle	WN	MN	1.0008	0.7271	1.2745	<0.0001	****
Week_12	SF	WN	MN	1.5285	1.2571	1.7998	<0.0001	****
Week_13	Control	WN	MN	1.7818	1.4765	2.0870	<0.0001	****
Week_13	Needle	WN	MN	1.0233	0.6838	1.3628	<0.0001	****
Week_13	SF	WN	MN	1.6597	1.3356	1.9839	<0.0001	****
Week_14	Control	WN	MN	1.5898	1.2118	1.9677	<0.0001	****
Week_14	Needle	WN	MN	0.8714	0.5141	1.2287	0.0002	***
Week_14	SF	WN	MN	1.5966	1.2813	1.9118	<0.0001	****
Week_15	Control	WN	MN	1.3339	1.0806	1.5871	<0.0001	****
Week_15	Needle	WN	MN	0.9000	0.5145	1.2856	0.0003	***
Week_15	SF	WN	MN	1.6695	1.3417	1.9973	<0.0001	****
Week_16	Control	WN	MN	1.3148	1.0152	1.6145	<0.0001	****
Week_16	Needle	WN	MN	0.8993	0.5243	1.2743	0.0002	***
Week_16	SF	WN	MN	1.7211	1.3620	2.0801	<0.0001	****
Week_17	Control	WN	MN	1.0999	0.8005	1.3993	<0.0001	****
Week_17	Needle	WN	MN	0.9074	0.5154	1.2994	0.0003	***
Week_17	SF	WN	MN	1.6611	1.3056	2.0167	<0.0001	****
Week_18	Control	WN	MN	1.3589	1.0769	1.6410	<0.0001	****
Week_18	Needle	WN	MN	0.9492	0.5255	1.3729	0.0004	***
Week_18	SF	WN	MN	1.7918	1.4179	2.1656	<0.0001	****
Week_19	Control	WN	MN	1.5472	1.2580	1.8364	<0.0001	****
Week_19	Needle	WN	MN	1.2893	0.8758	1.7028	<0.0001	****
Week_19	SF	WN	MN	1.7998	1.3412	2.2585	<0.0001	****
Week_20	Control	WN	MN	1.7273	1.4320	2.0226	<0.0001	****
Week_20	Needle	WN	MN	1.3372	0.8961	1.7784	<0.0001	****
Week_20	SF	WN	MN	1.8728	1.4393	2.3063	<0.0001	****
Week_21	Control	WN	MN	1.8837	1.5042	2.2633	<0.0001	****
Week_21	Needle	WN	MN	1.3298	0.8195	1.8401	0.0001	***
Week_21	SF	WN	MN	1.8646	1.4923	2.2369	<0.0001	****
Week_22	Control	WN	MN	1.7596	1.4338	2.0853	<0.0001	****
Week_22	Needle	WN	MN	1.1723	0.6638	1.6807	0.0003	***
Week_22	SF	WN	MN	1.9461	1.5386	2.3537	<0.0001	****
Split by Diet								
Week_0	WN	Control	Needle	0.0125	-0.0130	0.0381	0.2999	ns
Week_0	WN	Control	SF	-0.0028	-0.0316	0.0259	0.7940	ns
Week_0	WN	Needle	SF	-0.0154	-0.0387	0.0080	0.2847	ns

Week_0	MN	Control	Needle	0.0206	-0.0171	0.0584	0.4743	ns
Week_0	MN	Control	SF	0.0077	-0.0215	0.0368	0.4862	ns
Week_0	MN	Needle	SF	-0.0130	-0.0487	0.0227	0.4862	ns
Week_1	WN	Control	Needle	0.2286	-0.4120	0.8692	0.3497	ns
Week_1	WN	Control	SF	0.4935	-0.1262	1.1133	0.1499	ns
Week_1	WN	Needle	SF	0.2649	-0.3182	0.8481	0.3497	ns
Week_1	MN	Control	Needle	0.1268	-0.2001	0.4536	0.4986	ns
Week_1	MN	Control	SF	0.1242	-0.2112	0.4595	0.4986	ns
Week_1	MN	Needle	SF	-0.0026	-0.3095	0.3043	0.9820	ns
Week_2	WN	Control	Needle	0.1875	-0.3279	0.7030	0.3452	ns
Week_2	WN	Control	SF	0.3592	-0.1556	0.8741	0.2437	ns
Week_2	WN	Needle	SF	0.1717	-0.3048	0.6482	0.3452	ns
Week_2	MN	Control	Needle	0.0451	-0.3173	0.4074	0.9116	ns
Week_2	MN	Control	SF	0.0148	-0.3414	0.3711	0.9116	ns
Week_2	MN	Needle	SF	-0.0303	-0.3601	0.2996	0.9116	ns
Week_3	WN	Control	Needle	0.1814	-0.3168	0.6796	0.4229	ns
Week_3	WN	Control	SF	0.3088	-0.1435	0.7612	0.2563	ns
Week_3	WN	Needle	SF	0.1274	-0.2962	0.5510	0.4229	ns
Week_3	MN	Control	Needle	-0.0420	-0.3777	0.2936	0.9443	ns
Week_3	MN	Control	SF	-0.0319	-0.4463	0.3824	0.9443	ns
Week_3	MN	Needle	SF	0.0101	-0.3856	0.4058	0.9443	ns
Week_4	WN	Control	Needle	0.2594	-0.2276	0.7464	0.2569	ns
Week_4	WN	Control	SF	0.3197	-0.1244	0.7638	0.2133	ns
Week_4	WN	Needle	SF	0.0603	-0.3414	0.4620	0.6858	ns
Week_4	MN	Control	Needle	-0.1874	-0.5063	0.1314	0.4015	ns
Week_4	MN	Control	SF	-0.1792	-0.6052	0.2468	0.4015	ns
Week_4	MN	Needle	SF	0.0082	-0.4045	0.4209	0.9562	ns
Week_5	WN	Control	Needle	0.1664	-0.2682	0.6009	0.4755	ns
Week_5	WN	Control	SF	0.1940	-0.2016	0.5895	0.4755	ns
Week_5	WN	Needle	SF	0.0276	-0.3392	0.3943	0.8391	ns
Week_5	MN	Control	Needle	-0.2763	-0.6135	0.0609	0.1326	ns
Week_5	MN	Control	SF	-0.1827	-0.5918	0.2264	0.3615	ns
Week_5	MN	Needle	SF	0.0936	-0.3203	0.5076	0.5426	ns
Week_6	WN	Control	Needle	0.1055	-0.3411	0.5520	0.7466	ns
Week_6	WN	Control	SF	0.0515	-0.3763	0.4794	0.7466	ns
Week_6	WN	Needle	SF	-0.0539	-0.4140	0.3062	0.7466	ns
Week_6	MN	Control	Needle	-0.3582	-0.7006	-0.0157	0.0336	*
Week_6	MN	Control	SF	-0.0399	-0.3543	0.2746	0.7359	ns
Week_6	MN	Needle	SF	0.3183	-0.0155	0.6521	0.0336	*
Week_7	WN	Control	Needle	0.1199	-0.3041	0.5439	0.4544	ns
Week_7	WN	Control	SF	0.2430	-0.1651	0.6510	0.3849	ns

Week_7	WN	Needle	SF	0.1231	-0.2224	0.4686	0.4544	ns
Week_7	MN	Control	Needle	-0.3614	-0.6311	-0.0918	0.0101	*
Week_7	MN	Control	SF	-0.1039	-0.3720	0.1641	0.3033	ns
Week_7	MN	Needle	SF	0.2575	-0.0528	0.5679	0.0623	+
Week_8	WN	Control	Needle	0.1235	-0.2994	0.5463	0.6560	ns
Week_8	WN	Control	SF	0.1682	-0.2749	0.6113	0.6560	ns
Week_8	WN	Needle	SF	0.0447	-0.3199	0.4094	0.7439	ns
Week_8	MN	Control	Needle	-0.4090	-0.7248	-0.0932	0.0117	*
Week_8	MN	Control	SF	-0.0600	-0.3788	0.2588	0.6141	ns
Week_8	MN	Needle	SF	0.3490	0.0006	0.6974	0.0262	*
Week_9	WN	Control	Needle	0.0432	-0.3508	0.4372	0.8865	ns
Week_9	WN	Control	SF	0.0626	-0.3172	0.4424	0.8865	ns
Week_9	WN	Needle	SF	0.0194	-0.3437	0.3825	0.8865	ns
Week_9	MN	Control	Needle	-0.4690	-0.7999	-0.1381	0.0032	**
Week_9	MN	Control	SF	0.0312	-0.2712	0.3335	0.7833	ns
Week_9	MN	Needle	SF	0.5002	0.1608	0.8396	0.0032	**
Week_10	WN	Control	Needle	0.0840	-0.3049	0.4729	0.8692	ns
Week_10	WN	Control	SF	0.0835	-0.3128	0.4798	0.8692	ns
Week_10	WN	Needle	SF	-0.0005	-0.3659	0.3649	0.9970	ns
Week_10	MN	Control	Needle	-0.4326	-0.7551	-0.1101	0.0061	**
Week_10	MN	Control	SF	0.0406	-0.2742	0.3554	0.7265	ns
Week_10	MN	Needle	SF	0.4732	0.1053	0.8411	0.0061	**
Week_11	WN	Control	Needle	0.0946	-0.2760	0.4651	0.7218	ns
Week_11	WN	Control	SF	0.0489	-0.3150	0.4128	0.7218	ns
Week_11	WN	Needle	SF	-0.0457	-0.3762	0.2848	0.7218	ns
Week_11	MN	Control	Needle	-0.5803	-0.9256	-0.2350	0.0021	**
Week_11	MN	Control	SF	0.0087	-0.3363	0.3537	0.9452	ns
Week_11	MN	Needle	SF	0.5891	0.1917	0.9864	0.0023	**
Week_12	WN	Control	Needle	0.0957	-0.3009	0.4923	0.7817	ns
Week_12	WN	Control	SF	0.1294	-0.2682	0.5269	0.7817	ns
Week_12	WN	Needle	SF	0.0336	-0.3029	0.3702	0.7901	ns
Week_12	MN	Control	Needle	-0.5976	-0.9609	-0.2343	0.0013	**
Week_12	MN	Control	SF	-0.0363	-0.3952	0.3225	0.7876	ns
Week_12	MN	Needle	SF	0.5613	0.2112	0.9114	0.0013	**
Week_13	WN	Control	Needle	0.0787	-0.3186	0.4761	0.6942	ns
Week_13	WN	Control	SF	0.1318	-0.2668	0.5304	0.6942	ns
Week_13	WN	Needle	SF	0.0531	-0.3059	0.4121	0.6942	ns
Week_13	MN	Control	Needle	-0.6798	-1.0978	-0.2617	0.0023	**
Week_13	MN	Control	SF	0.0098	-0.3851	0.4047	0.9464	ns
Week_13	MN	Needle	SF	0.6896	0.2244	1.1548	0.0023	**
Week_14	WN	Control	Needle	0.0949	-0.3429	0.5328	0.8851	ns

Week_14	WN	Control	SF	0.0816	-0.3189	0.4821	0.8851	ns
Week_14	WN	Needle	SF	-0.0133	-0.4111	0.3845	0.9282	ns
Week_14	MN	Control	Needle	-0.6234	-1.1087	-0.1382	0.0060	**
Week_14	MN	Control	SF	0.0884	-0.3834	0.5602	0.6188	ns
Week_14	MN	Needle	SF	0.7118	0.2609	1.1628	0.0029	**
Week_15	WN	Control	Needle	0.2235	-0.1577	0.6047	0.4048	ns
Week_15	WN	Control	SF	0.1138	-0.2296	0.4572	0.4053	ns
Week_15	WN	Needle	SF	-0.1097	-0.4595	0.2401	0.4053	ns
Week_15	MN	Control	Needle	-0.2103	-0.6700	0.2494	0.2131	ns
Week_15	MN	Control	SF	0.4494	0.0467	0.8521	0.0158	*
Week_15	MN	Needle	SF	0.6597	0.1356	1.1839	0.0143	*
Week_16	WN	Control	Needle	0.1005	-0.3643	0.5653	0.8588	ns
Week_16	WN	Control	SF	0.0285	-0.3980	0.4550	0.8588	ns
Week_16	WN	Needle	SF	-0.0720	-0.4872	0.3432	0.8588	ns
Week_16	MN	Control	Needle	-0.3150	-0.7167	0.0866	0.0478	*
Week_16	MN	Control	SF	0.4347	0.0119	0.8575	0.0243	*
Week_16	MN	Needle	SF	0.7498	0.2463	1.2532	0.0045	**
Week_17	WN	Control	Needle	0.0257	-0.4436	0.4950	0.8838	ns
Week_17	WN	Control	SF	-0.0446	-0.4711	0.3820	0.8838	ns
Week_17	WN	Needle	SF	-0.0703	-0.4944	0.3538	0.8838	ns
Week_17	MN	Control	Needle	-0.1668	-0.5930	0.2594	0.2738	ns
Week_17	MN	Control	SF	0.5167	0.1012	0.9322	0.0097	**
Week_17	MN	Needle	SF	0.6834	0.1699	1.1969	0.0097	**
Week_18	WN	Control	Needle	0.2123	-0.2339	0.6586	0.3239	ns
Week_18	WN	Control	SF	-0.0312	-0.3636	0.3013	0.7989	ns
Week_18	WN	Needle	SF	-0.2435	-0.6451	0.1581	0.3239	ns
Week_18	MN	Control	Needle	-0.1974	-0.6800	0.2852	0.2688	ns
Week_18	MN	Control	SF	0.4017	-0.0874	0.8907	0.0626	+
Week_18	MN	Needle	SF	0.5990	0.0178	1.1803	0.0452	*
Week_19	WN	Control	Needle	0.0355	-0.4149	0.4858	0.8431	ns
Week_19	WN	Control	SF	0.0648	-0.2929	0.4226	0.8431	ns
Week_19	WN	Needle	SF	0.0294	-0.3806	0.4394	0.8431	ns
Week_19	MN	Control	Needle	-0.2224	-0.6893	0.2444	0.2045	ns
Week_19	MN	Control	SF	0.3175	-0.2732	0.9083	0.2045	ns
Week_19	MN	Needle	SF	0.5399	-0.1059	1.1858	0.1208	ns
Week_20	WN	Control	Needle	0.1231	-0.2939	0.5401	0.7165	ns
Week_20	WN	Control	SF	0.0453	-0.2885	0.3791	0.7165	ns
Week_20	WN	Needle	SF	-0.0778	-0.4556	0.3000	0.7165	ns
Week_20	MN	Control	Needle	-0.2670	-0.8043	0.2703	0.2826	ns
Week_20	MN	Control	SF	0.1908	-0.3773	0.7588	0.3594	ns
Week_20	MN	Needle	SF	0.4578	-0.2003	1.1158	0.2396	ns

Week_21	WN	Control	Needle	0.2520	-0.2269	0.7309	0.5255	ns
Week_21	WN	Control	SF	0.0955	-0.3169	0.5080	0.5417	ns
Week_21	WN	Needle	SF	-0.1565	-0.6057	0.2927	0.5331	ns
Week_21	MN	Control	Needle	-0.3019	-0.9467	0.3428	0.3291	ns
Week_21	MN	Control	SF	0.0764	-0.4447	0.5976	0.6962	ns
Week_21	MN	Needle	SF	0.3784	-0.2771	1.0338	0.3291	ns
Week_22	WN	Control	Needle	0.2645	-0.2216	0.7507	0.3912	ns
Week_22	WN	Control	SF	0.0605	-0.3400	0.4611	0.6904	ns
Week_22	WN	Needle	SF	-0.2040	-0.6813	0.2733	0.3912	ns
Week_22	MN	Control	Needle	-0.3228	-0.9302	0.2847	0.2120	ns
Week_22	MN	Control	SF	0.2471	-0.2738	0.7679	0.2120	ns
Week_22	MN	Needle	SF	0.5698	-0.0941	1.2338	0.1082	ns

Appendix Table 95

Pairwise comparison by Linear Contrast Expression - sig. summary

Predictor	Time_point	Factor	Group	Group.1	psihat	ci.lower	ci.upper	p.value	Sig.
Route	Week_6	MN	Control	Needle	-0.3582	-0.7006	-0.0157	0.0336	*
Route	Week_6	MN	Needle	SF	0.3183	-0.0155	0.6521	0.0336	*
Route	Week_7	MN	Control	Needle	-0.3614	-0.6311	-0.0918	0.0101	*
Route	Week_8	MN	Control	Needle	-0.4090	-0.7248	-0.0932	0.0117	*
Route	Week_8	MN	Needle	SF	0.3490	0.0006	0.6974	0.0262	*
Route	Week_9	MN	Control	Needle	-0.4690	-0.7999	-0.1381	0.0032	**
Route	Week_9	MN	Needle	SF	0.5002	0.1608	0.8396	0.0032	**
Route	Week_10	MN	Control	Needle	-0.4326	-0.7551	-0.1101	0.0061	**
Route	Week_10	MN	Needle	SF	0.4732	0.1053	0.8411	0.0061	**
Route	Week_11	MN	Control	Needle	-0.5803	-0.9256	-0.2350	0.0021	**
Route	Week_11	MN	Needle	SF	0.5891	0.1917	0.9864	0.0023	**
Route	Week_12	MN	Control	Needle	-0.5976	-0.9609	-0.2343	0.0013	**
Route	Week_12	MN	Needle	SF	0.5613	0.2112	0.9114	0.0013	**
Route	Week_13	MN	Control	Needle	-0.6798	-1.0978	-0.2617	0.0023	**
Route	Week_13	MN	Needle	SF	0.6896	0.2244	1.1548	0.0023	**
Route	Week_14	MN	Control	Needle	-0.6234	-1.1087	-0.1382	0.0060	**
Route	Week_14	MN	Needle	SF	0.7118	0.2609	1.1628	0.0029	**
Route	Week_15	MN	Control	SF	0.4494	0.0467	0.8521	0.0158	*
Route	Week_15	MN	Needle	SF	0.6597	0.1356	1.1839	0.0143	*
Route	Week_16	MN	Control	Needle	-0.3150	-0.7167	0.0866	0.0478	*
Route	Week_16	MN	Control	SF	0.4347	0.0119	0.8575	0.0243	*
Route	Week_16	MN	Needle	SF	0.7498	0.2463	1.2532	0.0045	**
Route	Week_17	MN	Control	SF	0.5167	0.1012	0.9322	0.0097	**

Route	Week_17	MN	Needle	SF	0.6834	0.1699	1.1969	0.0097	**
Route	Week_18	MN	Needle	SF	0.5990	0.0178	1.1803	0.0452	*

Conclusion

In conclusion, "Diet" was identified of being the most potent predictor for mouse weight gain over time. Interestingly, between the well-nourished groups, we never observed statistically significant differences in weight gains over time, suggesting that in that state, and conversely to the malnourished mouse groups, mouse weight was not affected by infection status or "Route". These data support the hypothesis that the nutritional state of an individual can directly impact their weight before and during *Leishmania donovani* infection.

Panel b

Data analysis

We analyzed a total of *N*=98 well-nourished (WN) and malnourished (MN) BALB/c mice for the occurrence frequency of a >=20% weight loss post-intradermal *Leishmania donovani* infection either by "needle" injection or sand fly bite (SF) (*N*: MN_Needle=20, MN_SF=34, WN_Needle=10, WN_SF=34) by contingency table analysis and logistic regression.

Contingency table

Due to the small sample sizes of some groups, there were several expected counts <5, why we opted for the Fisher's Exact test, which had the added benefit of producing exact p-value calculation. The analysis rendered a p-value of 0.00519, suggesting a statistically significant difference between groups. This was confirmed by the pairwise Fisher's Exact test corrected by the Benjamin-Hochberg method, although the only statistically significant difference was observed for well-nourished and malnourished sand fly infected mice (Appendix table 96).

Appendix Table 96

Pairwise Fisher's Exact test

group1	group2	n	estimate	p	conf.low	conf.high	alternative	p.adj	p.adj.signif
MN_Needle	MN_SF	54	2.7223	0.2910	0.4647	29.2690	two.sided	0.4360	ns
MN_Needle	WN_Needle	30	0.0000	0.5400	0.0000	10.8041	two.sided	0.6480	ns
MN_Needle	WN_SF	54	0.0000	0.1330	0.0000	3.0783	two.sided	0.3340	ns
MN_SF	WN_Needle	44	0.0000	0.1670	0.0000	1.8885	two.sided	0.3340	ns
MN_SF	WN_SF	68	0.0000	0.0049	0.0000	0.5050	two.sided	0.0295	*
WN_Needle	WN_SF	44	0.0000	1.0000	0.0000	Inf	two.sided	1.0000	ns

The observed odds ratios suggested that well-nourished BALB/c mice had a 0-fold likelihood of developing

a >=20% weight loss due to *L. donovani* infection, regardless of the infection route (Appendix table 97). Conversely, malnourished animals did develop the weight loss post infection. Although the malnourished mice infected by sand fly bite, did show a greater occurrence rate of >=20% weight loss compared to needle inoculated mice, that difference did not achieve statistical significance. The retrospective sample size and power calculations showed that our sample was sufficiently large enough at the total *N* to have sufficient power, the fact that the sample size per group were not equal (WN_needle only contained 10 mice) affected the actual test power (Appendix table 98). Either way, the contingency analysis suggested that "Diet", rather than infection "Route" was key in the occurrence of critical weight loss post infection.

Appendix Table 97

Odds Ratios

Groups	estimate	lower	upper	p.value
MN_Needle	1.0000	NA	NA	NA
MN_SF	2.7223	0.4647	29.2690	0.2912
WN_Needle	<0.0001	<0.0001	10.8041	0.5402
WN_SF	<0.0001	<0.0001	3.0783	0.1328

Appendix Table 98

Retrospective Power Calculation

	Calculation for	
Parameters	Sample size	Statistical power
Statistical power	0.8	**0.826**
Total n	**93**	98
Degrees of freedom	3	3
Non-centrality parameter	10.903	11.59
Type I error rate	0.05	0.05
Type II error rate	0.2	0.174

Logistic regression

Due to the lack of events in the well-nourished group, logistic regression was not possible here as it rendered nonsensical data due to its dependence on the maximum likelihood estimation, which rendered an infinite estimate under these circumstance.

Panel c

Here, we analyzed the occurrence of ocular pathology following *Leishmania donovani* infection in well-nourished (WN) and malnourished (MN) BALB/c mice. A total of *N*=98 BALB/c mice (WN_Needle=10, MN_Needle=20, MN_SF=34, WN_SF=34) were examined on a weekly bases post infection by either "needle" or sand fly (SF) route and occurrence of pathology was recorded as time-to-event data.

Survival analysis

The data was analyzed by the Mantel-Haenszel's log-rank test by use of the survdiff() function from the survival package in R. The test output is shown in appendix table 99, which was statistically significant (F(3)=95.73, <0.0001).

Appendix Table 99

Log-rank test

Groups	N	Observed	Expected	Chi-Square (O-E)^2/E	log-rank (O-E)^2/V
WN_Needle	10	0	3.8551	3.8551	4.5619
WN_SF	34	0	13.1074	13.1074	23.3213
MN_Needle	20	2	7.5019	4.0351	5.4924
MN_SF	34	30	7.5356	66.9693	95.3186

The pairwise Mantel-Haenszel' log-rank test, which was adjusted by the Benjamin-Hochberg correction, showed that the malnourished, sand fly inoculated group was statistically significant from all other groups, while there was no statistically significant difference between the remaining three groups (Appendix table 100).

Appendix Table 100

Pairwise Log-rank test

Groups	WN_Needle	WN_SF	MN_Needle
WN_SF	1.0000	NA	NA
MN_Needle	0.3733	0.0927	NA
MN_SF	<0.0001	<0.0001	<0.0001

In fact, only malnourished animals developed ocular pathology post infection and the grand majority of these were inoculated by sand fly (Appendix table 101), suggesting that sand fly bites significantly increased the occurrence of ocular pathology in malnourished hosts.

Appendix Table 101

Ocular pathology occurrence rate

	Well-nourished		Malnourished	
	Needle	Sand fly	Needle	Sand fly
Group N	10	34	20	34
Ocular Pathology	0	0	2	30

Occurrance Rate	0.00%	0.00%	10.00%	88.24%

Cox proportional hazards regression model

We also explored the data by Cox proportional hazards regression. Due to the lack of events in the well-nourished group, we had to resort to Firth's penalized maximum likelihood bias reduction method for Cox regression. The output showed that both predictors, "Diet" and "Route", were statistically significant (Appendix table 102).

Appendix Table 102

Firth's penalized maximum likelihood bias reduction method for Cox regression

Groups	coef	se(coef)	exp(coef)	lower 0.95	upper 0.95	Chisq	p	sig.
DietMN	4.89	1.47	133.59	18.59	16969.83	62.67	<0.0001	****
RouteSF	2.70	0.68	14.83	4.83	73.44	31.60	<0.0001	****

The odds ratios suggested that "Diet" was a much more potent predictor for ocular pathology, than "Route", although both were statistically significant. Malnourished mice were about 133.6-times more likely to develop ocular pathology than well-nourished mice compared to sand fly inoculated mice being 14.8-times more likely to developed ocular pathology compared to the once inoculated by needle (Appendix table 103).

Appendix Table 103

Odds ratios

Groups	AHR	2.5 %	97.5 %	p-value	sig.
DietMN	133.59	7.52	2373.94	<0.0001	****
RouteSF	14.83	3.89	56.51	<0.0001	****

The pairwise comparison clearly confirmed the observation of the log-rank test that the malnourished, sand fly inoculated group was statistically significantly different from the other three groups (Appendix table 104 and 105). Here, the analysis also separated well-nourished needle inoculated mice from the other three groups, which may be due to the small samples.

Appendix Table 104

Pariwise comparison by estimated marginal means

Groups	estimate	SE	df	z.ratio	p.value	sig.
WN Needle - MN Needle	-4.89	1.47	Inf	-3.33	0.0051	**
WN Needle - WN SF	-2.70	0.68	Inf	-3.95	0.0005	***
WN Needle - MN SF	-7.59	1.64	Inf	-4.63	<0.0001	****

MN Needle - WN SF	2.20	1.60	Inf	1.38	0.6697	ns
MN Needle - MN SF	-2.70	0.68	Inf	-3.95	0.0005	***
WN SF - MN SF	-4.89	1.47	Inf	-3.33	0.0051	**

Appendix Table 105

Pariwise comparison by estimated marginal means (letters)

Diet	Route	emmean	SE	df	asymp.LCL	asymp.UCL	.group
WN	Needle	0.00	0.00	Inf	0.00	0.00	a
WN	SF	2.70	0.68	Inf	1.00	4.40	b
MN	Needle	4.89	1.47	Inf	1.24	8.55	b
MN	SF	7.59	1.64	Inf	3.50	11.68	c

Panel d

Here, we present the parasite counts from several isolated tissues (brain, ears, eyes, liver, paw and spleen) according to qPCR as a measure of parasite dissemination to these tissues. To analyze the data, we had to re-scale it, due to the frequent occurrence of zero-values due to instances of no detection, by dividing all values by the smallest non-zero value in the dataset. This resulted in an approximate Poisson / negative binomial distribution, which allowed the convenient analysis of the re-scaled and rounded counts by the appropriate models for these distributions.

We analyzed varying total *N* of the different tissue samples (Appendix table 106). Total *N* and group *N* were dependent on available animals. While the spleen was always collected, other tissue types were only considered later in the study, as to why their total *N* is much lower. In general, a zero-inflated negative binomial model was the best fit our data, with the exception of eye and liver sample, where a zero-inflated Poisson and standard Poisson regression model fitted best, respectively. Model fit was assessed by overdispersion test and pseudo r^2.

Appendix Table 106

Summary information

		Group N			Selected model		Overdispersion test		
Tissue	Total N	MN_Needle	WN_SF	MN_SF	Type[1]	Model[2]	Ratio	p_value	Pseudo R^2
Brain	26	7	8	11	zero-inf.	NB	1.1272	0.6160	0.0578
Ear	38	11	12	15	zero-inf.	NB	2.1435	0.2400	0.3773
Eye	26	7	8	11	zero-inf.	Poisson	1.3351	0.4160	0.2482
Liver	26	7	8	11	standard	Poisson	1.0000	0.4608	0.3126
Paw	26	7	8	11	zero-inf.	NB	0.6729	0.5440	0.1567
Spleen	85	17	32	36	zero-inf.	NB	3.1538	0.0800	0.2736

[1]zero-inf. = zero-inflated
[2]NB = negative binomial

The summary of the best fitting models per tissue, according to dispersion test, rather than AIC in this case, is shown in appendix table 107. In general, malnourished, needle inoculated mice served as the reference sample in the regression analysis. Statistical significance was only observed for ear and spleen.

Appendix Table 107

Model summary outputs

Predictors	Estimate	Std. Error	z value	Pr(>|z|)	sig.
Brain					
(Intercept)	3.5699	0.7886	4.5270	<0.0001	****
DietMN_SF	-0.5966	0.9339	-0.6388	0.5230	ns
DietWN_SF	-1.3865	1.0766	-1.2879	0.1978	ns
Ear					
(Intercept)	1.6452	0.5816	2.8289	0.0047	**
DietMN_SF	1.7824	0.7586	2.3494	0.0188	*
DietWN_SF	4.0175	0.7953	5.0518	<0.0001	****
Eye					
(Intercept)	-20.8473	18851.5363	-0.0011	0.9991	ns
DietMN_SF	21.1547	18851.5363	0.0011	0.9991	ns
DietWN_SF	21.8020	18851.5363	0.0012	0.9991	ns
Liver					
(Intercept)	-19.3026	3562.9263	-0.0054	0.9957	ns
DietMN_SF	18.6964	3562.9263	0.0052	0.9958	ns
DietWN_SF	17.2231	3562.9264	0.0048	0.9961	ns
Paw					
(Intercept)	-0.1928	2.9165	-0.0661	0.9473	ns
DietMN_SF	3.3461	1.8853	1.7748	0.0759	+
DietWN_SF	-1.5465	2.2330	-0.6926	0.4886	ns
Spleen					
(Intercept)	0.8303	0.6791	1.2227	0.2214	ns
DietMN_SF	4.2820	0.8167	5.2431	<0.0001	****
DietWN_SF	0.4489	0.8374	0.5360	0.5919	ns

We applied a pairwise comparison based on the estimated marginal means for each tissue, respectively

(Appendix table 107 and 108). The analysis showed no statistically significant difference between groups for the tissues brain, eye and liver. For the paw, we observed a statistically significant difference between well-nourished and malnourished sand fly inoculated mice, but the result may not be reliable due to the small sample size. For the ear samples we observed statistically significant difference between all pairs with the well-nourished, sand fly inoculated mice clustering most strongly away from either malnourished group. In case of the spleen, it was the malnourished, sand fly inoculated group that was statistically significantly different from the other two groups, suggesting significantly higher and more frequent occurrence of parasites in spleen in this group.

Appendix Table 108

Pairwise comparison based on estimated marginal means

Predictor pairs	estimate	SE	df	z.ratio	p.value	sig.
Brain						
MN_Needle - MN_SF	0.5966	0.9339	Inf	0.6388	0.523	ns
MN_Needle - WN_SF	1.3865	1.0766	Inf	1.2879	0.523	ns
MN_SF - WN_SF	0.7899	0.9768	Inf	0.8087	0.523	ns
Ear						
MN_Needle - MN_SF	-1.7824	0.7586	Inf	-2.3494	0.0188	*
MN_Needle - WN_SF	-4.0175	0.7953	Inf	-5.0518	<0.0001	****
MN_SF - WN_SF	-2.2352	0.7291	Inf	-3.0657	0.0033	**
Eye						
MN_Needle - MN_SF	-21.1547	18851.5363	Inf	-0.0011	0.9991	ns
MN_Needle - WN_SF	-21.8020	18851.5363	Inf	-0.0012	0.9991	ns
MN_SF - WN_SF	-0.6473	0.6414	Inf	-1.0093	0.9385	ns
Liver						
MN_Needle - MN_SF	-18.6964	3562.9263	Inf	-0.0052	0.9961	ns
MN_Needle - WN_SF	-17.2231	3562.9264	Inf	-0.0048	0.9961	ns
MN_SF - WN_SF	1.4733	1.0801	Inf	1.3640	0.5177	ns
Paw						
MN_Needle - MN_SF	-3.3461	1.8853	Inf	-1.7748	0.1139	ns
MN_Needle - WN_SF	1.5465	2.2330	Inf	0.6926	0.4886	ns
MN_SF - WN_SF	4.8926	2.0285	Inf	2.4119	0.0476	*
Spleen						
MN_Needle - MN_SF	-4.2820	0.8167	Inf	-5.2431	<0.0001	****
MN_Needle - WN_SF	-0.4489	0.8374	Inf	-0.5360	0.5919	ns

MN_SF - WN_SF	3.8331	0.6678	Inf	5.7403	<0.0001	****

Appendix Table 109

Pairwise comparison letter code

Predictor levels	emmean	SE	df	asymp.LCL	asymp.UCL	.group
Brain						
WN_SF	2.1834	0.8822	Inf	0.0713	4.2954	a
MN_SF	2.9733	0.6029	Inf	1.5300	4.4166	a
MN_Needle	3.5699	0.7886	Inf	1.6820	5.4577	a
Ear						
MN_Needle	1.6452	0.5816	Inf	0.2529	3.0374	a
MN_SF	3.4275	0.4872	Inf	2.2613	4.5938	b
WN_SF	5.6627	0.5424	Inf	4.3641	6.9612	c
Eye						
MN_Needle	-20.8473	18851.5363	Inf	-45151.0445	45109.3499	a
MN_SF	0.3074	0.5689	Inf	-1.0544	1.6692	a
WN_SF	0.9547	0.3263	Inf	0.1735	1.7360	a
Liver						
MN_Needle	-19.3026	3562.9263	Inf	-8548.8761	8510.2709	a
WN_SF	-2.0794	1.0000	Inf	-4.4734	0.3145	a
MN_SF	-0.6061	0.4082	Inf	-1.5835	0.3712	a
Paw						
WN_SF	-1.7393	2.8074	Inf	-8.4601	4.9816	a
MN_Needle	-0.1928	2.9165	Inf	-7.1748	6.7893	ab
MN_SF	3.1533	2.8624	Inf	-3.6992	10.0059	b
Spleen						
MN_Needle	0.8303	0.6791	Inf	-0.7954	2.4561	a
WN_SF	1.2792	0.4900	Inf	0.1062	2.4522	a
MN_SF	5.1123	0.4537	Inf	4.0262	6.1984	b

Panel e

Data analysis

We analysed the frequency of *Leishmania donovani* dissemination to several different tissue sites (brain, ears, eyes, liver, paw and spleen) in a varying total *N* of well-nourished (WN) and malnourished (MN)

BALB/c mice infected intradermally either by “needle” injection or sand fly bite (SF) (Appendix table 110). We used contingency table analysis and logistic regression here. These are the same animals as presented in figure 3d. These analyses permitted looking at our data from another angle to fully comprehend the impact of infection route and state of nourishment on parasite dissemination.

Appendix Table 110

Sample size

Tissue	Total N	MN_Needle	MN_SF	WN_SF
Brain	26	7	11	8
Ear	38	11	15	12
Eye	26	7	11	8
Liver	26	7	11	8
Paw	26	7	11	8
Spleen	83	17	34	32

Contingency table

Due to the small sample sizes in most datasets, there were several expected counts <5, why we opted for the Fisher’s Exact test, which had the added benefit of exact p-value calculation, for all tissues except the spleen, where the sample size was much larger and a Chi-square test was applied. Only the spleen showed a statistical significant difference (Appendix table 111).

Appendix Table 111

Contingency table analyses

Tissue	n	p	p.signif	Test
Brain	26	0.5410	ns	Fisher’s Exact test
Ear	38	0.8870	ns	Fisher’s Exact test
Eye	26	0.1120	ns	Fisher’s Exact test
Liver	26	0.1750	ns	Fisher’s Exact test
Paw	26	0.8360	ns	Fisher’s Exact test
Spleen	83	0.0446	*	Chi-square test

Interestingly, the pairwise comparison did not show any statistical significant difference, even before adjusting p-values for multiple comparisons (Appendix table 112), suggesting that there is no statistically significant difference between groups within any tissue in terms of dissemination success.

Appendix Table 112

Pairwise comparison

group	n	estimate	p	p.adj	p.adj.signif
Brain - Fisher's Exact test					
MN_Needle - MN_SF	18	1.2926	1.0000	1.000	ns
MN_Needle - WN_SF	15	0.4753	0.6190	0.928	ns
MN_SF - WN_SF	19	0.3638	0.3700	0.928	ns
Liver - Fisher's Exact test					
MN_Needle - MN_SF	18	Inf	0.1190	0.357	ns
MN_Needle - WN_SF	15	Inf	1.0000	1.000	ns
MN_SF - WN_SF	19	0.2678	0.3380	0.507	ns
Paw - Fisher's Exact test					
MN_Needle - MN_SF	18	2.1565	1.0000	1.000	ns
MN_Needle - WN_SF	15	0.8660	1.0000	1.000	ns
MN_SF - WN_SF	19	0.3997	0.6030	1.000	ns
Spleen - Chi-square test					
MN_Needle - MN_SF	51	3.5406	0.0599	0.127	ns
MN_Needle - WN_SF	49	0.0630	0.8020	0.802	ns
MN_SF - WN_SF	66	2.9724	0.0847	0.127	ns
Ear - Fisher's Exact test					
MN_Needle - MN_SF	26	1.4763	1.0000	1.000	ns
MN_Needle - WN_SF	23	1.8241	0.6400	1.000	ns
MN_SF - WN_SF	27	1.2398	1.0000	1.000	ns
Eye - Fisher's Exact test					
MN_Needle - MN_SF	18	Inf	0.2450	0.368	ns
MN_Needle - WN_SF	15	Inf	0.0769	0.231	ns
MN_SF - WN_SF	19	2.5255	0.3770	0.377	ns

Conversely, when looking at the odds ratios, malnourished, sand fly inoculated mice were significantly more likely to experience parasite dissemination to the spleen than malnourished, needle inoculated mice, which served as the reference group in this analysis (Appendix table 113).

Appendix Table 113

Odds Ratios

Groups	estimate	lower	upper	p.value	sig.
Brain					
MN_Needle	1.0000	NA	NA	NA	NA

MN_SF	1.2926	0.1220	13.1405	1.0000	ns
WN_SF	0.4753	0.0362	5.2820	0.6193	ns
Ear					
MN_Needle	1.0000	NA	NA	NA	NA
MN_SF	1.4763	0.1562	14.0607	1.0000	ns
WN_SF	1.8241	0.1648	26.9593	0.6404	ns
Eye					
MN_Needle	1.0000	NA	NA	NA	NA
MN_SF	Inf	0.2687	Inf	0.2451	ns
WN_SF	Inf	0.6991	Inf	0.0769	+
Liver					
MN_Needle	1.0000	NA	NA	NA	NA
MN_SF	Inf	0.4574	Inf	0.1193	ns
WN_SF	Inf	0.0225	Inf	1.0000	ns
Paw					
MN_Needle	1.0000	NA	NA	NA	NA
MN_SF	2.1565	0.1311	137.3685	1.0000	ns
WN_SF	0.8660	0.0096	78.3189	1.0000	ns
Spleen					
MN_Needle	1.0000	NA	NA	NA	NA
MN_SF	3.7196	1.0912	14.4367	0.0399	*
WN_SF	1.4147	0.4021	5.5178	0.7543	ns

Applying a retrospective statistical power calculation showed that the sample size for most tissues was too small to detect a statistical difference difference and thus, our statistical power was well below the standard 80% for all tissues (Appendix table 114). This observation suggested that the lack of observing statistical significance may be due to type II errors. However, larger sample sizes were prohibitive due to cost and loss of life.

Appendix Table 114

Retrospective Power Calculation

	Calculation for	
Parameters	Sample size	Statistical power
Brain		
Statistical power	0.8	**0.161**

Total n	**191**	26
Degrees of freedom	2	2
Non-centrality parameter	9.635	1.315
Type I error rate	0.05	0.05
Type II error rate	0.2	0.839
Ear		
Statistical power	0.8	**0.082**
Total n	**905**	38
Degrees of freedom	2	2
Non-centrality parameter	9.635	0.405
Type I error rate	0.05	0.05
Type II error rate	0.2	0.918
Eye		
Statistical power	0.8	**0.482**
Total n	**53**	26
Degrees of freedom	2	2
Non-centrality parameter	9.635	4.745
Type I error rate	0.05	0.05
Type II error rate	0.2	0.518
Liver		
Statistical power	0.8	**0.413**
Total n	**63**	26
Degrees of freedom	2	2
Non-centrality parameter	9.635	3.979
Type I error rate	0.05	0.05
Type II error rate	0.2	0.587
Paw		
Statistical power	0.8	**0.115**
Total n	**313**	26
Degrees of freedom	2	2
Non-centrality parameter	9.635	0.802
Type I error rate	0.05	0.05
Type II error rate	0.2	0.885
Spleen		
Statistical power	0.8	**0.6**
Total n	**129**	83
Degrees of freedom	2	2

Non-centrality parameter	9.635	6.219
Type I error rate	0.05	0.05
Type II error rate	0.2	0.4

The problem of the small and unequal sample sizes between groups became evident when we looked at the contingency tables for each tissue (Appendix table 115). Small counts in most cells made these analyses not very robust. However, looking at the contingency tables it can be observed that malnourished, sand fly inoculated mice experienced more frequently dissemination of parasites to other tissues than well-nourished, sand fly inoculated or malnourished, needle inoculated mice. The latter had generally the lowest frequency of dissemination events, suggesting that a) parasite inoculation by sand fly bite increased the frequency of parasite dissemination, and that b) in the context of sand fly inoculation, malnourishment further exacerbate the frequency of parasite dissemination.

Appendix Table 115
Odds Ratios

Dissemination	MN_Needle	MN_SF	WN_SF
Brain			
NO	3	4	5
YES	**4**	**7**	**3**
Ear			
NO	3	3	2
YES	**8**	**12**	**10**
Eye			
NO	7	8	4
YES	**0**	**3**	**4**
Liver			
NO	7	7	7
YES	**0**	**4**	**1**
Paw			
NO	6	8	7
YES	**1**	**3**	**1**
Spleen			
NO	12	13	20
YES	**5**	**21**	**12**

Logistic regression

We applied a logistic regression model to the same data and assessed the two predictor variables "Diet" and "Route" without an interaction term to assess individual predictor contribution to the outcome. The data output showed that infection by sand fly bite ("Route") was statistically significant compared to needle inoculation only for the spleen. Malnourishment was close to being statistically significantly different from well-nourishment for the spleen, too. For all other tissues, there was no statistical significance observed (Appendix table 116).

Appendix Table 116

Logistic regression output

Groups	Estimate	lower CI	upper CI	Std. Error	partial.R2	z value	Pr(>|z|)	sig.
Brain								
(Intercept)	-0.7828	-3.2570	1.6385	1.2286	0.0000	-0.6371	0.5241	ns
DietMN	1.0704	-0.7742	3.0761	0.9624	0.0357	1.1123	0.2660	ns
RouteSF	0.2719	-1.7200	2.2484	0.9880	0.0022	0.2752	0.7831	ns
Ear								
(Intercept)	1.2040	-1.1368	3.7552	1.2145	0.0000	0.9913	0.3215	ns
DietMN	-0.2231	-2.3937	1.7544	1.0083	0.0013	-0.2213	0.8249	ns
RouteSF	0.4055	-1.4841	2.3056	0.9354	0.0048	0.4335	0.6647	ns
Eye								
(Intercept)	-17.5852	NA	208.3362	2465.3259	0.0000	-0.0071	0.9943	ns
DietMN	-0.9808	-3.0079	0.9169	0.9789	0.0411	-1.0019	0.3164	ns
RouteSF	17.5852	-313.1974	NA	2465.3258	0.1219	0.0071	0.9943	ns
Liver								
(Intercept)	-20.9524	NA	412.3356	4064.6350	0.0000	-0.0052	0.9959	ns
DietMN	1.3863	-0.8113	4.4936	1.2392	0.0663	1.1187	0.2633	ns
RouteSF	19.0065	-457.9217	NA	4064.6349	0.1852	0.0047	0.9963	ns
Paw								
(Intercept)	-2.7568	-6.6469	0.2147	1.6637	0.0000	-1.6571	0.0975	+
DietMN	0.9651	-1.3351	4.0962	1.2654	0.0252	0.7627	0.4457	ns
RouteSF	0.8109	-1.5137	3.9522	1.2748	0.0174	0.6361	0.5247	ns
Spleen								
(Intercept)	-1.8659	-3.3704	-0.4616	0.7357	0.0000	-2.5363	0.0112	*
DietMN	0.9904	0.0101	2.0112	0.5078	0.0350	1.9503	0.0511	+
RouteSF	1.3550	0.1458	2.6835	0.6387	0.0429	2.1217	0.0339	*

The odds ratios were nonsensical for eye and liver here (Appendix table 117), just as they had been for the contingency table analysis above (Appendix table 113). Looking at the spleen, the infection route had a bigger impact on parasite dissemination than diet, although either odds ratio was statistically significant according to the 95% confidence intervals. For all other tissues, we did not obtain statistical significance.

Appendix Table 117

Odds Ratios

Groups	OR	2.5 %	97.5 %	Effect_size
Brain				
(Intercept)	0.457	0.038	5.147	small
DietMN	2.917	0.461	21.675	small
RouteSF	1.312	0.179	9.473	very small
Ear				
(Intercept)	3.333	0.321	42.743	small
DietMN	0.800	0.091	5.780	very small
RouteSF	1.500	0.227	10.031	very small
Eye				
(Intercept)	0.000	NA	3.01e+90	large
DietMN	0.375	0.049	2.502	small
RouteSF	4.34e+07	0.000	NA	large
Liver				
(Intercept)	0.000	NA	1.19e+179	large
DietMN	4.000	0.444	89.442	medium
RouteSF	1.80e+08	0.000	NA	large
Paw				
(Intercept)	0.064	0.001	1.240	large
DietMN	2.625	0.263	60.113	small
RouteSF	2.250	0.220	52.048	small
Spleen				
(Intercept)	0.155	0.034	0.630	medium
DietMN	2.692	1.010	7.472	small
RouteSF	3.877	1.157	14.636	medium

The Wald test confirmed the logistic regression result, stating that only the infection route was statistically significant for the spleen, with “Diet” close to reaching statistical significance (Appendix table 118).

Appendix Table 118

Wald test

Predictor	chi2	df	P	sig.
Brain				
Diet	1.2372	1	0.2660	ns
Route	0.0758	1	0.7831	ns
Ear				
Diet	0.0490	1	0.8249	ns
Route	0.1879	1	0.6647	ns
Eye				
Diet	1.0039	1	0.3164	ns
Route	<0.0001	1	0.9943	ns
Liver				
Diet	1.2514	1	0.2633	ns
Route	<0.0001	1	0.9963	ns
Paw				
Diet	0.5817	1	0.4457	ns
Route	0.4047	1	0.5247	ns
Spleen				
Diet	3.8037	1	0.0511	+
Route	4.5017	1	0.0339	*

However, although, in general, there was no statistically significance in the logistic regression model with the exception of the infection route in the spleen that did not mean that there was no meaningful biological effect. A retrospective sample size and power calculation with the study data showed that the study was well underpowered for the logistic regression for all tissues (Appendix table 119), as it had already been the case for the contingency table analysis (Appendix table 114), suggesting that the lack of statistical significance may be due to type II errors. But larger sample sizes as indicated by the sample size calculation, were prohibitive due to cost and loss of life.

Appendix Table 119

Retrospective power analyses

Predictor	Calculation	Beta0	Beta1	R-square	alpha	Power	TotalN	NCP	Alternative
Brain									

Diet	Sample_size	0.154	1.070	0.002	0.05	0.80	**130**	2.769	not equal
Route	Sample_size	0.154	0.272	0.036	0.05	0.80	**1805**	2.800	not equal
Diet	Power	-0.783	1.070	0.002	0.05	**0.25**	26	1.305	not equal
Route	Power	-0.783	0.272	0.036	0.05	**0.06**	26	0.323	not equal
Ear									
Diet	Sample_size	1.322	-0.223	0.005	0.05	0.80	**3590**	-2.799	not equal
Route	Sample_size	1.322	0.405	0.001	0.05	0.80	**1314**	2.793	not equal
Diet	Power	1.204	-0.223	0.005	0.05	**0.06**	38	-0.297	not equal
Route	Power	1.204	0.405	0.001	0.05	**0.08**	38	0.493	not equal
Eye									
Diet	Sample_size	-0.999	-0.981	0.122	0.05	0.80	**260**	-2.758	not equal
Route	Sample_size	-0.999	17.585	0.041	0.05	0.80	**563119**	2.286	not equal
Diet	Power	-17.585	-0.981	0.122	0.05	**0.03**	26	0.000	not equal
Route	Power	-17.585	17.585	0.041	0.05	**0.00**	26	0.009	not equal
Liver									
Diet	Sample_size	-1.435	1.386	0.185	0.05	0.80	**101**	2.747	not equal
Route	Sample_size	-1.435	19.006	0.066	0.05	0.80	**1325232**	2.286	not equal
Diet	Power	-20.952	1.386	0.185	0.05	**0.02**	26	0.000	not equal
Route	Power	-20.952	19.006	0.066	0.05	**0.00**	26	0.002	not equal
Paw									
Diet	Sample_size	-1.435	0.965	0.017	0.05	0.80	**179**	2.770	not equal
Route	Sample_size	-1.435	0.811	0.025	0.05	0.80	**261**	2.778	not equal
Diet	Power	-2.757	0.965	0.017	0.05	**0.09**	26	0.677	not equal
Route	Power	-2.757	0.811	0.025	0.05	**0.07**	26	0.556	not equal
Spleen									
Diet	Sample_size	-0.169	0.990	0.043	0.05	0.80	**144**	2.779	not equal
Route	Sample_size	-0.169	1.355	0.035	0.05	0.80	**82**	2.756	not equal
Diet	Power	-1.866	0.990	0.043	0.05	**0.39**	83	1.703	not equal
Route	Power	-1.866	1.355	0.035	0.05	**0.68**	83	2.389	not equal

Even so, there was a good indication of potential biological significance. Considering the predicted probability of parasite dissemination for most tissues, it can be seen that being malnourished and inoculated by a sand fly increased the probability of parasite dissemination for most tissues beyond the other conditions (Appendix table 120). With exception of the spleen, the large confidence intervals did not render statistical significance in all other tissues, which did not exclude biological significance, though.

Appendix Table 120

Predicted probability of parasite dissemination

Factors	Diet	Route	fit	se.fit	Predicted_Probability	lower_CI	upper_CI
Brain							
1	WN	SF	-0.5108	0.7303	**0.3750**	0.1254	0.7152
2	MN	Needle	0.2877	0.7638	**0.5714**	0.2298	0.8563
3	MN	SF	0.5596	0.6268	**0.6364**	0.3387	0.8567
Ear							
1	WN	SF	1.6094	0.7746	**0.8333**	0.5228	0.9580
2	MN	Needle	0.9808	0.6770	**0.7273**	0.4143	0.9095
3	MN	SF	1.3863	0.6455	**0.8000**	0.5302	0.9341
Eye							
1	WN	SF	0.0000	0.7071	**0.5000**	0.2001	0.7999
2	MN	Needle	-18.5661	2465.3257	**0.0000**	0.0000	1.0000
3	MN	SF	-0.9808	0.6770	**0.2727**	0.0905	0.5857
Liver							
1	WN	SF	-1.9459	1.0690	**0.1250**	0.0173	0.5373
2	MN	Needle	-19.5661	4064.6348	**0.0000**	0.0000	1.0000
3	MN	SF	-0.5596	0.6268	**0.3636**	0.1433	0.6613
Paw							
1	WN	SF	-1.9459	1.0690	**0.1250**	0.0173	0.5373
2	MN	Needle	-1.7918	1.0801	**0.1429**	0.0197	0.5806
3	MN	SF	-0.9808	0.6770	**0.2727**	0.0905	0.5857
Spleen							
1	WN	SF	-0.5108	0.3651	**0.3750**	0.2268	0.5510
2	MN	Needle	-0.8755	0.5323	**0.2941**	0.1280	0.5419
3	MN	SF	0.4796	0.3529	**0.6176**	0.4472	0.7634

Figure 4

Panel a

Here, we analyzed the proportion of necrophiliacs, monocytes and lymphocytes in terminal bleeds from in well-nourished (WN) and malnourished (MN) BALB/c mice infected with *Leishmania donovani* parasite via sand fly bites (SF) for up to 30 weeks or not. We analyzed a total of N=40 BALB/c mice. The different groups had varying sample sizes (Appendix table 121). For the data analysis, we tested Poisson and

negative binomial regression models of the normalized cell counts, or beta regression after conversion of percentiles to ratios. Based on the Akaike information criterion (AIC), we selected a beta_regression model for the data analysis post data conversion. The model fit of the data was reasonable producing no statistically significant departure from 1 for its dispersion ratio and producing reasonable pseudo-R^2 values of all three cell groups (Appendix table 121).

Appendix Table 121

Summary information

Cells	Total N	MN_Ctrl	WN_Ctrl	MN_SF	WN_SF	Dispersion Ratio	Pseudo R^2
Lymphocytes	40	12	10	10	8	1.1012	0.5751
Monocytes	40	12	10	10	8	1.2556	0.5175
Neutrophils	40	12	10	10	8	1.1267	0.5146
Other	40	12	10	10	8	1.2062	0.1610

The model output showed that infection "Route" was always a statistically significant predictor for all three cell types, while the nutritional state of the mice had a statistical significant effect only for monocytes (Appendix table 122). The interaction between "Route" and "Diet" was statistically significant for lymphocytes and monocytes, but not for neutrophils. Please, note that "other" referred to the small reminder of detected blood cells that were not accounted for.

Appendix Table 122

Beta regression model output

Factors	Estimate	Std. Error	z value	Pr(>|z|)	sig.
Lymphocytes					
(Intercept)	0.8708	0.1195	7.2865	<0.0001	****
DietWN	0.2216	0.1814	1.2216	0.2219	ns
RouteSF	-0.9575	0.1695	-5.6489	<0.0001	****
DietWN:RouteSF	0.7197	0.2618	2.7486	0.0060	**
Monocytes					
(Intercept)	-5.0620	0.2985	-16.9568	<0.0001	****
DietWN	1.2948	0.3442	3.7624	0.0002	***
RouteSF	2.1254	0.3241	6.5577	<0.0001	****
DietWN:RouteSF	-1.9161	0.4384	-4.3709	<0.0001	****
Neutrophils					
(Intercept)	-1.0850	0.1289	-8.4149	<0.0001	****
DietWN	-0.3793	0.2010	-1.8871	0.0591	+
RouteSF	0.7821	0.1796	4.3559	<0.0001	****

DietWN:RouteSF	-0.3838	0.2838	-1.3520	0.1764	ns
Other					
(Intercept)	-3.1699	0.1470	-21.5603	<0.0001	****
DietWN	0.0428	0.2119	0.2020	0.8399	ns
RouteSF	-0.2999	0.2288	-1.3108	0.1899	ns
DietWN:RouteSF	-0.2699	0.3507	-0.7696	0.4415	ns

The likelihood ratio test confirmed that the inclusion of both predictors in the model was statistically significant for all three cell types (Appendix table 123).

Appendix Table 123

Likelihood ratio test against null model

#Df	LogLik	Df	Chisq	Pr(>Chisq)	sig.
Lymphocytes					
5	41.0899	NA	NA	NA	?
2	24.5001	-3	33.1796	<0.0001	****
Monocytes					
5	125.8189	NA	NA	NA	?
2	107.8980	-3	35.8418	<0.0001	****
Neutrophils					
5	42.3118	NA	NA	NA	?
2	28.9977	-3	26.6283	<0.0001	****
Other					
5	106.1766	NA	NA	NA	?
2	103.0245	-3	6.3041	0.0977	+

The pairwise comparison was based on the estimated marginal means. For lymphocytes, it showed that malnourished, sand fly inoculated mice had statistically significantly less lymphocytes in the blood than the three other groups (Appendix table 124 & Appendix table 125). For monocytes, the pairwise comparison showed that malnourished control animals had statistically significantly less monocytes in circulation. Conversely, after infection by sand fly bite, malnourished mice had statistically significantly more monocytes in circulation. For neutrophils, we observed statistically significantly more neutrophils in circulation in malnourished, sand fly inoculated mice compared to the other three groups.

Appendix Table 124

Pairwise comparison based on estimated marginal means

Predictor pairs	estimate	SE	df	z.ratio	p.value	sig.
Lymphocytes						
MN Ctrl - WN Ctrl	-0.0439	0.0357	Inf	-1.2295	0.2827	ns
MN Ctrl - MN SF	0.2266	0.0390	Inf	5.8162	<0.0001	****
WN Ctrl - WN SF	0.0473	0.0399	Inf	1.1862	0.2827	ns
MN SF - WN SF	-0.2232	0.0428	Inf	-5.2151	<0.0001	****
Monocytes						
MN Ctrl - WN Ctrl	-0.0163	0.0049	Inf	-3.3427	0.0017	**
MN Ctrl - MN SF	-0.0441	0.0075	Inf	-5.8959	<0.0001	****
WN Ctrl - WN SF	-0.0051	0.0074	Inf	-0.6920	0.4890	ns
MN SF - WN SF	0.0227	0.0093	Inf	2.4337	0.0179	*
Neutrophils						
MN Ctrl - WN Ctrl	0.0648	0.0338	Inf	1.9150	0.0832	+
MN Ctrl - MN SF	-0.1723	0.0391	Inf	-4.4110	<0.0001	****
WN Ctrl - WN SF	-0.0684	0.0380	Inf	-1.7977	0.0867	+
MN SF - WN SF	0.1687	0.0428	Inf	3.9445	0.0002	***
Other						
MN Ctrl - WN Ctrl	-0.0017	0.0084	Inf	-0.2016	0.8402	ns
MN Ctrl - MN SF	0.0101	0.0076	Inf	1.3292	0.2757	ns
WN Ctrl - WN SF	0.0178	0.0081	Inf	2.2079	0.0988	+
MN SF - WN SF	0.0060	0.0073	Inf	0.8227	0.4928	ns

Appendix Table 125

Pairwise comparison letter code

Diet	Route	emmean	SE	df	asymp.LCL	asymp.UCL	.group
Lymphocytes							
MN	SF	0.4783	0.0300	Inf	0.4034	0.5532	a
WN	SF	0.7015	0.0305	Inf	0.6253	0.7778	b
MN	Ctrl	0.7049	0.0249	Inf	0.6428	0.7670	b
WN	Ctrl	0.7488	0.0258	Inf	0.6844	0.8132	b
Monocytes							
MN	Ctrl	0.0063	0.0019	Inf	0.0016	0.0110	a

WN	Ctrl	0.0226	0.0048	Inf	0.0107	0.0345	b
WN	SF	0.0277	0.0060	Inf	0.0128	0.0426	b
MN	SF	0.0504	0.0074	Inf	0.0319	0.0689	c
Neutrophils							
WN	Ctrl	0.1878	0.0237	Inf	0.1286	0.2471	a
MN	Ctrl	0.2526	0.0243	Inf	0.1918	0.3134	a
WN	SF	0.2562	0.0299	Inf	0.1814	0.3309	a
MN	SF	0.4249	0.0306	Inf	0.3484	0.5013	b
Other							
WN	SF	0.0242	0.0052	Inf	0.0112	0.0372	a
MN	SF	0.0302	0.0053	Inf	0.0169	0.0434	a
MN	Ctrl	0.0403	0.0057	Inf	0.0261	0.0545	a
WN	Ctrl	0.0420	0.0063	Inf	0.0261	0.0579	a

Panel b

Data analysis

Here, we present the serum chemistry analysis of a total of N=35 well-nourished (WN) or malnourished (MN) BALB/c mice infected by sand fly bite (SF) or not (Ctrl) (Appendix table 126). A total of 8 targets were measured (alanine aminotransferase (ALT), albumin (ALB), alkaline phosphatase (ALP), amylase (AMY), Globulin (GLOB), Glucose (GLU), total protein (TP) & urea nitrogen (BUN)) per mouse.

Appendix Table 126

Summary information

Targets	Total N	WN_Ctrl	MN_Ctrl	WN_SF	MN_SF
alanine aminotransferase (ALT)	35	7	6	10	12
albumin (ALB)	35	7	6	10	12
alkaline phosphatase (ALP)	35	7	6	10	12
amylase (AMY)	35	7	6	10	12
Globulin (GLOB)	35	7	6	10	12
Glucose (GLU)	35	7	6	10	12
total protein (TP)	35	7	6	10	12
urea nitrogen (BUN)	35	7	6	10	12

Appendix Table 126

Summary information (continued)

Targets	Transformation
alanine aminotransferase (ALT)	Box-Cox power Transformed
albumin (ALB)	untransformed
alkaline phosphatase (ALP)	Box-Cox power Transformed
amylase (AMY)	Box-Cox power Transformed
Globulin (GLOB)	Box-Cox power Transformed
Glucose (GLU)	untransformed
total protein (TP)	untransformed
urea nitrogen (BUN)	untransformed

We needed to analyze the data with a two-way approach to account for the two predictors, "Diet" and "Route", both of which were between-subject factors.

For a two-way ANOVA, we had to assess the data for compliance with assumptions:

- Data normality
- Homogeneity of variance
- No significant outliers

Assumption analyses

Data normality

The assessment of the untransformed data distribution for each group was conducted by Shapiro-Wilks test and QQ-plot after splitting the data by both predictors. Note that all groups consisted of <30 data points (Appendix table 126), which made groups too small to assess data distribution reliably by Shapiro-Wilks test. We executed the test anyway as an indicator of gross departure of data normality. Thus, we performed the analyses by Shapiro-Wilks test (Appendix table 127) and QQ-plots (Fig.S1a-1). We found occasional departure of normality, which could not all be remedied by data transformation.

Appendix Table 127

Univariate Shapiro-Wilks test results

Diet	Route	variable	statistic	p	Outcome
alanine aminotransferase (ALT)					
WN	Ctrl	Counts	0.7818	0.0269	sig.
WN	SF	Counts	0.9528	0.7013	ns
MN	Ctrl	Counts	0.8360	0.1207	ns
MN	SF	Counts	0.8928	0.1280	ns
albumin (ALB)					
WN	Ctrl	Counts	0.9666	0.8733	ns

WN	SF	Counts	0.9584	0.7680	ns
MN	Ctrl	Counts	0.9380	0.6433	ns
MN	SF	Counts	0.9607	0.7937	ns
alkaline phosphatase (ALP)					
WN	Ctrl	Counts	0.9451	0.6854	ns
WN	SF	Counts	0.8895	0.1673	ns
MN	Ctrl	Counts	0.8065	0.0672	ns
MN	SF	Counts	0.9450	0.5652	ns
amylase (AMY)					
WN	Ctrl	Counts	0.9726	0.9167	ns
WN	SF	Counts	0.9436	0.5932	ns
MN	Ctrl	Counts	0.8452	0.1438	ns
MN	SF	Counts	0.8068	0.0112	sig.
Globulin (GLOB)					
WN	Ctrl	Counts	0.6004	0.0003	sig.
WN	SF	Counts	0.7753	0.0073	sig.
MN	Ctrl	Counts	0.7315	0.0130	sig.
MN	SF	Counts	0.8712	0.0677	ns
Glucose (GLU)					
WN	Ctrl	Counts	0.8510	0.1256	ns
WN	SF	Counts	0.9041	0.2431	ns
MN	Ctrl	Counts	0.9287	0.5699	ns
MN	SF	Counts	0.8833	0.0966	ns
total protein (TP)					
WN	Ctrl	Counts	0.8936	0.2939	ns
WN	SF	Counts	0.9169	0.3319	ns
MN	Ctrl	Counts	0.9999	1.0000	ns
MN	SF	Counts	0.9164	0.2578	ns
urea nitrogen (BUN)					
WN	Ctrl	Counts	0.9630	0.8441	ns
WN	SF	Counts	0.9304	0.4519	ns
MN	Ctrl	Counts	0.9182	0.4928	ns
MN	SF	Counts	0.8974	0.1469	ns

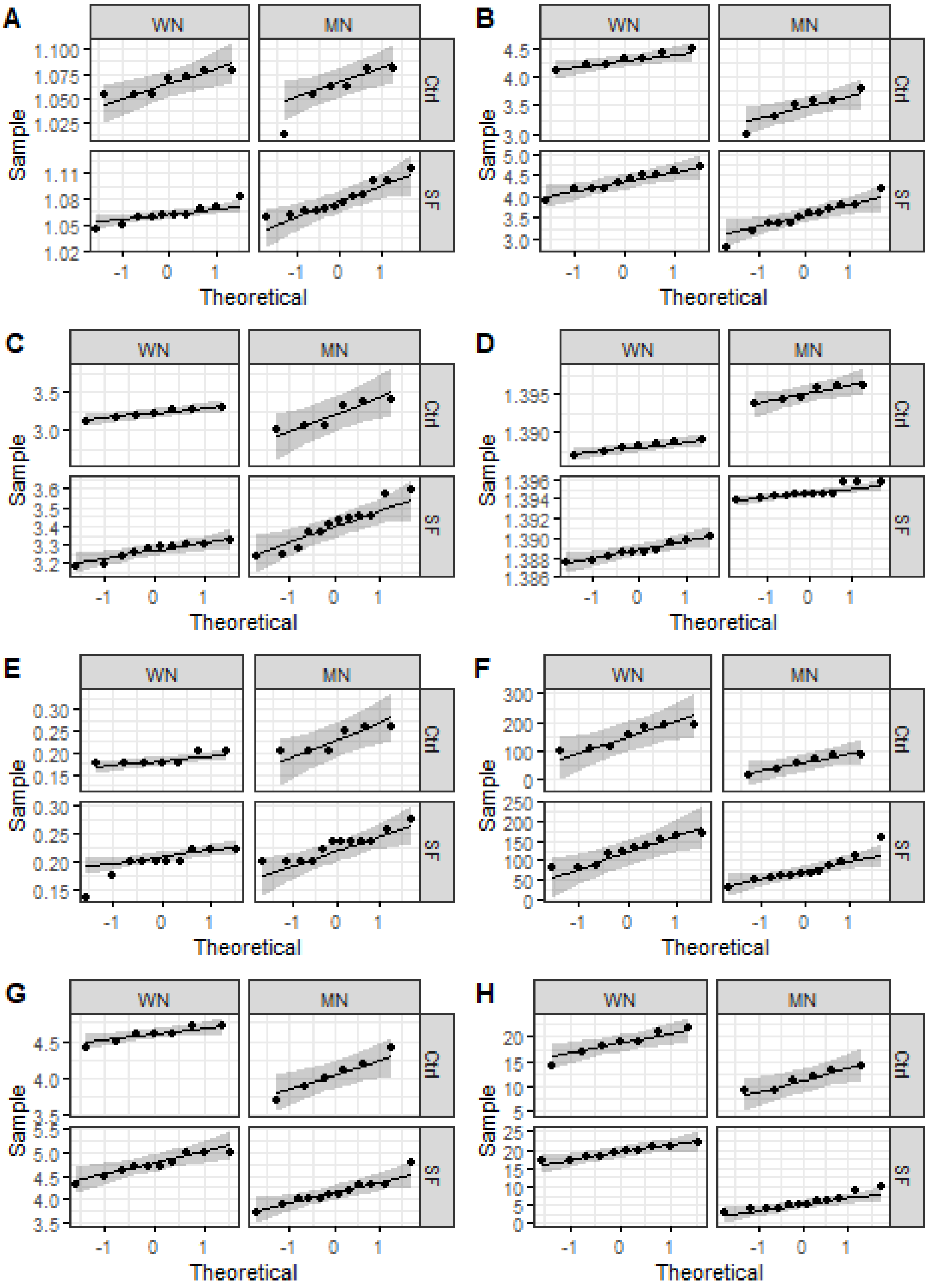

Fig.4b-1: QQ-plots of serum chemistry data: A) alanine aminotransferase (ALT), B) albumin (ALB), C) alkaline phosphatase (ALP), D) amylase (AMY), E) Globulin (GLOB), F) Glucose (GLU), G) total protein (TP) and H) urea nitrogen (BUN)

Homogeneity of variance

The assessment of homogeneity of variance was conducted by Levene's test for the dataset. The analysis output showed that assumption of homogeneity between groups held all targets accept for alkaline phosphatase (Appendix table 128).

Appendix Table 128

Assessment of homogeneity of variance by target

Targets	df1	df2	statistic	p	sig.
alanine aminotransferase (ALT)	3	31	1.1073	0.3610	ns
albumin (ALB)	3	31	1.0760	0.3736	ns
alkaline phosphatase (ALP)	3	31	8.5139	0.0003	***
amylase (AMY)	3	31	1.2940	0.2939	ns
Globulin (GLOB)	3	31	1.4187	0.2560	ns
Glucose (GLU)	3	31	0.6033	0.6178	ns
total protein (TP)	3	31	0.9974	0.4070	ns
urea nitrogen (BUN)	3	31	0.1293	0.9419	ns

Outliers

Conversely, we detected outliers for all targets except for alkaline phosphatase (Appendix table 129). Only one of these outliers was classed as extreme (Globulin (GLOB)).

Appendix Table 129

List of possible outliers

Diet	Route	Tissue	is.outlier	is.extreme
alanine aminotransferase (ALT)				
MN	Ctrl	alanine aminotransferase (ALT)	TRUE	FALSE
WN	SF	alanine aminotransferase (ALT)	TRUE	FALSE
WN	SF	alanine aminotransferase (ALT)	TRUE	FALSE
albumin (ALB)				
MN	SF	albumin (ALB)	TRUE	FALSE
amylase (AMY)				
MN	SF	amylase (AMY)	TRUE	FALSE
MN	SF	amylase (AMY)	TRUE	FALSE
Globulin (GLOB)				
WN	SF	Globulin (GLOB)	TRUE	FALSE

WN	SF	Globulin (GLOB)	TRUE	TRUE
Glucose (GLU)				
MN	SF	Glucose (GLU)	TRUE	FALSE
total protein (TP)				
MN	SF	total protein (TP)	TRUE	FALSE
urea nitrogen (BUN)				
MN	SF	urea nitrogen (BUN)	TRUE	FALSE

Two-way analysis

Based on the assumption analysis, we applied appropriate two-way tests for each target (Appendix table 130).

Appendix Table 130

Applied tests

Targets	Two-way test	One-way test
alanine aminotransferase (ALT)	Robust two-way ANOVA	Robust one-way ANOVA
albumin (ALB)	Standard two-way ANOVA	Standard one-way ANOVA
alkaline phosphatase (ALP)	Robust two-way ANOVA	Robust one-way ANOVA
amylase (AMY)	Robust two-way ANOVA	Robust one-way ANOVA
Globulin (GLOB)	Simple linear regression	Not applicable for linear regression
Glucose (GLU)	Standard two-way ANOVA	Standard one-way ANOVA
total protein (TP)	Standard two-way ANOVA	Standard one-way ANOVA
urea nitrogen (BUN)	Standard two-way ANOVA	Standard one-way ANOVA

Appendix Table 130

Applied tests (continued)

Targets	Pairwise
alanine aminotransferase (ALT)	Linear contrast expression
albumin (ALB)	Estimated marginal means analysis
alkaline phosphatase (ALP)	Linear contrast expression
amylase (AMY)	Linear contrast expression
Globulin (GLOB)	Estimated marginal means analysis
Glucose (GLU)	Estimated marginal means analysis
total protein (TP)	Estimated marginal means analysis
urea nitrogen (BUN)	Estimated marginal means analysis

The respective two-way analyses showed that there was no statistical significant differences detected for alanine aminotransferase and alkaline phosphatase (Appendix table 131). All other targets had detectable statistical significant differences for "Diet", suggesting that any detected significant difference detected in target levels were due to the nutritional status of the mice rather than the infection "Route". Only urea nitrogen showed statistical significance for "Route" and the interaction term, suggesting that both predictor were relevant to the detected levels of this target.

Appendix Table 131

Two-way analysis

Effect	DFn	DFd	Statistic	p.value	p<.05	ges
alanine aminotransferase (ALT)						
Diet	1	31	1.2224	0.2910		NA
Route	1	31	0.3431	0.5690		NA
Diet:Route	1	31	1.6730	0.2200		NA
albumin (ALB)						
Diet	1	31	77.1180	<0.0001	*	0.713
Route	1	31	0.4620	0.5020		0.015
Diet:Route	1	31	0.0002	0.9900		0.000
alkaline phosphatase (ALP)						
Diet	1	31	0.7158	0.4390		NA
Route	1	31	4.3699	0.0860	+	NA
Diet:Route	1	31	1.5655	0.2680		NA
amylase (AMY)						
Diet	1	31	288.2789	0.0010	***	NA
Route	1	31	0.0104	0.9220		NA
Diet:Route	1	31	1.8554	0.2040		NA
Globulin (GLOB)						
Diet	1	31	20.7636	0.0001	****	NA
Route	1	31	1.0594	0.3113		NA
Diet:Route	1	31	0.8660	0.3593		NA
Glucose (GLU)						
Diet	1	31	29.2640	<0.0001	*	0.486
Route	1	31	0.0540	0.8170		0.002
Diet:Route	1	31	3.2070	0.0830		0.094
total protein (TP)						

Diet	1	31	52.0380	<0.0001	*	0.627
Route	1	31	2.1060	0.1570		0.064
Diet:Route	1	31	0.1040	0.7490		0.003
urea nitrogen (BUN)						
Diet	1	31	243.9170	<0.0001	*	0.887
Route	1	31	10.5640	0.0030	*	0.254
Diet:Route	1	31	18.3990	0.0002	*	0.372

For the analysis of the simple main effect for each respective between-subject factor for each target, we performed one-way analyses with the data split by the predictor that was not used within the function. The results showed that statistical significance for each target, including those that had not shown statistical significance in the two-way analysis (Appendix table 132). Again all samples showed statistical significant differences for the comparison of well-nourished and malnourished mice for both control and sand fly infected ones. Only alkaline phosphatase and urea nitrogen also showed statistical significance for the comparison of control vs sand fly infected mice, but only for the malnourished mice.

Appendix Table 132

Simple main effect analysis

Factor	Effect	statistic	df1	df2	p.value	Sig.	effsize
alanine aminotransferase (ALT)							
WN	Route	0.8436	1	11.0300	0.3780	ns	0.3520
MN	Route	2.5191	1	7.9018	0.1516	ns	0.6778
Ctrl	Diet	0.4190	1	6.5740	0.5394	ns	0.3388
SF	Diet	6.6157	1	13.2865	0.0229	*	0.7166
albumin (ALB)							
WN	Route	0.2260	1	31.0000	0.6380	ns	0.0070
MN	Route	0.2360	1	31.0000	0.6300	ns	0.0080
Ctrl	Diet	28.7920	1	31.0000	<0.0001	*	0.4820
SF	Diet	48.3270	1	31.0000	<0.0001	*	0.6090
alkaline phosphatase (ALP)							
WN	Route	2.5948	1	11.5678	0.1341	ns	0.4663
MN	Route	5.8150	1	7.8255	0.0431	*	0.7513
Ctrl	Diet	0.0521	1	6.0890	0.8270	ns	0.2699
SF	Diet	9.7419	1	12.6641	0.0083	**	0.8595
amylase (AMY)							
WN	Route	2.2199	1	12.8555	0.1604	ns	0.5173

MN	Route	0.5645	1	7.8712	0.4743	ns	0.2528
Ctrl	Diet	208.9552	1	8.7817	<0.0001	****	1.1061
SF	Diet	248.6190	1	12.8663	<0.0001	****	1.1373
Glucose (GLU)							
WN	Route	2.0250	1	31.0000	0.1650	ns	0.0610
MN	Route	1.2360	1	31.0000	0.2750	ns	0.0380
Ctrl	Diet	22.2650	1	31.0000	<0.0001	*	0.4180
SF	Diet	10.2060	1	31.0000	0.0030	*	0.2480
total protein (TP)							
WN	Route	1.5880	1	31.0000	0.2170	ns	0.0490
MN	Route	0.6230	1	31.0000	0.4360	ns	0.0200
Ctrl	Diet	17.1730	1	31.0000	0.0002	*	0.3560
SF	Diet	34.9690	1	31.0000	<0.0001	*	0.5300
urea nitrogen (BUN)							
WN	Route	0.4850	1	31.0000	0.4920	ns	0.0150
MN	Route	28.4790	1	31.0000	<0.0001	*	0.4790
Ctrl	Diet	37.5290	1	31.0000	<0.0001	*	0.5480
SF	Diet	224.7880	1	31.0000	<0.0001	*	0.8790

Since both predictors had only two levels each, the pairwise comparison reflected the observations of the one-way analyses above (Appendix table 133).

Appendix Table 133

Pairwise comparison

contrast	df	statistic	p.value	Sig.
alanine aminotransferase (ALT)				
MN Ctrl - MN SF	31	-0.0188	0.1516	ns
WN Ctrl - MN Ctrl	31	0.0073	0.5394	ns
WN Ctrl - WN SF	31	0.0046	0.3780	ns
WN SF - MN SF	31	-0.0162	0.0229	*
albumin (ALB)				
MN Ctrl - MN SF	31	-0.4860	0.6304	ns
WN Ctrl - MN Ctrl	31	5.3658	<0.0001	****
WN Ctrl - WN SF	31	-0.4755	0.6378	ns
WN SF - MN SF	31	6.9518	<0.0001	****

alkaline phosphatase (ALP)				
MN Ctrl - MN SF	31	-0.1965	0.0431	*
WN Ctrl - MN Ctrl	31	0.0173	0.8270	ns
WN Ctrl - WN SF	31	-0.0481	0.1341	ns
WN SF - MN SF	31	-0.1312	0.0083	**
amylase (AMY)				
MN Ctrl - MN SF	31	0.0003	0.4743	ns
WN Ctrl - MN Ctrl	31	-0.0070	<0.0001	****
WN Ctrl - WN SF	31	-0.0006	0.1604	ns
WN SF - MN SF	31	-0.0060	<0.0001	****
Globulin (GLOB)				
MN Ctrl - MN SF	31	-0.0007	1.0000	ns
WN Ctrl - MN Ctrl	31	-0.0461	0.0096	**
WN Ctrl - WN SF	31	-0.0164	0.6860	ns
WN SF - MN SF	31	-0.0304	0.0341	*
Glucose (GLU)				
MN Ctrl - MN SF	31	-1.1120	0.2747	ns
WN Ctrl - MN Ctrl	31	4.7185	<0.0001	****
WN Ctrl - WN SF	31	1.4230	0.1647	ns
WN SF - MN SF	31	3.1947	0.0032	**
total protein (TP)				
MN Ctrl - MN SF	31	-0.7890	0.4361	ns
WN Ctrl - MN Ctrl	31	4.1440	0.0002	***
WN Ctrl - WN SF	31	-1.2600	0.2171	ns
WN SF - MN SF	31	5.9134	<0.0001	****
urea nitrogen (BUN)				
MN Ctrl - MN SF	31	5.3366	<0.0001	****
WN Ctrl - MN Ctrl	31	6.1261	<0.0001	****
WN Ctrl - WN SF	31	-0.6961	0.4915	ns
WN SF - MN SF	31	14.9929	<0.0001	****

Statistical power

Considering the small group sizes (Appendix tables 126), we wanted to ensure that the study design was not significantly underpowered. Thus, we performed a retrospective sample size and power analysis on the data by target.

Effect size estimation based on partial eta^2

Effect sizes were calculated by predictor and the different potential interaction combinations of them. Appendix tables 134 showed the respective effect sizes. Note that upper ends of the confidence intervals were automatically set to 1 for this type of calculation. Large effect sizes reflected statistically meaningful differences in the data analysis. The partial eta^2 values from the effect size calculation were then used for the retrospective power calculations.

Appendix Table 134

Effect size

Parameter	Eta2_partial	CI_low	CI_high	Effect Size
alanine aminotransferase (ALT)				
Diet	0.0539	0.0000	1	small
Route	0.0471	0.0000	1	small
Diet:Route	0.0771	0.0000	1	medium
albumin (ALB)				
Diet	0.6994	0.5399	1	large
Route	0.0147	0.0000	1	small
Diet:Route	0.0000	0.0000	1	very small
alkaline phosphatase (ALP)				
Diet	0.1379	0.0063	1	medium
Route	0.2028	0.0337	1	large
Diet:Route	0.1081	0.0000	1	medium
amylase (AMY)				
Diet	0.8626	0.7822	1	large
Route	0.0143	0.0000	1	small
Diet:Route	0.0483	0.0000	1	small
Globulin (GLOB)				
Diet	0.3390	0.1263	1	large
Route	0.0135	0.0000	1	small
Diet:Route	0.0135	0.0000	1	small
Glucose (GLU)				
Diet	0.5107	0.2977	1	large
Route	0.0014	0.0000	1	very small
Diet:Route	0.0938	0.0000	1	medium
total protein (TP)				

Diet	0.6050	0.4127	1	large
Route	0.0632	0.0000	1	medium
Diet:Route	0.0033	0.0000	1	very small
urea nitrogen (BUN)				
Diet	0.8634	0.7835	1	large
Route	0.2614	0.0680	1	large
Diet:Route	0.3725	0.1554	1	large

Retrospective minimum total sample size estimation for 80% power

The accepted rule of thumb is to have at least 80% (0.8 as ratio) statistical power in once data. For small mean differences within data with a high level of complexity, this is often hard to achieve, because of cost and ability to manage large sample sizes. In most instances, proposed sample sizes based on our data, suggested that actual sample sizes were frequently too small for a chance of detecting statistical significant differences, particularly, for the interaction of predictors (Appendix tables 135).

Appendix Table 135

Minimum optimal sample size calculation

Effect	power	n.total	ncp	df1	df2
alanine aminotransferase (ALT) - Diet					
Diet	0.8	**140**	7.96	1	137.61
alanine aminotransferase (ALT) - Route					
Route	0.8	**161**	7.94	1	158.61
alanine aminotransferase (ALT) - Diet:Route					
Diet	0.8	**96**	8.02	1	91.94
Route	0.8	**96**	8.02	1	91.94
Diet:Route	0.8	**96**	8.02	1	91.94
albumin (ALB) - Diet					
Diet	0.8	**7**	14.05	1	4.04
albumin (ALB) - Route					
Route	0.8	**529**	7.88	1	526.34
albumin (ALB) - Diet:Route					
Diet	0.8	**1592383**	7.85	1	1592379.00
Route	0.8	**1592383**	7.85	1	1592379.00
Diet:Route	0.8	**1592383**	7.85	1	1592379.00

alkaline phosphatase (ALP) - Diet					
Diet	0.8	**52**	8.17	1	49.08
alkaline phosphatase (ALP) - Route					
Route	0.8	**33**	8.37	1	30.89
alkaline phosphatase (ALP) - Diet:Route					
Diet	0.8	**67**	8.10	1	62.77
Route	0.8	**67**	8.10	1	62.77
Diet:Route	0.8	**67**	8.10	1	62.77
amylase (AMY) - Diet					
Diet	0.8	**5**	26.55	1	2.23
amylase (AMY) - Route					
Route	0.8	**543**	7.88	1	540.71
amylase (AMY) - Diet:Route					
Diet	0.8	**157**	7.95	1	152.68
Route	0.8	**157**	7.95	1	152.68
Diet:Route	0.8	**157**	7.95	1	152.68
Globulin (GLOB) - Diet					
Diet	0.8	**18**	8.95	1	15.45
Globulin (GLOB) - Route					
Route	0.8	**578**	7.88	1	575.62
Globulin (GLOB) - Diet:Route					
Diet	0.8	**578**	7.88	1	573.62
Route	0.8	**578**	7.88	1	573.62
Diet:Route	0.8	**578**	7.88	1	573.62
Glucose (GLU) - Diet					
Diet	0.8	**10**	10.30	1	7.87
Glucose (GLU) - Route					
Route	0.8	**5683**	7.85	1	5680.50
Glucose (GLU) - Diet:Route					
Diet	0.8	**78**	8.06	1	73.89
Route	0.8	**78**	8.06	1	73.89

Diet:Route	0.8	**78**	8.06	1	73.89
total protein (TP) - Diet					
Diet	0.8	**8**	11.67	1	5.62
total protein (TP) - Route					
Route	0.8	**119**	7.98	1	116.25
total protein (TP) - Diet:Route					
Diet	0.8	**2341**	7.86	1	2336.38
Route	0.8	**2341**	7.86	1	2336.38
Diet:Route	0.8	**2341**	7.86	1	2336.38
urea nitrogen (BUN) - Diet					
Diet	0.8	**5**	26.69	1	2.22
urea nitrogen (BUN) - Route					
Route	0.8	**25**	8.59	1	22.26
urea nitrogen (BUN) - Diet:Route					
Diet	0.8	**16**	9.35	1	11.76
Route	0.8	**16**	9.35	1	11.76
Diet:Route	0.8	**16**	9.35	1	11.76

Retrospective calculation of statistical power in our data analysis

With the exception of urea nitrogen, we generally observed that our study was underpowered for the detection of a statistical significant difference, in particular, for the infection route and the interaction with "Diet" (Appendix Table 136). Even so, excessively large proposed sample sizes and small retrospective statistical power can also be an indicator that there is in fact no meaningful biological difference in this instance for the infection route and, thus identifying "Diet" as the main cause of difference in target levels is a robust outcome.

Appendix Table 136

Statistical power of data

Effect	power	n.total	ncp	df1	df2
alanine aminotransferase (ALT) - Diet					
Diet	**0.28**	35	2.00	1	33
alanine aminotransferase (ALT) - Route					
Route	**0.25**	35	1.73	1	33

alanine aminotransferase (ALT) - Diet:Route					
Diet	**0.38**	35	2.92	1	31
Route	**0.38**	35	2.92	1	31
Diet:Route	**0.38**	35	2.92	1	31
albumin (ALB) - Diet					
Diet	**1.00**	35	81.42	1	33
albumin (ALB) - Route					
Route	**0.11**	35	0.52	1	33
albumin (ALB) - Diet:Route					
Diet	**0.05**	35	0.00	1	31
Route	**0.05**	35	0.00	1	31
Diet:Route	**0.05**	35	0.00	1	31
alkaline phosphatase (ALP) - Diet					
Diet	**0.63**	35	5.60	1	33
alkaline phosphatase (ALP) - Route					
Route	**0.82**	35	8.90	1	33
alkaline phosphatase (ALP) - Diet:Route					
Diet	**0.51**	35	4.24	1	31
Route	**0.51**	35	4.24	1	31
Diet:Route	**0.51**	35	4.24	1	31
amylase (AMY) - Diet					
Diet	**1.00**	35	219.66	1	33
amylase (AMY) - Route					
Route	**0.11**	35	0.51	1	33
amylase (AMY) - Diet:Route					
Diet	**0.25**	35	1.78	1	31
Route	**0.25**	35	1.78	1	31
Diet:Route	**0.25**	35	1.78	1	31
Globulin (GLOB) - Diet					
Diet	**0.98**	35	17.95	1	33
Globulin (GLOB) - Route					

Route	**0.10**	35	0.48	1	33
Globulin (GLOB) - Diet:Route					
Diet	**0.10**	35	0.48	1	31
Route	**0.10**	35	0.48	1	31
Diet:Route	**0.10**	35	0.48	1	31
Glucose (GLU) - Diet					
Diet	**1.00**	35	36.53	1	33
Glucose (GLU) - Route					
Route	**0.06**	35	0.05	1	33
Glucose (GLU) - Diet:Route					
Diet	**0.45**	35	3.62	1	31
Route	**0.45**	35	3.62	1	31
Diet:Route	**0.45**	35	3.62	1	31
total protein (TP) - Diet					
Diet	**1.00**	35	53.61	1	33
total protein (TP) - Route					
Route	**0.32**	35	2.36	1	33
total protein (TP) - Diet:Route					
Diet	**0.06**	35	0.12	1	31
Route	**0.06**	35	0.12	1	31
Diet:Route	**0.06**	35	0.12	1	31
urea nitrogen (BUN) - Diet					
Diet	**1.00**	35	221.26	1	33
urea nitrogen (BUN) - Route					
Route	**0.93**	35	12.39	1	33
urea nitrogen (BUN) - Diet:Route					
Diet	**0.99**	35	20.77	1	31
Route	**0.99**	35	20.77	1	31
Diet:Route	**0.99**	35	20.77	1	31

Panel c

Data analysis

Here, we present statistical comparison of systemic heme-oxygenase-1 of a total of N=67 well-nourished (WN) or malnourished (MN) BALB/c mice infected by sand fly bite (SF) or not (Ctrl) (Appendix table 137).

Appendix Table 137

Summary information

Targets	Total N	WN_Ctrl	MN_Ctrl	WN_SF	MN_SF	Transformation
Figure 4c	67	15	8	21	23	Box-Cox power Transformed

We needed to analyze the data with a two-way approach to account for the two predictors, "Diet" and "Route", both of which were between-subject factors.

For a two-way ANOVA, we had to assess the data for compliance with assumptions:

- Data normality
- Homogeneity of variance
- No significant outliers

Assumption analyses

Please, note that all assumption test results shown were post data transformation, where applicable (Appendix table 137).

Data normality

The assessment of the untransformed data distribution for each group was conducted by Shapiro-Wilks test and QQ-plot after splitting the data by both predictors. Note that all groups consisted of <30 data points (range[8 to 23]) (Appendix table 137), which made groups too small to assess data distribution reliably by Shapiro-Wilks test. We executed the test anyway as an indicator of gross departure of data normality. Thus, we performed the analyses by Shapiro-Wilks test (Appendix table 138) and QQ-plots (Fig.S1a-1). We found a departure of normality for the malnourished, sand fly infected group, which was improved by data transformation but not completely remedied.

Appendix Table 138

Univariate Shapito-Wilks test results

Diet	Route	variable	statistic	p	Outcome
WN	Ctrl	Counts	0.9176	0.1770	ns
WN	SF	Counts	0.9522	0.3741	ns

MN	Ctrl	Counts	0.9788	0.9569	ns
MN	SF	Counts	0.9026	0.0286	sig.

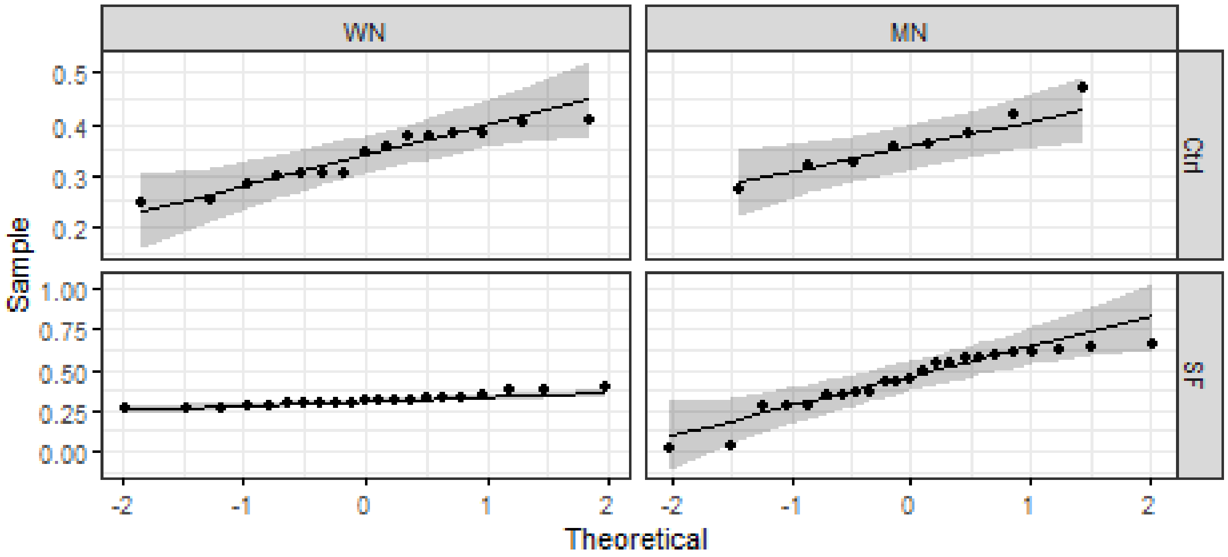

Fig.4c-1: QQ-plots of Heme-oxyginase-1 data

Homogeneity of variance

The assessment of homogeneity of variance was conducted by Levene's test for the dataset. The analysis output showed that assumption of homogeneity between groups was violated as the manlourished, sand fly infected group had much larger variance than other three groups (Appendix table 139).

Appendix Table 139

Assessment of homogeneity of variance by target

Targets	df1	df2	statistic	p	sig.
Figure 4c	3	63	13.654	<0.0001	****

Outliers

A total of one outlier was observed, but not an extreme one (Appendix table 140).

Appendix Table 140

List of possible outliers

Diet	Route	Tissue	is.outlier	is.extreme
WN	SF	Serum	TRUE	FALSE

Two-way analysis

Based on the assumption analysis, we applied an appropriate two-way test to the respective datasets (Appendix table 141).

Appendix Table 141

Applied tests

Two-way test	One-way test	Pairwise
Robust two-way ANOVA	Robust one-way ANOVA	Linear contrast expression

The respective two-way analyses showed that statistical significance for "Diet" and its interaction with "Route", but not for "Route" by itself (Appendix table 142), suggesting that malnourishment significantly increased HO-1 expression.

Appendix Table 142

Two-way analysis

Effect	DFn	DFd	Statistic	p.value	p<.05	ges
Diet	1	31	12.2698	0.002	**	NA
Route	1	31	2.0016	0.170		NA
Diet:Route	1	31	7.0260	0.014	*	NA

For the analysis of the simple main effect for each respective between-subject factor for each target, we performed one-way analyses with the data split by always by the predictor that was not used as within the function. The results showed statistical significance when both malnourished groups were compared (Appendix table 143).

Appendix Table 143

Simple main effect analysis

Factor	Effect	statistic	df1	df2	p.value	Sig.	effsize
WN	Route	2.0470	1	19.3826	0.1684	ns	0.4332
MN	Route	5.5716	1	24.9806	0.0264	*	0.3924
Ctrl	Diet	1.0120	1	14.0782	0.3314	ns	0.3640
SF	Diet	17.8952	1	20.4862	0.0004	***	0.7159

Since both predictors had only two levels each, the pairwise comparison reflected the observations of the one-way analyses above (Table 144).

Appendix Table 144

Pairwise comparison

contrast	df	statistic	p.value	Sig.
MN Ctrl - MN SF	31	-0.0950	0.0264	*
WN Ctrl - MN Ctrl	31	-0.0271	0.3314	ns
WN Ctrl - WN SF	31	0.0260	0.1684	ns
WN SF - MN SF	31	-0.1480	0.0004	***

Statistical power

Considering the small group sizes (Appendix tables 137), we wanted to ensure that the study design was not significantly statistically underpowered. Thus, we performed a retrospective sample size and power analysis on the data by target.

Effect size estimation based on partial eta^2

Effect sizes were calculated by predictor and the different potential interaction combinations of them. Appendix tables 145 shows the respective effect sizes. Note that upper ends of the confidence intervals were automatically set to 1 for this type of calculation. Large effect sizes reflected statistically meaningful differences in the data analysis. The partial eta^2 values from the effect size calculation were then used for the retrospective power calculations.

Appendix Table 145

Effect size

Parameter	Eta2_partial	CI_low	CI_high	Effect Size
Figure 4c				
Diet	0.1188	0.0225	1	medium
Route	0.0548	0.0000	1	small
Diet:Route	0.0799	0.0063	1	medium

Retrospective minimum total sample size estimation for 80% power

The accepted rule of thumb is to have at least 80% (0.8 as ratio) statistical power in once data. For small mean differences within data of a high level of complexity, this is often hard to achieve, because of cost and ability to manage large sample sizes. In most instances, proposed sample sizes based on our data suggested that actual sample sizes were frequently too small for a chance of detecting statistical significant differences, particularly, for infection "Route" (Appendix tables 146).

Appendix Table 146

Minimum optimal sample size calculation

Parameters	effect	power	n.total	ncp	df1	df2
Diet	Diet	0.8	**61**	8.12	1	58.19
Route	Route	0.8	**138**	7.96	1	135.39
Diet:Route	Diet	0.8	**93**	8.02	1	88.38
Diet:Route	Route	0.8	**93**	8.02	1	88.38
Diet:Route	Diet:Route	0.8	**93**	8.02	1	88.38

Retrospective calculation of statistical power in our data analysis

The retrospective power calculation suggested that the study was underpowered, but not terribly (Appendix Table 147). Even so, excessively large proposed sample sizes and small retrospective statistical power can also be an indicator that there was in fact no meaningful biological difference to be found. Considering that statistical significant difference were still found, suggested that the above observations were real.

Appendix Table 147

Statistical power of data

Parameters	effect	power	n.total	ncp	df1	df2
Diet	Diet	**0.84**	67	9.03	1	65
Route	Route	**0.49**	67	3.88	1	65
Diet:Route	Diet	**0.66**	67	5.82	1	63
Diet:Route	Route	**0.66**	67	5.82	1	63
Diet:Route	Diet:Route	**0.66**	67	5.82	1	63

Panel d

Data analysis

Here, we present statistical comparison of serum cytokine concentrations (in pg/ml) for a total of N=38 well-nourished (WN) or malnourished (MN) BALB/c mice infected by sand fly bite (SF) or not (Ctrl) (Appendix table 148). A total of 11 targets were measured (IFN-gamma, IL-10, IL-12p70, IL-17A, IL-18, IL-2, IL-22, IL-4, IL-5, IL-6 & TNF-alpha) per mouse.

Appendix Table 148

Summary information

Targets	Total N	WN_Ctrl	MN_Ctrl	WN_SF	MN_SF	Transformation
IFN-gamma	38	7	7	11	13	Box-Cox power Transformed
IL-10	38	7	7	11	13	Box-Cox power Transformed

IL-12p70	38	7	7	11	13	Box-Cox power Transformed
IL-17A	38	7	7	11	13	Box-Cox power Transformed
IL-18	38	7	7	11	13	Box-Cox power Transformed
IL-2	38	7	7	11	13	Box-Cox power Transformed
IL-22	38	7	7	11	13	Box-Cox power Transformed
IL-4	38	7	7	11	13	Box-Cox power Transformed
IL-5	38	7	7	11	13	Box-Cox power Transformed
IL-6	38	7	7	11	13	Box-Cox power Transformed
TNF-alpha	38	7	7	11	13	Box-Cox power Transformed

We needed to analyze the data with a two-way approach to account for the two predictors, "Diet" and "Route", both of which were between-subject factors.

For a two-way ANOVA, we had to assess the data for compliance with assumptions:

- Data normality
- Homogeneity of variance
- No significant outliers

Assumption analyses

Please, note that all assumption test results shown were post data transformation, where applicable (Appendix table 148).

Data normality

The assessment of the untransformed data distribution for each group was conducted by Shapiro-Wilks test and QQ-plot after splitting the data by both predictors. Note that all groups consisted of <30 data points (range[7 to 13]) (Appendix table 148), which made groups too small to assess data distribution reliably by Shapiro-Wilks test. We executed the test anyway as an indicator of gross departure of data normality. Thus, we performed the analyses by Shapiro-Wilks test (Appendix table 149) and QQ-plots (Fig.S1a-1). We found occasional departure of normality, which could not all be remedied by data transformation.

Appendix Table 149

Univariate Shapito-Wilks test results

Diet	Route	variable	statistic	p	Outcome
IFN-gamma					
WN	Ctrl	Counts	0.9476	0.7075	ns
WN	SF	Counts	0.9163	0.2890	ns
MN	Ctrl	Counts	0.9088	0.3873	ns
MN	SF	Counts	0.9848	0.9951	ns

IL-10					
WN	Ctrl	Counts	0.9146	0.4288	ns
WN	SF	Counts	0.9234	0.3476	ns
MN	Ctrl	Counts	0.9419	0.6557	ns
MN	SF	Counts	0.8749	0.0608	ns
IL-12p70					
WN	Ctrl	Counts	0.8118	0.0535	ns
WN	SF	Counts	0.8217	0.0182	sig.
MN	Ctrl	Counts	0.8917	0.2838	ns
MN	SF	Counts	0.8981	0.1261	ns
IL-17A					
WN	Ctrl	Counts	0.8546	0.1353	ns
WN	SF	Counts	0.8886	0.1336	ns
MN	Ctrl	Counts	0.9320	0.5683	ns
MN	SF	Counts	0.9724	0.9212	ns
IL-18					
WN	Ctrl	Counts	0.9540	0.7661	ns
WN	SF	Counts	0.9226	0.3411	ns
MN	Ctrl	Counts	0.9828	0.9721	ns
MN	SF	Counts	0.8763	0.0636	ns
IL-2					
WN	Ctrl	Counts	0.9561	0.7851	ns
WN	SF	Counts	0.9565	0.7270	ns
MN	Ctrl	Counts	0.8783	0.2190	ns
MN	SF	Counts	0.8492	0.0278	sig.
IL-22					
WN	Ctrl	Counts	0.8472	0.1158	ns
WN	SF	Counts	0.9432	0.5584	ns
MN	Ctrl	Counts	0.8818	0.2347	ns
MN	SF	Counts	0.9459	0.5382	ns
IL-4					
WN	Ctrl	Counts	0.8578	0.1445	ns
WN	SF	Counts	0.9244	0.3567	ns
MN	Ctrl	Counts	0.9393	0.6326	ns
MN	SF	Counts	0.9397	0.4530	ns

IL-5					
WN	Ctrl	Counts	0.9194	0.4648	ns
WN	SF	Counts	0.9288	0.3986	ns
MN	Ctrl	Counts	0.9708	0.9042	ns
MN	SF	Counts	0.8572	0.0354	sig.
IL-6					
WN	Ctrl	Counts	0.8487	0.1195	ns
WN	SF	Counts	0.8269	0.0213	sig.
MN	Ctrl	Counts	0.7714	0.0211	sig.
MN	SF	Counts	0.9486	0.5777	ns
TNF-alpha					
WN	Ctrl	Counts	0.8392	0.0976	ns
WN	SF	Counts	0.9171	0.2950	ns
MN	Ctrl	Counts	0.8987	0.3229	ns
MN	SF	Counts	0.9372	0.4218	ns

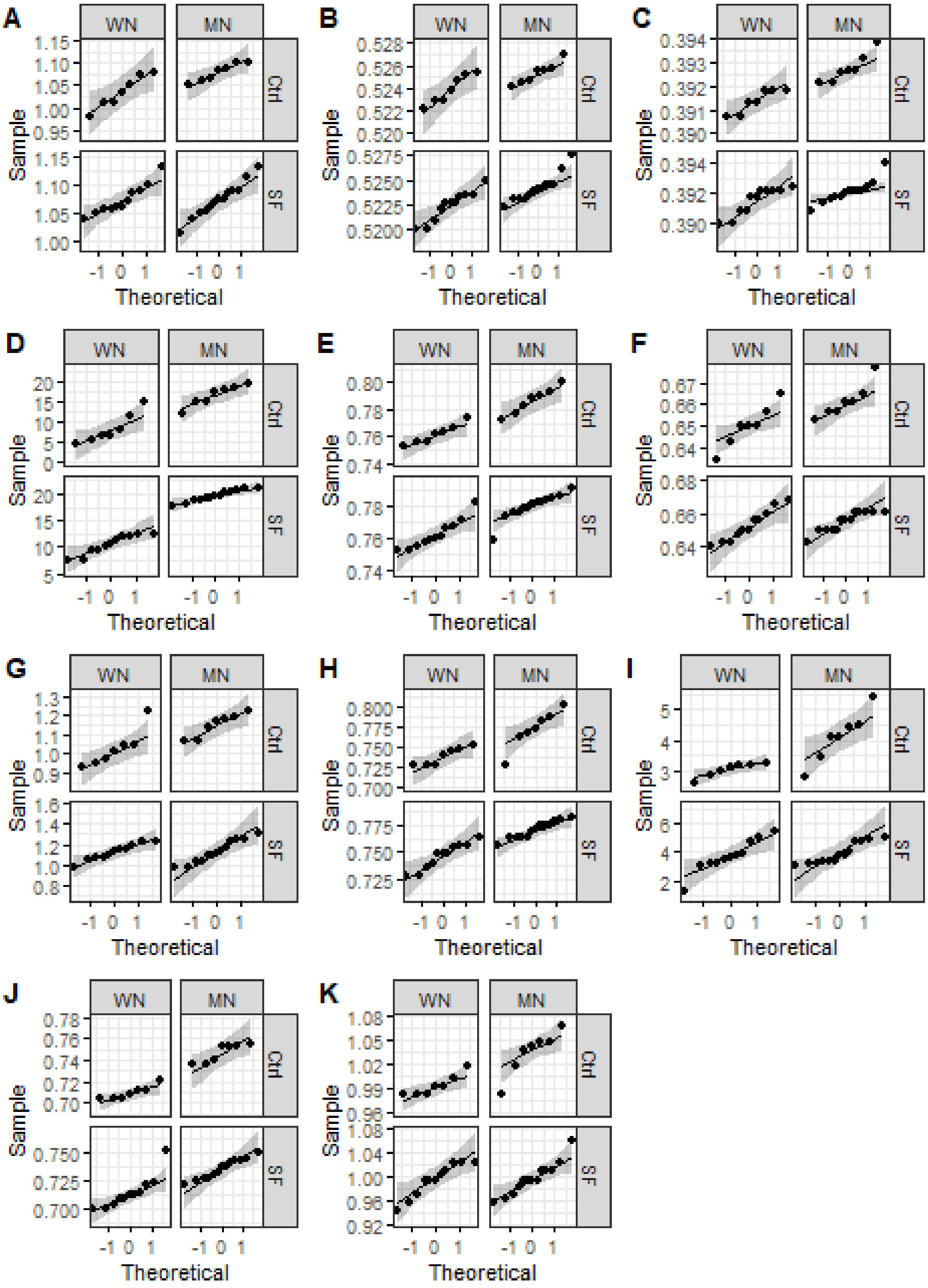

Fig.4d-1: QQ-plots of serum cytokine data: A) IFN-gamma, B) IL-10, C) IL-12p70, D) IL-17A, E) IL-18, F) IL-2, G) IL-22, H) IL-4, I) IL-5, J) IL-6 and K) TNF-alpha

Homogeneity of variance

The assessment of homogeneity of variance was conducted by Levene's test for the dataset. The analysis output showed that assumption of homogeneity between groups held for all cytokines (Appendix table 150).

Appendix Table 150

Assessment of homogeneity of variance by target

Targets	df1	df2	statistic	p	sig.
IFN-gamma	3	34	0.5093	0.6786	ns
IL-10	3	34	0.2930	0.8302	ns
IL-12p70	3	34	0.7980	0.5036	ns
IL-17A	3	34	1.7416	0.1770	ns
IL-18	3	34	0.3842	0.7650	ns
IL-2	3	34	0.5862	0.6282	ns
IL-22	3	34	0.8989	0.4518	ns
IL-4	3	34	2.1753	0.1090	ns
IL-5	3	34	1.9035	0.1476	ns
IL-6	3	34	0.5496	0.6519	ns
TNF-alpha	3	34	0.6531	0.5865	ns

Outliers

Conversely, we detected outliers for 9 of the 11 cytokines (Appendix table 151). Only two of these outliers were classified as extreme (one each for IL-12p70 and IL-6).

Appendix Table 151

List of possible outliers

Diet	Route	Tissue	is.outlier	is.extreme
IL-10				
MN	SF	IL-10	TRUE	FALSE
IL-12p70				
MN	Ctrl	IL-12p70	TRUE	FALSE
MN	SF	IL-12p70	TRUE	TRUE
MN	SF	IL-12p70	TRUE	FALSE
IL-18				
MN	SF	IL-18	TRUE	FALSE

IL-2				
WN	Ctrl	IL-2	TRUE	FALSE
WN	Ctrl	IL-2	TRUE	FALSE
MN	Ctrl	IL-2	TRUE	FALSE
IL-22				
WN	Ctrl	IL-22	TRUE	FALSE
IL-4				
MN	Ctrl	IL-4	TRUE	FALSE
IL-5				
WN	SF	IL-5	TRUE	FALSE
IL-6				
WN	SF	IL-6	TRUE	TRUE
TNF-alpha				
MN	Ctrl	TNF-alpha	TRUE	FALSE
MN	SF	TNF-alpha	TRUE	FALSE

Two-way analysis

Based on the assumption analysis, we decided to applied an appropriate two-way tests to the respective datasets (Appendix table 152).

Appendix Table 152

Applied tests

Targets	Two-way test	One-way test	Pairwise
IFN-gamma	Standard two-way ANOVA	Standard one-way ANOVA	Estimated marginal means analysis
IL-10	Standard two-way ANOVA	Standard one-way ANOVA	Estimated marginal means analysis
IL-12p70	Robust two-way ANOVA	Robust one-way ANOVA	Linear contrast expression
IL-17A	Standard two-way ANOVA	Standard one-way ANOVA	Estimated marginal means analysis
IL-18	Standard two-way ANOVA	Standard one-way ANOVA	Estimated marginal means analysis
IL-2	Robust two-way ANOVA	Robust one-way ANOVA	Linear contrast expression
IL-22	Standard two-way ANOVA	Standard one-way ANOVA	Estimated marginal means analysis
IL-4	Standard two-way ANOVA	Standard one-way ANOVA	Estimated marginal means analysis
IL-5	Robust two-way ANOVA	Robust one-way ANOVA	Linear contrast expression
IL-6	Robust two-way ANOVA	Robust one-way ANOVA	Linear contrast expression
TNF-alpha	Standard two-way ANOVA	Standard one-way ANOVA	Estimated marginal means analysis

The respective two-way analyses showed that there was no statistical significant differences detected for IL-22 (Appendix table 153), suggesting that this cytokine was not statistically significantly affected by either nutritional status and/or infection route. All other targets had detectable statistical significant differences mostly in relation to “Diet” with the exception of IFN-γ, where only the interaction term of “Diet” and “Route” was statistically significant. IL-10 and IL-17A also showed statistical significance for the infection route, but not for the interaction term. Conversely, IL-6 showed a statistically significant interaction between “Diet” and “Route”, while not for “Route” on its own. Finally, TNF-α showed statistical significance for both predictors as much as their interaction. This suggested that most serum cytokine levels were affected by the nutritional state of the mice, while some changed their serum concentration with respect to the infection.

Appendix Table 153

Two-way analysis

Effect	DFn	DFd	Statistic	p.value	p<.05	ges
IFN-gamma						
Diet	1	34	2.7010	0.1100		0.074
Route	1	34	2.2870	0.1400		0.063
Diet:Route	1	34	4.6710	0.0380	*	0.121
IL-10						
Diet	1	34	14.8460	0.0005	*	0.304
Route	1	34	8.1980	0.0070	*	0.194
Diet:Route	1	34	0.1240	0.7270		0.004
IL-12p70						
Diet	1	31	10.8281	0.0040	**	NA
Route	1	31	0.5867	0.4550		NA
Diet:Route	1	31	3.1397	0.0950	+	NA
IL-17A						
Diet	1	34	149.0550	<0.0001	*	0.814
Route	1	34	13.2900	0.0009	*	0.281
Diet:Route	1	34	0.3000	0.5870		0.009
IL-18						
Diet	1	34	52.9080	<0.0001	*	0.609
Route	1	34	1.0700	0.3080		0.030
Diet:Route	1	34	1.9850	0.1680		0.055
IL-2						
Diet	1	31	6.8192	0.0170	*	NA

Route	1	31	0.1834	0.6740		NA
Diet:Route	1	31	1.2226	0.2840		NA
IL-22						
Diet	1	34	2.4590	0.1260		0.067
Route	1	34	0.9990	0.3250		0.029
Diet:Route	1	34	3.8250	0.0590		0.101
IL-4						
Diet	1	34	40.7310	<0.0001	*	0.545
Route	1	34	0.2550	0.6170		0.007
Diet:Route	1	34	1.2570	0.2700		0.036
IL-5						
Diet	1	31	5.3171	0.0330	*	NA
Route	1	31	0.5066	0.4860		NA
Diet:Route	1	31	3.4406	0.0800	+	NA
IL-6						
Diet	1	31	77.5334	0.0010	***	NA
Route	1	31	1.4924	0.2430		NA
Diet:Route	1	31	4.6554	0.0490	*	NA
TNF-alpha						
Diet	1	34	4.4970	0.0410	*	0.117
Route	1	34	5.0150	0.0320	*	0.129
Diet:Route	1	34	4.9680	0.0330	*	0.127

For the analysis of the simple main effect for each respective between-subject factor for each target, we performed one-way analyses with the data split by always by the predictor that was not used as within the function. The results showed that statistical significance for each cytokine, including IL-22, which had not shown statistical significance in the two-way analysis (Table 154). Results were quite varied between cytokines, frequently showing statistically significant differences between well-nourished uninfected and sand fly infected mice (IFN-γ, IL-10, IL-17A, IL-22 & IL-5) and well- and malnourished uninfected control groups showed statistical significant differences for all measured serum cytokines. The latter indicated that the nutritional status of the mice altered their steady state levels of measured cytokines, increasing them significantly in the malnourished group; a difference negated after parasite infection by sand fly.

Appendix Table 154

Simple main effect analysis

Factor	Effect	statistic	df1	df2	p.value	Sig.	effsize
IFN-gamma							
WN	Route	6.7820	1	34.0000	0.0140	*	0.1660
MN	Route	0.1750	1	34.0000	0.6780	ns	0.0050
Ctrl	Diet	7.3710	1	34.0000	0.0100	*	0.1780
SF	Diet	0.0001	1	34.0000	0.9920	ns	0.0000
IL-10							
WN	Route	5.0460	1	34.0000	0.0310	*	0.1290
MN	Route	3.2760	1	34.0000	0.0790	ns	0.0880
Ctrl	Diet	4.2600	1	34.0000	0.0470	*	0.1110
SF	Diet	10.7100	1	34.0000	0.0020	*	0.2400
IL-12p70							
WN	Route	0.1683	1	11.8637	0.6890	ns	0.2715
MN	Route	7.4009	1	11.0197	0.0199	*	0.7033
Ctrl	Diet	23.5358	1	11.3978	0.0005	***	1.0176
SF	Diet	1.8848	1	11.0264	0.1971	ns	0.4002
IL-17A							
WN	Route	4.5990	1	34.0000	0.0390	*	0.1190
MN	Route	8.9910	1	34.0000	0.0050	*	0.2090
Ctrl	Diet	48.8890	1	34.0000	<0.0001	*	0.5900
SF	Diet	100.4660	1	34.0000	<0.0001	*	0.7470
IL-18							
WN	Route	0.0850	1	34.0000	0.7720	ns	0.0020
MN	Route	2.9700	1	34.0000	0.0940	ns	0.0800
Ctrl	Diet	30.7250	1	34.0000	<0.0001	*	0.4750
SF	Diet	24.1680	1	34.0000	<0.0001	*	0.4150
IL-2							
WN	Route	0.2541	1	12.7323	0.6228	ns	0.2539
MN	Route	2.7345	1	9.6835	0.1302	ns	0.5742
Ctrl	Diet	5.9103	1	11.4433	0.0326	*	0.8855
SF	Diet	0.9822	1	12.2585	0.3408	ns	0.3648
IL-22							
WN	Route	4.4090	1	34.0000	0.0430	*	0.1150
MN	Route	0.4150	1	34.0000	0.5240	ns	0.0120
Ctrl	Diet	6.2810	1	34.0000	0.0170	*	0.1560

SF	Diet	0.0030	1	34.0000	0.9560	ns	0.0001
IL-4							
WN	Route	1.3380	1	34.0000	0.2550	ns	0.0380
MN	Route	0.1740	1	34.0000	0.6790	ns	0.0050
Ctrl	Diet	22.7750	1	34.0000	<0.0001	*	0.4010
SF	Diet	19.2140	1	34.0000	0.0001	*	0.3610
IL-5							
WN	Route	6.5238	1	9.5085	0.0297	*	0.6051
MN	Route	0.4086	1	11.7601	0.5350	ns	0.2637
Ctrl	Diet	10.8185	1	6.9422	0.0135	*	0.9481
SF	Diet	0.0365	1	16.0327	0.8509	ns	0.2319
IL-6							
WN	Route	0.2440	1	13.6262	0.6292	ns	0.3896
MN	Route	6.9094	1	11.8268	0.0223	*	0.6698
Ctrl	Diet	75.3053	1	10.4658	<0.0001	****	1.0480
SF	Diet	32.7090	1	15.9433	<0.0001	****	0.9228
TNF-alpha							
WN	Route	0.0020	1	34.0000	0.9670	ns	0.0000
MN	Route	9.9810	1	34.0000	0.0030	*	0.2270
Ctrl	Diet	9.3580	1	34.0000	0.0040	*	0.2160
SF	Diet	0.1070	1	34.0000	0.7450	ns	0.0030

Since both predictors had only two levels each, the pairwise comparison reflected the observations of the one-way analyses above (Table 155).

Appendix Table 155

Pairwise comparison

contrast	df	statistic	p.value	Sig.
IFN-gamma				
MN Ctrl - MN SF	34	0.4188	0.6780	ns
WN Ctrl - MN Ctrl	34	-2.7150	0.0103	*
WN Ctrl - WN SF	34	-2.6042	0.0136	*
WN SF - MN SF	34	0.0102	0.9919	ns
IL-10				
MN Ctrl - MN SF	34	1.8101	0.0791	ns

WN Ctrl - MN Ctrl	34	-2.0639	0.0467	*
WN Ctrl - WN SF	34	2.2463	0.0313	*
WN SF - MN SF	34	-3.2726	0.0024	**
IL-12p70				
MN Ctrl - MN SF	31	0.0007	0.0199	*
WN Ctrl - MN Ctrl	31	-0.0014	0.0005	***
WN Ctrl - WN SF	31	-0.0001	0.6890	ns
WN SF - MN SF	31	-0.0005	0.1971	ns
IL-17A				
MN Ctrl - MN SF	34	-2.9985	0.0050	**
WN Ctrl - MN Ctrl	34	-6.9920	<0.0001	****
WN Ctrl - WN SF	34	-2.1445	0.0392	*
WN SF - MN SF	34	-10.0233	<0.0001	****
IL-18				
MN Ctrl - MN SF	34	1.7233	0.0939	ns
WN Ctrl - MN Ctrl	34	-5.5430	<0.0001	****
WN Ctrl - WN SF	34	-0.2916	0.7723	ns
WN SF - MN SF	34	-4.9161	<0.0001	****
IL-2				
MN Ctrl - MN SF	31	0.0056	0.1302	ns
WN Ctrl - MN Ctrl	31	-0.0116	0.0326	*
WN Ctrl - WN SF	31	-0.0025	0.6228	ns
WN SF - MN SF	31	-0.0035	0.3408	ns
IL-22				
MN Ctrl - MN SF	34	0.6439	0.5240	ns
WN Ctrl - MN Ctrl	34	-2.5062	0.0172	*
WN Ctrl - WN SF	34	-2.0998	0.0432	*
WN SF - MN SF	34	-0.0550	0.9565	ns
IL-4				
MN Ctrl - MN SF	34	0.4177	0.6788	ns
WN Ctrl - MN Ctrl	34	-4.7723	<0.0001	****
WN Ctrl - WN SF	34	-1.1569	0.2554	ns
WN SF - MN SF	34	-4.3833	0.0001	***
IL-5				
MN Ctrl - MN SF	31	0.2460	0.5350	ns

WN Ctrl - MN Ctrl	31	-1.0729	0.0135	*
WN Ctrl - WN SF	31	-0.7580	0.0297	*
WN SF - MN SF	31	-0.0688	0.8509	ns
IL-6				
MN Ctrl - MN SF	31	0.0114	0.0223	*
WN Ctrl - MN Ctrl	31	-0.0367	<0.0001	****
WN Ctrl - WN SF	31	-0.0020	0.6292	ns
WN SF - MN SF	31	-0.0234	<0.0001	****
TNF-alpha				
MN Ctrl - MN SF	34	3.1593	0.0033	**
WN Ctrl - MN Ctrl	34	-3.0590	0.0043	**
WN Ctrl - WN SF	34	-0.0412	0.9674	ns
WN SF - MN SF	34	-0.3273	0.7454	ns

Statistical power

Considering the small group sizes (Appendix tables 148), we wanted to ensure that the study design was not significantly statistically underpowered. Thus, we performed a retrospective sample size and power analysis on the data by target.

Effect size estimation based on partial eta^2

Effect sizes were calculated by predictor and the different potential interaction combinations of them. Appendix tables 156 shows the respective effect sizes. Note that upper ends of the confidence intervals were automatically set to 1 for this type of calculation. Large effect sizes reflected statistically meaningful differences in the data analysis. The partial eta^2 values from the effect size calculation were then used for the retrospective power calculations.

Appendix Table 156

Effect size

Parameter	Eta2_partial	CI_low	CI_high	Effect Size
IFN-gamma				
Diet	0.0221	0.0000	1	small
Route	0.0436	0.0000	1	small
Diet:Route	0.0190	0.0000	1	small
IL-10				
Diet	0.1642	0.0202	1	large
Route	0.0569	0.0000	1	small

Diet:Route	0.0000	0.0000	1	very small
IL-12p70				
Diet	0.1276	0.0062	1	medium
Route	0.0030	0.0000	1	very small
Diet:Route	0.0093	0.0000	1	very small
IL-17A				
Diet	0.5505	0.3554	1	large
Route	0.1633	0.0198	1	large
Diet:Route	0.0588	0.0000	1	small
IL-18				
Diet	0.2859	0.0927	1	large
Route	0.0809	0.0000	1	medium
Diet:Route	0.0933	0.0000	1	medium
IL-2				
Diet	0.1391	0.0102	1	medium
Route	0.0229	0.0000	1	small
Diet:Route	0.0849	0.0000	1	medium
IL-22				
Diet	0.0697	0.0000	1	medium
Route	0.0256	0.0000	1	small
Diet:Route	0.0138	0.0000	1	small
IL-4				
Diet	0.4960	0.2919	1	large
Route	0.0076	0.0000	1	very small
Diet:Route	0.0634	0.0000	1	medium
IL-5				
Diet	0.0728	0.0000	1	medium
Route	0.0192	0.0000	1	small
Diet:Route	0.0814	0.0000	1	medium
IL-6				
Diet	0.3582	0.1521	1	large
Route	0.1521	0.0152	1	large
Diet:Route	0.2372	0.0592	1	large
TNF-alpha				

Diet	0.2127	0.0445	1	large
Route	0.1328	0.0080	1	medium
Diet:Route	0.1641	0.0202	1	large

Retrospective minimum total sample size estimation for 80% power

The accepted rule of thumb is to have at least 80% (0.8 as ratio) statistical power in once data. For small mean differences within data of a high level of complexity, this is often hard to achieve, because of cost and ability to manage large sample sizes. In most instances, proposed sample sizes based on our data, suggested that actual sample sizes were frequently too small for a chance of detecting statistical significant differences, particularly, for the interaction of predictors (Appendix tables 157).

Appendix Table 157

Minimum optimal sample size calculation

Effect	power	n.total	ncp	df1	df2
IFN-gamma - Diet					
Diet	0.8	**350**	7.89	1	347.23
IFN-gamma - Route					
Route	0.8	**175**	7.94	1	172.01
IFN-gamma - Diet:Route					
Diet	0.8	**407**	7.89	1	402.23
Route	0.8	**407**	7.89	1	402.23
Diet:Route	0.8	**407**	7.89	1	402.23
IL-10 - Diet					
Diet	0.8	**42**	8.24	1	39.96
IL-10 - Route					
Route	0.8	**133**	7.97	1	130.02
IL-10 - Diet:Route					
Diet	0.8	**223551**	7.85	1	223546.80
Route	0.8	**223551**	7.85	1	223546.80
Diet:Route	0.8	**223551**	7.85	1	223546.80
IL-12p70 - Diet					
Diet	0.8	**56**	8.14	1	53.65
IL-12p70 - Route					

Route	0.8	**2609**	7.86	1	2606.86
IL-12p70 - Diet:Route					
Diet	0.8	**840**	7.87	1	835.89
Route	0.8	**840**	7.87	1	835.89
Diet:Route	0.8	**840**	7.87	1	835.89
IL-17A - Diet					
Diet	0.8	**9**	10.79	1	6.81
IL-17A - Route					
Route	0.8	**43**	8.24	1	40.24
IL-17A - Diet:Route					
Diet	0.8	**128**	7.97	1	123.66
Route	0.8	**128**	7.97	1	123.66
Diet:Route	0.8	**128**	7.97	1	123.66
IL-18 - Diet					
Diet	0.8	**22**	8.69	1	19.70
IL-18 - Route					
Route	0.8	**92**	8.02	1	89.16
IL-18 - Diet:Route					
Diet	0.8	**79**	8.06	1	74.28
Route	0.8	**79**	8.06	1	74.28
Diet:Route	0.8	**79**	8.06	1	74.28
IL-2 - Diet					
Diet	0.8	**51**	8.17	1	48.58
IL-2 - Route					
Route	0.8	**338**	7.89	1	335.37
IL-2 - Diet:Route					
Diet	0.8	**87**	8.04	1	82.58
Route	0.8	**87**	8.04	1	82.58
Diet:Route	0.8	**87**	8.04	1	82.58
IL-22 - Diet					
Diet	0.8	**107**	8.00	1	104.75

IL-22 - Route					
Route	0.8	**301**	7.90	1	298.94
IL-22 - Diet:Route					
Diet	0.8	**562**	7.88	1	557.09
Route	0.8	**562**	7.88	1	557.09
Diet:Route	0.8	**562**	7.88	1	557.09
IL-4 - Diet					
Diet	0.8	**11**	10.14	1	8.30
IL-4 - Route					
Route	0.8	**1023**	7.86	1	1020.31
IL-4 - Diet:Route					
Diet	0.8	**118**	7.98	1	113.91
Route	0.8	**118**	7.98	1	113.91
Diet:Route	0.8	**118**	7.98	1	113.91
IL-5 - Diet					
Diet	0.8	**102**	8.00	1	99.95
IL-5 - Route					
Route	0.8	**403**	7.89	1	400.15
IL-5 - Diet:Route					
Diet	0.8	**91**	8.03	1	86.53
Route	0.8	**91**	8.03	1	86.53
Diet:Route	0.8	**91**	8.03	1	86.53
IL-6 - Diet					
Diet	0.8	**17**	9.06	1	14.23
IL-6 - Route					
Route	0.8	**46**	8.21	1	43.75
IL-6 - Diet:Route					
Diet	0.8	**28**	8.54	1	23.48
Route	0.8	**28**	8.54	1	23.48
Diet:Route	0.8	**28**	8.54	1	23.48
TNF-alpha - Diet					

Diet	0.8	**32**	8.40	1	29.10
TNF-alpha - Route					
Route	0.8	**54**	8.15	1	51.22
TNF-alpha - Diet:Route					
Diet	0.8	**43**	8.26	1	38.09
Route	0.8	**43**	8.26	1	38.09
Diet:Route	0.8	**43**	8.26	1	38.09

Retrospective calculation of statistical power in our data analysis

With the exception of urea nitrogen, we generally observed that the our study was underpowered for the detection of a statistical significant difference, in particular, for the infection route and the interaction with "Diet" (Appendix Table 158). Even so, excessively large proposed sample sizes and small retrospective statistical power can also be an indicator that there was in fact no meaningful biological difference to be found. Considering that statistical significant difference were still found so frequently, suggested that the above observations were real.

**Appendix Table 158*

Statistical power of data

Effect	power	n.total	ncp	df1	df2
IFN-gamma - Diet					
Diet	**0.15**	38	0.86	1	36
IFN-gamma - Route					
Route	**0.25**	38	1.73	1	36
IFN-gamma - Diet:Route					
Diet	**0.13**	38	0.74	1	34
Route	**0.13**	38	0.74	1	34
Diet:Route	**0.13**	38	0.74	1	34
IL-10 - Diet					
Diet	**0.76**	38	7.47	1	36
IL-10 - Route					
Route	**0.31**	38	2.29	1	36
IL-10 - Diet:Route					
Diet	**0.05**	38	0.00	1	34
Route	**0.05**	38	0.00	1	34

Diet:Route	**0.05**	38	0.00	1	34
IL-12p70 - Diet					
Diet	**0.63**	38	5.56	1	36
IL-12p70 - Route					
Route	**0.06**	38	0.11	1	36
IL-12p70 - Diet:Route					
Diet	**0.09**	38	0.36	1	34
Route	**0.09**	38	0.36	1	34
Diet:Route	**0.09**	38	0.36	1	34
IL-17A - Diet					
Diet	**1.00**	38	46.53	1	36
IL-17A - Route					
Route	**0.76**	38	7.41	1	36
IL-17A - Diet:Route					
Diet	**0.32**	38	2.37	1	34
Route	**0.32**	38	2.37	1	34
Diet:Route	**0.32**	38	2.37	1	34
IL-18 - Diet					
Diet	**0.97**	38	15.21	1	36
IL-18 - Route					
Route	**0.43**	38	3.34	1	36
IL-18 - Diet:Route					
Diet	**0.48**	38	3.91	1	34
Route	**0.48**	38	3.91	1	34
Diet:Route	**0.48**	38	3.91	1	34
IL-2 - Diet					
Diet	**0.67**	38	6.14	1	36
IL-2 - Route					
Route	**0.15**	38	0.89	1	36
IL-2 - Diet:Route					
Diet	**0.45**	38	3.53	1	34
Route	**0.45**	38	3.53	1	34

Diet:Route	**0.45**	38	3.53	1	34
IL-22 - Diet					
Diet	**0.38**	38	2.85	1	36
IL-22 - Route					
Route	**0.16**	38	1.00	1	36
IL-22 - Diet:Route					
Diet	**0.11**	38	0.53	1	34
Route	**0.11**	38	0.53	1	34
Diet:Route	**0.11**	38	0.53	1	34
IL-4 - Diet					
Diet	**1.00**	38	37.40	1	36
IL-4 - Route					
Route	**0.08**	38	0.29	1	36
IL-4 - Diet:Route					
Diet	**0.34**	38	2.57	1	34
Route	**0.34**	38	2.57	1	34
Diet:Route	**0.34**	38	2.57	1	34
IL-5 - Diet					
Diet	**0.39**	38	2.98	1	36
IL-5 - Route					
Route	**0.13**	38	0.74	1	36
IL-5 - Diet:Route					
Diet	**0.43**	38	3.37	1	34
Route	**0.43**	38	3.37	1	34
Diet:Route	**0.43**	38	3.37	1	34
IL-6 - Diet					
Diet	**0.99**	38	21.21	1	36
IL-6 - Route					
Route	**0.72**	38	6.82	1	36
IL-6 - Diet:Route					
Diet	**0.92**	38	11.82	1	34
Route	**0.92**	38	11.82	1	34

Diet:Route	**0.92**	38	11.82	1	34
TNF-alpha - Diet					
Diet	**0.88**	38	10.27	1	36
TNF-alpha - Route					
Route	**0.65**	38	5.82	1	36
TNF-alpha - Diet:Route					
Diet	**0.76**	38	7.46	1	34
Route	**0.76**	38	7.46	1	34
Diet:Route	**0.76**	38	7.46	1	34

Supplementary Figures

Figure S1

Panel a

Data analysis

In figure S1a, we present the longitudinal weekly observation of mouse body weight pre *Leishmania donovani* infection, to assess the impact of well-nourishing and malnourishing diets on the BALB/c mouse weight. Information of a total of N=89 BALB/c mice (WN=44, MN=45) over the course of 7 weeks are shown here; "Week_0" being the weight before shipment, "Week_7" being the final weight before infection.

We needed to analyze the data with a two-way approach to account for the two predictors, "Time_point" was the within-subject factor, while "Diet" was the between-subject factors in the analysis with "Weight_g" being the dependent outcome variable.

For a two-way mixed ANOVA, we had to assess the data for compliance with assumptions:

- Data normality
- Homogeneity of variance
- Homogeneity of Covariance
- No significant outliers
- Assumption of sphericity

Assumption analyses

Data normality

The assessment of the untransformed data distribution for each group was conducted by Shapiro-Wilks test and QQ-plot after splitting the data by both predictors. Note that all groups consisted of *N*=WN=44, MN=45 individuals, which made groups large enough to assess data distribution reliably by Shapiro-Wilks test. Thus, we performed the analyses by Shapiro-Wilks test (Appendix table 159) and QQ-plots (Fig.S1a-1) and found deviations from normality only at the Week_0 time point.

Appendix Table 160

Univariate Shapito-Wilks test results

Weeks p.i.	variable	statistic	p	Outcome
WN				
Week_0	Counts	0.8924	0.0006	sig.
Week_2	Counts	0.9701	0.3041	ns
Week_3	Counts	0.9667	0.2302	ns
Week_4	Counts	0.9807	0.6618	ns
Week_5	Counts	0.9857	0.8545	ns
Week_6	Counts	0.9873	0.9040	ns
Week_7	Counts	0.9852	0.8363	ns
MN				
Week_0	Counts	0.8879	0.0004	sig.
Week_2	Counts	0.9628	0.1555	ns
Week_3	Counts	0.9512	0.0565	ns
Week_4	Counts	0.9600	0.1218	ns
Week_5	Counts	0.9684	0.2521	ns
Week_6	Counts	0.9503	0.0522	ns
Week_7	Counts	0.9647	0.1847	ns

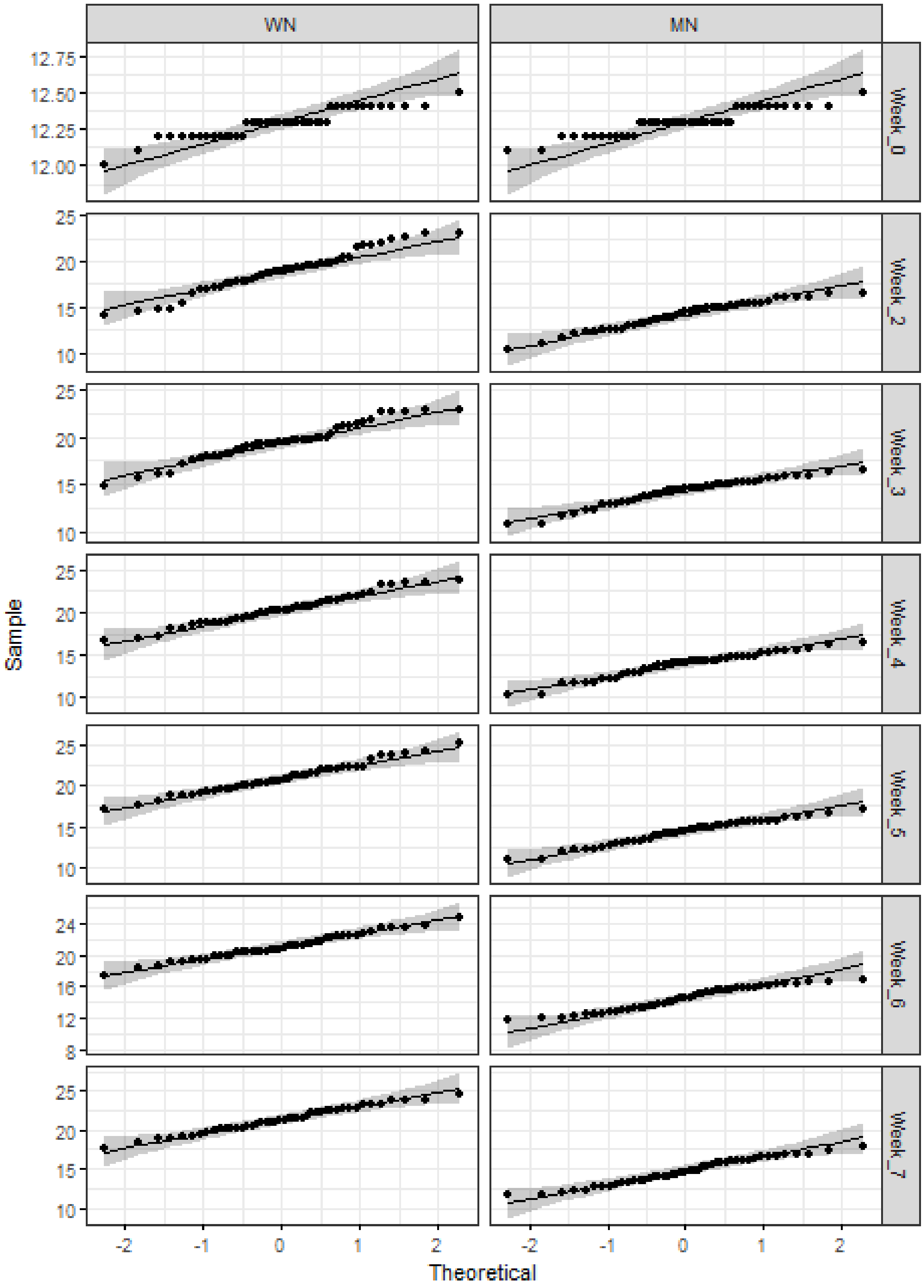

Fig.S1a-1: QQ-plots of mouse weights pre-infestation split into groups by predictor variables

Homogeneity of variance

The assessment of homogeneity of variance was conducted by Levene's test for the dataset split by the within-subject factor ("Time_point"). The analysis output showed that assumption of homogeneity between groups held for each week (Appendix table 160).

Appendix Table 161

Assessment of homogeneity of variance by week

Weeks p.i.	df1	df2	statistic	p	Sig.
Week_0	1	87	0.3565	0.5520	ns
Week_2	1	87	3.7582	0.0558	+
Week_3	1	87	2.5150	0.1164	ns
Week_4	1	87	2.0777	0.1531	ns
Week_5	1	87	1.2097	0.2744	ns
Week_6	1	87	0.0243	0.8765	ns
Week_7	1	87	0.2668	0.6068	ns

Outliers

We found no outliers in the data (Appendix table 161).

Appendix Table 162

List of possible outliers

Outcome
No outliers detected

Homogeneity of covariance

There was a statistically significant violation of the assumption of homogeneity of covariance as the p-value was <0.0001. We settled for a robust two-way ANOVA to buffer some of the effect of this violation, but only low p-values can be regarded as significant (Appendix table 162).

Appendix Table 163

Box's M-test for homogenetiy of covariance

statistic	p.value	parameter
173.6597	<0.0001	1

Sphericity

We ran a standard two-way ANOVA on the untransformed data to obtain information on the Mauchly's test of sphericity, which showed a clear violation of this assumption. This required the application of

Greenhouse-Geisser sphericity correction to the data (Appendix table 163).

Appendix Table 164

Mauchly's Test for Sphericity

Effect	W	p	p<.05
Time_point	0.001	<0.0001	*
Diet:Time_point	0.001	<0.0001	*

Two-way mixed analysis

Based on the assumption analysis, we decided to apply a Robust two-way mixed ANOVA to the dataset to determine the effects of "Diet" and time post infection ("Time_point") on mouse weight over time (Appendix table 164). The test output showed that both individual predictors were statistically significant, with the two-way interactions also being statistically significant.

Appendix Table 165

Robust two-way ANOVA

Predictor	value	p.value
Diet	1473.979	0.001
Time_point	4255.219	0.001
Diet:Time_point	1474.155	0.001

For the analysis of the simple main effect for each respective between-subject factor, we performed Robust one-way ANOVA with the between-subject factor ("Diet") split by the within-subject factor ("Time_point"). The results showed that "Diet" caused statistically significant differences with the exception of "Week_0" (Appendix table 165).

Appendix Table 166

Robust one-way ANOVA

Weeks p.i.	test	df1	df2	p.value	effsize	effsize_ci_lower	effsize_ci_upper	Sig.
Week_0	0.0800	1	70.5479	0.7781	0.1079	0.0000	0.3362	ns
Week_2	117.3500	1	59.3467	<0.0001	1.0245	0.9068	1.1081	****
Week_3	196.3361	1	62.4439	<0.0001	1.0807	1.0095	1.1399	****
Week_4	305.9447	1	65.4906	<0.0001	1.1164	1.0687	1.1530	****
Week_5	310.8601	1	68.4596	<0.0001	1.1196	1.0742	1.1517	****
Week_6	337.1300	1	70.9299	<0.0001	1.1099	1.0692	1.1457	****
Week_7	287.8534	1	70.8023	<0.0001	1.1042	1.0507	1.1431	****

For the pairwise comparison, we applied a Linear contrast expression (Appendix table 166). Since the "Diet" predictor only had two factor levels, the output showed the same result as the Robust one-way ANOVA above (Appendix table 165).

Appendix Table 167

Robust one-way ANOVA - sig. summary

Weeks p.i.	psihat	ci.lower	ci.upper	p.value	Sig.
Week_0	-0.0056	-0.0447	0.0336	0.7781	ns
Week_2	4.7384	3.8632	5.6135	<0.0001	****
Week_3	5.2222	4.4773	5.9671	<0.0001	****
Week_4	6.3616	5.6353	7.0878	<0.0001	****
Week_5	6.4700	5.7378	7.2021	<0.0001	****
Week_6	6.4950	5.7896	7.2003	<0.0001	****
Week_7	6.6050	5.8287	7.3813	<0.0001	****

Conclusion

In conclusion, "Diet" was a potent predictor for mouse weight gain over time pre-infestation.

Panel c

We analyzed a total of N=86 BALB/c mice (MN=43, WN=43). These were the same mice as analyzed in figure 2a for parasite dissemination events. Here, we quantified sand fly feeding success by counting the number of visibly fed sand flies. For the data analysis we tested several Poisson and negative binomial-type regression models. Based on the Akaike information criterion (AIC) we selected a standard poisson regression model for the data analysis post data re-scaling. The model fitted the data well producing no statistically significant departure from 1 for its dispersion ratio (chisq_statistic: 82.9122, dispersion_ratio: 0.987, residual_df: 84, p_value: 0.5131), but showing only a small pseudo-R^2 (Nagelkerke (Cragg and Uhler): 0.030404). The model output showed that "Diet" was not a statistically significant predictor, suggesting that the nutritional state of the mice did not impact the sand flies ability to feed on them (Appendix table 167).

Appendix Table 168

Negative binomial regression model output

Predictors	Estimate	Std. Error	z value	Pr(>|z|)	sig.
(Intercept)	2.3851	0.0463	51.5431	<0.0001	****
DietWN_SF	0.1036	0.0638	1.6243	0.1043	ns

The pairwise comparison based on the estimated marginal means showed that sand flies fed just as well on well-nourished animals as on malnourished animals excluding any unintended parasite inoculation bias

due to the nutritional state (Appendix table 168 and 169).

Appendix Table 169

Pairwise comparison based on estimated marginal means

Predictor pairs	estimate	SE	df	z.ratio	p.value	sig.
MN_SF - WN_SF	-0.1036	0.0638	Inf	-1.6243	0.1043	ns

Appendix Table 170

Pairwise comparison letter code

Diet	emmean	SE	df	asymp.LCL	asymp.UCL	.group
MN_SF	2.3851	0.0463	Inf	2.2814	2.4888	a
WN_SF	2.4888	0.0439	Inf	2.3903	2.5873	a

Figure S3

Panel c

Data analysis

Here, we present the statistical comparisons of spleen and liver weights (g), respectively, of a total of *N*=77 well-nourished (WN) or malnourished (MN) BALB/c mice, uninfected (Ctrl), infected by sand fly bite (SF) or by "needle" inoculation (Appendix table 170).

Appendix Table 171

Summary information

Targets	Total N	WN_Ctrl	MN_Ctrl	WN_Needle	MN_Needle	WN_SF	MN_SF	Transformation
Liver	77	15	10	10	11	15	16	untransformed
Spleen	77	15	10	10	11	15	16	untransformed

We needed to analyze the data with a two-way approach to account for the two predictors, "Diet" and "Route", both of which were between-subject factors.

For a two-way ANOVA, we had to assess the data for compliance with assumptions:

- Data normality
- Homogeneity of variance
- No significant outliers

Assumption analyses

Please, note that all assumption test results shown are post data transformation, where applicable (Appendix table 170).

Data normality

The assessment of the untransformed data distribution for each group was conducted by Shapiro-Wilks test and QQ-plot after splitting the data by both predictors. Note that all groups consisted of <30 data points (range[10 to 16]) (Appendix table 170), which made groups too small to assess data distribution reliably by Shapiro-Wilks test. We executed the test anyway as an indicator of gross departure of data normality. Thus, we performed the analyses by Shapiro-Wilks test (Appendix table 171) and QQ-plots (Fig.S3c-1). We found no departure of normality for the liver weights, but encountered minor deviation for the well-nourished, sand fly infected and malnourished control groups for the spleen weights, respectively. However, the QQ-plot suggested that all data points fell within the 95% confidence intervals, with the exception of the well-nourished, needle inoculated group's liver weights. Any attempt by data transformation resulted in more deviation from normality in the other groups, thus, we omitted any data transformation.

Appendix Table 172
Univariate Shapito-Wilks test results

Diet	Route	variable	statistic	p	Outcome
Liver					
WN	Ctrl	Counts	0.9589	0.6738	ns
WN	Needle	Counts	0.9128	0.3009	ns
WN	SF	Counts	0.9830	0.9861	ns
MN	Ctrl	Counts	0.9636	0.8261	ns
MN	Needle	Counts	0.9299	0.4102	ns
MN	SF	Counts	0.9789	0.9540	ns
Spleen					
WN	Ctrl	Counts	0.9389	0.3685	ns
WN	Needle	Counts	0.9168	0.3310	ns
WN	SF	Counts	0.8736	0.0381	sig.
MN	Ctrl	Counts	0.8065	0.0174	sig.
MN	Needle	Counts	0.8682	0.0735	ns
MN	SF	Counts	0.9107	0.1195	ns

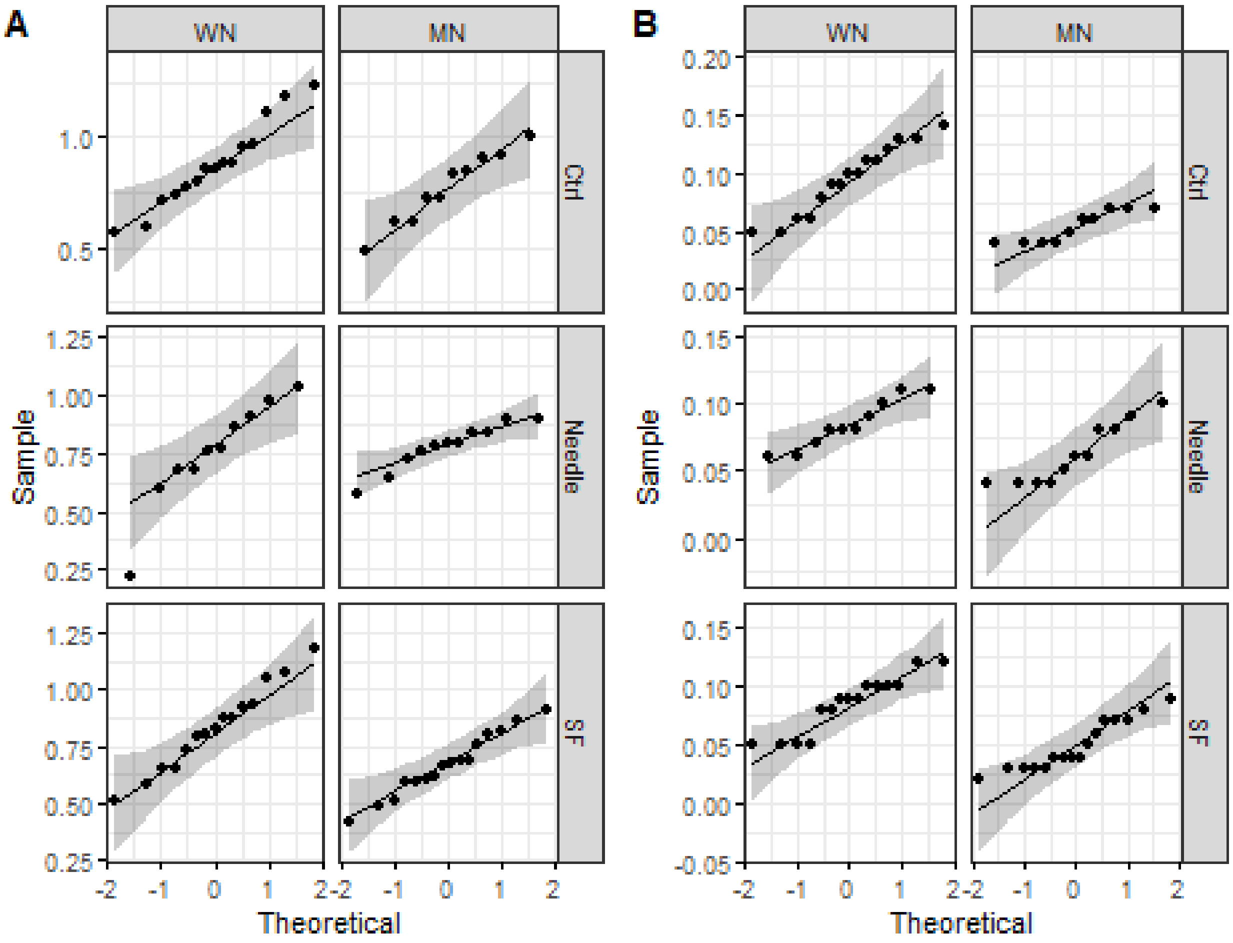

Fig.S3c-1: QQ-plots of serum chemistry targets' data: A) Liver and B) Spleen

Homogeneity of variance

The assessment of homogeneity of variance was conducted by Levene's test for the dataset. The analysis output showed that assumption of homogeneity between groups held for all organ weights (Appendix table 172).

Appendix Table 173

Assessment of homogeneity of variance by target

Targets	df1	df2	statistic	p	sig.
Liver	5	71	1.1097	0.3631	ns
Spleen	5	71	1.0924	0.3722	ns

Outliers

Conversely, we detected two outliers for the liver (Appendix table 173), non of which were extreme.

Appendix Table 174
List of possible outliers

Diet	Route	Tissue	is.outlier	is.extreme
Liver				
WN	Needle	Liver	TRUE	FALSE
MN	Needle	Liver	TRUE	FALSE

Two-way analysis

Based on the assumption analysis, we applied an appropriate two-way test to the respective datasets (Appendix table 174).

Appendix Table 175
Applied tests

Targets	Two-way test	One-way test	Pairwise
Liver	Standard two-way ANOVA	Standard one-way ANOVA	Estimated marginal means analysis
Spleen	Simple linear regression	Not applicable for linear regression	Estimated marginal means analysis

The respective two-way analyses showed that there was only statistical significant differences with respect to the animals nutritional state (Appendix table 175), while the infection route did not make any difference with respect to the organs' weight. There was also no statistically significant interaction between both predictors.

Appendix Table 176
Two-way analysis

Effect	DFn	DFd	Statistic	p.value	p<.05	ges
Liver						
Diet	1	71	5.4720	0.0220	*	0.072
Route	2	71	1.2640	0.2890		0.034
Diet:Route	2	71	1.8740	0.1610		0.050
Spleen						
Diet	1	71	42.3545	<0.0001	****	NA
Route	2	71	0.9552	0.3896		NA
Diet:Route	2	71	0.9608	0.3875		NA

The analysis of the simple main effect was only performed for the liver weights, as the spleen had been analyzed by linear regression. Each respective between-subject factor was used to perform a one-way

analyses with the data split always by the predictor that was not used as within the function. The results showed that statistical significance was only observed for the comparison of well-nourished and malnourished, sand fly infected mice (Table 176).

Appendix Table 177

Simple main effect analysis

Factor	Effect	statistic	df1	df2	p.value	Sig.	effsize
Liver							
WN	Route	1.578	2	71	0.214	ns	0.043
MN	Route	1.560	2	71	0.217	ns	0.042
Ctrl	Diet	2.373	1	71	0.128	ns	0.032
Needle	Diet	0.121	1	71	0.728	ns	0.002
SF	Diet	6.725	1	71	0.012	*	0.087

The pairwise comparison confirmed this observation for the liver weights showing only statistical significance for the well-nourished and malnourished, sand fly infected mice (Table 177). This suggested that only infection by sand fly altered the observed average liver weights, resulting in lower average weights in the malnourished group compared to their well-nourished counter part. This observation was also true for the spleen weights (Table 177). However, here the uninfected control groups also showed statistical significant difference in weights.

Appendix Table 178

Pairwise comparison

contrast	df	statistic	p.value	Sig.
Liver				
MN Ctrl - MN Needle	71	-0.1316	0.8957	ns
MN Ctrl - MN SF	71	1.3755	0.2600	ns
MN Needle - MN SF	71	1.5625	0.2600	ns
WN Ctrl - MN Ctrl	71	1.5403	0.1279	ns
WN Ctrl - WN Needle	71	1.7725	0.2418	ns
WN Ctrl - WN SF	71	0.6882	0.4936	ns
WN Needle - MN Needle	71	-0.3485	0.7285	ns
WN Needle - WN SF	71	-1.1570	0.3767	ns
WN SF - MN SF	71	2.5933	0.0115	*
Spleen				
MN Ctrl - MN Needle	71	-0.0078	0.9998	ns
MN Ctrl - MN SF	71	0.0046	1.0000	ns

MN Ctrl - WN Needle	71	-0.0300	0.0652	+
MN Needle - MN SF	71	0.0124	0.9376	ns
MN Needle - WN SF	71	-0.0228	0.1911	ns
WN Ctrl - MN Ctrl	71	0.0407	0.0007	***
WN Ctrl - MN Needle	71	0.0328	0.0082	**
WN Ctrl - WN Needle	71	0.0107	0.9883	ns
WN Ctrl - WN SF	71	0.0100	0.9819	ns
WN Needle - MN Needle	71	0.0222	0.3620	ns
WN Needle - MN SF	71	0.0346	0.0052	**
WN Needle - WN SF	71	-0.0007	1.0000	ns
WN SF - MN SF	71	0.0353	0.0008	***

Statistical power

Considering the small group sizes (Appendix tables 170), we wanted to ensure that the study design was not significantly statistically underpowered. Thus, we performed a retrospective sample size and power analysis on the data by target.

Effect size estimation based on partial eta^2

Effect sizes were calculated by predictor and the different potential interaction combinations of them. Appendix tables 178 shows the respective effect sizes. Note that upper ends of the confidence intervals were automatically set to 1 for this type of calculation. Large effect sizes reflected statistically meaningful differences in the data analysis. The partial eta^2 values from the effect size calculation were then used for the retrospective power calculations.

Appendix Table 179

Effect size

Parameter	Eta2_partial	CI_low	CI_high	Effect Size
Liver				
Diet	0.0543	0.0004	1	small
Route	0.0322	0.0000	1	small
Diet:Route	0.0501	0.0000	1	small
Spleen				
Diet	0.3479	0.2055	1	large
Route	0.0220	0.0000	1	small
Diet:Route	0.0264	0.0000	1	small

Retrospective minimum total sample size estimation for 80% power

The accepted rule of thumb is to have at least 80% (0.8 as ratio) statistical power in once data. For small mean differences within data of a high level of complexity, this is often hard to achieve, because of cost and ability to manage large sample sizes. In most instances, proposed sample sizes based on our data, suggested that actual sample sizes were frequently too small for a chance of detecting statistical significant differences, particularly, for the interaction of predictors (Appendix tables 179).

Appendix Table 180

Minimum optimal sample size calculation

Effect	power	n.total	ncp	df1	df2
Liver - Diet					
Diet	0.8	**139**	7.96	1	136.66
Liver - Route					
Route	0.8	**293**	9.73	2	289.22
Liver - Diet:Route					
Diet	0.8	**151**	7.95	1	144.71
Route	0.8	**186**	9.80	2	179.62
Diet:Route	0.8	**186**	9.80	2	179.62
Spleen - Diet					
Diet	0.8	**17**	9.00	1	14.87
Spleen - Route					
Route	0.8	**432**	9.70	2	428.82
Spleen - Diet:Route					
Diet	0.8	**292**	7.90	1	285.95
Route	0.8	**360**	9.72	2	353.01
Diet:Route	0.8	**360**	9.72	2	353.01

Retrospective calculation of statistical power in our data analysis

We generally observed that our study was underpowered for the detection of a statistical significant difference, in particular, for the infection route and the interaction with "Diet" (Appendix Table 180). Even so, excessively large proposed sample sizes and small retrospective statistical power can also be an indicator that there is in fact no meaningful biological difference to be found. Considering that statistical significant difference were still found, suggested that the above observations were real.

Appendix Table 181
Statistical power of data

Effect	power	n.total	ncp	df1	df2
Liver - Diet					
Diet	**0.55**	77	4.42	1	75
Liver - Route					
Route	**0.27**	77	2.56	2	74
Liver - Diet:Route					
Diet	**0.51**	77	4.06	1	71
Route	**0.41**	77	4.06	2	71
Diet:Route	**0.41**	77	4.06	2	71
Spleen - Diet					
Diet	**1.00**	77	41.09	1	75
Spleen - Route					
Route	**0.19**	77	1.73	2	74
Spleen - Diet:Route					
Diet	**0.30**	77	2.08	1	71
Route	**0.22**	77	2.08	2	71
Diet:Route	**0.22**	77	2.08	2	71

References

[1] J. J. Allaire et al. *rmarkdown: Dynamic Documents for R*. 2024. URL: https://github.com/rstudio/rmarkdown.

[2] JJ Allaire et al. *rmarkdown: Dynamic Documents for R*. R package version 2.27. 2024. URL: https://github.com/rstudio/rmarkdown.

[3] Tomas J. Aragon. *epitools: Epidemiology Tools*. R package version 0.5-10.1. 2020. URL: https://CRAN.R-project.org/package=epitools.

[4] Mattan S. Ben-Shachar, Daniel Lüdecke, and Dominique Makowski. "effectsize: Estimation of Effect Size Indices and Standardized Parameters". In: *Journal of Open Source Software* 5.56 (2020), p. 2815. DOI: 10.21105/joss.02815[3]. URL: https://doi.org/10.21105/joss.02815.

[5] Metin Bulus. *pwrss: Statistical Power and Sample Size Calculation Tools*. R package version 0.3.1. 2023. URL: https://CRAN.R-project.org/package=pwrss.

[3] https://doi.org/10.21105/joss.02815

[6] Francisco Cribari-Neto and Achim Zeileis. "Beta Regression in R". In: *Journal of Statistical Software* 34.2 (2010), pp. 1–24. DOI: 10.18637/jss.v034.i02[4].

[7] Sam Firke. *janitor: Simple Tools for Examining and Cleaning Dirty Data*. R package version 2.2.0. 2023. URL: https://CRAN.R-project.org/package=janitor.

[8] John Fox and Sanford Weisberg. *An R Companion to Applied Regression*. Third. Thousand Oaks CA: Sage, 2019. URL: https://socialsciences.mcmaster.ca/jfox/Books/Companion/.

[9] Marek Gagolewski. "stringi: Fast and portable character string processing in R". In: *Journal of Statistical Software* 103.2 (2022), pp. 1–59. DOI: 10.18637/jss.v103.i02[5].

[10] Philippe Grosjean and Frederic Ibanez. *pastecs: Package for Analysis of Space-Time Ecological Series*. R package version 1.4.2. 2024. URL: https://CRAN.R-project.org/package=pastecs.

[11] Bettina Grün, Ioannis Kosmidis, and Achim Zeileis. "Extended Beta Regression in R: Shaken, Stirred, Mixed, and Partitioned". In: *Journal of Statistical Software* 48.11 (2012), pp. 1–25. DOI: 10.18637/jss.v048.i11[6].

[12] Frank E Harrell Jr. *Hmisc: Harrell Miscellaneous*. R package version 5.1-3. 2024. URL: https://CRAN.R-project.org/package=Hmisc.

[13] Torsten Hothorn, Frank Bretz, and Peter Westfall. "Simultaneous Inference in General Parametric Models". In: *Biometrical Journal* 50.3 (2008), pp. 346–363.

[14] Alboukadel Kassambara. *ggpubr: 'ggplot2' Based Publication Ready Plots*. R package version 0.6.0. 2023. URL: https://CRAN.R-project.org/package=ggpubr.

[15] Alboukadel Kassambara. *rstatix: Pipe-Friendly Framework for Basic Statistical Tests*. R package version 0.7.2. 2023. URL: https://CRAN.R-project.org/package=rstatix.

[16] Lukasz Komsta and Frederick Novomestky. *moments: Moments, Cumulants, Skewness, Kurtosis and Related Tests*. R package version 0.14.1. 2022. URL: https://CRAN.R-project.org/package=moments.

[17] Ioannis Kosmidis and Achim Zeileis. *Extended-Support Beta Regression for [0, 1] Responses*. Unpublished Manuscript. 2024.

[18] Kuhn and Max. "Building Predictive Models in R Using the caret Package". In: *Journal of Statistical Software* 28.5 (2008), pp. 1–26. DOI: 10.18637/jss.v028.i05[7]. URL: https://www.jstatsoft.org/index.php/jss/article/view/v028i05.

[19] Russell V. Lenth. *emmeans: Estimated Marginal Means, aka Least-Squares Means*. R package version 1.10.3. 2024. URL: https://CRAN.R-project.org/package=emmeans.

[20] Lesnoff et al. *aod: Analysis of Overdispersed Data*. R package version 1.3.3. 2012. URL: https://cran.r-project.org/package=aod.

[21] Daniel Lüdecke. "sjmisc: Data and Variable Transformation Functions." In: *Journal of Open Source Software* 3.26 (2018), p. 754. DOI: 10.21105/joss.00754[8].

[4] https://doi.org/10.18637/jss.v034.i02
[5] https://doi.org/10.18637/jss.v103.i02
[6] https://doi.org/10.18637/jss.v048.i11
[7] https://doi.org/10.18637/jss.v028.i05
[8] https://doi.org/10.21105/joss.00754

[22] Daniel Lüdecke et al. "performance: An R Package for Assessment, Comparison and Testing of Statistical Models". In: *Journal of Open Source Software* 6.60 (2021), p. 3139. DOI: 10.21105/joss.03139[9].

[23] Patrick Mair and Rand Wilcox. "Robust Statistical Methods in R Using the WRS2 Package". In: *Behavior Research Methods* 52 (2020). DOI: 10.3758/s13428-019-01246-w[10].

[24] Salvatore S. Mangiafico. *rcompanion: Functions to Support Extension Education Program Evaluation*. version 2.4.36. Rutgers Cooperative Extension. New Brunswick, New Jersey, 2024. URL: https://CRAN.R-project.org/package=rcompanion/.

[25] Tiago Olivoto and Alessandro Dal'Col Lúcio. "metan: An R package for multi-environment trial analysis". In: *Methods in Ecology and Evolution* 11.6 (2020), pp. 783–789. DOI: 10.1111/2041-210X.13384[11].

[26] José Pinheiro, Douglas Bates, and R Core Team. *nlme: Linear and Nonlinear Mixed Effects Models*. R package version 3.1-165. 2024. URL: https://CRAN.R-project.org/package=nlme.

[27] José C. Pinheiro and Douglas M. Bates. *Mixed-Effects Models in S and S-PLUS*. New York: Springer, 2000. DOI: 10.1007/b98882[12].

[28] R Core Team. *R: A Language and Environment for Statistical Computing*. R Foundation for Statistical Computing. Vienna, Austria, 2024. URL: https://www.R-project.org/.

[29] R Core Team. *R: A Language and Environment for Statistical Computing*. R Foundation for Statistical Computing. Vienna, Austria, 2024. URL: https://www.R-project.org/.

[30] Xavier Robin et al. "pROC: an open-source package for R and S+ to analyze and compare ROC curves". In: *BMC Bioinformatics* 12 (2011), p. 77.

[31] Francisco Rodriguez-Sanchez and Connor P. Jackson. *grateful: Facilitate citation of R packages*. 2023. URL: https://pakillo.github.io/grateful/.

[32] RStudio Team. *RStudio: Integrated Development for R*. Type: Computer Program. RStudio, PBC., 2023. URL: http://www.rstudio.com/..

[33] Simon Urbanek and Kent Johnson. *tiff: Read and Write TIFF Images*. R package version 0.1-12. 2023. URL: https://CRAN.R-project.org/package=tiff.

[34] W. N. Venables and B. D. Ripley. *Modern Applied Statistics with S*. Fourth. ISBN 0-387-95457-0. New York: Springer, 2002. URL: https://www.stats.ox.ac.uk/pub/MASS4/.

[35] Hadley Wickham. *conflicted: An Alternative Conflict Resolution Strategy*. R package version 1.2.0. 2023. URL: https://CRAN.R-project.org/package=conflicted.

[36] Hadley Wickham et al. "Welcome to the tidyverse". In: *Journal of Open Source Software* 4.43 (2019), p. 1686. DOI: 10.21105/joss.01686[13].

[37] Yihui Xie. *bookdown: Authoring Books and Technical Documents with R Markdown*. Boca Raton, Florida: Chapman and Hall/CRC, 2016. ISBN: 978-1138700109. URL: https://bookdown.org/yihui/bookdown.

[9] https://doi.org/10.21105/joss.03139

[10] https://doi.org/10.3758/s13428-019-01246-w

[11] https://doi.org/10.1111/2041-210X.13384

[12] https://doi.org/10.1007/b98882

[13] https://doi.org/10.21105/joss.01686

[38] Yihui Xie. *bookdown: Authoring Books and Technical Documents with R Markdown*. R package version 0.40. 2024. URL: https://github.com/rstudio/bookdown.

[39] Yihui Xie. *Dynamic Documents with R and knitr*. 2nd. ISBN 978-1498716963. Boca Raton, Florida: Chapman and Hall/CRC, 2015. URL: https://yihui.org/knitr/.

[40] Yihui Xie. "knitr: A Comprehensive Tool for Reproducible Research in R". In: *Implementing Reproducible Computational Research*. Ed. by Victoria Stodden, Friedrich Leisch, and Roger D. Peng. ISBN 978-1466561595. Chapman and Hall/CRC, 2014.

[41] Yihui Xie. *knitr: A General-Purpose Package for Dynamic Report Generation in R*. R package version 1.48. 2024. URL: https://yihui.org/knitr/.

[42] Yihui Xie, J.J. Allaire, and Garrett Grolemund. *R Markdown: The Definitive Guide*. Boca Raton, Florida: Chapman and Hall/CRC, 2018. ISBN: 9781138359338. URL: https://bookdown.org/yihui/rmarkdown.

[43] Yihui Xie, Christophe Dervieux, and Emily Riederer. *R Markdown Cookbook*. Boca Raton, Florida: Chapman and Hall/CRC, 2020. ISBN: 9780367563837. URL: https://bookdown.org/yihui/rmarkdown-cookbook.

[44] Achim Zeileis and Torsten Hothorn. "Diagnostic Checking in Regression Relationships". In: *R News* 2.3 (2002), pp. 7–10. URL: https://CRAN.R-project.org/doc/Rnews/.
