# Supplementary material for: Malnutrition exacerbates pathogenesis of Lutzomyia longipalpis sand fly-transmitted Leishmania donovani
Source: Commun Biol. 2025 May 13;8:746. doi: 10.1038/s42003-025-08106-8 (PMC12075822; doi:10.1038/s42003-025-08106-8)
Supplement: Supplementary file 5 — Reporting Summary [file 42003_2025_8106_MOESM5_ESM.pdf]

nature portfolio

Corresponding author(s): Kamhawi, COMMSBIO-24-6178-T

Last updated by author(s): 2025/07/04

Reporting Summary

Nature Portfolio wishes to improve the reproducibility of the work that we publish. This form provides structure for consistency and transparency in reporting. For further information on Nature Portfolio policies, see our Editorial Policies and the Editorial Policy Checklist.

Statistics

For all statistical analyses, confirm that the following items are present in the figure legend, table legend, main text, or Methods section.

n/a	Confirmed	
☐	☒	The exact sample size (*n*) for each experimental group/condition, given as a discrete number and unit of measurement
☐	☒	A statement on whether measurements were taken from distinct samples or whether the same sample was measured repeatedly
☐	☒	The statistical test(s) used AND whether they are one- or two-sided<br>*Only common tests should be described solely by name; describe more complex techniques in the Methods section.*
☐	☒	A description of all covariates tested
☐	☒	A description of any assumptions or corrections, such as tests of normality and adjustment for multiple comparisons
☐	☒	A full description of the statistical parameters including central tendency (e.g. means) or other basic estimates (e.g. regression coefficient) AND variation (e.g. standard deviation) or associated estimates of uncertainty (e.g. confidence intervals)
☐	☒	For null hypothesis testing, the test statistic (e.g. *F*, *t*, *r*) with confidence intervals, effect sizes, degrees of freedom and *P* value noted<br>*Give P values as exact values whenever suitable.*
☒	☐	For Bayesian analysis, information on the choice of priors and Markov chain Monte Carlo settings
☒	☐	For hierarchical and complex designs, identification of the appropriate level for tests and full reporting of outcomes
☐	☒	Estimates of effect sizes (e.g. Cohen's *d*, Pearson's *r*), indicating how they were calculated

Our web collection on statistics for biologists contains articles on many of the points above.

Software and code

Policy information about availability of computer code

Data collection	Statistical analysis was performed using R software.
Data analysis	R code report for stadistical analysis be access at https://github.com/joedoehl/Malnutrition-exacerbates-pathogenesis-of-sand-fly-transmitted-Leishmania-donovani.git. Code was deposited in a DOI-minting repository: 10.5281/zenodo.15127944.

For manuscripts utilizing custom algorithms or software that are central to the research but not yet described in published literature, software must be made available to editors and reviewers. We strongly encourage code deposition in a community repository (e.g. GitHub). See the Nature Portfolio guidelines for submitting code & software for further information.

Data

Policy information about availability of data

All manuscripts must include a data availability statement. This statement should provide the following information, where applicable:

- Accession codes, unique identifiers, or web links for publicly available datasets
- A description of any restrictions on data availability
- For clinical datasets or third party data, please ensure that the statement adheres to our policy

All data are available as supplementary data files. Statistical analysis and R code report can be access at https://github.com/joedoehl/Malnutrition-exacerbates-pathogenesis-of-sand-fly-transmitted-Leishmania-donovani.git. 16S DNA FASTQ sequencing files have been uploaded to the NCBI BioProject database and can be accessed at http://www.ncbi.nlm.nih.gov/bioproject/1245452 with accession number PRJNA1245452.

Research involving human participants, their data, or biological material

Policy information about studies with human participants or human data. See also policy information about sex, gender (identity/presentation), and sexual orientation and race, ethnicity and racism.

Reporting on sex and gender	N/A
Reporting on race, ethnicity, or other socially relevant groupings	N/A
Population characteristics	N/A
Recruitment	N/A
Ethics oversight	N/A

Note that full information on the approval of the study protocol must also be provided in the manuscript.

Field-specific reporting

Please select the one below that is the best fit for your research. If you are not sure, read the appropriate sections before making your selection.

☒ Life sciences ☐ Behavioural & social sciences ☐ Ecological, evolutionary & environmental sciences

For a reference copy of the document with all sections, see nature.com/documents/nr-reporting-summary-flat.pdf

Life sciences study design

All studies must disclose on these points even when the disclosure is negative.

Sample size	Statistical analysis was performed using R software. + P ≤ 0.9, *P ≤ 0.05, **P ≤ 0.01, ***P ≤ 0.001, and ****P ≤ 0.0001. Refer to supplementary material for the full statistical report for details on how the statistical analysis of the data were conducted. Details for sample sizes and number of replicates were indicated in the figure legend.
Data exclusions	All data from this study is included.
Replication	All experiments were conducted independently in 2 or more biological replicates.
Randomization	Post-weaning, three-week-old BALB/c mice were assigned randomly to a diet group.
Blinding	All experiments were conducted blinded, and the blinded code was broken post-data analysis.

Reporting for specific materials, systems and methods

We require information from authors about some types of materials, experimental systems and methods used in many studies. Here, indicate whether each material, system or method listed is relevant to your study. If you are not sure if a list item applies to your research, read the appropriate section before selecting a response.

Materials & experimental systems

n/a	Involved in the study	
☐	☒	Antibodies
☒	☐	Eukaryotic cell lines
☒	☐	Palaeontology and archaeology
☐	☒	Animals and other organisms
☒	☐	Clinical data
☒	☐	Dual use research of concern
☒	☐	Plants

Methods

n/a	Involved in the study	
☒	☐	ChIP-seq
☐	☒	Flow cytometry
☒	☐	MRI-based neuroimaging

Antibodies

Antibodies used	anti-mouse CD16/32 (TruStain FcX, clone 93; Biolegend, Cat No. 101320), PE-Cy7 CD11b (clone: M1/70; Biolegend, Cat No. 101216), Alexa Fluor 700 Ly6C (clone HK1.4; Biolegend, Cat No. 128024), PerCP-eFluor 710-Ly6G (clone: 1A; eBioscience, Cat No. 46966882),

and APC IL1-B pro-form (clone: NJTEN3; eBioscience, Cat No. 17-7114-80).

Validation	All antibodies were validated by the company for flow cytometry analysis for mouse species.

Animals and other research organisms

Policy information about studies involving animals; ARRIVE guidelines recommended for reporting animal research, and Sex and Gender in Research

Laboratory animals	Three-week-old female BALB/c mice were purchased from Jackson Laboratories. Four to six-week-old male Golden Syrian hamsters were purchased from Harlan Laboratories.
Wild animals	N/A
Reporting on sex	Three-week-old female BALB/c mice were purchased from Jackson Laboratories. Four to six-week-old male Golden Syrian hamsters were purchased from Harlan Laboratories.
Field-collected samples	N/A
Ethics oversight	All animal experiments were approved by NIAID Animal Care and Use Committee under the LMV23E and LMVR4E animal protocols. The NIAID DIR Animal Care and Use Program complies with the Guide for the Care and Use of Laboratory Animals and with the NIH Office of Animal Care and Use and Animal Research Advisory Committee guidelines.

Note that full information on the approval of the study protocol must also be provided in the manuscript.

Plants

Seed stocks	N/A
Novel plant genotypes	N/A
Authentication	N/A

Flow Cytometry

Plots

Confirm that:

☒ The axis labels state the marker and fluorochrome used (e.g. CD4-FITC).

☒ The axis scales are clearly visible. Include numbers along axes only for bottom left plot of group (a 'group' is an analysis of identical markers).

☒ All plots are contour plots with outliers or pseudocolor plots.

☒ A numerical value for number of cells or percentage (with statistics) is provided.

Methodology

Sample preparation	At 24 h and 72 h post-infection, mice ears were collected and processed as previously described16. Briefly, single cell suspensions were stained with the LIVE/DEAD fixable Aqua dead cell marker (Thermo-Fisher Scientific, L34957, 1:2000 dilution), anti-mouse CD16/32 (TruStain FcX, clone 93; Biolegend, Cat No. 101320, 1:50 dilution), PE-Cy7 CD11b (clone: M1/70; Biolegend, Cat No. 101216), Alexa Fluor 700 Ly6C (clone HK1.4; Biolegend, Cat No. 128024), and PerCP-eFluor 710-Ly6G (clone: 1A; eBioscience, Cat No. 46966882) for 30 min. Cells were fixed (Biolegend, Cat No. 420801) and permeabilized (Biolegend, Cat No. 421002), followed by intracellular staining with APC IL1- pro-form (clone: NJTEN3; eBioscience, Cat No. 17-7114-80) overnight. All antibodies were used at 1:100 dilution. All cells were acquired using MACSQuant 16 (Miltenyi Biotec) and data analyzed using FlowJo 10 software. Gates were established using Fluorescence Minus One (FMO) and unstained controls.
Instrument	All cells were acquired using MACSQuant 16 (Miltenyi Biotec)
Software	Data analyzed using FlowJo 10 software
Cell population abundance	All cells were acquired for flow cytometry experiments and the number of cells reported. No sorting experiments were performed in this study.

Gating strategy

Gates were established using Fluorescence Minus One (FMO) and unstained controls. Gating strategy reported in Figure S1.

☒ Tick this box to confirm that a figure exemplifying the gating strategy is provided in the Supplementary Information.
